# Straightforward Access to the Dispirocyclic Framework via Regioselective Intramolecular Michael Addition

**DOI:** 10.3390/molecules30153164

**Published:** 2025-07-29

**Authors:** Weilun Cao, Junmin Dong, Xuan Pan, Zhanzhu Liu

**Affiliations:** State Key Laboratory of Bioactive Substances and Functions of Natural Medicines, Institute of Materia Medica, Peking Union Medical College and Chinese Academy of Medical Sciences, Beijing 100050, China; caoweilun@imm.ac.cn (W.C.); dongjunmin@imm.ac.cn (J.D.)

**Keywords:** spiro compounds, regioselectivity, transition-metal free

## Abstract

In this article, an efficient and straightforward protocol for the construction of complex dispirocyclic skeletons via regioselective intramolecular Michael addition is presented. Diverse dispirocyclic compounds were synthesized under mild and transition-metal-free conditions with good to excellent yields. Most stereoisomers were conveniently separated by column chromatography, and their relative configurations were identified by single-crystal X-Ray diffraction of representative compounds. A scale-up experiment validated the practicality of this method. In an in vitro assay, some dispirocyclic compounds exhibited potent cytotoxicity with an IC_50_ value of 10^−6^ mol/L.

## 1. Introduction

**Figure 1:**
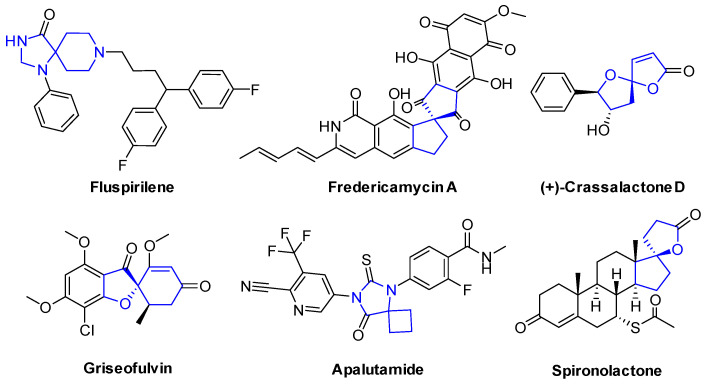
Spiro compounds occur in natural products and pharmaceutical molecules.

Spirocyclic scaffolds occupy a prominent position in medicinal chemistry and represent a major focus in contemporary drug discovery [1]. These structures are widely distributed in various natural products and pharmaceutical molecules [1,2,3,4,5,6] (Figure 1). Owing to their relatively high content of sp^3^-hybridized carbons and exceptional three-dimensional expandability, spiro compounds are highly attractive for drug discovery [7].

In this regard, several efficient methods have been developed, including NHC-catalyzed [3 + 4] annulation [8], Michael–Michael cascade [9,10], organocatalytic cascade [11,12], and transition metal catalytic procedures [13]. Nevertheless, reports on the synthesis of dispirocyclic skeletons remain relatively limited to date [14,15,16]. Therefore, a simple and practical strategy for the construction of dispirocycles is highly desired.

**Figure 2:**
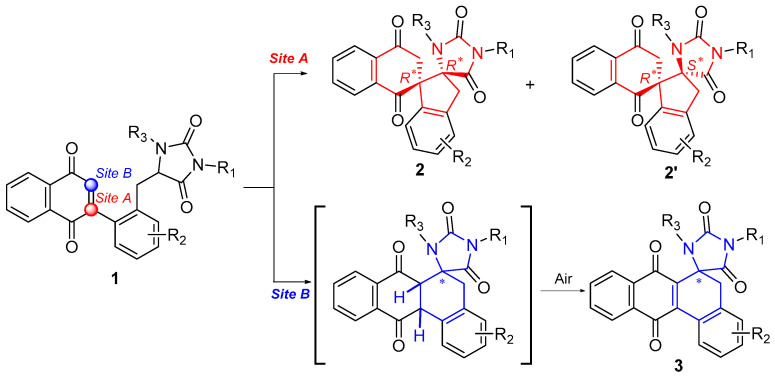
Regioselective intramolecular Michael addition of quinones to construct dispirocyclic or fused polycyclic skeletons.

Herein, we report a facile and efficient approach to rapidly construct dispirocyclic compounds from quinones via highly regioselective intramolecular Michael addition (Figure 2). The protocol proceeds under very mild conditions with a good functional group compatibility. A total of 68 dispirocyclic compounds were synthesized in good to excellent yields, and in nearly all the cases, stereoisomers were conveniently separated by column chromatography. The preliminary cytotoxicity of the synthetic compounds was also evaluated.

## 2. Results and Discussion

### 2.1. Synthesis

#### 2.1.1. Conditional Screening

Our study commenced with the treatment of naphthoquinone **1a** [17] with LiHMDS in THF at −78 °C for 1 h. Under these conditions, Michael addition proceeded in a non-regioselective manner. When the reaction took place at site A, compound **2a** was obtained, and when it occurred at site B, the resulting product was oxidized by air during workup to yield compound **3a**, with yields of 18% and 25%, respectively (Table 1, entry 1). The structure of compound **2a** was identified by its ^1^H NMR, ^13^C NMR spectra, and HRMS. The relative (R^*^, R^*^) configuration was determined by single-crystal X-Ray diffraction analysis (CCDC 2452353). However, to our disappointment, compound **3a** was difficult to purify since it was accompanied by a few inseparable impurities despite our tedious purification efforts. The utilization of NaHMDS decreased the yield of dispirocycle **2a** (entry 2). Exposure of substrate **1a** to KHMDS or *n-*BuLi resulted in a complete loss of dispirocyclic products (entries 3 and 4), indicating that strong bases were disadvantageous for this transformation. Moreover, compound **1a** was recovered completely in the presence of DBU or NaH in THF at room temperature for 3 h (entries 5 and 6).

To our surprise, NaOH emerged as an effective base for this intramolecular Michael addition, and a total yield of 65% was achieved in the presence of NaOH in CH_3_OH at room temperature for 3 h (entry 7). Moreover, in this case, we obtained another new product **2′a** (33%) apart from **2a** (18%) and **3a** (14%), which was the major product of this reaction. Compound **2′a** was identified as a stereoisomer of compound **2a** by comparison of their NMR spectra and HRMS. Treatment of compound **1a** with KOH gave similar results compared with those in the presence of NaOH (entry 8). A slight decrease in yield was observed upon treatment with LiOH (entry 9). Whereas the employment of CH_3_ONa led to a reduced yield of compounds **2a**/**2′a** and an improved yield of naphthoquinone **3a** (entry 10). A complete decomposition of **1a** was observed in the presence of *t*BuOK in *t*BuOH at room temperature (entry 11). Subsequent reaction of compound **1a** with carbonates led to the formation of three products with an overall yield from 20% to 50% (entries 12–14).

Based on the above results, NaOH was identified as the optimal base. However, it is noteworthy that compound **1a** exhibited poor solubility in CH_3_OH, which presumably resulted in a relatively low concentration of the substrate in this reaction. Consequently, a brief solvent screening was conducted (entries 15–18). Initially, the reaction was performed in CH_2_Cl_2_ or THF at room temperature for 3 h, which afforded all three products with total yields of approximately 45% and 48%, respectively (entries 15 and 16). The solubility of substrate **1a** in CH_2_Cl_2_ and THF was generally acceptable, whereas the yields of compound **2a** and **2′a** were still unsatisfactory, which was probably attributed to the low solubility of NaOH. An attempt to increase the temperature to improve the solubility of **1a** failed; instead, this shifted the regioselectivity of Michael addition toward the formation of naphthoquinone **3a,** accompanied by a few inseparable impurities (entry 17). Finally, a mixture of CH_2_Cl_2_ and CH_3_OH (5:1, *v*/*v*) was employed, which provided satisfactory solubility for both **1a** and NaOH. After 1 h, the total yield of **2a**, **2′a**, and **3a** achieved 82% (Entry 18).

Further investigation revealed that temperature significantly influenced the regioselectivity of this Michael addition. When the reaction was conducted at −20 °C, dispirocyclic products **2a** (35%) and **2′a** (58%) were afforded in 93% total yield with no detectable naphthoquinone **3a** (entry 20). Additionally, we also tried to reduce the amount of NaOH utilized in this reaction, and found that the Michael addition reaction could still proceed smoothly under catalytic amounts of NaOH (0.2 eq.) (entry 21).

#### 2.1.2. Substrate Expansion

After systematic exploration, the optimal reaction conditions were identified as the treatment of quinone **1** with a catalytic amount of NaOH (0.2 eq.) in a mixture of CH_2_Cl_2_ and CH_3_OH (5:1) at −20 °C, which exclusively afforded dispirocycles in excellent yields.

**Figure 3:**
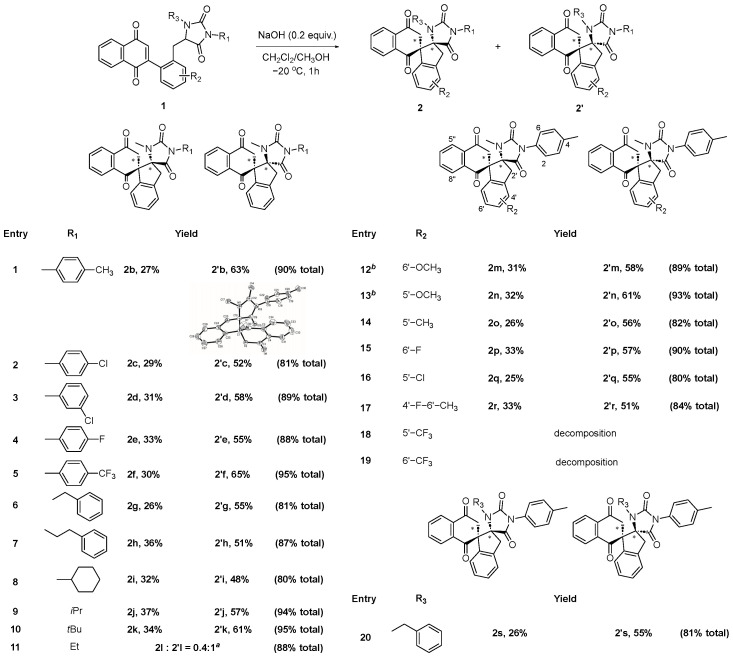
Investigation of substrate scope for the naphthoquinone derivatives. Treatment of compound **1** with a catalytic amount of NaOH (0.2 eq.) in a mixture of CH_2_Cl_2_ and CH_3_OH (5:1) at −20 °C. *^a^* Determined by ^1^H NMR. *^b^* Reaction was completed within 20 min.

With the optimized reaction conditions in hand, we then turned our attention to explore the substrate scope of this regioselective Michael addition to assemble structurally diverse dispirocyclic compounds (Figure 3). Initially, the variation in R_1_ was surveyed. As listed in Table 2, a series of phenyl rings bearing electron-withdrawing or electron-donating groups were well tolerated in this reaction, generating the corresponding dispirocyclic products in good to excellent total yields (81–95%) (entries 1–5). When R_1_ was benzyl, phenylethyl, or alkyl, compounds 1g-l were smoothly converted to the desired dispirocyclic products in good yields (81–95%) (entries 6–11), indicating that R_1_ exerted little if any influence on the transformation.

Subsequently, the effect of R_2_ substituents was studied. As illustrated in Table 2, the Michael addition worked well with various electron-donating and weak electron-withdrawing substrates, and in all cases, the desired products were obtained in good to excellent yields (80–93%) (entries 12–17). It was noteworthy that the reaction of substrates with an electron-donating group was much faster, usually being completed within 20 min with comparable yields (entries 12 and 13). However, the reaction failed when R_2_ was trifluoromethyl, which was a strong electron-withdrawing group (entries 18 and 19). Additionally, we investigated the influence of R_3_ substituents: compound **1s** bearing a benzyl as R_2_ substituent underwent efficient Michael addition to deliver dispirocyclic products **2s**/**2′s** in 81% total yield (entry 20).

In most cases, stereoisomers **2** and **2′** could be conveniently separated by column chromatography, except for **2l** and **2′l**. Dispirocyclic products **2′** were always the predominant products with an approximate *d.r.* value of 1:2. The relative configuration of compound **2′** was determined by single-crystal X-Ray diffraction analysis of **2′b** (CCDC 2372028).

**Figure 4:**
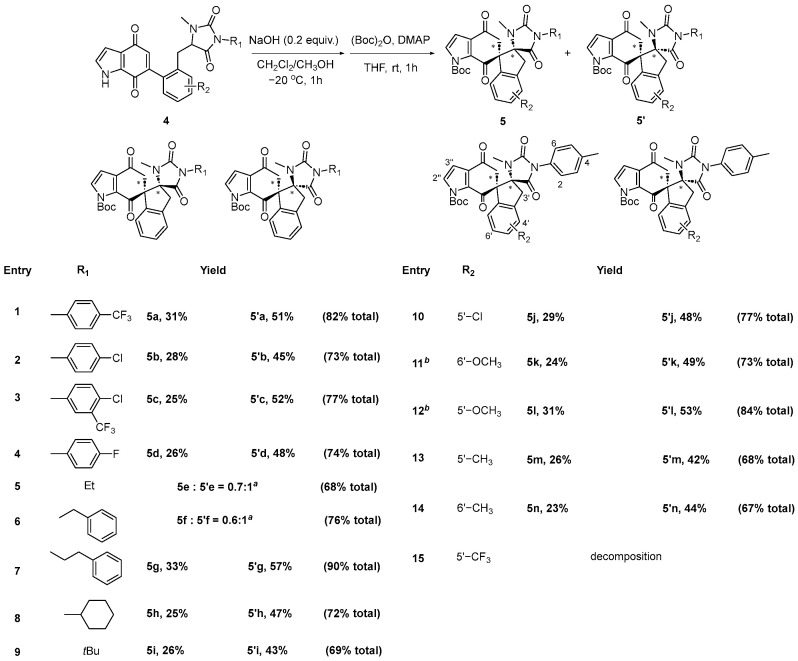
Investigation of substrate scope for the indolequinone derivatives. Treatment of compound **4** with a catalytic amount of NaOH (0.2 eq.) in a mixture of CH_2_Cl_2_ and CH_3_OH (5:1) at −20 °C. *^a^* Determined by ^1^H NMR. *^b^* Reaction was completed within 20 min.

In addition, we also explored the feasibility of this protocol using indolequinones **4** as substrates. It is well recognized that indole is a common and privileged motif in pharmaceutical molecules; hence, application of this methodology on indolequinones would not only broaden the substrate scope but would also merge indolequinone with the spirocyclic structure. As depicted in Figure 4, indolequinones **4** underwent smooth intramolecular Michael addition, resulting in the formation of the expected dispirocyclic compounds in good to excellent yields, which were easily separated by column chromatography after protection of the indole *N*- with Boc-, except compounds **5e**/**5’e** and **5f**/**5’f**. The R_1_ substituent was found to have no apparent influence on the transformation. The yields for products **5a**-**5n** ranged from 23% to 33%, while those for **5’a**-**5’n** ranged from 42% to 57%. We also explored the variation in R_2_ substituents and found that, similar to naphthoquinone substrates, electron-donating groups such as methoxy could accelerate the reaction (entries 11 and 12).

A scale-up synthesis of **2f** and **2′f** was then performed. This intramolecular Michael addition readily afforded the dispirocyclic products on a gram scale in good yields, which exemplified the potential of this protocol.

### 2.2. Cytotoxicity Test

Finally, the cytotoxicity of these dispirocyclic compounds was tested by MTT assay against five tumor cell lines, including human breast cancer cells (MCF-5), human liver cancer cells (HepG2), human colorectal cancer cells (HCT-16), human gastric cancer cells (HGC27), and human glioblastoma cells (U251). Some of the cytotoxicity results are listed in Table 2, and the other results (IC_50_ > 10 μmol/L) are attached in the Supplementary Materials. As can be seen in Table 2, in general, compounds **2** and **2′** exhibited potent cytotoxicity with an IC_50_ value of 10^−6^ mol/L. In contrast, compounds **5** and **5’** containing the indole motif normally did not show any inhibitory activity against tumor cells at a concentration of 10 μmol/L, except compounds **5’b** and **5’d**. The stronger potency of compound **2′** compared with **2** suggested that the configuration was essential for the cytotoxicity. Among all the tested compounds, most compounds were effective against MCF-7, HepG2, and HCT-116, and ineffective towards HGC27 and U251. In addition, it was apparent that R_1_ played a significant role in the cytotoxicity. When R_1_ was an aromatic ring, the compounds exerted more potent cytotoxicity than compounds with alkyl groups. The variation in R_2_ substituents did not affect the cytotoxicity dramatically.

## 3. Experimental Section

### 3.1. Materials

Reagents and solvents were purchased from commercial suppliers and used without further purification unless otherwise stated. ^1^H NMR and ^13^C NMR spectra were measured on Quantum-1 400 MHz (^1^H, 400 MHz; ^13^C, 100 MHz; ^19^F{H}, 376 MHz) or Quantum-1Plus 500 MHz (^1^H, 500 MHz; ^13^C, 125 MHz) spectrometers. Chemical shifts were given in ppm. Coupling constants were given in Hertz. High-resolution mass spectra were carried out on a Thermo Exactive Plus spectrometer. The ionization method was ESI, and the mass analyzer type was Orbitrap. Melting points were carried out on a microscopic melting point apparatus.

### 3.2. General Procedure for the Synthesis of Dispirocyclic Compounds

The general procedure for the preparation of (±)-(1′R*,4R*)-3-methyl-1-(*m*-tolyl)-1″H,3′H-dispiro [imidazolidine-4,2′-indene-1′,2″-naphthalene]-1″,2,4″,5(3″*H*)-tetraone **2a** and (±)-(1′S*, 4R*)-3-methyl-1-(*m*-tolyl)-1″*H*,3′*H*-dispiro[imidazolidine-4,2′-indene-1′,2″-naphthalene]-1″,2,4″,5(3″*H*)-tetraone **2′a** is as follows:

To a solution of 5-(2-(1,4-dioxo-1,4-dihydronaphthalen-2-yl)benzyl)-1-methyl-3-(*m*-tolyl)imidazolidine-2,4-dione **1a** (450 mg, 1.0 mmol) in CH_2_Cl_2_ (5 mL) and CH_3_OH (1 mL), NaOH (8 mg, 0.2 mmol) was added at −20 °C under argon. Then, the reaction was stirred at this temperature for 1 h. The mixture was quenched with 1N HCl solution (10 mL), extracted with CH_2_Cl_2_ (20 mL × 3), washed with brine (20 mL), dried over anhydrous Na_2_SO_4_, and concentrated under reduced pressure. The residue was purified by column chromatography (silica gel, PET/EtOAc = 5:1) to give compound **2a** (white solid, 158 mg, 35%) and **2′a** (white solid, 261 mg, 58%).

**2a**:

m.p. = 182–184 °C; ^1^H NMR (500 MHz, CDCl_3_) δ 8.08–8.03 (m, 1H), 7.98 (dd, *J* = 7.6, 1.2 Hz, 1H), 7.73 (td, *J* = 7.6, 1.2 Hz, 1H), 7.66 (td, *J* = 7.6, 1.2 Hz, 1H), 7.41 (d, *J* = 6.4 Hz, 3H), 7.17 (q, *J* = 6.8, 5.6 Hz, 2H), 7.06 (d, *J* = 7.6 Hz, 1H), 6.79–6.73 (m, 1H), 6.67 (s, 1H), 3.85 (d, *J* = 16.8 Hz, 1H), 3.76 (d, *J* = 15.6 Hz, 1H), 3.18 (d, *J* = 16.8 Hz, 1H), 3.15 (d, *J* = 15.6 Hz, 1H), 2.73 (s, 3H), 2.27 (s, 3H); ^13^C NMR (125 MHz, CDCl_3_) δ 193.7, 193.2, 170.6, 154.6, 140.2, 139.9, 138.8, 135.7, 135.3, 134.2, 134.0, 131.1, 129.7, 129.1, 128.8, 128.5 (2C), 126.1, 126.1, 124.5, 123.2, 122.6, 76.7, 68.6, 44.3, 39.9, 28.1, 21.4; HRMS (ESI) calcd for C_28_H_23_N_2_O_4_ [M + H]+ 451.1652, found 451.1655.

**2′a**:

m.p. = 181–183 °C; ^1^H NMR (500 MHz, CDCl_3_) δ 8.05 (dd, *J* = 7.5, 1.5 Hz, 1H), 8.00 (dd, *J* = 7.5, 1.5 Hz, 1H), 7.81 (td, *J* = 7.5, 1.5 Hz, 1H), 7.73 (td, *J* = 7.5, 1.5 Hz, 1H), 7.47–7.40 (m, 2H), 7.43–7.35 (m, 2H), 7.15 (t, *J* = 8.0 Hz, 1H), 7.06 (d, *J* = 8.0 Hz, 1H), 6.49 (d, *J* = 8.0 Hz, 1H), 6.43 (s, 1H), 3.91 (d, *J* = 16.5 Hz, 1H), 3.71 (d, *J* = 18.5 Hz, 1H), 3.23 (d, *J* = 16.5 Hz, 1H), 3.01 (d, *J* = 18.5 Hz, 1H), 2.54 (s, 3H), 2.27 (s, 3H); ^13^C NMR (125 MHz, CDCl_3_) δ 195.0, 190.9, 171.3, 154.2, 140.7, 138.7, 137.8, 136.3, 135.3, 135.1, 134.7, 130.7, 129.4, 129.1, 128.6, 128.5, 127.3, 127.2, 126.2, 125.8, 124.6, 122.7, 73.4, 67.6, 46.3, 39.9, 27.2, 21.4; HRMS (ESI) calcd for C_28_H_23_N_2_O_4_ [M + H]^+^ 451.1652, found 451.1649.

(±)-(1′R*,4R*)-3-methyl-1-(*p*-tolyl)-1″*H*,3′*H*-dispiro[imidazolidine-4,2′-indene-1′,2″-naphthalene]-1″,2,4″,5(3″*H*)-tetraone **2b** and (±)-(1′S*,4R*)-3-methyl-1-(*p*-tolyl)-1″*H*,3′*H*-dispiro[imidazolidine-4,2′-indene-1′,2″-naphthalene]-1″,2,4″,5(3″*H*)-tetraone **2′b**.

Compounds **2b** and **2′b** were prepared according to the general procedure. Purification by column chromatography (silica gel, PET/EtOAc = 5:1, *v*/*v*) generated compounds **2b** (white solid, 122 mg, 27%) and **2′b** (white solid, 284 mg, 63%).

**2b**:

m.p. = 185–187 °C; ^1^H NMR (400 MHz, CDCl_3_) δ 8.04 (d, *J* = 7.6 Hz, 1H), 7.95 (d, *J* = 7.6 Hz, 1H), 7.71 (t, *J* = 7.6 Hz, 1H), 7.64 (t, *J* = 7.6 Hz, 1H), 7.46–7.37 (m, 3H), 7.15 (d, *J* = 7.2 Hz, 1H), 7.09 (d, *J* = 8.0 Hz, 2H), 6.83 (d, *J* = 8.0 Hz, 2H), 3.86 (d, *J* = 16.4 Hz, 1H), 3.75 (d, *J* = 15.6 Hz, 1H), 3.17 (d, *J* = 16.4 Hz, 1H), 3.14 (d, *J* = 15.6 Hz, 1H), 2.72 (s, 3H), 2.30 (s, 3H); ^13^C NMR (100 MHz, CDCl_3_) δ 193.7, 193.2, 170.5, 154.7, 140.3, 139.9, 138.2, 135.6, 135.3, 134.2, 134.0, 129.6, 129.5 (2C), 128.6, 128.4, 128.4, 126.0, 125.3 (2C), 124.4, 123.2, 76.7, 68.5, 44.2, 39.8, 28.1, 21.2; HRMS (ESI) calcd for C_28_H_23_N_2_O_4_ [M + H]^+^ 451.1652, found 451.1651.

**2′b**:

m.p. = 186–188 °C; ^1^H NMR (400 MHz, CDCl_3_) δ 8.04 (dd, *J* = 7.6, 1.6 Hz, 1H), 7.98 (dd, *J* = 7.6, 1.6 Hz, 1H), 7.78 (td, *J* = 7.6, 1.6 Hz, 1H), 7.72 (td, *J* = 7.6, 1.6 Hz, 1H), 7.47–7.34 (m, 4H), 7.06 (d, *J* = 8.0 Hz, 2H), 6.57 (d, *J* = 8.0 Hz, 2H), 3.89 (d, *J* = 16.8 Hz, 1H), 3.70 (d, *J* = 18.8 Hz, 1H), 3.22 (d, *J* = 16.8 Hz, 1H), 3.01 (d, *J* = 18.8 Hz, 1H), 2.53 (s, 3H), 2.30 (s, 3H); ^13^C NMR (100 MHz, CDCl_3_) δ 195.1, 190.9, 171.4, 154.3, 140.7, 138.2, 137.9, 136.3, 135.3, 135.1, 134.7, 129.4, 129.3 (2C), 128.5, 128.3, 127.2, 127.1, 125.8, 125.3 (2C), 124.5, 73.4, 67.5, 46.3, 39.9, 27.2, 21.2; HRMS (ESI) calcd for C_28_H_23_N_2_O_4_ [M + H]^+^ 451.1652, found 451.1655.

(±)-(1′R*,4R*)-1-(4-chlorophenyl)-3-methyl-1″*H*,3′*H*-dispiro[imidazolidine-4,2′-indene-1′,2″-naphthalene]-1″,2,4″,5(3″*H*)-tetraone **2c** and (±)-(1′S*,4R*)-1-(4-chlorophenyl)-3-methyl-1″*H*,3′*H*-dispiro[imidazolidine-4,2′-indene-1′,2″-naphthalene]-1″,2,4″,5(3″*H*)-tetraone **2′c**.

Compounds **2c** and **2′c** were prepared according to the general procedure. Purification by column chromatography (silica gel, PET/EtOAc = 5:1, *v*/*v*) generated compounds **2c** (white solid, 137 mg, 29%) and **2′c** (white solid, 245 mg, 52%).

**2c**:

m.p. = 186–188 °C; ^1^H NMR (400 MHz, CDCl_3_) δ 8.04 (d, *J* = 7.6 Hz, 1H), 7.91 (d, *J* = 7.6 Hz, 1H), 7.72 (t, *J* = 7.6 Hz, 1H), 7.63 (t, *J* = 7.6 Hz, 1H), 7.48–7.36 (m, 3H), 7.30–7.24 (m, 2H), 7.14 (d, *J* = 7.2 Hz, 1H), 6.95 (d, *J* = 8.4 Hz, 2H), 3.85 (d, *J* = 16.4 Hz, 1H), 3.77 (d, *J* = 15.6 Hz, 1H), 3.18 (d, *J* = 16.4 Hz, 1H), 3.13 (d, *J* = 15.6 Hz, 1H), 2.73 (s, 3H); ^13^C NMR (125 MHz, CDCl_3_) δ 193.8, 193.1, 170.3, 154.1, 140.0, 139.7, 135.5, 135.4, 134.1, 134.1, 133.9, 129.8, 129.7, 129.1 (2C), 128.6, 128.4, 126.5 (2C), 125.9, 124.5, 123.2, 76.7, 68.7, 44.3, 39.6, 28.1; HRMS (ESI) calcd for C_27_H_20_ClN_2_O_4_ [M + H]^+^ 471.1106, found 471.1104.

**2′c**:

m.p. = 184–186 °C; ^1^H NMR (500 MHz, CDCl_3_) δ 8.03 (dd, *J* = 7.6, 1.2 Hz, 1H), 7.98 (dd, *J* = 7.6, 1.2 Hz, 1H), 7.78 (td, *J* = 7.6, 1.2 Hz, 1H), 7.71 (td, *J* = 7.6, 1.2 Hz, 1H), 7.50–7.33 (m, 4H), 7.26–7.20 (m, 2H), 6.87–6.61 (m, 2H), 3.90 (d, *J* = 16.8 Hz, 1H), 3.69 (d, *J* = 18.8 Hz, 1H), 3.23 (d, *J* = 16.8 Hz, 1H), 3.03 (d, *J* = 18.8 Hz, 1H), 2.55 (s, 3H); ^13^C NMR (125 MHz, CDCl_3_) δ 194.9, 190.9, 171.1, 153.7, 140.5, 137.7, 136.3, 135.3, 135.1, 134.8, 133.9, 129.5, 128.9 (2C), 128.6, 127.2, 127.1, 126.5 (2C), 125.8, 124.6, 73.5, 67.7, 46.3, 39.8, 27.2; HRMS (ESI) calcd for C_27_H_20_ClN_2_O_4_ [M + H]^+^ 471.1106, found 471.1106.

(±)-(1′R*,4R*)-1-(3-chlorophenyl)-3-methyl-1″*H*,3′*H*-dispiro[imidazolidine-4,2′-indene-1′,2″-naphthalene]-1″,2,4″,5(3″*H*)-tetraone **2d** and (±)-(1′S*,4R*)-1-(3-chlorophenyl)-3-methyl-1″*H*,3′*H*-dispiro[imidazolidine-4,2′-indene-1′,2″-naphthalene]-1″,2,4″,5(3″*H*)-tetraone **2′d**.

Compounds **2d** and **2′d** were prepared according to the general procedure. Purification by column chromatography (silica gel, PET/EtOAc = 5:1, *v*/*v*) generated compounds **2d** (white solid, 146 mg, 31%) and **2′d** (white solid, 273 mg, 58%).

**2d**:

m.p. = 180–182 °C; ^1^H NMR (500 MHz, CDCl_3_) δ 8.06 (dd, *J* = 7.5, 1.5 Hz, 1H), 7.95 (dd, *J* = 7.5, 1.5 Hz, 1H), 7.75 (td, *J* = 7.5, 1.5 Hz, 1H), 7.68 (td, *J* = 7.5, 1.5 Hz, 1H), 7.47–7.39 (m, 3H), 7.24–7.21 (m, 2H), 7.17–7.12 (m, 1H), 6.96 (ddd, *J* = 6.0, 3.5, 2.0 Hz, 1H), 6.85–6.81 (m, 1H), 3.84 (d, *J* = 16.5 Hz, 1H), 3.77 (d, *J* = 15.5 Hz, 1H), 3.18 (d, *J* = 16.5 Hz, 1H), 3.13 (d, *J* = 15.5 Hz, 1H), 2.74 (s, 3H); ^13^C NMR (125 MHz, CDCl_3_) δ 193.6, 193.1, 170.3, 153.9, 140.0, 139.7, 135.6, 135.5, 134.4, 134.3, 134.1, 132.3, 129.9, 129.7, 128.6, 128.5, 128.3, 126.1, 125.5, 124.5, 123.5, 123.3, 76.6, 68.9, 44.4, 39.7, 28.2; HRMS (ESI) calcd for C_27_H_20_ClN_2_O_4_ [M + H]^+^ 471.1106, found 471.1110.

**2′d**:

m.p. = 181–183 °C; ^1^H NMR (400 MHz, CDCl_3_) δ 8.08–8.02 (m, 1H), 8.01–7.96 (m, 1H), 7.82 (td, *J* = 7.6, 1.2 Hz, 1H), 7.74 (td, *J* = 7.6, 1.2 Hz, 1H), 7.47–7.37 (m, 4H), 7.25–7.17 (m, 2H), 6.77 (dt, *J* = 6.8, 2.0 Hz, 1H), 6.65–6.60 (m, 1H), 3.92 (d, *J* = 16.8 Hz, 1H), 3.69 (d, *J* = 18.8 Hz, 1H), 3.23 (d, *J* = 16.8 Hz, 1H), 3.02 (d, *J* = 18.8 Hz, 1H), 2.55 (s, 3H); ^13^C NMR (100 MHz, CDCl_3_) δ 194.9, 190.8, 171.0, 153.5, 140.5, 137.7, 136.3, 135.3, 135.2, 134.9, 134.3, 131.9, 129.7, 129.5, 128.6, 128.4, 127.2 (2C), 125.8, 125.6, 124.6, 123.6, 73.5, 67.7, 46.3, 39.8, 27.2; HRMS (ESI) calcd for C_27_H_20_ClN_2_O_4_ [M + H]^+^ 471.1106, found 471.1102.

(±)-(1′R*,4R*)-1-(4-fluorophenyl)-3-methyl-1″*H*,3′*H*-dispiro[imidazolidine-4,2′-indene-1′,2″-naphthalene]-1″,2,4″,5(3″*H*)-tetraone **2e** and (±)-(1′S*,4R*)-1-(4-fluorophenyl)-3-methyl-1″*H*,3′*H*-dispiro[imidazolidine-4,2′-indene-1′,2″-naphthalene]-1″,2,4″,5(3″*H*)-tetraone **2′e**.

Compounds **2e** and **2′e** were prepared according to the general procedure. Purification by column chromatography (silica gel, PET/EtOAc = 5:1, *v*/*v*) generated compounds **2e** (white solid, 150 mg, 33%) and **2′e** (white solid, 250 mg, 55%).

**2e**:

m.p. = 186–188 °C; ^1^H NMR (400 MHz, CDCl_3_) δ 8.04 (dd, *J* = 7.6, 1.2 Hz, 1H), 7.92 (dd, *J* = 7.6, 1.2 Hz, 1H), 7.72 (td, *J* = 7.6, 1.2 Hz, 1H), 7.63 (td, *J* = 7.6, 1.2 Hz, 1H), 7.48–7.37 (m, 3H), 7.18–7.11 (m, 1H), 7.01–6.96 (m, 4H), 3.86 (d, *J* = 16.4 Hz, 1H), 3.77 (d, *J* = 15.6 Hz, 1H), 3.18 (d, *J* = 16.4 Hz, 1H), 3.14 (d, *J* = 15.6 Hz, 1H), 2.73 (s, 3H); ^13^C NMR (125 MHz, CDCl_3_) δ 193.8, 193.1, 170.4, 162.9, 161.0, 154.3, 140.1, 139.8, 135.5, 135.4, 134.2, 134.1, 129.7, 128.6, 128.4, 127.3, 127.2, 127.2, 127.1, 125.9, 124.5, 123.2, 116.0, 115.8, 76.7, 68.6, 44.3, 39.7, 28.1; ^19^F{H} NMR (376 MHz, CDCl_3_) δ −112.8; HRMS (ESI) calcd for C_27_H_20_FN_2_O_4_ [M + H]^+^ 455.1402, found 455.1401.

**2′e**:

m.p. = 186–188 °C; ^1^H NMR (400 MHz, CDCl_3_) δ 8.04 (dd, *J* = 7.6, 1.2 Hz, 1H), 7.99 (dd, *J* = 7.6, 1.2 Hz, 1H), 7.79 (td, *J* = 7.6, 1.2 Hz, 1H), 7.72 (td, *J* = 7.6, 1.2 Hz, 1H), 7.47–7.35 (m, 4H), 6.99–6.91 (m, 2H), 6.77–6.70 (m, 2H), 3.90 (d, *J* = 16.8 Hz, 1H), 3.70 (d, *J* = 18.4 Hz, 1H), 3.23 (d, *J* = 16.8 Hz, 1H), 3.03 (d, *J* = 18.4 Hz, 1H), 2.55 (s, 3H); ^13^C NMR (125 MHz, CDCl_3_) δ 194.9, 190.9, 171.3, 162.9, 160.9, 153.9, 140.5, 137.8, 136.3, 135.3, 135.1, 134.8, 129.5, 128.6, 127.3, 127.2, 127.2, 127.2, 126.9, 126.8, 125.8, 124.6, 115.9, 115.7, 73.5, 67.6, 46.3, 39.8, 27.2; ^19^F{H} NMR (376 MHz, CDCl_3_) δ −112.6; HRMS (ESI) calcd for C_27_H_20_FN_2_O_4_ [M + H]^+^ 455.1402, found 455.1400.

(±)-(1′R*,4R*)-3-methyl-1-(4-(trifluoromethyl)phenyl)-1″*H*,3′*H*-dispiro[imidazolidine-4,2′-indene-1′,2″-naphthalene]-1″,2,4″,5(3″*H*)-tetraone **2f** and (±)-(1′S*,4R*)-3-methyl-1-(4-(trifluoromethyl)phenyl)-1″*H*,3′*H*-dispiro[imidazolidine-4,2′-indene-1′,2″-naphthalene]-1″,2,4″,5(3″*H*)-tetraone **2′f**.

Compounds **2f** and **2′f** were prepared according to the general procedure. Purification by column chromatography (silica gel, PET/EtOAc = 5:1, *v*/*v*) generated compounds **2f** (white solid, 152 mg, 30%) and **2′f** (white solid, 328 mg, 65%).

**2f**:

m.p. = 176–178 °C; ^1^H NMR (400 MHz, CDCl_3_) δ 8.05 (d, *J* = 7.6 Hz, 1H), 7.89 (d, *J* = 7.6 Hz, 1H), 7.73 (t, *J* = 7.6 Hz, 1H), 7.63 (t, *J* = 7.6 Hz, 1H), 7.57 (d, *J* = 8.4 Hz, 2H), 7.47–7.39 (m, 3H), 7.16 (t, *J* = 7.6 Hz, 3H), 3.87 (d, *J* = 16.4 Hz, 1H), 3.78 (d, *J* = 15.6 Hz, 1H), 3.20 (d, *J* = 16.4 Hz, 1H), 3.14 (d, *J* = 15.6 Hz, 1H), 2.75 (s, 3H); ^13^C NMR (125 MHz, CDCl_3_) δ 193.7, 193.0, 170.3, 153.8, 139.9, 139.6, 135.5, 135.5, 134.4, 134.2 (2C), 129.9 (q, *J* = 35.0 Hz, 1C), 129.8, 128.6, 128.5, 126.0 (q, *J* = 4.0 Hz, 2C), 126.0, 125.3 (2C), 124.5, 123.8 (q, *J* = 272.5 Hz, 1C),123.2, 76.7, 68.8, 44.4, 39.6, 28.2; ^19^F{H} NMR (376 MHz, CDCl_3_) δ −62.6; HRMS (ESI) calcd for C_28_H_20_F_3_N_2_O_4_ [M + H]^+^ 505.1370, found 505.1366.

**2′f**:

m.p. = 179–181 °C; ^1^H NMR (400 MHz, CDCl_3_) δ 8.05–7.96 (m, 2H), 7.78 (td, *J* = 7.6, 1.2 Hz, 1H), 7.69 (td, *J* = 7.6, 1.2 Hz, 1H), 7.54 (d, *J* = 8.0 Hz, 2H), 7.48–7.35 (m, 4H), 7.00 (d, *J* = 8.0 Hz, 2H), 3.92 (d, *J* = 16.8 Hz, 1H), 3.70 (d, *J* = 18.4 Hz, 1H), 3.25 (d, *J* = 16.8 Hz, 1H), 3.05 (d, *J* = 18.4 Hz, 1H), 2.56 (s, 3H); ^13^C NMR (125 MHz, CDCl_3_) δ 194.8, 190.9, 171.1, 153.4, 140.4, 137.6, 136.2, 135.2, 135.2, 134.8, 134.1, 129.9 (q, *J* = 32.5 Hz, 1C), 129.5, 128.7, 127.2, 127.1, 125.9 (q, *J* = 4.0 Hz, 2C), 125.8, 125.2 (2C), 124.6, 123.7 (q, *J* = 272.5 Hz, 1C) 73.5, 67.7, 46.3, 39.8, 27.3; ^19^F{H} NMR (376 MHz, CDCl_3_) δ −62.6; HRMS (ESI) calcd for C_28_H_20_F_3_N_2_O_4_ [M + H]^+^ 505.1370, found 505.1375.

(±)-(1′R*,4R*)-1-benzyl-3-methyl-1″*H*,3′*H*-dispiro[imidazolidine-4,2′-indene-1′,2″-naphthalene]-1″,2,4″,5(3″*H*)-tetraone **2g** and (±)-(1′S*,4R*)-1-benzyl-3-methyl-1″*H*,3′*H*-dispiro[imidazolidine-4,2′-indene-1′,2″-naphthalene]-1″,2,4″,5(3″*H*)-tetraone **2′g**.

Compound **2g** and **2′g** was prepared according to the general procedure. Purification by column chromatography (silica gel, PET/EtOAc = 5:1, *v*/*v*) generated compounds **2g** (white solid, 117 mg, 26%) and **2′g** (white solid, 248 mg, 55%).

**2g**:

m.p. = 185–187 °C; ^1^H NMR (400 MHz, CDCl_3_) δ 8.02 (dd, *J* = 7.6, 1.2 Hz, 1H), 7.69 (td, *J* = 7.6, 1.2 Hz, 1H), 7.60 (dd, *J* = 7.6, 1.2 Hz, 1H), 7.54 (td, *J* = 7.6, 1.2 Hz, 1H), 7.44–7.32 (m, 3H), 7.29–7.21 (m, 3H), 7.16–7.08 (m, 3H), 4.26 (d, *J* = 14.4 Hz, 1H), 4.21 (d, *J* = 14.4 Hz, 1H), 3.74 (d, *J* = 16.4 Hz, 1H), 3.61 (d, *J* = 16.0 Hz, 1H), 3.03 (d, *J* = 16.4 Hz, 1H), 3.03 (d, *J* = 16.0 Hz, 1H), 2.63 (s, 3H); ^13^C NMR (125 MHz, CDCl_3_) δ 193.2, 193.2, 171.3, 155.6, 140.4, 139.9, 135.3, 135.1, 134.9, 134.0, 133.9, 129.5, 129.1 (2C), 128.7 (2C), 128.4, 128.1, 127.9, 125.9, 124.4, 123.1, 77.4, 77.2, 76.9, 76.9, 67.8, 43.9, 42.8, 39.9, 28.0; HRMS (ESI) calcd for C_28_H_23_N_2_O_4_ [M + H]^+^ 451.1652, found 451.1649.

**2′g**:

m.p. = 186–188 °C; ^1^H NMR (400 MHz, CDCl_3_) δ 7.96 (dd, *J* = 7.6, 1.2 Hz, 1H), 7.84 (dd, *J* = 7.6, 1.2 Hz, 1H), 7.66 (td, *J* = 7.6, 1.2 Hz, 1H), 7.57 (td, *J* = 7.6, 1.2 Hz, 1H), 7.42–7.34 (m, 3H), 7.33–7.28 (m, 1H), 7.25–7.20 (m, 3H), 7.18–7.13 (m, 2H), 4.04 (d, *J* = 14.0 Hz, 1H), 3.93 (d, *J* = 14.0 Hz, 1H), 3.75 (d, *J* = 16.8 Hz, 1H), 3.64 (d, *J* = 18.4 Hz, 1H), 3.05 (d, *J* = 16.8 Hz, 1H), 2.95 (d, *J* = 18.4 Hz, 1H), 2.44 (s, 3H); ^13^C NMR (100 MHz, CDCl_3_) δ 195.0, 191.1, 172.2, 155.0, 140.6, 137.9, 136.0, 135.3, 135.1, 134.8, 134.6, 129.3, 129.1 (2C), 128.7 (2C), 128.4, 128.0, 126.7, 126.6, 125.8, 124.5, 77.5, 77.2, 76.8, 73.7, 67.2, 46.1, 42.6, 39.5, 26.9; HRMS (ESI) calcd for C_28_H_23_N_2_O_4_ [M + H]^+^ 451.1652, found 451.1653.

(±)-(1′R*,4R*)-3-methyl-1-phenethyl-1″*H*,3′*H*-dispiro[imidazolidine-4,2′-indene-1′,2″-naphthalene]-1″,2,4″,5(3″*H*)-tetraone **2h** and (±)-(1′S*,4R*)-3-methyl-1-phenethyl-1″*H*,3′*H*-dispiro[imidazolidine-4,2′-indene-1′,2″-naphthalene]-1″,2,4″,5(3″*H*)-tetraone **2′h**.

Compounds **2h** and **2′h** were prepared according to the general procedure. Purification by column chromatography (silica gel, PET/EtOAc = 5:1, *v*/*v*) generated compounds **2h** (white solid, 167 mg, 36%) and **2′h** (white solid, 237 mg, 51%).

**2h**:

m.p. = 179–181 °C; ^1^H NMR (400 MHz, CDCl_3_) δ 8.21–8.03 (m, 1H), 8.00–7.93 (m, 1H), 7.79 (td, *J* = 7.6, 1.2 Hz, 1H), 7.70 (td, *J* = 7.6, 1.2 Hz, 1H), 7.43–7.33 (m, 3H), 7.26–7.15 (m, 3H), 7.14–7.03 (m, 3H), 3.72 (d, *J* = 16.8 Hz, 1H), 3.64 (d, *J* = 16.0 Hz, 1H), 3.41 (ddd, *J* = 13.2, 10.8, 5.6 Hz, 1H), 3.31 (ddd, *J* = 13.2, 10.8, 5.6 Hz, 1H), 3.01 (d, *J* = 16.0 Hz, 1H), 2.98 (d, *J* = 16.8 Hz, 1H), 2.68 (ddd, *J* = 13.2, 10.8, 5.6 Hz, 1H), 2.62 (s, 3H), 2.40 (ddd, *J* = 13.2, 10.8, 5.6 Hz, 1H); ^13^C NMR (125 MHz, CDCl_3_) δ 193.7, 193.1, 171.3, 155.4, 140.4, 140.0, 137.7, 135.6, 135.3, 134.4, 134.1, 129.6, 128.9 (2C), 128.6 (2C), 128.4 (2C), 126.7, 125.9, 124.5, 123.1, 76.8, 67.8, 44.0, 40.2, 39.7, 33.2, 27.8; HRMS (ESI) calcd for C_29_H_25_N_2_O_4_ [M + H]^+^ 465.1809, found 465.1806.

**2′h**:

m.p. = 181–183 °C; ^1^H NMR (400 MHz, CDCl_3_) δ 8.17–8.08 (m, 1H), 7.93–7.85 (m, 1H), 7.78–7.69 (m, 2H), 7.46–7.30 (m, 4H), 7.29–7.17 (m, 3H), 7.11–7.04 (m, 2H), 3.72 (d, *J* = 16.8 Hz, 1H), 3.64 (d, *J* = 18.4 Hz, 1H), 3.24–3.05 (m, 2H), 2.99 (d, *J* = 16.8 Hz, 1H), 2.96 (d, *J* = 18.4 Hz, 1H), 2.44 (s, 3H), 2.39–2.20 (m, 2H); ^13^C NMR (100 MHz, CDCl_3_) δ 195.0, 191.0, 172.2, 154.9, 140.7, 137.9, 137.5, 136.3, 135.4, 135.0, 134.6, 129.3, 128.9 (2C), 128.6 (2C), 128.5, 127.1, 126.8 (2C), 125.7, 124.5, 73.5, 67.2, 46.2, 39.9, 39.4, 33.6, 26.8; HRMS (ESI) calcd for C_29_H_25_N_2_O_4_ [M + H]^+^ 465.1809, found 465.1807.

(±)-(1′R*,4R*)-1-cyclohexyl-3-methyl-1″*H*,3′*H*-dispiro[imidazolidine-4,2′-indene-1′,2″-naphthalene]-1″,2,4″,5(3″*H*)-tetraone **2i** and (±)-(1′S*,4R*)-1-cyclohexyl-3-methyl-1″*H*,3′*H*-dispiro[imidazolidine-4,2′-indene-1′,2″-naphthalene]-1″,2,4″,5(3″*H*)-tetraone **2′i**.

Compounds **2i** and **2′i** were prepared according to the general procedure. Purification by column chromatography (silica gel, PET/EtOAc = 5:1, *v*/*v*) generated compounds **2i** (white solid, 142 mg, 32%) and **2i** (white solid, 213 mg, 48%).

**2i**:

m.p. = 171–173 °C; ^1^H NMR (400 MHz, CDCl_3_) δ 8.09 (dd, *J* = 7.6, 1.2 Hz, 1H), 7.95 (dd, *J* = 7.6, 1.2 Hz, 1H), 7.78 (td, *J* = 7.6, 1.6 Hz, 1H), 7.70 (td, *J* = 7.6, 1.6 Hz, 1H), 7.43–7.33 (m, 3H), 7.11 (dd, *J* = 6.4, 2.8 Hz, 1H), 3.73 (d, *J* = 16.4 Hz, 1H), 3.67 (d, *J* = 15.6 Hz, 1H), 3.56 (tt, *J* = 12.4, 4.0 Hz, 1H), 3.07 (d, *J* = 15.6 Hz, 1H), 3.04 (d, *J* = 16.4 Hz, 1H), 2.59 (s, 3H), 1.85 (qd, *J* = 12.4, 3.6 Hz, 1H), 1.74–1.66 (m, 1H), 1.65–1.58 (m, 1H), 1.56–1.48 (m, 1H), 1.44–1.29 (m, 2H), 1.30–1.23 (m, 1H), 1.22–0.94 (m, 3H); ^13^C NMR (125 MHz, CDCl_3_) δ 193.8, 193.3, 171.2, 155.5, 140.6, 140.1, 135.8, 135.2, 134.4, 133.8, 129.5, 128.4, 128.3, 126.1, 124.4, 123.0, 76.4, 67.5, 52.1, 43.9, 40.1, 28.6, 28.3, 27.9, 25.7, 25.6, 24.9; HRMS (ESI) calcd for C_27_H_27_N_2_O_4_ [M + H]^+^ 443.1965, found 443.1968.

**2′i**:

m.p. = 174–176 °C; ^1^H NMR (500 MHz, CDCl_3_) δ 8.13 (dd, *J* = 7.0, 2.0 Hz, 1H), 7.92 (dd, *J* = 7.0, 2.0 Hz, 1H), 7.82–7.65 (m, 2H), 7.48–7.31 (m, 4H), 3.77 (d, *J* = 16.5 Hz, 1H), 3.68 (d, *J* = 18.5 Hz, 1H), 3.53–3.45 (m, 1H), 3.06 (d, *J* = 16.5 Hz, 1H), 2.96 (d, *J* = 18.5 Hz, 1H), 2.44 (s, 3H), 1.86–1.41 (m, 5H), 1.15–1.01 (m, 3H), 0.86 (t, *J* = 13.3 Hz, 2H); ^13^C NMR (125 MHz, CDCl_3_) δ 195.1, 191.2, 172.2, 155.3, 141.2, 138.0, 136.3, 135.4, 135.0, 134.5, 129.2, 128.4, 127.3, 127.1, 125.6, 124.5, 72.9, 67.0, 51.8, 46.4, 40.0, 28.9, 28.5, 27.0, 25.7, 25.6, 24.8; HRMS (ESI) calcd for C_27_H_27_N_2_O_4_ [M + H]^+^ 443.1965, found 443.1961.

(±)-(1′R*,4R*)-1-isopropyl-3-methyl-1″*H*,3′*H*-dispiro[imidazolidine-4,2′-indene-1′,2″-naphthalene]-1″,2,4″,5(3″*H*)-tetraone **2j** and (±)-(1′S*,4R*)-1-isopropyl-3-methyl-1″*H*,3′*H*-dispiro[imidazolidine-4,2′-indene-1′,2″-naphthalene]-1″,2,4″,5(3″*H*)-tetraone **2′j**.

Compounds **2j** and **2′j** were prepared according to the general procedure. Purification by column chromatography (silica gel, PET/EtOAc = 5:1, *v*/*v*) generated compounds **2j** (white solid, 149 mg, 37%) and **2′j** (white solid, 230 mg, 57%).

**2j**:

m.p. = 178–180 °C; ^1^H NMR (400 MHz, CDCl_3_) δ 8.09 (dd, *J* = 7.6, 1.2 Hz, 1H), 7.96 (dd, *J* = 7.6, 1.6 Hz, 1H), 7.77 (td, *J* = 7.6, 1.6 Hz, 1H), 7.70 (td, *J* = 7.6, 1.2 Hz, 1H), 7.43–7.33 (m, 3H), 7.15–7.08 (m, 1H), 3.96 (p, *J* = 6.8 Hz, 1H), 3.73 (d, *J* = 16.4 Hz, 1H), 3.68 (d, *J* = 16.0 Hz, 1H), 3.05 (d, *J* = 16.0 Hz, 1H), 3.01 (d, *J* = 16.4 Hz, 1H), 2.59 (s, 3H), 1.16 (d, *J* = 6.8 Hz, 3H), 0.94 (d, *J* = 6.8 Hz, 3H); ^13^C NMR (125 MHz, CDCl_3_) δ 193.8, 193.3, 171.1, 155.4, 140.5, 140.1, 135.8, 135.2, 134.4, 133.9, 129.5, 128.4, 128.3, 126.0, 124.4, 123.0, 76.4, 67.6, 44.4, 43.9, 40.0, 27.8, 19.0, 18.8; HRMS (ESI) calcd for C_24_H_23_N_2_O_4_ [M + H]^+^ 403.1652, found 403.1648.

**2′j**:

m.p. = 178–180 °C; ^1^H NMR (400 MHz, CDCl_3_) δ 8.17–8.08 (m, 1H), 7.97–7.89 (m, 1H), 7.82–7.68 (m, 2H), 7.45–7.30 (m, 4H), 3.98–3.84 (m, 1H), 3.77 (d, *J* = 16.4 Hz, 1H), 3.68 (d, *J* = 18.4 Hz, 1H), 3.07 (d, *J* = 16.4 Hz, 1H), 2.97 (d, *J* = 18.4 Hz, 1H), 2.44 (s, 3H), 0.90 (d, *J* = 5.2 Hz, 3H), 0.88 (d, *J* = 5.2 Hz, 3H); ^13^C NMR (125 MHz, CDCl_3_) δ 195.1, 191.1, 172.1, 155.1, 141.2, 137.9, 136.2, 135.4, 135.0, 134.7, 129.2, 128.4, 127.3, 127.1, 125.6, 124.5, 72.9, 67.0, 46.4, 44.0, 40.0, 26.9, 19.3, 19.0; HRMS (ESI) calcd for C_24_H_23_N_2_O_4_ [M + H]^+^ 403.1652, found 403.1653.

(±)-(1′R*,4R*)-1-(*tert*-butyl)-3-methyl-1″*H*,3′*H*-dispiro[imidazolidine-4,2′-indene-1′,2″-naphthalene]-1″,2,4″,5(3″*H*)-tetraone **2k** and (±)-(1′S*,4R*)-1-(*tert*-butyl)-3-methyl-1″*H*,3′*H*-dispiro[imidazolidine-4,2′-indene-1′,2″-naphthalene]-1″,2,4″,5(3″*H*)-tetraone **2′k**.

Compounds **2k** and **2′k** were prepared according to the general procedure. Purification by column chromatography (silica gel, PET/EtOAc = 5:1, *v*/*v*) generated compounds **2k** (white solid, 142 mg, 34%) and **2′k** (white solid, 254 mg, 61%).

**2k**:

m.p. = 177–179 °C; ^1^H NMR (500 MHz, CDCl_3_) δ 8.09 (dd, *J* = 7.5, 1.5 Hz, 1H), 8.02 (dd, *J* = 7.5, 1.5 Hz, 1H), 7.78 (td, *J* = 7.5, 1.5 Hz, 1H), 7.73 (td, *J* = 7.5, 1.5 Hz, 1H), 7.40–7.35 (m, 3H), 7.11 (dd, *J* = 7.0, 2.0 Hz, 1H), 3.72 (d, *J* = 16.5 Hz, 1H), 3.67 (d, *J* = 16.0 Hz, 1H), 3.03 (d, *J* = 16.0 Hz, 1H), 2.99 (d, *J* = 16.5 Hz, 1H), 2.58 (s, 3H), 1.19 (s, 9H); ^13^C NMR (100 MHz, CDCl_3_) δ 193.7, 193.5, 171.8, 156.2, 140.5, 140.3, 135.9, 135.1, 134.5, 133.9, 129.5, 128.5, 128.3, 126.1, 124.3, 123.1, 77.5, 77.2, 76.8, 76.4, 67.9, 58.4, 44.0, 40.1, 27.9 (3C), 27.6; HRMS (ESI) calcd for C_25_H_25_N_2_O_4_ [M + H]^+^ 417.1809, found 417.1807.

**2′k**:

m.p. = 176–177 °C; ^1^H NMR (500 MHz, CDCl_3_) δ 8.20 (dd, *J* = 7.5, 1.5 Hz, 1H), 8.00 (dd, *J* = 7.5, 1.5 Hz, 1H), 7.85 (td, *J* = 7.5, 1.5 Hz, 1H), 7.80 (td, *J* = 7.5, 1.5 Hz, 1H), 7.47–7.34 (m, 4H), 3.79 (d, *J* = 16.5 Hz, 1H), 3.69 (d, *J* = 18.5 Hz, 1H), 3.09 (d, *J* = 16.5 Hz, 1H), 3.01 (d, *J* = 18.5 Hz, 1H), 2.45 (s, 3H), 1.18 (s, 9H); ^13^C NMR (100 MHz, CDCl_3_) δ 195.2, 191.3, 172.8, 155.9, 141.2, 138.2, 136.4, 135.5, 135.0, 134.6, 129.2, 128.3, 127.4, 127.1, 125.6, 124.4, 77.5, 77.2, 76.8, 72.8, 67.2, 58.2, 46.5, 40.1, 28.3 (3C), 26.8; HRMS (ESI) calcd for C_25_H_25_N_2_O_4_ [M + H]^+^ 417.1809, found 417.1807.

1-ethyl-3-methyl-1″*H*,3′*H*-dispiro[imidazolidine-4,2′-indene-1′,2″-naphthalene]-1″,2,4″,5(3″*H*)-tetraone **2l** and **2′l**.

Compounds **2l** and **2′l** were prepared according to the general procedure. Purification by column chromatography (silica gel, PET/EtOAc = 5:1, *v*/*v*) generated compounds **2l** and **2′l** (white solid, 342 mg, 88%). m.p. = 181–183 °C; ^1^H NMR (400 MHz, CDCl_3_) δ 8.15–8.10 (m, 1H), 8.09–8.06 (m, 0.4H), 7.96–7.93 (m, 0.4H), 7.91–7.87 (m, 1H), 7.80–7.67 (m, 3H), 7.45–7.30 (m, 5H), 7.16–7.05 (m, 0.4H), 3.83–3.64 (m, 1.8H), 3.66 (d, *J* = 18.8 Hz, 1H), 3.26–3.11 (m, 0.8H), 3.08–2.91 (m, 4.8H), 2.61 (s, 1.2H), 2.44 (s, 3H), 0.81 (t, *J* = 7.2 Hz, 1.2H), 0.60 (t, *J* = 7.2 Hz, 3H); ^13^C NMR (100 MHz, CDCl_3_) δ 195.0, 193.6, 193.2, 191.0, 172.2, 171.3, 155.5, 155.1, 140.8, 140.4, 140.0, 137.9, 136.3, 135.7, 135.3, 135.2, 134.9, 134.6, 134.3, 134.0, 129.5, 129.2, 128.4, 128.4, 128.3, 127.1, 126.9, 125.9, 125.7, 124.5, 124.4, 123.1, 76.8, 73.5, 67.8, 67.2, 46.2, 44.0, 39.7, 39.5, 34.2, 33.9, 27.8, 26.8, 12.8, 12.4; HRMS (ESI) calcd for C_23_H_21_N_2_O_4_ [M + H]^+^ 389.1496, found 389.1496.

(±)-(1′R*,4R*)-6’-methoxy-3-methyl-1-(*p-*tolyl)-1″*H*,3′*H*-dispiro[imidazolidine-4,2′-indene-1′,2″-naphthalene]-1″,2,4″,5(3″*H*)-tetraone **2m** and (±)-(1′S*,4R*)-6’-methoxy-3-methyl-1-(*p-*tolyl)-1″*H*,3′*H*-dispiro[imidazolidine-4,2′-indene-1′,2″-naphthalene]-1″,2,4″,5(3″*H*)-tetraone **2′m**.

Compounds **2m** and **2′m** were prepared according to the general procedure; the reaction was completed within 20 min. Purification by column chromatography (silica gel, PET/EtOAc = 5:1, *v*/*v*) generated compounds **2m** (white solid, 149 mg, 31%) and **2′m** (white solid, 279 mg, 58%).

**2m**:

m.p. = 185–187 °C; ^1^H NMR (400 MHz, CDCl_3_) δ 8.04 (d, *J* = 7.6 Hz, 1H), 7.95 (d, *J* = 7.6 Hz, 1H), 7.70 (td, *J* = 7.6, 1.6 Hz, 1H), 7.67–7.59 (m, 1H), 7.30 (d, *J* = 8.4 Hz, 1H), 7.09 (d, *J* = 8.0 Hz, 2H), 6.96 (dd, *J* = 8.4, 2.4 Hz, 1H), 6.66 (d, *J* = 2.4 Hz, 1H), 3.82 (s, 3H), 3.77 (d, *J* = 16.4 Hz, 1H), 3.70 (d, *J* = 15.6 Hz, 1H), 3.14 (d, *J* = 15.6 Hz, 1H), 3.10 (d, *J* = 16.4 Hz, 1H), 2.74 (s, 3H), 2.29 (s, 3H); ^13^C NMR (125 MHz, CDCl_3_) δ 193.7, 193.1, 170.4, 160.1, 154.7, 141.6, 138.2, 135.6, 135.3, 134.2, 134.0, 131.7, 129.6 (2C), 128.6, 128.4, 126.0, 125.3 (2C), 125.1, 115.2, 108.9, 77.1, 68.5, 55.6, 44.2, 39.1, 28.1, 21.2; HRMS (ESI) calcd for C_29_H_25_N_2_O_5_ [M + H]^+^ 481.1758, found 481.1754.

**2′m**:

m.p. = 184–186 °C; ^1^H NMR (400 MHz, CDCl_3_) δ 8.04 (dd, *J* = 7.6, 1.2 Hz, 1H), 7.99 (dd, *J* = 7.6, 1.2 Hz, 1H), 7.79 (td, *J* = 7.6, 1.2 Hz, 1H), 7.72 (td, *J* = 7.6, 1.2 Hz, 1H), 7.26 (d, *J* = 8.4 Hz, 1H), 7.09–7.02 (m, 2H), 6.99–6.90 (m, 2H), 6.60–6.53 (m, 2H), 3.86 (s, 3H), 3.82 (d, *J* = 16.4 Hz, 1H), 3.71 (d, *J* = 18.8 Hz, 1H), 3.15 (d, *J* = 16.4 Hz, 1H), 3.02 (d, *J* = 18.8 Hz, 1H), 2.57 (s, 3H), 2.30 (s, 3H); ^13^C NMR (125 MHz, CDCl_3_) δ 195.1, 190.9, 171.3, 160.0, 154.3, 142.1, 138.2, 136.3, 135.3, 135.1, 134.7, 129.5, 129.4 (2C), 128.2, 127.2, 127.1, 125.4 (2C), 125.2, 115.9, 110.6, 73.8, 67.6, 55.7, 46.2, 39.2, 27.2, 21.2; HRMS (ESI) calcd for C_29_H_25_N_2_O_5_ [M + H]^+^ 481.1758, found 481.1755.

(±)-(1′R*,4R*)-5’-methoxy-3-methyl-1-(*p-*tolyl)-1″*H*,3′*H*-dispiro[imidazolidine-4,2′-indene-1′,2″-naphthalene]-1″,2,4″,5(3″*H*)-tetraone **2n** and (±)-(1′S*,4R*)-5’-methoxy-3-methyl-1-(*p-*tolyl)-1″*H*,3′*H*-dispiro[imidazolidine-4,2′-indene-1′,2″-naphthalene]-1″,2,4″,5(3″*H*)-tetraone **2′n**.

Compounds **2n** and **2′n** were prepared according to the general procedure; the reaction was completed within 20 min. Purification by column chromatography (silica gel, PET/EtOAc = 5:1, *v*/*v*) generated compounds **2n** (white solid, 154 mg, 32%) and **2′n** (white solid, 293 mg, 61%).

**2n**:

m.p. = 188–190 °C; ^1^H NMR (400 MHz, CDCl_3_) δ 8.04 (ddd, *J* = 7.6, 1.6, 0.8 Hz, 1H), 7.95 (ddd, *J* = 7.6, 1.6, 0.8 Hz, 1H), 7.70 (td, *J* = 7.6, 1.6 Hz, 1H), 7.63 (td, *J* = 7.6, 1.6 Hz, 1H), 7.12–7.06 (m, 2H), 7.04 (d, *J* = 9.2 Hz, 1H), 6.95–6.89 (m, 2H), 6.85–6.79 (m, 2H), 3.86 (s, 3H), 3.82 (d, *J* = 16.6 Hz, 1H), 3.69 (d, *J* = 15.6 Hz, 1H), 3.12 (d, *J* = 15.6 Hz, 1H), 3.11 (d, *J* = 16.6 Hz, 1H), 2.75 (s, 3H), 2.29 (s, 3H); ^13^C NMR (125 MHz, CDCl_3_) δ 193.8, 193.6, 170.4, 160.9, 154.6, 141.3, 138.1, 135.6, 135.2, 134.2, 133.9, 132.2, 129.5 (2C), 128.5, 128.3, 125.9, 125.3 (2C), 124.0, 114.3, 109.7, 76.8, 67.9, 55.6, 44.4, 39.7, 28.0, 21.2; HRMS (ESI) calcd for C_29_H_25_N_2_O_5_ [M + H]^+^ 481.1758, found 481.1761.

**2′n**:

m.p. = 186–188 °C; ^1^H NMR (400 MHz, CDCl_3_) δ 8.04 (dd, *J* = 7.6, 1.2 Hz, 1H), 7.98 (dd, *J* = 7.6, 1.2 Hz, 1H), 7.78 (td, *J* = 7.6, 1.2 Hz, 1H), 7.72 (td, *J* = 7.6, 1.2 Hz, 1H), 7.31 (d, *J* = 8.4 Hz, 1H), 7.09–7.03 (m, 2H), 6.98 (dd, *J* = 8.4, 2.4 Hz, 1H), 6.88 (d, *J* = 2.4 Hz, 1H), 6.62–6.46 (m, 2H), 3.87 (s, 3H), 3.86 (d, *J* = 16.8 Hz, 1H), 3.69 (d, *J* = 18.8 Hz, 1H), 3.16 (d, *J* = 16.8 Hz, 1H), 2.98 (d, *J* = 18.8 Hz, 1H), 2.57 (s, 3H), 2.30 (s, 3H); ^13^C NMR (125 MHz, CDCl_3_) δ 195.4, 191.1, 171.3, 160.7, 154.3, 139.4, 138.2, 136.4, 135.3, 135.0, 134.6, 132.7, 129.4 (2C), 128.2, 127.1, 127.1, 126.5, 125.3 (2C), 114.6, 109.6, 73.6, 66.9, 55.7, 46.5, 39.9, 27.2, 21.2; HRMS (ESI) calcd for C_29_H_25_N_2_O_5_ [M + H]^+^ 481.1758, found 481.1757.

(±)-(1′R*,4R*)-3,5’-dimethyl-1-(*p-*tolyl)-1″*H*,3′*H*-dispiro[imidazolidine-4,2′-indene-1′,2″-naphthalene]-1″,2,4″,5(3″*H*)-tetraone **2o** and (±)-(1′S*,4R*)-3,5’-dimethyl-1-(*p-*tolyl)-1″*H*,3′*H*-dispiro[imidazolidine-4,2′-indene-1′,2″-naphthalene]-1″,2,4″,5(3″*H*)-tetraone **2′o**.

Compounds **2o** and **2′o** were prepared according to the general procedure. Purification by column chromatography (silica gel, PET/EtOAc = 5:1, *v*/*v*) generated compounds **2o** (white solid, 121 mg, 26%) and **2′o** (white solid, 261 mg, 56%).

**2o**:

m.p. = 183–185 °C; ^1^H NMR (400 MHz, CDCl_3_) δ 8.04 (dd, *J* = 7.6, 1.2 Hz, 1H), 7.95 (dd, *J* = 7.6, 1.2 Hz, 1H), 7.70 (td, *J* = 7.6, 1.2 Hz, 1H), 7.63 (td, *J* = 7.6, 1.2 Hz, 1H), 7.24–7.17 (m, 2H), 7.10–7.06 (m, 2H), 7.02 (d, *J* = 7.6 Hz, 1H), 6.86–6.79 (m, 2H), 3.81 (d, *J* = 16.8 Hz, 1H), 3.72 (d, *J* = 15.6 Hz, 1H), 3.12 (d, *J* = 15.6 Hz, 1H), 3.12 (d, *J* = 16.8 Hz, 1H), 2.73 (s, 3H), 2.42 (s, 3H), 2.29 (s, 3H); ^13^C NMR (125 MHz, CDCl_3_) δ 193.9, 193.4, 170.5, 154.7, 139.9, 139.7, 138.2, 137.4, 135.7, 135.3, 134.2, 133.9, 129.6 (2C), 129.3, 128.6, 128.4, 126.0, 125.3 (2C), 125.1, 122.9, 76.8, 68.3, 44.3, 39.7, 28.1, 21.6, 21.2; HRMS (ESI) calcd for C_29_H_25_N_2_O_4_ [M + H]^+^ 465.1809, found 465.1811.

**2′o**:

m.p. = 184–186 °C; ^1^H NMR (400 MHz, CDCl_3_) δ 8.04 (d, *J* = 7.6 Hz, 1H), 7.98 (d, *J* = 7.6 Hz, 1H), 7.78 (t, *J* = 7.6 Hz, 1H), 7.72 (t, *J* = 7.6 Hz, 1H), 7.33–7.22 (m, 2H), 7.18 (s, 1H), 7.06 (d, *J* = 8.0 Hz, 2H), 6.58 (d, *J* = 8.0 Hz, 2H), 3.85 (d, *J* = 16.8 Hz, 1H), 3.69 (d, *J* = 18.8 Hz, 1H), 3.17 (d, *J* = 16.8 Hz, 1H), 2.99 (d, *J* = 18.8 Hz, 1H), 2.55 (s, 3H), 2.42 (s, 3H), 2.30 (s, 3H); ^13^C NMR (125 MHz, CDCl_3_) δ 195.3, 191.1, 171.4, 154.3, 139.4, 138.2, 138.0, 137.8, 136.4, 135.3, 135.0, 134.6, 129.4, 129.4 (2C), 128.2, 127.1, 127.1, 125.4, 125.3 (2C), 125.1, 73.5, 67.3, 46.4, 39.8, 27.2, 21.6, 21.2; HRMS (ESI) calcd for C_29_H_25_N_2_O_4_ [M + H]^+^ 465.1809, found 465.1805.

(±)-(1′R*,4R*)-6’-fluoro-3-methyl-1-(*p*-tolyl)-1″*H*,3′*H*-dispiro[imidazolidine-4,2′-indene-1′,2″-naphthalene]-1″,2,4″,5(3″*H*)-tetraone **2p** and (±)-(1′S*,4R*)-6’-fluoro-3-methyl-1-(*p-*tolyl)-1″*H*,3′*H*-dispiro[imidazolidine-4,2′-indene-1′,2″-naphthalene]-1″,2,4″,5(3″*H*)-tetraone **2′p**.

Compounds **2p** and **2′p** were prepared according to the general procedure. Purification by column chromatography (silica gel, PET/EtOAc = 5:1, *v*/*v*) generated compounds **2p** (white solid, 155 mg, 33%) and **2′p** (white solid, 267 mg, 57%).

**2p**:

m.p. = 181–183 °C; ^1^H NMR (400 MHz, CDCl_3_) δ 8.06–8.01 (m, 1H), 8.00–7.88 (m, 1H), 7.72 (td, *J* = 7.6, 1.6 Hz, 1H), 7.65 (td, *J* = 7.6, 1.6 Hz, 1H), 7.37 (dd, *J* = 8.4, 5.2 Hz, 1H), 7.17–7.06 (m, 3H), 6.86 (dd, *J* = 8.4, 2.4 Hz, 1H), 6.84–6.79 (m, 2H), 3.79 (d, *J* = 16.4 Hz, 1H), 3.67 (d, *J* = 15.6 Hz, 1H), 3.15 (d, *J* = 15.6 Hz, 1H), 3.14 (d, *J* = 16.4 Hz, 1H), 2.75 (s, 3H), 2.30 (s, 3H); ^13^C NMR (125 MHz, CDCl_3_) δ 193.2, 192.8, 170.1, 163.11 (d, *J* = 246.8 Hz), 154.6, 142.31 (d, *J* = 7.7 Hz), 138.3, 135.4 (2C), 135.3 (d, *J* = 2.5 Hz), 134.2, 134.1, 129.6 (2C), 128.5, 128.4, 126.1, 125.70 (d, *J* = 8.7 Hz), 125.3 (2C), 116.81 (d, *J* = 22.6 Hz), 110.78 (d, *J* = 23.4 Hz), 77.0, 68.4, 44.1, 39.1, 28.1, 21.3; ^19^F{H} NMR (376 MHz, CDCl_3_) δ −113.2; HRMS (ESI) calcd for C_28_H_22_FN_2_O_4_ [M + H]^+^ 469.1558, found 469.1561.

**2′p**:

m.p. = 180–182 °C; ^1^H NMR (400 MHz, CDCl_3_) δ 8.04 (dd, *J* = 7.6, 1.6 Hz, 1H), 7.98 (dd, *J* = 7.6, 1.6 Hz, 1H), 7.80 (td, *J* = 7.6, 1.6 Hz, 1H), 7.74 (td, *J* = 7.6, 1.6 Hz, 1H), 7.37–7.29 (m, 1H), 7.16–7.08 (m, 2H), 7.06 (d, *J* = 8.0 Hz, 2H), 6.57 (d, *J* = 8.0 Hz, 2H), 3.84 (d, *J* = 16.4 Hz, 1H), 3.71 (d, *J* = 18.4 Hz, 1H), 3.19 (d, *J* = 16.4 Hz, 1H), 2.99 (d, *J* = 18.4 Hz, 1H), 2.56 (s, 3H), 2.30 (s, 3H); ^13^C NMR (125 MHz, CDCl_3_) δ 194.6, 190.5, 170.9, 162.9 (d, *J* = 246.7 Hz), 154.2, 142.7 (d, *J* = 8.2 Hz), 138.3, 136.0, 135.3, 135.2, 134.9, 133.3 (d, *J* = 2.5 Hz), 129.4 (2C), 128.1, 127.2, 127.2, 125.8 (d, *J* = 8.8 Hz), 125.3 (2C), 116.7 (d, *J* = 22.7 Hz), 113.1 (d, *J* = 23.5 Hz), 73.8, 67.4, 45.9, 39.1, 27.2, 21.2; ^19^F{H} NMR (376 MHz, CDCl_3_) δ −112.8; HRMS (ESI) calcd for C_28_H_22_FN_2_O_4_ [M + H]^+^ 469.1558, found 469.1558.

(±)-(1′R*,4R*)-5’-chloro-3-methyl-1-(*p-*tolyl)-1″*H*,3′*H*-dispiro[imidazolidine-4,2′-indene-1′,2″-naphthalene]-1″,2,4″,5(3″*H*)-tetraone **2q** and (±)-(1′S*,4R*)-5’-chloro-3-methyl-1-(*p-*tolyl)-1″*H*,3′*H*-dispiro[imidazolidine-4,2′-indene-1′,2″-naphthalene]-1″,2,4″,5(3″*H*)-tetraone **2′q**.

Compounds **2q** and **2′q** were prepared according to the general procedure. Purification by column chromatography (silica gel, PET/EtOAc = 5:1, *v*/*v*) generated compounds **2q** (white solid, 121 mg, 25%) and **2′q** (white solid, 267 mg, 55%).

**2q**:

m.p. = 185–187 °C; ^1^H NMR (400 MHz, CDCl_3_) δ 8.03 (dd, *J* = 7.6, 1.2 Hz, 1H), 7.95 (dd, *J* = 7.6, 1.2 Hz, 1H), 7.71 (td, *J* = 7.6, 1.2 Hz, 1H), 7.65 (td, *J* = 7.6, 1.2 Hz, 1H), 7.45–7.35 (m, 2H), 7.12–7.05 (m, 3H), 6.85–6.75 (m, 2H), 3.82 (d, *J* = 16.8 Hz, 1H), 3.69 (d, *J* = 15.6 Hz, 1H), 3.15 (d, *J* = 16.8 Hz, 1H), 3.13 (d, *J* = 15.6 Hz, 1H), 2.75 (s, 3H), 2.30 (s, 3H); ^13^C NMR (125 MHz, CDCl_3_) δ 193.3, 192.9, 170.1, 154.6, 141.7, 138.8, 138.4, 135.5, 135.4 (2C), 134.2, 134.1, 129.6 (2C), 128.8, 128.4 (2C), 126.1, 125.3 (2C), 124.8, 124.4, 76.7, 68.0, 44.1, 39.4, 28.2, 21.3; HRMS (ESI) (ESI) calcd for C_28_H_22_ClN_2_O_4_ [M + H]^+^ 485.1263, found 485.1261.

**2′q**:

m.p. = 184–186 °C; ^1^H NMR (400 MHz, CDCl_3_) δ 8.04 (d, *J* = 7.6 Hz, 1H), 7.98 (d, *J* = 7.6 Hz, 1H), 7.79 (t, *J* = 7.6 Hz, 1H), 7.73 (t, *J* = 7.6 Hz, 1H), 7.47–7.31 (m, 3H), 7.06 (d, *J* = 8.4 Hz, 2H), 6.58 (d, *J* = 8.4 Hz, 2H), 3.86 (d, *J* = 16.8 Hz, 1H), 3.69 (d, *J* = 18.4 Hz, 1H), 3.20 (d, *J* = 16.8 Hz, 1H), 2.98 (d, *J* = 18.4 Hz, 1H), 2.55 (s, 3H), 2.30 (s, 3H); ^13^C NMR (125 MHz, CDCl_3_) δ 194.7, 190.6, 170.9, 154.2, 139.8, 139.2, 138.4, 136.1, 135.3 (2C), 135.2, 134.9, 129.4 (2C), 128.9, 128.1, 127.3, 127.2, 127.0, 125.3 (2C), 124.8, 73.4, 67.0, 46.1, 39.5, 27.2, 21.2; HRMS (ESI) calcd for C_28_H_22_ClN_2_O_4_ [M + H]^+^ 485.1263, found 485.1265.

(±)-(1′R*,4R*)-4’-fluoro-3,6’-dimethyl-1-(*p-*tolyl)-1″*H*,3′*H*-dispiro[imidazolidine-4,2′-indene-1′,2″-naphthalene]-1″,2,4″,5(3″*H*)-tetraone **2r** and (±)-(1′S*,4R*)-4’-fluoro-3,6’-dimethyl-1-(*p-*tolyl)-1″*H*,3′*H*-dispiro[imidazolidine-4,2′-indene-1′,2″-naphthalene]-1″,2,4″,5(3″*H*)-tetraone **2′r**.

Compounds **2r** and **2′r** were prepared according to the general procedure. Purification by column chromatography (silica gel, PET/EtOAc = 5:1, *v*/*v*) generated compounds **2r** (white solid, 159 mg, 33%) and **2′r** (white solid, 246 mg, 51%).

**2r**:

m.p. = 180–182 °C; ^1^H NMR (400 MHz, CDCl_3_) δ 8.04 (dd, *J* = 7.6, 1.2 Hz, 1H), 7.95 (dd, *J* = 7.6, 1.2 Hz, 1H), 7.71 (td, *J* = 7.6, 1.2 Hz, 1H), 7.64 (td, *J* = 7.6, 1.2 Hz, 1H), 7.13–7.05 (m, 2H), 6.95 (d, *J* = 9.6 Hz, 1H), 6.85–6.78 (m, 2H), 6.75 (s, 1H), 3.72 (d, *J* = 16.8 Hz, 1H), 3.71 (d, *J* = 15.6 Hz, 1H), 3.25 (d, *J* = 16.8 Hz, 1H), 3.13 (d, *J* = 15.6 Hz, 1H), 2.76 (s, 3H), 2.40 (s, 3H), 2.30 (s, 3H); ^13^C NMR (125 MHz, CDCl_3_) δ 193.3, 192.8, 170.1, 158.4 (d, *J* = 248.8 Hz), 154.6, 143.3 (d, *J* = 5.8 Hz), 141.3 (d, *J* = 6.8 Hz), 138.3, 135.4, 135.4, 134.2 (2C), 129.6 (2C), 128.5, 128.4, 126.0, 125.3 (2C), 123.3 (d, *J* = 18.8 Hz), 119.6 (d, *J* = 2.6 Hz), 116.9 (d, *J* = 19.7 Hz), 76.8, 68.6, 44.2, 35.6, 28.0, 21.7, 21.2; ^19^F{H} NMR (376 MHz, CDCl_3_) δ −118.0; HRMS (ESI) calcd for C_29_H_24_FN_2_O_4_ [M + H]^+^ 483.1715, found 483.1714.

**2′r**:

m.p. = 184–186 °C; ^1^H NMR (400 MHz, CDCl_3_) δ 8.04 (dd, *J* = 8.0, 1.2 Hz, 1H), 8.02–7.96 (m, 1H), 7.84–7.77 (m, 1H), 7.77–7.70 (m, 1H), 7.07 (d, *J* = 8.0 Hz, 2H), 7.00 (s, 1H), 6.93 (d, *J* = 9.6 Hz, 1H), 6.62–6.55 (m, 2H), 3.76 (d, *J* = 16.8 Hz, 1H), 3.70 (d, *J* = 18.4 Hz, 1H), 3.30 (d, *J* = 16.8 Hz, 1H), 3.02 (d, *J* = 18.4 Hz, 1H), 2.58 (s, 3H), 2.44 (s, 3H), 2.30 (s, 3H); ^13^C NMR (100 MHz, CDCl_3_) δ 194.7, 190.7, 171.0, 158.5 (d, *J* = 248.0 Hz), 154.2, 143.7 (d, *J* = 5.6 Hz), 141.5 (d, *J* = 6.4 Hz), 138.4, 136.1, 135.3, 135.1, 134.9, 129.4 (2C), 128.2, 127.2 (2C), 125.4 (2C), 121.9, 121.9 (d, *J* = 2.8 Hz), 121.4 (d, *J* = 18.8 Hz), 116.5 (d, *J* = 19.5 Hz), 73.6, 67.7, 46.1, 35.7, 27.1, 21.7, 21.2; ^19^F{H} NMR (376 MHz, CDCl_3_) δ −117.6; HRMS (ESI) (ESI) calcd for C_29_H_24_FN_2_O_4_ [M + H]^+^ 483.1715, found 483.1717.

(±)-(1′R*,4R*)-3-benzyl-1-(*p-*tolyl)-1″*H*,3′*H*-dispiro[imidazolidine-4,2′-indene-1′,2″-naphthalene]-1″,2,4″,5(3″*H*)-tetraone **2s** and (±)-(1′S*,4R*)-3-benzyl-1-(*p-*tolyl)-1″*H*,3′*H*-dispiro[imidazolidine-4,2′-indene-1′,2″-naphthalene]-1″,2,4″,5(3″*H*)-tetraone **2′s**.

Compounds **2s** and **2′s** were prepared according to the general procedure. Purification by column chromatography (silica gel, PET/EtOAc = 5:1, *v*/*v*) generated compounds **2s** (white solid, 137 mg, 26%) and **2′s** (white solid, 290 mg, 55%).

**2s**:

m.p. = 182–184 °C; ^1^H NMR (400 MHz, CDCl_3_) δ 8.02 (d, *J* = 7.6 Hz, 1H), 7.92 (d, *J* = 7.6 Hz, 1H), 7.69 (t, *J* = 7.6 Hz, 1H), 7.61 (t, *J* = 7.6 Hz, 1H), 7.51–7.42 (m, 2H), 7.27–7.18 (m, 4H), 7.15–7.08 (m, 3H), 6.94–6.85 (m, 4H), 4.92 (d, *J* = 15.2 Hz, 1H), 3.70 (d, *J* = 16.8 Hz, 1H), 3.69 (d, *J* = 15.2 Hz, 1H), 3.68 (d, *J* = 16.0 Hz, 1H), 3.08 (d, *J* = 16.0 Hz, 1H), 2.86 (d, *J* = 16.8 Hz, 1H), 2.31 (s, 3H); ^13^C NMR (125 MHz, CDCl_3_) δ 193.9, 193.2, 170.3, 155.3, 140.4, 140.3, 138.2, 137.1, 135.5, 135.3, 134.1, 134.0, 129.5 (2C), 129.5, 128.6 (2C), 128.5 (2C), 128.5 (2C), 128.4, 128.2, 126.0, 125.2, 125.2 (2C), 123.0, 78.0, 68.8, 45.7, 43.6, 39.9, 21.3; HRMS (ESI) calcd for C_34_H_27_N_2_O_4_ [M + H]^+^ 527.1965, found 527.1967.

**2′s**:

m.p. = 180–182 °C; ^1^H NMR (400 MHz, CDCl_3_) δ 8.06–7.99 (m, 2H), 7.81 (td, *J* = 7.6, 1.2 Hz, 1H), 7.72 (td, *J* = 7.6, 1.2 Hz, 1H), 7.47 (dd, *J* = 4.4, 1.2 Hz, 2H), 7.38 (dt, *J* = 8.4, 4.0 Hz, 1H), 7.22–7.13 (m, 3H), 7.07 (d, *J* = 7.6 Hz, 2H), 6.97 (d, *J* = 7.6 Hz, 1H), 6.80 (d, *J* = 7.2 Hz, 2H), 6.64 (d, *J* = 8.4 Hz, 2H), 4.88 (d, *J* = 16.0 Hz, 1H), 3.66 (d, *J* = 18.8 Hz, 2H), 3.52 (d, *J* = 16.0 Hz, 1H), 2.98 (d, *J* = 18.8 Hz, 1H), 2.76 (d, *J* = 16.8 Hz, 1H), 2.30 (s, 3H); ^13^C NMR (125 MHz, CDCl_3_) δ 195.4, 190.8, 171.2, 154.7, 141.0, 138.2, 137.9, 137.5, 136.4, 135.3, 135.2, 134.9, 129.3 (2C), 129.0, 128.5, 128.5 (2C), 128.3, 128.1 (2C), 127.9, 127.3 (2C), 125.7, 125.4, 125.2 (2C), 74.4, 68.0, 46.6, 44.7, 40.4, 21.3; HRMS (ESI) calcd for C_34_H_27_N_2_O_4_ [M + H]^+^ 527.1965, found 527.1961.

The general procedure for the preparation of (±)-*tert*-butyl (1′R*,4R*)-3-methyl-2,4″,5,7″-tetraoxo-1-(4-(trifluoromethyl)phenyl)-4″,7″-dihydro-3′*H*-dispiro[imidazolidine-4,2′-indene-1′,6″-indole]-1″(5″*H*)-carboxylaten **5a** and (±)-*tert*-butyl (1′S*,4R*)-3-methyl-2,4″,5,7″0-tetraoxo-1-(4-(trifluoromethyl)phenyl)-4″,7″-dihydro-3′*H*-dispiro[imidazolidine-4,2′-indene-1′,6″-indole]-1″(5″*H*)-carboxylate **5’a** is as follows:

To a solution of 6-(2-((3-methyl-2,5-dioxo-1-(4-(trifluoromethyl)phenyl)imidazolidin-4-yl)methyl)phenyl)-1H-indole-4,7-dione **4a** (493 mg, 1.0 mmol) in CH_2_Cl_2_ (5 mL) and CH_3_OH (1 mL), NaOH (8 mg, 0.2 mmol) was added at −20 °C under argon. Then, the reaction was stirred at this temperature for 1 h. The mixture was quenched with 1N HCl solution and extracted with CH_2_Cl_2_ (3 × 20 mL), washed with saturated NaCl solution, dried over anhydrous Na_2_SO_4_, and concentrated under reduced pressure.

The residue was dissolved in THF (10 mL), and DMAP (49 mg, 0.4 mmol) was added. The mixture was stirred, followed by the dropwise addition of (Boc)_2_O (0.46 mL, 2 mmol). The reaction was allowed to proceed at room temperature for 3 h. The mixture was quenched with hydrochloric acid, extracted with CH_2_Cl_2_ (3 × 20 mL), washed with saturated NaCl solution, dried over anhydrous Na_2_SO_4_, and concentrated under reduced pressure. The residue was purified by column chromatography (petroleum ether/EtOAc = 5:1) to afford compound **5a** (white solid, 184 mg, 31%) and compound **5’a** (white solid, 303 mg, 51%).

**5a**:

m.p. = 163–165 °C; ^1^H NMR (400 MHz, CDCl_3_) δ 7.43 (d, *J* = 3.2 Hz, 1H), 7.42–7.35 (m, 3H), 7.24–7.14 (m, 5H), 6.52 (d, *J* = 3.2 Hz, 1H), 3.79 (d, *J* = 16.4 Hz, 1H), 3.57 (d, *J* = 16.4 Hz, 1H), 3.21 (d, *J* = 16.4 Hz, 1H), 3.00 (d, *J* = 16.4 Hz, 1H), 2.71 (s, 3H), 1.50 (s, 9H); ^13^C NMR (125 MHz, CDCl_3_) δ 190.1, 183.0, 170.6, 154.3, 148.4, 147.1, 139.7, 139.4, 134.3, 132.5, 130.0, 129.9, 129.5, 128.3, 127.2 (2C), 124.5, 123.7, 121.4 (2C), 120.4 (q, *J* = 248.8 Hz, 1C), 106.7, 86.7, 76.5, 70.4, 46.0, 39.0, 28.2, 27.5 (3C); ^19^F{H} NMR (376 MHz, CDCl_3_) δ −57.8; HRMS (ESI) calcd for C_31_H_27_F_3_N_3_O_6_ [M + H]^+^ 594.1846, found 594.1848.

**5’a**:

m.p. = 161–163 °C; ^1^H NMR (400 MHz, CDCl_3_) δ 7.50 (d, *J* = 3.2 Hz, 1H), 7.44–7.35 (m, 4H), 7.22–7.17 (m, 2H), 7.11–7.06 (m, 2H), 6.62 (d, *J* = 3.2 Hz, 1H), 3.90 (d, *J* = 16.8 Hz, 1H), 3.56 (d, *J* = 18.4 Hz, 1H), 3.21 (d, *J* = 16.8 Hz, 1H), 2.90 (d, *J* = 18.4 Hz, 1H), 2.60 (s, 3H), 1.61 (s, 9H); ^13^C NMR (125 MHz, CDCl_3_) δ 188.2, 183.6, 171.3, 153.4, 148.3, 146.7, 140.1, 137.5, 135.1, 133.7, 130.3, 129.7, 129.3, 128.4, 126.9 (2C), 126.1, 124.5, 121.4 (2C), 120.4 (q, *J* = 256.8 Hz, 1C), 107.8, 87.4, 73.6, 68.6, 48.5, 39.8, 27.8 (3C), 27.6; ^19^F{H} NMR (376 MHz, CDCl_3_) δ −57.8; HRMS (ESI) calcd for C_31_H_27_F_3_N_3_O_6_ [M + H]^+^ 594.1846, found 594.1847.

(±)-*tert*-butyl (1′R*,4R*)-1-(4-chlorophenyl)-3-methyl-2,4″,5,7″-tetraoxo-4″,7″-dihydro-3′*H*-dispiro[imidazolidine-4,2′-indene-1′,6″-indole]-1″(5″*H*)-carboxylate **5b** and (±)-*tert*-butyl (1′S*,4R*)-1-(4-chlorophenyl)-3-methyl-2,4″,5,7″-tetraoxo-4″,7″-dihydro-3′*H*-dispiro[imidazolidine-4,2′-indene-1′,6″-indole]-1″(5″*H*)-carboxylate **5’b**.

Compounds **5b** and **5’b** were prepared according to the general procedure. Purification by column chromatography (silica gel, PET/EtOAc = 5:1, *v*/*v*) generated compounds **5b** (white solid, 157 mg, 28%) and **5’b** (white solid, 252 mg, 45%).

**5b**:

m.p. = 163–165 °C; ^1^H NMR (400 MHz, CDCl_3_) δ 7.42 (d, *J* = 3.2 Hz, 1H), 7.40–7.36 (m, 3H), 7.37–7.29 (m, 2H), 7.17–7.14 (m, 1H), 7.13–7.06 (m, 2H), 6.51 (d, *J* = 3.2 Hz, 1H), 3.79 (d, *J* = 16.4 Hz, 1H), 3.57 (d, *J* = 16.4 Hz, 1H), 3.19 (d, *J* = 16.4 Hz, 1H), 2.99 (d, *J* = 16.4 Hz, 1H), 2.69 (s, 3H), 1.51 (s, 9H); ^13^C NMR (100 MHz, CDCl_3_) δ 188.5, 181.5, 169.2, 153.3, 146.1, 138.9, 138.5, 133.6, 133.2, 131.7, 129.3, 129.2, 128.8, 128.4 (2C), 127.6, 126.3 (2C), 123.8, 123.1, 106.3, 86.6, 76.7, 70.6, 46.5, 39.7, 29.0, 28.3 (3C); HRMS (ESI) calcd for C_30_H_27_ClN_3_O_6_ [M + H]^+^ 560.1583, found 560.1581.

**5’b**:

m.p. = 165–167 °C; ^1^H NMR (400 MHz, CDCl_3_) δ 7.49 (d, *J* = 3.2 Hz, 1H), 7.44–7.34 (m, 4H), 7.34–7.29 (m, 2H), 7.02–6.96 (m, 2H), 6.61 (d, *J* = 3.2 Hz, 1H), 3.89 (d, *J* = 16.4 Hz, 1H), 3.55 (d, *J* = 18.4 Hz, 1H), 3.20 (d, *J* = 16.4 Hz, 1H), 2.89 (d, *J* = 18.4 Hz, 1H), 2.59 (s, 3H), 1.61 (s, 9H); ^13^C NMR (125 MHz, CDCl_3_) δ 188.2, 183.8, 171.3, 153.5, 146.7, 140.2, 137.7, 135.2, 133.8, 130.4, 129.9, 129.6, 129.3 (2C), 129.1, 128.4, 126.6 (2C), 126.1, 124.6, 107.9, 87.5, 73.6, 68.7, 48.5, 39.9, 27.9 (3C), 27.6; HRMS (ESI) calcd for C_30_H_27_ClN_3_O_6_ [M + H]^+^ 560.1583, found 560.1582.

(±)-*tert*-butyl (1′R*,4R*)-1-(4-chloro-3-(trifluoromethyl)phenyl)-3-methyl-2,4″,5,7″-tetraoxo-4″,7″-dihydro-3′*H*-dispiro[imidazolidine-4,2′-indene-1′,6″-indole]-1″(5″*H*)-carboxylate **5c** and (±)-*tert*-butyl (1′S*,4R*)-1-(4-chloro-3-(trifluoromethyl)phenyl)-3-methyl-2,4″,5,7″-tetraoxo-4″,7″-dihydro-3′*H*-dispiro[imidazolidine-4,2′-indene-1′,6″-indole]-1″(5″*H*)-carboxylate **5’c**.

Compounds **5c** and **5’c** were prepared according to the general procedure. Purification by column chromatography (silica gel, PET/EtOAc = 5:1, *v*/*v*) generated compounds **5c** (white solid, 157 mg, 25%) and **5’c** (white solid, 327 mg, 52%).

**5c**:

m.p. = 168–180 °C; ^1^H NMR (400 MHz, CDCl_3_) δ 7.51 (d, *J* = 8.8 Hz, 1H), 7.44 (d, *J* = 3.2 Hz, 1H), 7.42 (d, *J* = 2.4 Hz, 1H), 7.40–7.35 (m, 4H), 7.17–7.11 (m, 1H), 6.50 (d, *J* = 3.2 Hz, 1H), 3.78 (d, *J* = 16.4 Hz, 1H), 3.57 (d, *J* = 16.4 Hz, 1H), 3.20 (d, *J* = 16.4 Hz, 1H), 2.98 (d, *J* = 16.4 Hz, 1H), 2.71 (s, 3H), 1.50 (s, 9H); ^13^C NMR (125 MHz, CDCl_3_) δ 190.1, 183.0, 170.6, 153.9, 147.0, 139.6, 139.3, 134.4, 132.5, 132.1, 131.8, 130.3, 130.3, 129.8, 129.7, 129.1 (q, *J* = 32.0 Hz), 128.4, 124.9 (q, *J* = 5.3 Hz), 124.6, 123.8, 122.4 (q, *J* = 273.4 Hz), 106.8, 86.9, 76.6, 70.8, 46.3, 39.0, 28.3, 27.5 (3C); ^19^F{H} NMR (376 MHz, CDCl_3_) δ −62.7; HRMS (ESI) calcd for C_31_H_26_ClF_3_N_3_O_6_ [M + H]^+^ 628.1457, found 628.1459.

**5’c**:

m.p. = 166–168 °C; ^1^H NMR (400 MHz, CDCl_3_) δ 7.49 (d, *J* = 8.4 Hz, 1H), 7.46 (d, *J* = 3.2 Hz, 1H), 7.43–7.34 (m, 4H), 7.32–7.26 (m, 2H), 6.54 (d, *J* = 3.2 Hz, 1H), 3.90 (d, *J* = 16.8 Hz, 1H), 3.51 (d, *J* = 18.4 Hz, 1H), 3.21 (d, *J* = 16.8 Hz, 1H), 2.89 (d, *J* = 18.4 Hz, 1H), 2.59 (s, 3H), 1.61 (s, 9H); ^13^C NMR (125 MHz, CDCl_3_) δ 188.0, 183.6, 171.1, 152.9, 146.5, 139.9, 137.5, 135.1, 133.8, 132.1, 131.7, 130.3, 130.1, 129.6, 129.4, 129.0 (q, *J* = 31.1 Hz), 128.5, 126.2, 124.6, 124.6 (q, *J* = 5.3 Hz), 122.35 (q, *J* = 273.8 Hz), 107.9, 87.6, 73.8, 68.8, 48.5, 39.7, 27.8 (3C), 27.6; ^19^F{H} NMR (376 MHz, CDCl_3_) δ −62.7; HRMS (ESI) calcd for C_31_H_26_ClF_3_N_3_O_6_ [M + H]^+^ 628.1457, found 628.1452.

(±)-*tert*-butyl (1′R*,4R*)-1-(4-fluorophenyl)-3-methyl-2,4″,5,7″-tetraoxo-4″,7″-dihydro-3′*H*-dispiro[imidazolidine-4,2′-indene-1′,6″-indole]-1″(5″*H*)-carboxylate **5d** and (±)-*tert*-butyl (1′S*,4R*)-1-(4-fluorophenyl)-3-methyl-2,4″,5,7″-tetraoxo-4″,7″-dihydro-3′*H*-dispiro[imidazolidine-4,2′-indene-1′,6″-indole]-1″(5″*H*)-carboxylate **5’d**.

Compounds **5d** and **5’d** were prepared according to the general procedure. Purification by column chromatography (silica gel, PET/EtOAc = 5:1, *v*/*v*) generated compounds **5d** (white solid, 141 mg, 26%) and **5’d** (white solid, 261 mg, 48%).

**5d**:

m.p. = 166–168 °C; ^1^H NMR (400 MHz, CDCl_3_) δ 7.42 (d, *J* = 3.2 Hz, 1H), 7.41–7.35 (m, 3H), 7.19–6.99 (m, 5H), 6.52 (d, *J* = 3.2 Hz, 1H), 3.79 (d, *J* = 16.4 Hz, 1H), 3.57 (d, *J* = 16.4 Hz, 1H), 3.19 (d, *J* = 16.4 Hz, 1H), 3.00 (d, *J* = 16.4 Hz, 1H), 2.69 (s, 3H), 1.50 (s, 9H); ^13^C NMR (125 MHz, CDCl_3_) δ 190.3, 183.1, 170.8, 162.1 (d, *J* = 248.1 Hz), 154.7, 147.2, 139.8, 139.5, 134.5, 132.5, 130.1, 129.5, 128.3, 127.8 (d, *J* = 8.8 Hz, 2C), 127.4 (d, *J* = 3.1 Hz), 124.5, 123.8, 116.0 (d, *J* = 22.8 Hz, 2C), 106.7, 86.7, 76.7, 70.4, 46.0, 39.1, 28.2, 27.5 (3C); ^19^F{H} NMR (376 MHz, CDCl_3_) δ −112.7; HRMS (ESI) calcd for C_30_H_27_FN_3_O_6_ [M + H]^+^ 544.1878, found 544.1872.

**5’d**:

m.p. = 165–167 °C; ^1^H NMR (400 MHz, CDCl_3_) δ 7.51 (d, *J* = 3.2 Hz, 1H), 7.46–7.33 (m, 4H), 7.08–6.94 (m, 4H), 6.62 (d, *J* = 3.2 Hz, 1H), 3.89 (d, *J* = 17.2 Hz, 1H), 3.56 (d, *J* = 18.4 Hz, 1H), 3.20 (d, *J* = 17.2 Hz, 1H), 2.89 (d, *J* = 18.4 Hz, 1H), 2.59 (s, 3H), 1.61 (s, 9H); ^13^C NMR (100 MHz, CDCl_3_) δ 188.2, 183.8, 171.5, 161.8 (d, *J* = 248.2 Hz), 153.7, 146.8, 140.2, 137.7, 135.3, 133.8, 130.3, 129.3, 128.4, 127.4 (d, *J* = 8.6 Hz, 2C), 127.3 (d, *J* = 3.1 Hz), 126.1, 124.6, 115.95 (d, *J* = 22.7 Hz, 2C), 107.9, 87.5, 73.6, 68.7, 48.5, 39.8, 27.8 (3C), 27.6; ^19^F{H} NMR (376 MHz, CDCl_3_) δ −112.8; HRMS (ESI) calcd for C_30_H_27_FN_3_O_6_ [M + H]^+^ 544.1878, found 544.1877.

(±)-*tert*-butyl 1-ethyl-3-methyl-2,4″,5,7″-tetraoxo-4″,7″-dihydro-3′*H*-dispiro[imidazolidine-4,2′-indene-1′,6″-indole]-1″(5″*H*)-carboxylate **5e** and **5’e**.

Compounds **5e** and **5’e** were prepared according to the general procedure. Purification by column chromatography (silica gel, PET/EtOAc = 5:1, *v*/*v*) generated compounds **5e** and **5’e** (white solid, 325 mg, 68%). m.p. = 161–163 °C; ^1^H NMR (500 MHz, CDCl_3_) δ 7.53 (d, *J* = 3.5 Hz, 1H), 7.49 (d, *J* = 3.5 Hz, 0.7H), 7.41–7.32 (m, 6.1H), 7.12 (d, *J* = 6.5 Hz, 0.7H), 6.65 (d, *J* = 3.5 Hz, 1H), 6.54 (d, *J* = 3.5 Hz, 0.7H), 3.79 (d, *J* = 17.0 Hz, 1H), 3.71 (d, *J* = 16.5 Hz, 0.7H), 3.52 (d, *J* = 18.5 Hz, 1H), 3.51 (d, *J* = 16.5 Hz, 0.7H), 3.43–3.21 (m, 3.4H), 3.05 (d, *J* = 17.0 Hz, 1H), 3.00 (d, *J* = 16.5 Hz, 0.7H), 2.91 (d, *J* = 16.5 Hz, 0.7H), 2.83 (d, *J* = 18.5 Hz, 1H), 2.55 (s, 2.1H), 2.47 (s, 3H), 1.61 (s, 9H), 1.58 (s, 6.3H), 0.97 (t, *J* = 7.0 Hz, 2.1H), 0.71 (t, *J* = 7.0 Hz, 3H); ^13^C NMR (125 MHz, CDCl_3_) δ 190.1, 188.4, 183.8, 183.4, 172.2, 171.5, 155.6, 154.9, 147.3, 146.9, 140.6, 139.9, 137.8, 136.7, 135.5, 134.7, 133.7, 132.3, 130.3, 129.4, 129.1, 128.3, 128.2, 127.7, 125.9, 124.5, 124.3, 123.5, 107.8, 106.7, 87.2, 86.4, 76.9, 73.7, 69.5, 68.2, 48.5, 45.3, 39.4, 39.3, 34.1, 33.8, 27.8 (3C), 27.7, 27.6 (3C), 27.3, 13.2, 12.8; HRMS (ESI) calcd for C_26_H_28_N_3_O_6_ [M + H]^+^ 478.1973, found 478.1976.

(±)-*tert*-butyl 1-benzyl-3-methyl-2,4″,5,7″-tetraoxo-4″,7″-dihydro-3′*H*-dispiro[imidazolidine-4,2′-indene-1′,6″-indole]-1″(5″*H*)-carboxylate **5f** and **5’f**.

Compounds **5f** and **5’f** were prepared according to the general procedure. Purification by column chromatography (silica gel, PET/EtOAc = 5:1, *v*/*v*) generated compounds **5f** and **5’f** (white solid, 410 mg, 76%). m.p. = 166–178 °C; ^1^H NMR (400 MHz, CDCl_3_) δ 7.39–7.30 (m, 6.4H), 7.30–7.25 (m, 2.2H), 7.25–7.17 (m, 5.8H), 7.12–7.07 (m, 0.6H), 7.03 (d, *J* = 3.2 Hz, 1H), 6.23 (d, *J* = 3.2 Hz, 0.6H), 6.20 (d, *J* = 3.2 Hz, 1H), 4.42–4.39 (m, 3.2H), 3.80 (d, *J* = 16.8 Hz, 1H), 3.71 (d, *J* = 16.4 Hz, 0.6H), 3.48 (d, *J* = 18.4 Hz, 1H), 3.42 (d, *J* = 16.8 Hz, 0.6H), 3.04 (d, *J* = 16.8 Hz, 1H), 3.03 (d, *J* = 16.4 Hz, 0.6H), 2.91 (d, *J* = 16.8 Hz, 0.6H), 2.77 (d, *J* = 18.4 Hz, 1H), 2.58 (s, 1.8H), 2.47 (s, 3H), 1.61 (s, 9H), 1.58 (s, 5.2H); ^13^C NMR (125 MHz, CDCl_3_) ^13^C NMR (100 MHz, CDCl_3_) δ 189.7, 188.3, 183.5, 183.2, 172.0, 171.6, 155.8, 155.2, 147.3, 147.0, 140.8, 140.4, 139.7, 137.7, 135.7, 135.5, 135.4, 135.1, 130.5, 129.7, 129.3, 129.1 (2C), 129.0, 128.9 (2C), 128.7 (2C), 128.6 (2C), 128.4, 128.3, 128.3, 128.1, 127.9 (2C), 125.8, 124.5, 124.4, 123.6, 107.1, 106.7, 86.9, 86.4, 76.8, 73.9, 69.5, 68.0, 48.6, 45.3, 42.8, 42.6, 39.8, 39.6, 27.8, 27.8 (3C), 27.6 (3C), 27.5; HRMS (ESI) calcd for C_31_H_30_N_3_O_6_ [M + H]^+^ 540.2129, found 540.2129.

(±)-*tert*-butyl (1′R*,4R*)-3-methyl-2,4″,5,7″-tetraoxo-1-phenethyl-4″,7″-dihydro-3′*H*-dispiro[imidazolidine-4,2′-indene-1′,6″-indole]-1″(5″*H*)-carboxylate **5g** and (±)-*tert*-butyl (1′S*,4R*)-3-methyl-2,4″,5,7″-tetraoxo-1-(*p-*tolyl)-4″,7″-dihydro-3′*H*-dispiro[imidazolidine-4,2′-indene-1′,6″-indole]-1″(5″*H*)-carboxylate **5’g**.

Compounds **5g** and **5’g** were prepared according to the general procedure. Purification by column chromatography (silica gel, PET/EtOAc = 5:1, *v*/*v*) generated compounds **5g** (white solid, 183 mg, 33%) and **5’g** (white solid, 316 mg, 57%).

**5g**:

m.p. = 163–165 °C; ^1^H NMR (500 MHz, CDCl_3_) δ 7.50 (d, *J* = 3.0 Hz, 1H), 7.40–7.30 (m, 3H), 7.27–7.23 (m, 2H), 7.21–7.14 (m, 3H), 7.09 (d, *J* = 7.0 Hz, 1H), 6.55 (d, *J* = 3.0 Hz, 1H), 3.72 (d, *J* = 16.5 Hz, 1H), 3.57 (ddd, *J* = 13.5, 10.5, 6.0 Hz, 1H), 3.49 (ddd, *J* = 13.5, 10.5, 6.0 Hz, 1H), 3.41 (d, *J* = 16.5 Hz, 1H), 2.98 (d, *J* = 16.5 Hz, 1H), 2.85 (d, *J* = 16.5 Hz, 1H), 2.76 (td, *J* = 13.0, 11.0, 6.0 Hz, 1H), 2.62 (td, *J* = 13.0, 11.0, 6.0 Hz, 1H), 2.55 (s, 3H), 1.59 (s, 9H); ^13^C NMR (125 MHz, CDCl_3_) δ 190.3, 183.1, 171.5, 155.6, 147.4, 140.5, 139.9, 137.9 (2C), 132.5, 130.1, 129.4, 129.0 (2C), 128.6 (2C), 128.2, 126.7, 124.5, 123.4, 106.8, 86.6, 77.6, 69.2, 45.2, 40.2, 39.4, 33.5, 27.8, 27.7 (3C); HRMS (ESI) calcd for C_32_H_32_N_3_O_6_ [M + H]^+^ 554.2286, found 554.2288.

**5’g**:

m.p. = 164–166 °C; ^1^H NMR (500 MHz, CDCl_3_) δ 7.50 (d, *J* = 3.2 Hz, 1H), 7.42–7.27 (m, 6H), 7.22 (t, *J* = 7.4 Hz, 1H), 7.14 (d, *J* = 7.4 Hz, 2H), 6.65 (d, *J* = 3.2 Hz, 1H), 3.75 (d, *J* = 16.8 Hz, 1H), 3.51 (d, *J* = 18.4 Hz, 1H), 3.47–3.38 (m, 2H), 2.99 (d, *J* = 16.8 Hz, 1H), 2.83 (d, *J* = 18.4 Hz, 1H), 2.50–2.42 (m, 4H), 2.30 (ddd, *J* = 12.4, 11.6, 5.6 Hz, 1H), 1.61 (s, 9H);^13^C NMR (125 MHz, CDCl_3_) δ 188.4, 183.9, 172.3, 154.8, 146.9, 140.5, 137.8, 137.6, 135.5, 133.8, 130.3, 129.2, 128.9 (2C), 128.7 (2C), 128.3, 126.9, 126.0, 124.6, 107.8, 87.3, 73.8, 68.3, 48.5, 40.1, 39.5, 34.1, 27.9 (3C), 27.4; HRMS (ESI) calcd for C_32_H_32_N_3_O_6_ [M + H]^+^ 554.2286, found 554.2282.

(±)-*tert*-butyl (1′R*,4R*)-1-cyclohexyl-3-methyl-2,4″,5,7″-tetraoxo-4″,7″-dihydro-3′*H*-dispiro[imidazolidine-4,2′-indene-1′,6″-indole]-1″(5″*H*)-carboxylate **5h** and (±)-*tert*-butyl (1′S*,4R*)-1-cyclohexyl-3-methyl-2,4″,5,7″-tetraoxo-4″,7″-dihydro-3′*H*-dispiro[imidazolidine-4,2′-indene-1′,6″-indole]-1″(5″*H*)-carboxylate **5’h**.

Compounds **5h** and **5’h** were prepared according to the general procedure. Purification by column chromatography (silica gel, PET/EtOAc = 5:1, *v*/*v*) generated compounds **5h** (white solid, 133 mg, 25%) and **5’h** (white solid, 250 mg, 47%).

**5h**:

m.p. = 167–169 °C; ^1^H NMR (400 MHz, CDCl_3_) δ 7.49 (d, *J* = 3.2 Hz, 1H), 7.41–7.30 (m, 3H), 7.14–7.07 (m, 1H), 6.54 (d, *J* = 3.2 Hz, 1H), 3.68 (d, *J* = 16.4 Hz, 1H), 3.66 (tt, *J* = 12.4, 4.0 Hz, 1H), 3.48 (d, *J* = 16.4 Hz, 1H), 3.00 (d, *J* = 16.4 Hz, 1H), 2.89 (d, *J* = 16.4 Hz, 1H), 2.54 (s, 3H), 1.96 (qd, *J* = 12.4, 3.6 Hz, 1H), 1.73 (dd, *J* = 20.4, 12.0 Hz, 3H), 1.59 (s, 9H), 1.64–1.53 (m, 2H), 1.51–1.35 (m, 2H), 1.27–1.06 (m, 2H); ^13^C NMR (125 MHz, CDCl_3_) δ 190.2, 183.5, 171.5, 155.7, 147.4, 140.3, 140.0, 134.8, 132.4, 129.8, 129.3, 128.1, 124.4, 123.5, 106.8, 86.4, 76.4, 69.5, 51.9, 45.4, 39.6, 28.9, 28.8, 27.8, 27.7 (3C), 25.8, 25.8, 25.0; HRMS (ESI) calcd for C_30_H_34_N_3_O_6_ [M + H]^+^ 532.2442, found 532.2441.

**5’h**:

m.p. = 167–169 °C; ^1^H NMR (400 MHz, CDCl_3_) δ 7.53 (d, *J* = 3.2 Hz, 1H), 7.41–7.29 (m, 4H), 6.65 (d, *J* = 3.2 Hz, 1H), 3.77 (d, *J* = 16.8 Hz, 1H), 3.67 (tt, *J* = 13.2, 4.0 Hz, 1H), 3.51 (d, *J* = 18.4 Hz, 1H), 3.03 (d, *J* = 16.8 Hz, 1H), 2.80 (d, *J* = 18.4 Hz, 1H), 2.45 (s, 3H), 1.95–1.82 (m, 1H), 1.81–1.62 (m, 3H), 1.60 (s, 9H), 1.24–1.01 (m, 6H); ^13^C NMR (125 MHz, CDCl_3_) δ 188.5, 184.0, 172.3, 154.9, 147.0, 140.8, 137.9, 135.6, 133.7, 130.2, 129.0, 128.2, 125.8, 124.5, 107.9, 87.2, 73.0, 68.1, 51.5, 48.6, 39.8, 29.2, 28.7, 27.8 (3C), 27.3, 25.8, 25.7, 24.9; HRMS (ESI) calcd for C_30_H_34_N_3_O_6_ [M + H]^+^ 532.2442, found 532.2445.

(±)-*tert*-butyl (1′R*,4R*)-1-(*tert*-butyl)-3-methyl-2,4″,5,7″-tetraoxo-4″,7″-dihydro-3′*H*-dispiro[imidazolidine-4,2′-indene-1′,6″-indole]-1″(5″*H*)-carboxylate **5i** and (±)-*tert*-butyl (1′S*,4R*)-1-(*tert*-butyl)-3-methyl-2,4″,5,7″-tetraoxo-4″,7″-dihydro-3′*H*-dispiro[imidazolidine-4,2′-indene-1′,6″-indole]-1″(5″*H*)-carboxylate **5’i**.

Compounds **5i** and **5’i** were prepared according to the general procedure. Purification by column chromatography (silica gel, PET/EtOAc = 5:1, *v*/*v*) generated compounds **5i** (white solid, 132 mg, 26%) and **5’i** (white solid, 217 mg, 43%).

**5i**:

m.p. = 166–168 °C; ^1^H NMR (400 MHz, CDCl_3_) δ 7.51 (d, *J* = 3.2 Hz, 1H), 7.41–7.29 (m, 3H), 7.14–7.06 (m, 1H), 6.59 (d, *J* = 3.2 Hz, 1H), 3.69 (d, *J* = 16.4 Hz, 1H), 3.50 (d, *J* = 16.4 Hz, 1H), 2.97 (d, *J* = 16.4 Hz, 1H), 2.88 (d, *J* = 16.4 Hz, 1H), 2.52 (s, 3H), 1.58 (s, 9H), 1.32 (s, 9H); ^13^C NMR (125 MHz, CDCl_3_) δ 190.3, 184.0, 172.2, 156.3, 147.4, 140.1, 140.1, 135.0, 132.5, 129.7, 129.4, 128.1, 124.3, 123.7, 107.0, 86.4, 76.5, 69.9, 58.2, 45.6, 39.7, 28.1 (3C), 27.6 (3C), 27.5; HRMS (ESI) calcd for C_28_H_32_N_3_O_6_ [M + H]^+^ 506.2286, found 506.2288.

**5’i**:

m.p. = 164–166 °C; ^1^H NMR (400 MHz, CDCl_3_) δ 7.58 (d, *J* = 3.2 Hz, 1H), 7.39–7.29 (m, 4H), 6.69 (d, *J* = 3.2 Hz, 1H), 3.77 (d, *J* = 16.8 Hz, 1H), 3.47 (d, *J* = 18.4 Hz, 1H), 3.01 (d, *J* = 16.8 Hz, 1H), 2.80 (d, *J* = 18.4 Hz, 1H), 2.42 (s, 3H), 1.60 (s, 9H), 1.29 (s, 9H); ^13^C NMR (125 MHz, CDCl_3_) δ 188.6, 184.1, 172.6, 155.5, 147.1, 140.8, 138.1, 135.7, 134.0, 130.4, 129.0, 128.2, 125.8, 124.5, 107.9, 87.2, 73.0, 68.4, 58.1, 48.7, 39.7, 28.4 (3C), 27.8 (3C), 27.1; HRMS (ESI) calcd for C_28_H_32_N_3_O_6_ [M + H]^+^ 506.2286, found 506.2287.

(±)-*tert*-butyl (1′R*,4R*)-5’-chloro-3-methyl-2,4″,5,7″-tetraoxo-1-(*p-*tolyl)-4″,7″-dihydro-3′*H*-dispiro[imidazolidine-4,2′-indene-1′,6″-indole]-1″(5″*H*)-carboxylate **5j** and (±)-*tert*-butyl (1′S*,4R*)-5’-chloro-3-methyl-2,4″,5,7″-tetraoxo-1-(*p-*tolyl)-4″,7″-dihydro-3′*H*-dispiro[imidazolidine-4,2′-indene-1′,6″-indole]-1″(5″*H*)-carboxylate **5’j**.

Compounds **5j** and **5’j** were prepared according to the general procedure. Purification by column chromatography (silica gel, PET/EtOAc = 5:1, *v*/*v*) generated compounds **5j** (white solid, 166 mg, 29%) and **5’j** (white solid, 276 mg, 48%).

**5j**:

m.p. = 163–165 °C; ^1^H NMR (400 MHz, CDCl_3_) δ 7.43 (d, J = 3.2 Hz, 1H), 7.36 (s, 1H), 7.34 (d, J = 8.4 Hz, 1H), 7.15 (d, J = 8.4 Hz, 2H), 7.09 (d, J = 8.4 Hz, 1H), 6.93 (d, J = 8.4 Hz, 2H), 6.54 (d, J = 3.2 Hz, 1H), 3.73 (d, J = 16.8 Hz, 1H), 3.47 (d, J = 16.4 Hz, 1H), 3.17 (d, J = 16.8 Hz, 1H), 2.98 (d, J = 16.4 Hz, 1H), 2.71 (s, 3H), 2.32 (s, 3H), 1.47 (s, 9H); ^13^C NMR (100 MHz, CDCl_3_) δ 189.6, 182.9, 170.5, 154.9, 147.3, 141.5, 138.6, 138.4, 135.3, 134.2, 132.8, 130.2, 129.7 (2C), 128.8, 128.6, 125.8 (2C), 125.2, 124.8, 107.0, 86.7, 76.5, 70.0, 46.1, 38.8, 28.4, 27.5 (3C), 21.3; HRMS (ESI) calcd for C_31_H_29_ClN_3_O_6_ [M + H]^+^ 574.1740, found 574.1744.

**5’j**:

m.p. = 162–164 °C; ^1^H NMR (400 MHz, CDCl_3_) δ 7.52 (d, *J* = 3.2 Hz, 1H), 7.43–7.29 (m, 3H), 7.17–7.11 (m, 2H), 6.86–6.80 (m, 2H), 6.62 (d, *J* = 3.2 Hz, 1H), 3.86 (d, *J* = 16.8 Hz, 1H), 3.54 (d, *J* = 18.4 Hz, 1H), 3.17 (d, *J* = 16.8 Hz, 1H), 2.83 (d, *J* = 18.4 Hz, 1H), 2.59 (s, 3H), 2.33 (s, 3H), 1.61 (s, 9H); ^13^C NMR (125 MHz, CDCl_3_) δ 187.8, 183.5, 171.1, 153.9, 146.7, 139.8, 138.9, 138.3, 135.4, 135.1, 133.5, 130.5, 129.6 (2C), 128.7, 128.6, 127.3, 125.5 (2C), 124.8, 108.1, 87.5, 73.6, 68.1, 48.3, 39.5, 27.9 (3C), 27.6, 21.3; HRMS (ESI) calcd for C_31_H_29_ClN_3_O_6_ [M + H]^+^ 574.1740, found 574.1737.

(±)-*tert*-butyl (1′R*,4R*)-6’-methoxy-3-methyl-2,4″,5,7″-tetraoxo-1-(*p-*tolyl)-4″,7″-dihydro-3′*H*-dispiro[imidazolidine-4,2′-indene-1′,6″-indole]-1″(5″*H*)-carboxylate **5k** and (±)-*tert*-butyl (1′S*,4R*)-6’-methoxy-3-methyl-2,4″,5,7″-tetraoxo-1-(*p-*tolyl)-4″,7″-dihydro-3′*H*-dispiro[imidazolidine-4,2′-indene-1′,6″-indole]-1″(5″*H*)-carboxylate **5’k**.

Compounds **5k** and **5’k** were prepared according to the general procedure; the reaction was completed within 20 min. Purification by column chromatography (silica gel, PET/EtOAc = 5:1, *v*/*v*) generated compounds **5k** (white solid, 137 mg, 24%) and **5’k** (white solid, 279 mg, 49%).

**5k**:

m.p. = 166–168 °C; ^1^H NMR (400 MHz, CDCl_3_) δ 7.51 (d, *J* = 3.2 Hz, 1H), 7.24 (d, *J* = 8.4 Hz, 1H), 7.13 (d, *J* = 8.4 Hz, 2H), 6.97–6.90 (m, 2H), 6.86–6.79 (m, 2H), 6.62 (d, *J* = 3.2 Hz, 1H), 3.85 (s, 3H), 3.81 (d, *J* = 16.4 Hz, 1H), 3.56 (d, *J* = 18.4 Hz, 1H), 3.12 (d, *J* = 16.4 Hz, 1H), 2.88 (d, *J* = 18.4 Hz, 1H), 2.61 (s, 3H), 2.32 (s, 3H), 1.61 (s, 9H); ^13^C NMR (125 MHz, CDCl_3_) δ 190.1, 183.1, 170.7, 159.9, 155.0, 147.3, 141.4, 138.3, 134.5, 132.6, 131.5, 130.0, 129.7 (2C), 128.9, 125.9 (2C), 125.1, 115.2, 109.5, 106.8, 86.6, 76.9, 70.4, 55.6, 46.0, 38.5, 28.2, 27.6 (3C), 21.3; HRMS (ESI) calcd for C_32_H_32_N_3_O_7_ [M + H]^+^ 570.2235, found 570.2240.

**5’k**:

m.p. = 165–167 °C; ^1^H NMR (400 MHz, CDCl_3_) δ 7.43 (d, *J* = 3.2 Hz, 1H), 7.26 (d, *J* = 8.4 Hz, 1H), 7.16 (d, *J* = 8.4 Hz, 2H), 6.96 (d, *J* = 8.4 Hz, 2H), 6.93 (dd, *J* = 8.4, 2.4 Hz, 1H), 6.68 (d, *J* = 2.4 Hz, 1H), 6.54 (d, *J* = 3.2 Hz, 1H), 3.81 (s, 3H), 3.71 (d, *J* = 16.4 Hz, 1H), 3.51 (d, *J* = 16.4 Hz, 1H), 3.11 (d, *J* = 16.4 Hz, 1H), 3.00 (d, *J* = 16.4 Hz, 1H), 2.70 (s, 3H), 2.33 (s, 3H), 1.50 (s, 9H); ^13^C NMR (125 MHz, CDCl_3_) δ 188.3, 183.9, 171.5, 159.8, 154.1, 146.9, 141.8, 138.2, 135.4, 133.8, 130.3, 129.6 (2C), 129.5, 128.7, 125.6 (2C), 125.2, 115.8, 110.9, 108.0, 87.4, 74.0, 68.6, 55.7, 48.5, 39.2, 27.9 (3C), 27.6, 21.3; HRMS (ESI) calcd for C_32_H_32_N_3_O_7_ [M + H]^+^ 570.2235, found 570.2233.

(±)-*tert*-butyl (1′R*,4R*)-5’-methoxy-3-methyl-2,4″,5,7″-tetraoxo-1-(*p-*tolyl)-4″,7″-dihydro-3′*H*-dispiro[imidazolidine-4,2′-indene-1′,6″-indole]-1″(5″*H*)-carboxylate **5l** and (±)-*tert*-butyl (1′S*,4R*)-5’-methoxy-3-methyl-2,4″,5,7″-tetraoxo-1-(*p-*tolyl)-4″,7″-dihydro-3′*H*-dispiro[imidazolidine-4,2′-indene-1′,6″-indole]-1″(5″*H*)-carboxylate **5’l**.

Compounds **5l** and **5’l** were prepared according to the general procedure; the reaction was completed within 20 min. Purification by column chromatography (silica gel, PET/EtOAc = 5:1, *v*/*v*) generated compounds **5l** (white solid, 177 mg, 31%) and **5’l** (white solid, 302 mg, 53%).

**5l**:

m.p. = 169–171 °C; ^1^H NMR (400 MHz, CDCl_3_) δ 7.43 (d, *J* = 3.2 Hz, 1H), 7.16 (d, *J* = 8.0 Hz, 2H), 7.05 (d, *J* = 9.2 Hz, 1H), 6.96 (d, *J* = 8.0 Hz, 2H), 6.90 (d, *J* = 6.8 Hz, 1H), 6.89 (s, 1H), 6.54 (d, *J* = 3.2 Hz, 1H), 3.85 (s, 3H), 3.74 (d, *J* = 16.4 Hz, 1H), 3.48 (d, *J* = 16.4 Hz, 1H), 3.14 (d, *J* = 16.4 Hz, 1H), 2.99 (d, *J* = 16.4 Hz, 1H), 2.72 (s, 3H), 2.33 (s, 3H), 1.49 (s, 9H); ^13^C NMR (125 MHz, CDCl_3_) δ 190.3, 183.7, 170.8, 160.8, 155.0, 147.3, 141.1, 138.3, 134.6, 132.7, 132.0, 129.9, 129.7 (2C), 128.8, 125.9 (2C), 124.7, 114.1, 109.8, 106.8, 86.5, 76.8, 69.8, 55.6, 46.4, 39.2, 28.2, 27.5 (3C), 21.3; HRMS (ESI) calcd for C_32_H_32_N_3_O_7_ [M + H]^+^ 570.2235, found 570.2232.

**5’l**:

m.p. = 166–168 °C; ^1^H NMR (400 MHz, CDCl_3_) δ 7.52–7.49 (m, 1H), 7.33 (d, *J* = 8.8 Hz, 1H), 7.13 (d, *J* = 7.8 Hz, 2H), 6.95 (d, *J* = 8.8 Hz, 1H), 6.90–6.78 (m, 3H), 6.63–6.59 (m, 1H), 3.85 (s, 3H), 3.85 (d, *J* = 16.4 Hz, 1H), 3.54 (d, *J* = 18.4 Hz, 1H), 3.13 (d, *J* = 16.4 Hz, 1H), 2.84 (d, *J* = 18.4 Hz, 1H), 2.61 (s, 3H), 2.33 (s, 3H), 1.60 (s, 9H); ^13^C NMR (125 MHz, CDCl_3_) δ 188.5, 184.3, 171.4, 160.5, 154.1, 146.9, 139.3, 138.2, 135.3, 133.9, 132.4, 130.3, 129.6 (2C), 128.7, 126.8, 125.5 (2C), 114.3, 109.6, 108.0, 87.2, 73.8, 67.9, 55.6, 48.8, 40.0, 27.9 (3C), 27.6, 21.3; HRMS (ESI) calcd for C_32_H_32_N_3_O_7_ [M + H]^+^ 570.2235, found 570.2237.

(±)-*tert*-butyl (1′R*,4R*)-3,5’-dimethyl-2,4″,5,7″-tetraoxo-1-(*p-*tolyl)-4″,7″-dihydro-3′*H*-dispiro[imidazolidine-4,2′-indene-1′,6″-indole]-1″(5″*H*)-carboxylate **5m** and (±)-*tert*-butyl (1′S*,4R*)-3,5’-dimethyl-2,4″,5,7″-tetraoxo-1-(*p-*tolyl)-4″,7″-dihydro-3′*H*-dispiro[imidazolidine-4,2′-indene-1′,6″-indole]-1″(5″*H*)-carboxylate **5’m**.

Compounds **5m** and **5’m** were prepared according to the general procedure. Purification by column chromatography (silica gel, PET/EtOAc = 5:1, *v*/*v*) generated compounds **5m** (white solid, 144 mg, 26%) and **5’m** (white solid, 232 mg, 42%).

**5m**:

m.p. = 161–163 °C; ^1^H NMR (500 MHz, CDCl_3_) δ 7.43 (d, *J* = 3.0 Hz, 1H), 7.18 (d, *J* = 8.0 Hz, 3H), 7.15 (s, 1H), 7.03 (d, *J* = 8.0 Hz, 1H), 6.96 (d, *J* = 8.0 Hz, 2H), 6.55 (d, *J* = 3.0 Hz, 1H), 3.74 (d, *J* = 16.5 Hz, 1H), 3.51 (d, *J* = 16.5 Hz, 1H), 3.14 (d, *J* = 16.5 Hz, 1H), 2.99 (d, *J* = 16.5 Hz, 1H), 2.70 (s, 3H), 2.41 (s, 3H), 2.33 (s, 3H), 1.48 (s, 9H); ^13^C NMR (125 MHz, CDCl_3_) δ 190.3, 183.6, 170.9, 155.1, 147.4, 139.7, 139.5, 138.3, 137.1, 134.6, 132.6, 129.9, 129.7 (2C), 129.1, 128.9, 125.9 (2C), 125.1, 123.5, 106.8, 86.5, 76.7, 70.2, 46.2, 39.1, 28.3, 27.5 (3C), 21.6, 21.3; HRMS (ESI) calcd for C_32_H_32_N_3_O_6_ [M + H]^+^ 554.2286, found 554.2281.

**5’m**:

m.p. = 164–166 °C; ^1^H NMR (400 MHz, CDCl_3_) δ 7.51 (d, *J* = 3.2 Hz, 1H), 7.30 (d, *J* = 8.0 Hz, 1H), 7.22 (d, *J* = 8.0 Hz, 1H), 7.17 (s, 1H), 7.13 (d, *J* = 8.0 Hz, 2H), 6.83 (d, *J* = 8.0 Hz, 2H), 6.62 (d, *J* = 3.2 Hz, 1H), 3.84 (d, *J* = 16.8 Hz, 1H), 3.54 (d, *J* = 18.4 Hz, 1H), 3.14 (d, *J* = 16.8 Hz, 1H), 2.85 (d, *J* = 18.4 Hz, 1H), 2.59 (s, 3H), 2.41 (s, 3H), 2.33 (s, 3H), 1.60 (s, 9H); ^13^C NMR (125 MHz, CDCl_3_) δ 188.4, 184.1, 171.6, 154.1, 146.9, 139.2, 138.1, 137.9, 137.5, 135.3, 133.8, 130.3, 129.6 (2C), 129.2, 128.7, 125.7, 125.5 (2C), 125.1, 108.0, 87.3, 73.7, 68.4, 48.6, 39.8, 27.9 (3C), 27.6, 21.5, 21.2; HRMS (ESI) calcd for C_32_H_32_N_3_O_6_ [M + H]^+^ 554.2286, found 554.2286.

(±)-*tert*-butyl (1′R*,4R*)-3,6’-dimethyl-2,4″,5,7″-tetraoxo-1-(*p-*tolyl)-4″,7″-dihydro-3′*H*-dispiro[imidazolidine-4,2′-indene-1′,6″-indole]-1″(5″*H*)-carboxylate **5n** and (±)-*tert*-butyl (1′S*,4R*)-3,6’-dimethyl-2,4″,5,7″-tetraoxo-1-(*p-*tolyl)-4″,7″-dihydro-3′*H*-dispiro[imidazolidine-4,2′-indene-1′,6″-indole]-1″(5″*H*)-carboxylate **5’n**.

Compounds **5n** and **5’n** were prepared according to the general procedure. Purification by column chromatography (silica gel, PET/EtOAc = 5:1, *v*/*v*) generated compounds **5n** (white solid, 127 mg, 23%) and **5’n** (white solid, 244 mg, 44%).

**5n**:

m.p. = 162–164 °C; ^1^H NMR (400 MHz, CDCl_3_) δ 7.43 (d, *J* = 3.2 Hz, 1H), 7.25–7.13 (m, 4H), 7.01–6.89 (m, 3H), 6.54 (d, *J* = 3.2 Hz, 1H), 3.74 (d, *J* = 16.4 Hz, 1H), 3.53 (d, *J* = 16.4 Hz, 1H), 3.13 (d, *J* = 16.4 Hz, 1H), 2.99 (d, *J* = 16.4 Hz, 1H), 2.68 (s, 3H), 2.39 (s, 3H), 2.33 (s, 3H), 1.50 (s, 9H); ^13^C NMR (125 MHz, CDCl_3_) δ 190.3, 183.4, 170.8, 155.0, 147.3, 140.1, 138.3, 138.0, 136.7, 134.6, 132.6, 130.4, 130.0, 129.7 (2C), 128.9, 125.9 (2C), 124.4, 124.1, 106.8, 86.5, 77.4, 77.2, 76.9, 70.2, 46.0, 38.9, 28.2, 27.5 (4C), 21.7, 21.3; HRMS (ESI) calcd for C_32_H_32_N_3_O_6_ [M + H]^+^ 554.2286, found 554.2288.

**5’n**:

m.p. = 165–167 °C; ^1^H NMR (500 MHz, CDCl_3_) δ 7.51 (d, *J* = 3.2 Hz, 1H), 7.25–7.17 (m, 3H), 7.13 (d, *J* = 8.0 Hz, 2H), 6.83 (d, *J* = 8.4 Hz, 2H), 6.62 (d, *J* = 3.2 Hz, 1H), 3.83 (d, *J* = 16.8 Hz, 1H), 3.54 (d, *J* = 18.4 Hz, 1H), 3.14 (d, *J* = 16.8 Hz, 1H), 2.87 (d, *J* = 18.4 Hz, 1H), 2.59 (s, 3H), 2.42 (s, 3H), 2.32 (s, 3H), 1.61 (s, 9H); ^13^C NMR (125 MHz, CDCl_3_) δ 188.3, 184.1, 171.6, 154.1, 146.9, 140.5, 138.1, 138.1, 135.4, 134.8, 133.8, 130.3, 130.1, 129.6 (2C), 128.7, 126.6, 125.5 (2C), 124.2, 107.9, 87.3, 73.8, 68.5, 48.6, 39.6, 27.9 (3C), 27.6, 21.7, 21.3; HRMS (ESI) calcd for C_32_H_32_N_3_O_6_ [M + H]^+^ 554.2286, found 554.2285.

48.6, 39.6, 27.9 (3C), 27.6, 21.7, 21.3; HRMS (ESI) calcd for C_32_H_32_N_3_O_6_ [M + H]^+^ 554.2286, found 554.2283.

### 3.3. Scale-Up Synthesis of 2f and 2′f

To a solution of 5-(2-(1,4-dioxo-1,4-dihydronaphthalen-2-yl)benzyl)-1-methyl-3-(4-(trifluoromethyl)phenyl)imidazolidine-2,4-dione **1f** (3.02 g, 6.0 mmol) in CH_2_Cl_2_ (50 mL) at −20 °C under argon, a solution of NaOH (48 mg, 1.2 mmol) in CH_3_OH (10 mL) was added dropwise over 5 min. The reaction mixture was stirred for 1 h. Then, the reaction was quenched with 1N HCl (50 mL), extracted with CH_2_Cl_2_ (3 × 50 mL), washed with brine (100 mL), dried over anhydrous Na_2_SO_4_, and concentrated under reduced pressure. The residue was purified by column chromatography (silica gel, PET/EtOAc = 5:1), yielding compound **2f** (white solid, 0.97 g, 32%) and compound **2′f** (white solid, 1.87 g, 62%).

### 3.4. Cytotoxicity

MTT assay was used to detect the antiproliferative effects of the compounds in vitro. U251 cells were seeded in 96-well plates at 1 × 10^4^ per well. The cells were treated with different concentrations (10, 1, and 0.1 μM) of the compounds and Epacadostat (positive control) and incubated at 37 °C with 5% CO_2_ for 48 h. MTT solution with a final concentration of 0.5 mg/mL was added to each well, and incubated at 37 °C for 3 h in dark conditions. DMSO was added to each well, and the OD value was measured at 570 nm. The inhibition of compounds on cell proliferation was calculated as follows: cell proliferation inhibition (%) = (OD control-OD compound)/(OD control-OD blank) × 100%. The IC_50_ value was calculated by GraphPad Prism 8.0 software.

## 4. Conclusions

In conclusion, a classic intramolecular Michael addition with high regioselectivity was employed in our work, which enabled facile transformation of naphthoquinone or indolequinone to a biologically interesting dispirocyclic skeleton. This method was mild, efficient, operationally simple, scalable, and transition-metal free. A wide variety of functional groups were well tolerated in this reaction, and the corresponding dispirocyclic products were obtained in good to excellent yields. A gram-scale experiment exemplified the practicality of this protocol. In addition, some compounds exhibited potent cytotoxicity in vitro. Further exploration of the potential of dispirocyclic compounds in the development of novel anti-cancer agents is ongoing in our laboratory, and the results will be reported in due course.

## Figures and Tables

**Table 1:** Optimization of the reaction conditions *^a^*.

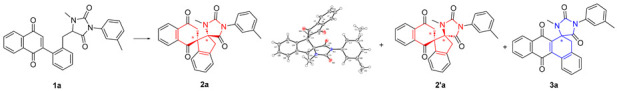								
Entry	Base	Equiv.	Solvent	Temp. (°C)	t (h)	Yield *^b^* (%)		
						2a	2′a	3a *^c^*
1	LiHMDS	1.0	THF	−78	1	18	—	25
2	NaHMDS	1.0	THF	−78	1	Trace	—	21
3	KHMDS	1.0	THF	−78	1	—	—	18
4	*n*BuLi *^d^*	1.0	THF	−78	1	—	—	—
5	DBU *^e^*	1.0	THF	rt	3	—	—	—
6	NaH *^e^*	1.0	THF	rt	3	—	—	—
7	NaOH	1.0	CH_3_OH	rt	3	18	33	14
8	KOH	1.0	CH_3_OH	rt	3	16	34	11
9	LiOH	1.0	CH_3_OH	rt	3	10	25	12
10	CH_3_ONa	1.0	CH_3_OH	rt	3	15	11	26
11	*t*BuOK *^d^*	1.0	*t*BuOH	rt	3	—	—	—
12	Cs_2_CO_3_	1.0	CH_3_OH	rt	3	12	24	14
13	K_2_CO_3_	1.0	CH_3_OH	rt	3	13	22	13
14	Na_2_CO_3_	1.0	CH_3_OH	rt	3	8	12	Trace
15	NaOH	1.0	CH_2_Cl_2_	rt	3	12	23	10
16	NaOH	1.0	THF	rt	3	15	21	12
17	NaOH	1.0	CH_3_OH	50	1	Trace	—	45
18	NaOH	1.0	CH_2_Cl_2_/CH_3_OH (5:1, *v*/*v*)	rt	1	22	43	17
19	NaOH	1.0	CH_2_Cl_2_/CH_3_OH (5:1, *v*/*v*)	0	1	36	53	5
20	NaOH	1.0	CH_2_Cl_2_/CH_3_OH (5:1, *v*/*v*)	−20	1	35	58	—
21	NaOH	0.2	CH_2_Cl_2_/CH_3_OH (5:1, *v*/*v*)	−20	1	37	55	—

*^a^* Reaction conditions: compound **1a** (0.2 mmol) and base in solvent (5.0 mL) were stirred under argon. *^b^* Determined after column chromatography. *^c^* Total yield of compound **3a** and inseparable impurities. *^d^* Compound **1a** was decomposed completely. *^e^* Starting material **1a** was recycled completely.

**Table 2:** Cytotoxicity of some dispirocyclic compounds against different cancer cell lines.

Compound	IC_50_ (μmol/L)				
	MCF-7	HepG2	HCT-116	HGC27	U251
2a	>10	>10	>10	>10	>10
2′a	2.861	4.335	3.433	9.252	>10
2c	>10	>10	>10	>10	>10
2′c	3.174	3.486	3.460	9.561	>10
2d	>10	>10	>10	>10	>10
2′d	2.816	2.316	5.001	8.746	>10
2e	>10	>10	>10	>10	>10
2′e	3.486	3.252	3.722	6.895	9.953
2f	3.691	5.774	2.948	>10	>10
2′f	3.819	3.231	3.950	6.971	>10
2g	>10	>10	>10	>10	>10
2′g	3.081	3.465	3.781	7.861	>10
2m	>10	>10	>10	>10	>10
2′m	2.682	1.730	2.345	5.225	8.982
2n	>10	>10	>10	>10	>10
2′n	4.343	2.901	3.708	9.271	>10
2o	>10	>10	>10	>10	>10
2′o	2.250	3.296	2.377	9.779	>10
2p	2.015	2.247	2.278	>10	>10
2′p	2.460	1.632	2.398	8.036	9.627
2q	>10	>10	>10	>10	>10
2′q	1.915	2.503	3.109	8.871	9.541
2r	4.230	2.353	3.233	9.911	>10
2′r	2.148	3.733	3.400	9.238	>10
5’b	3.732	5.307	4.220	>10	>10
5’d	1.918	2.647	1.528	9.038	4.093

## Data Availability

The data supporting this article have been included as part of the Supplementary Materials. Crystallographic data for **2a** and **2′b** were deposited at the Cambridge Crystallographic Data Centre under CCDC 2452353 and CCDC 2372028 and can be obtained from https://www.ccdc.cam.ac.uk/ (CCDC 2452353 accessed on 19 May 2025, CCDC 2372028 accessed on 19 July 2024).

## Supplementary Materials

The following supporting information can be downloaded at https://www.mdpi.com/article/10.3390/molecules30153164/s1: Table S1: Cytotoxicity of dispirocyclic compounds; Schemes S1–S3: Synthesis of compound **1** and compound **4**; Figures S1–S140: ^1^H, ^13^C, and ^19^F{H} NMR spectra of compounds **2a**-**5’n**; Figures S141–S144: ^1^H, ^13^C spectra of compounds **3m** and **3n**; Figures S145 and S146: Single-crystal X-Ray diffraction analysis of **2a** and **2′b**.
