# Supplementary material for: Straightforward Access to the Dispirocyclic Framework via Regioselective Intramolecular Michael Addition
Source: Molecules. 2025 Jul 29;30(15):3164. doi: 10.3390/molecules30153164 (PMC12348457; doi:10.3390/molecules30153164)

Article

Straightforward Access to the Dispirocyclic Framework via Regioselective Intramolecular Michael Addition

Weilun Cao, Junmin Dong, Xuan Pan * and Zhanzhu Liu *

State Key Laboratory of Bioactive Substances and Functions of Natural Medicines, Institute of Materia Medica, Peking Union Medical College and Chinese Academy of Medical Sciences, Beijing 100050, China

* Correspondence: panxuan@imm.ac.cn (X.P.); liuzhanzhu@imm.ac.cn (Z.L.)

Abstract

In this article, an efficient and straightforward protocol for the construction of complex dispirocyclic skeletons via regioselective intramolecular Michael addition is presented. Diverse dispirocyclic compounds were synthesized under mild and transition-metal-free conditions with good to excellent yields. Most stereoisomers were conveniently separated by column chromatography, and their relative configurations were identified by single-crystal X-Ray diffraction of representative compounds. A scale-up experiment validated the practicality of this method. In an in vitro assay, some dispirocyclic compounds exhibited potent cytotoxicity with an IC_{50} value of 10^{-6} mol/L.

Keywords: spiro compounds; regioselectivity; transition-metal free

---

Contents

Table S1 Cytotoxicity of dispirocyclic compounds

Scheme S1-S3 General synthetic route for compounds **1** and **4**

Figure S1-S140 ^{1}H, ^{13}C and $^{19}F\{H\}$ NMR spectra of compounds **2a-5'n**.

Figure S141-S144 ^{1}H, ^{13}C spectra of compounds **3m** and **3n**.

Figure S145-S146 Single-crystal X-ray diffraction analysis of **2a** and **2'b**

Table S1. Cytotoxicity of dispirocyclic compounds against different cancer cell lines.

Compound	IC50 (μmol/L)				
	MCF-7	HepG2	HCT-116	HGC27	U251
2a	>10	>10	10	>10	>10
2'a	2.861	4.335	3.433	9.252	>10
2b	4.654	4.473	6.476	>10	>10
2'b	6.738	0.294	1.683	9.154	9.577
2c	>10	>10	>10	>10	>10
2'c	3.174	3.486	3.460	9.561	>10
2d	>10	>10	>10	>10	>10
2'd	2.816	2.316	5.001	8.746	>10
2e	>10	>10	>10	>10	>10
2'e	3.486	3.252	3.722	6.895	9.953
2f	3.691	5.774	2.948	>10	>10
2'f	3.819	3.231	3.950	6.971	>10
2g	>10	>10	>10	>10	>10
2'g	3.081	3.465	3.781	7.861	>10
2h	>10	>10	>10	>10	>10
2'h	>10	>10	>10	>10	>10
2i	>10	>10	>10	>10	>10
2'i	>10	>10	>10	>10	>10
2j	>10	>10	>10	>10	>10
2'j	>10	>10	>10	>10	>10
2k	>10	>10	>10	>10	>10
2'k	>10	>10	>10	>10	>10
2l and 2'l	>10	>10	>10	>10	>10
2m	>10	>10	>10	>10	>10
2'm	2.682	1.730	2.345	5.225	8.982
2n	>10	>10	>10	>10	>10
2'n	4.343	2.901	3.708	9.271	>10
2o	>10	>10	>10	>10	>10
2'o	2.250	3.296	2.377	9.779	>10
2p	2.015	2.247	2.278	>10	>10
2'p	2.460	1.632	2.398	8.036	9.627
2q	>10	>10	>10	>10	>10
2'q	1.915	2.503	3.109	8.871	9.541
2r	4.230	2.353	3.233	9.911	>10
2'r	2.148	3.733	3.400	9.238	>10

5a	>10	>10	>10	>10	7.255
5'a	>10	>10	>10	>10	>10
5b	>10	>10	>10	>10	>10
5'b	3.732	5.307	4.220	>10	>10
5c	>10	>10	>10	>10	>10
5'c	>10	>10	>10	>10	>10
5d	>10	>10	>10	>10	>10
5'd	1.918	2.647	1.528	9.038	4.093
5e and 5'e	>10	>10	>10	>10	>10
5f and 5'f	>10	>10	>10	>10	6.672
5g	>10	>10	>10	>10	>10
5'g	>10	>10	>10	>10	>10
5h	>10	>10	>10	>10	>10
5'h	>10	>10	>10	>10	>10
5i	>10	>10	>10	>10	7.255
5'i	>10	>10	>10	>10	>10
5j	>10	>10	>10	>10	>10
5'j	>10	>10	>10	>10	2.130
5k	>10	>10	>10	>10	>10
5'k	>10	>10	>10	>10	>10
5l	>10	>10	>10	>10	>10
5'l	>10	>10	>10	>10	>10
5m	>10	>10	>10	>10	>10
5'm	>10	>10	>10	>10	>10
5n	>10	>10	>10	>10	>10
5'n	>10	>10	>10	>10	>10

The synthesis of compounds **4** was performed following the procedure reported in ref. 17, and the detailed synthetic route was as described in Scheme S1 and S2.

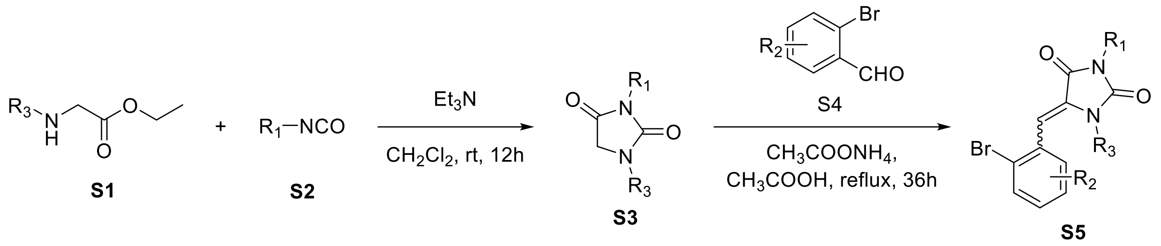

Scheme S1: Synthesis of compounds **S5**

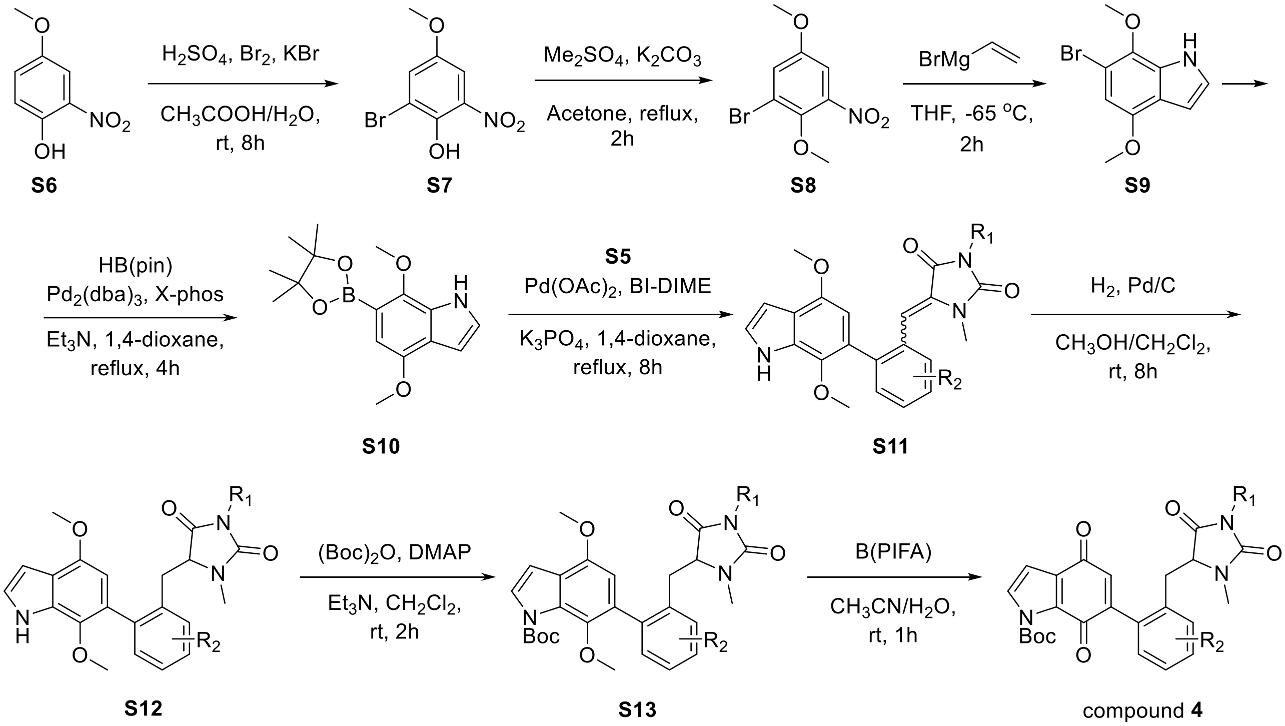

Scheme S2: Synthesis of compounds **4**

Synthesis of compounds **1** was carried out according to the procedure for compounds **4**, which was shown in Scheme 3.

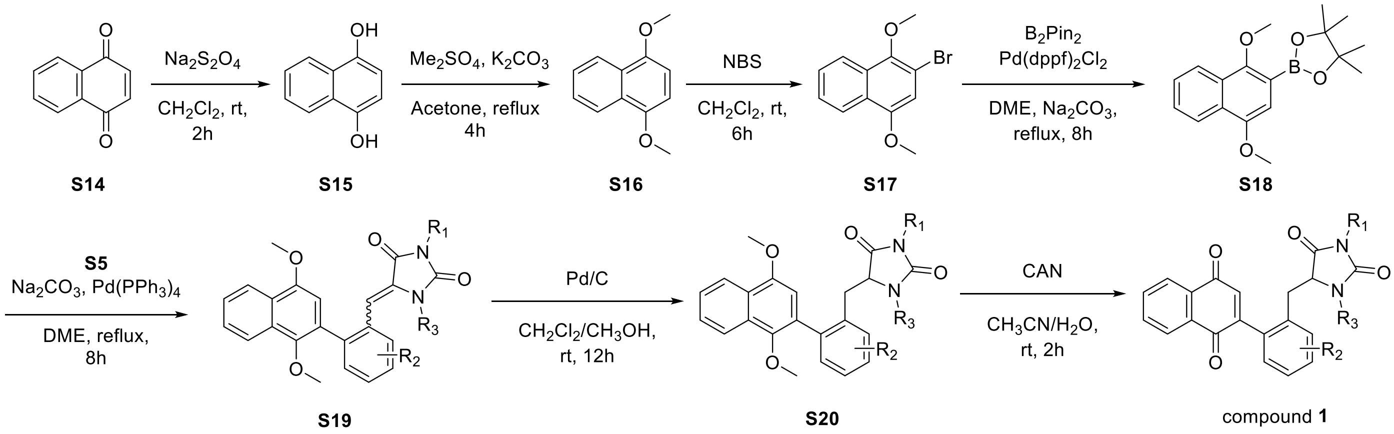

Scheme S3: Synthesis of compounds **1**

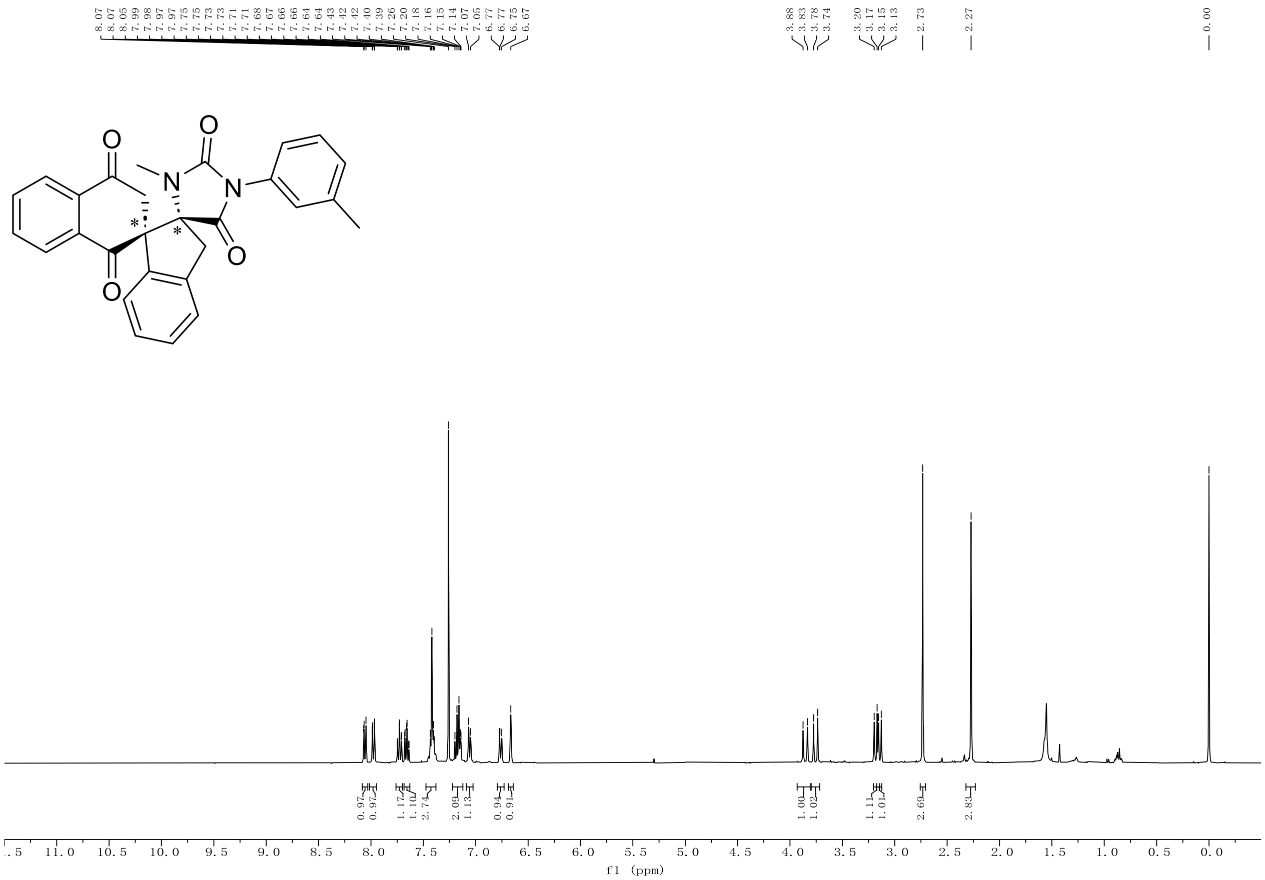

Figure S1: ^{1}H NMR spectra of compound **2a**

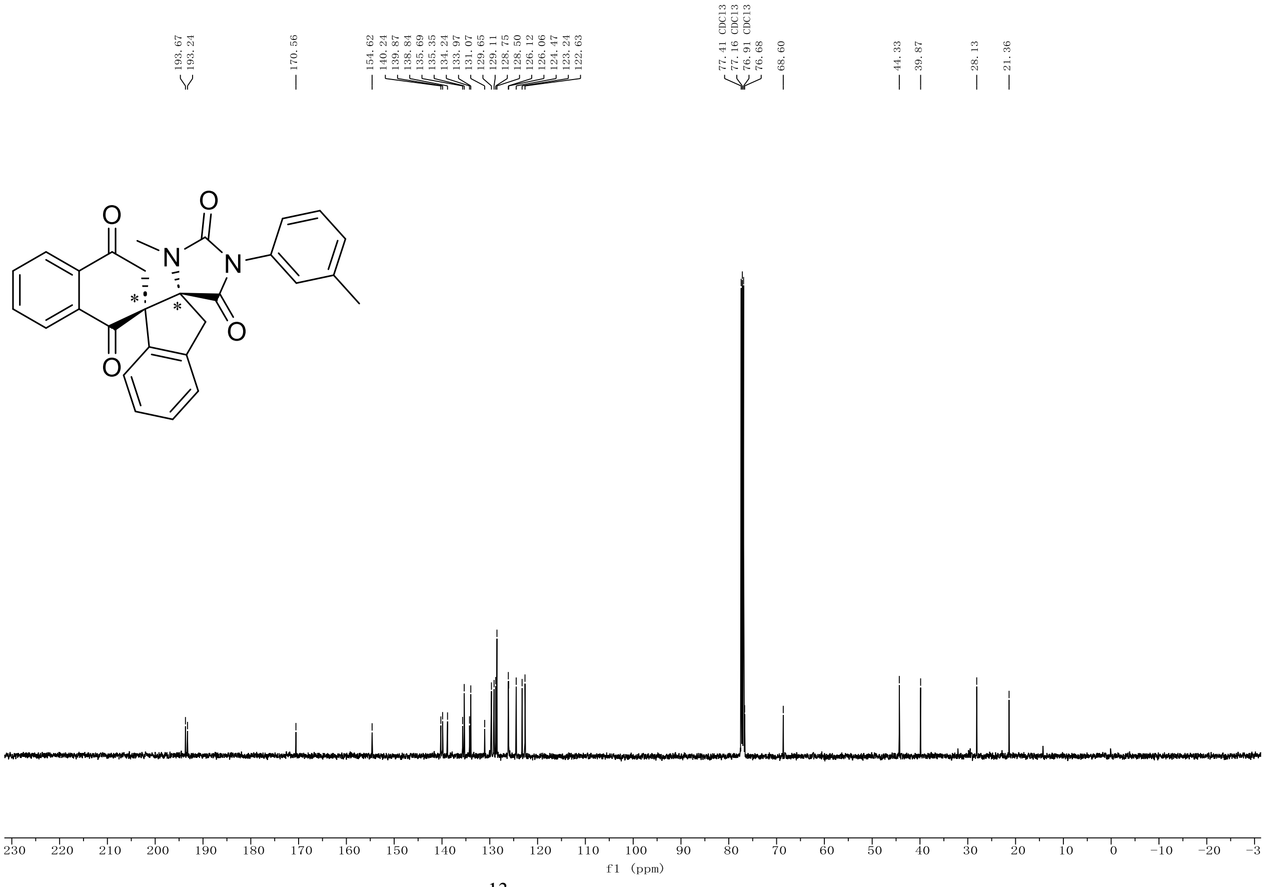

Figure S2: ^{13}C NMR spectra of compound **2a**

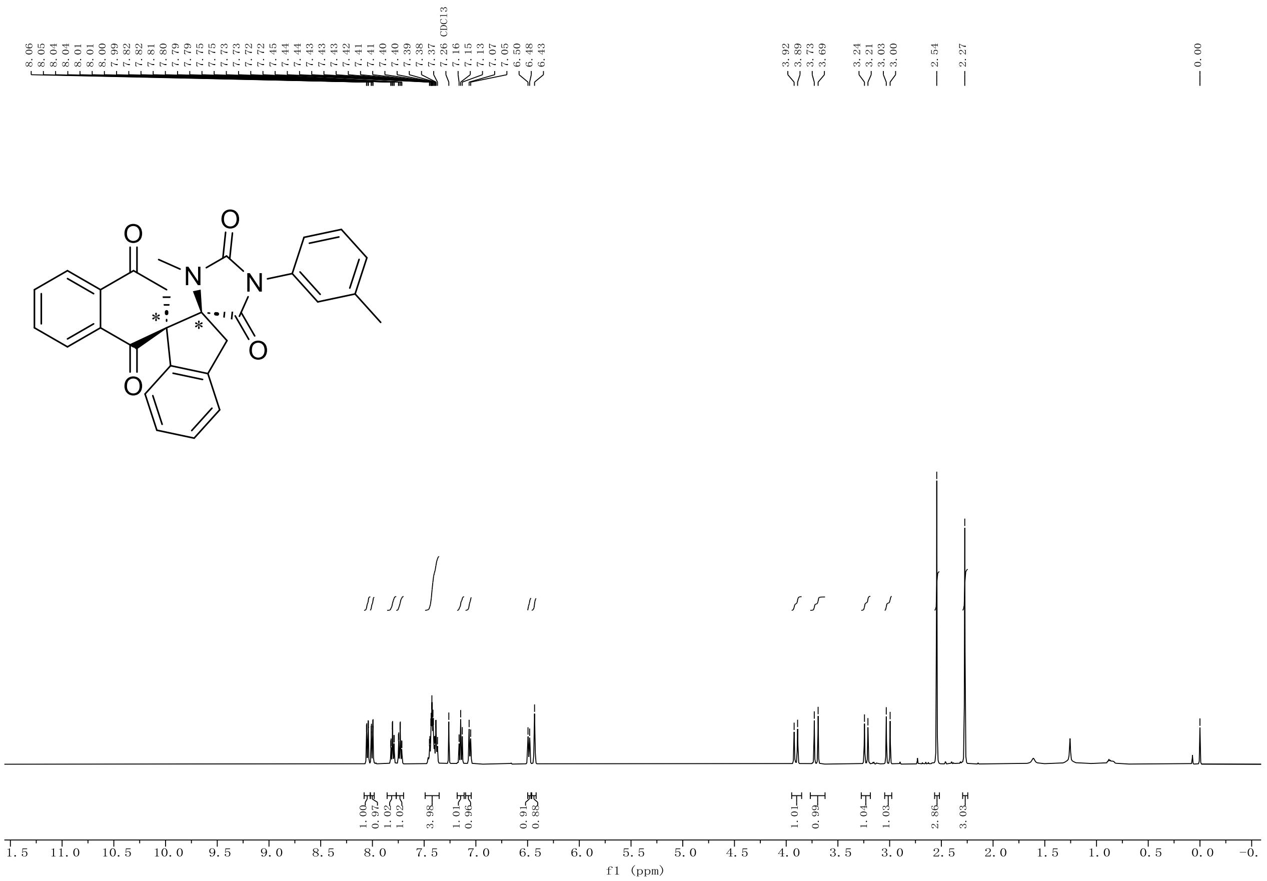

Figure S3: ^{1}H NMR spectra of compound **2'a**

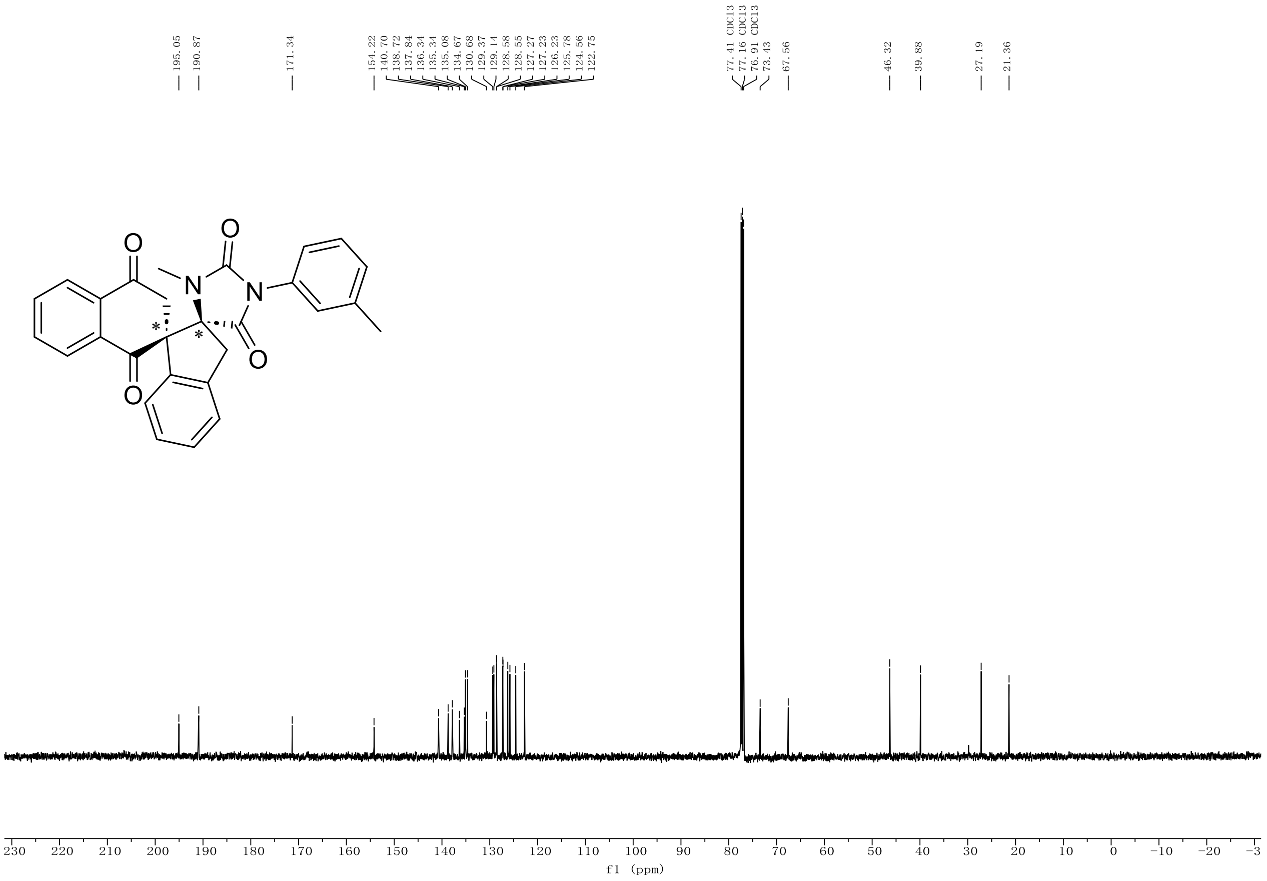

Figure S4: ^{13}C NMR spectra of compound **2'a**

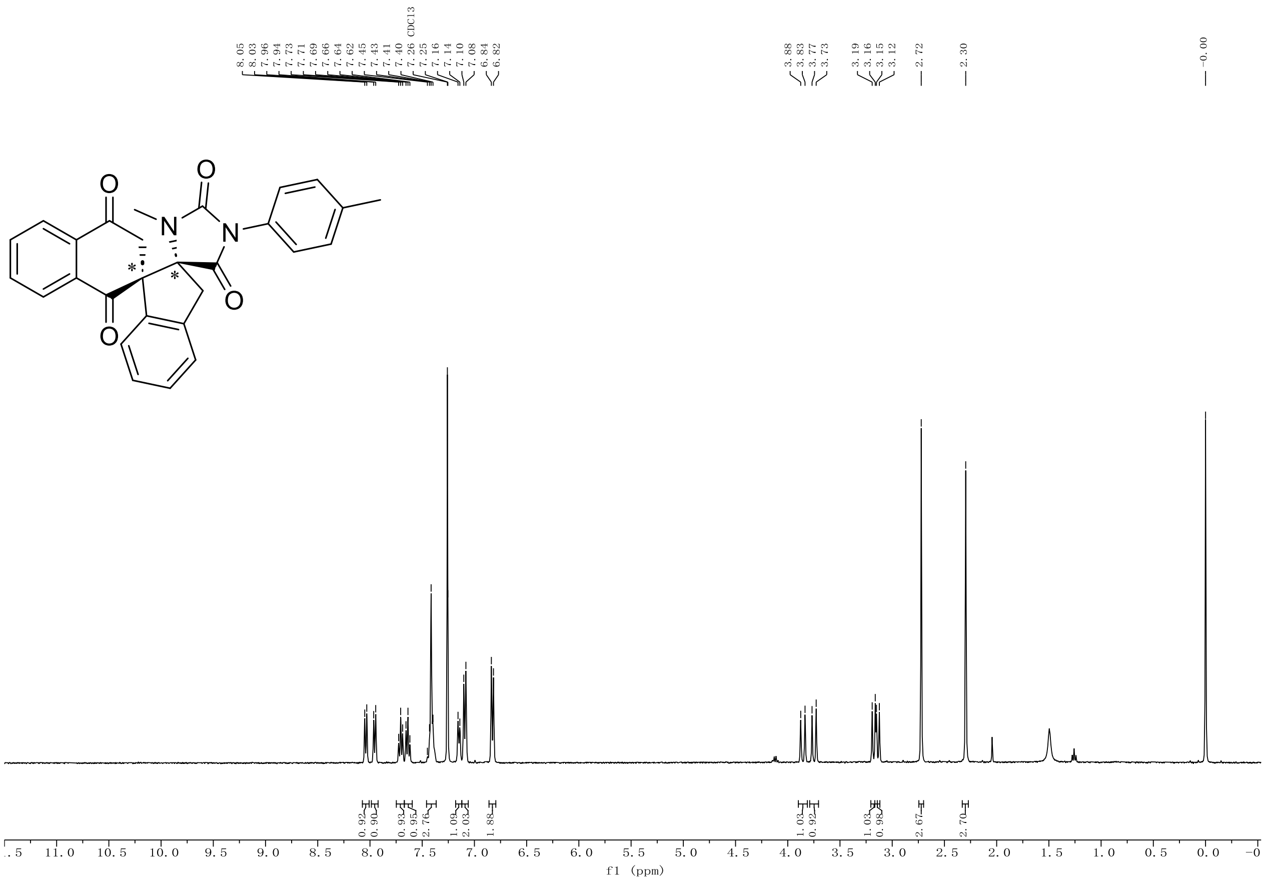

Figure S5: ^{1}H NMR spectra of compound **2b**

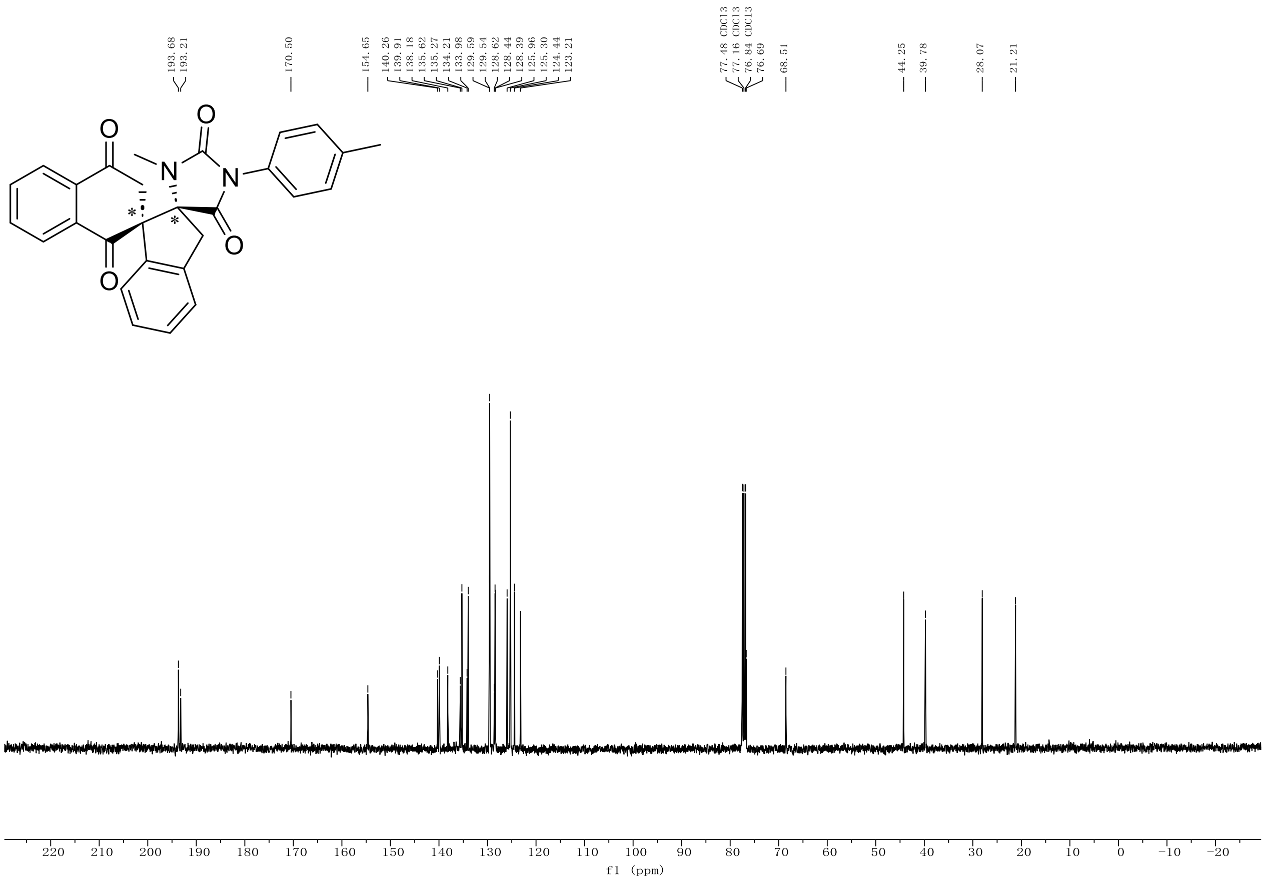

Figure S6: ^{13}C NMR spectra of compound **2b**

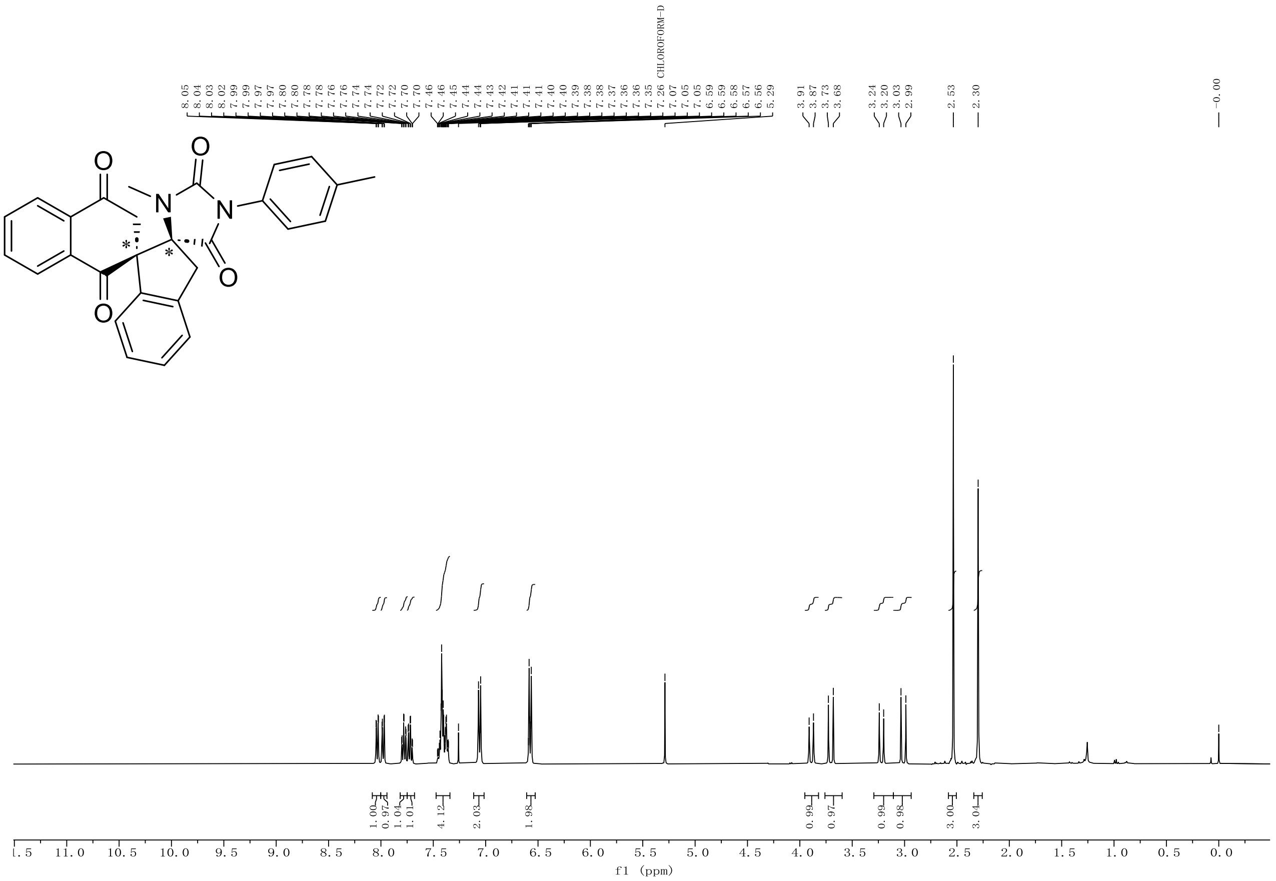

Figure S7: ^{1}H NMR spectra of compound **2'b**

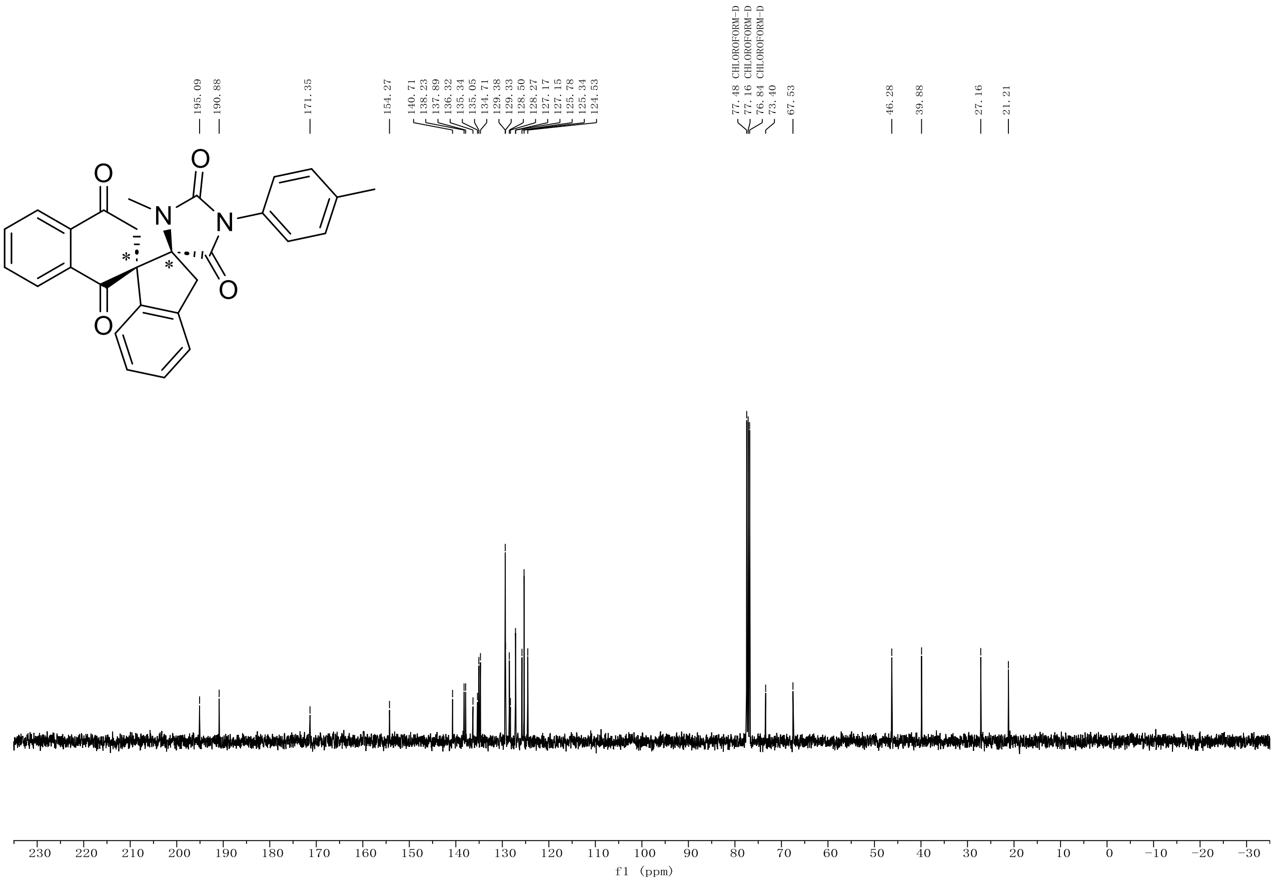

Figure S8: ^{13}C NMR spectra of compound **2'b**

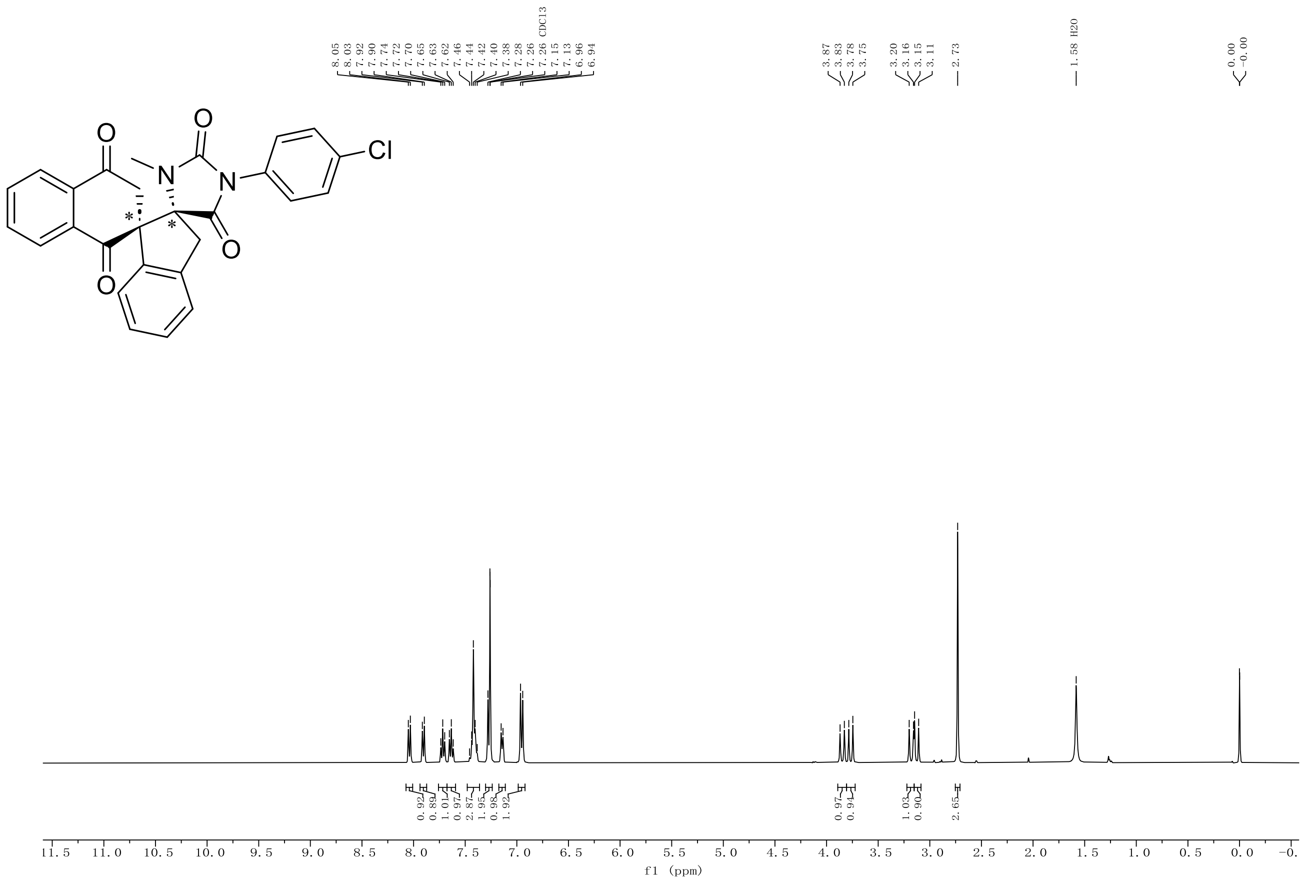

Figure S9: ^{1}H NMR spectra of compound **2c**

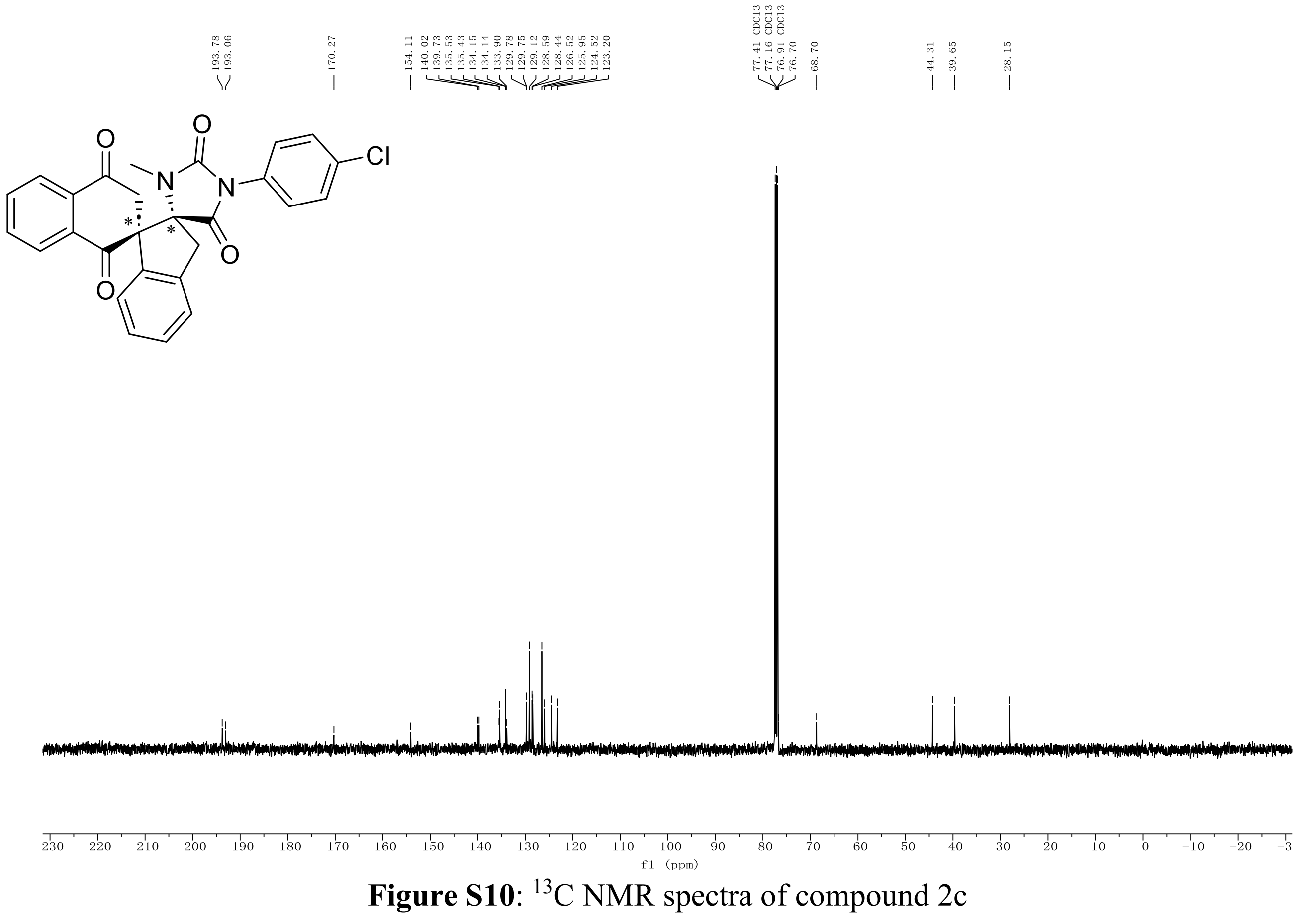

Figure S10: ^{13}C NMR spectra of compound 2c

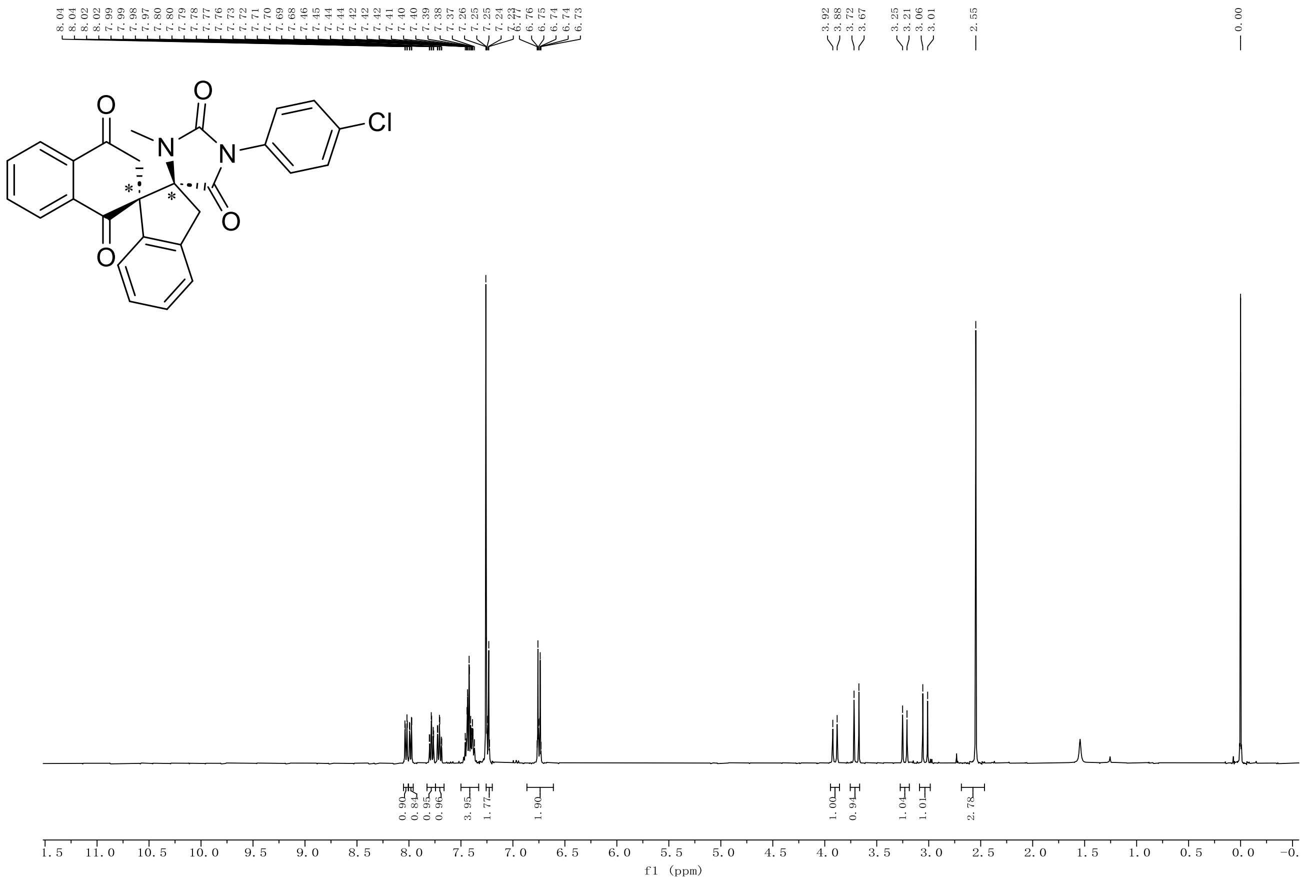

Figure S11: ^{1}H NMR spectra of compound **2'c**

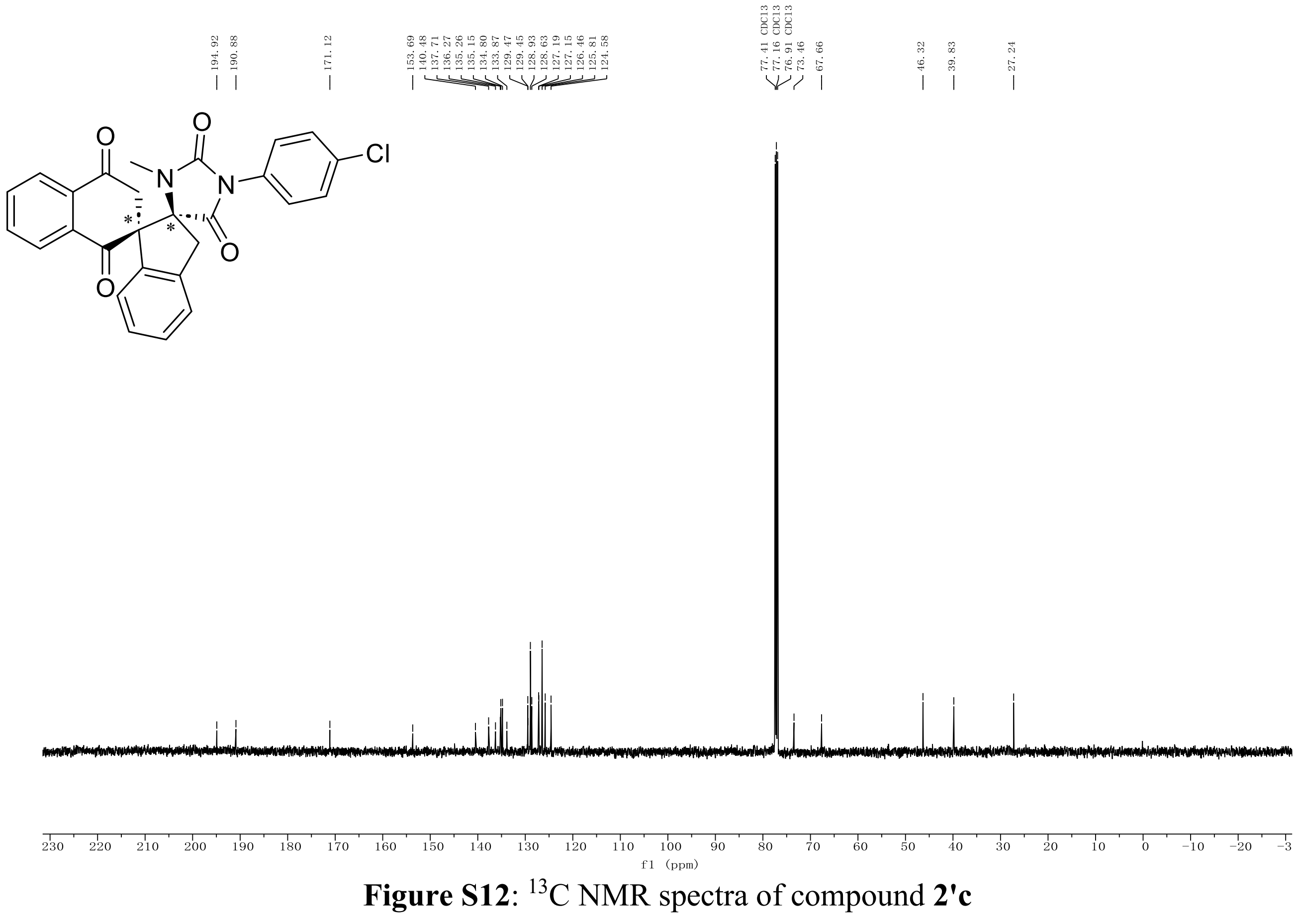

Figure S12: ^{13}C NMR spectra of compound **2'c**

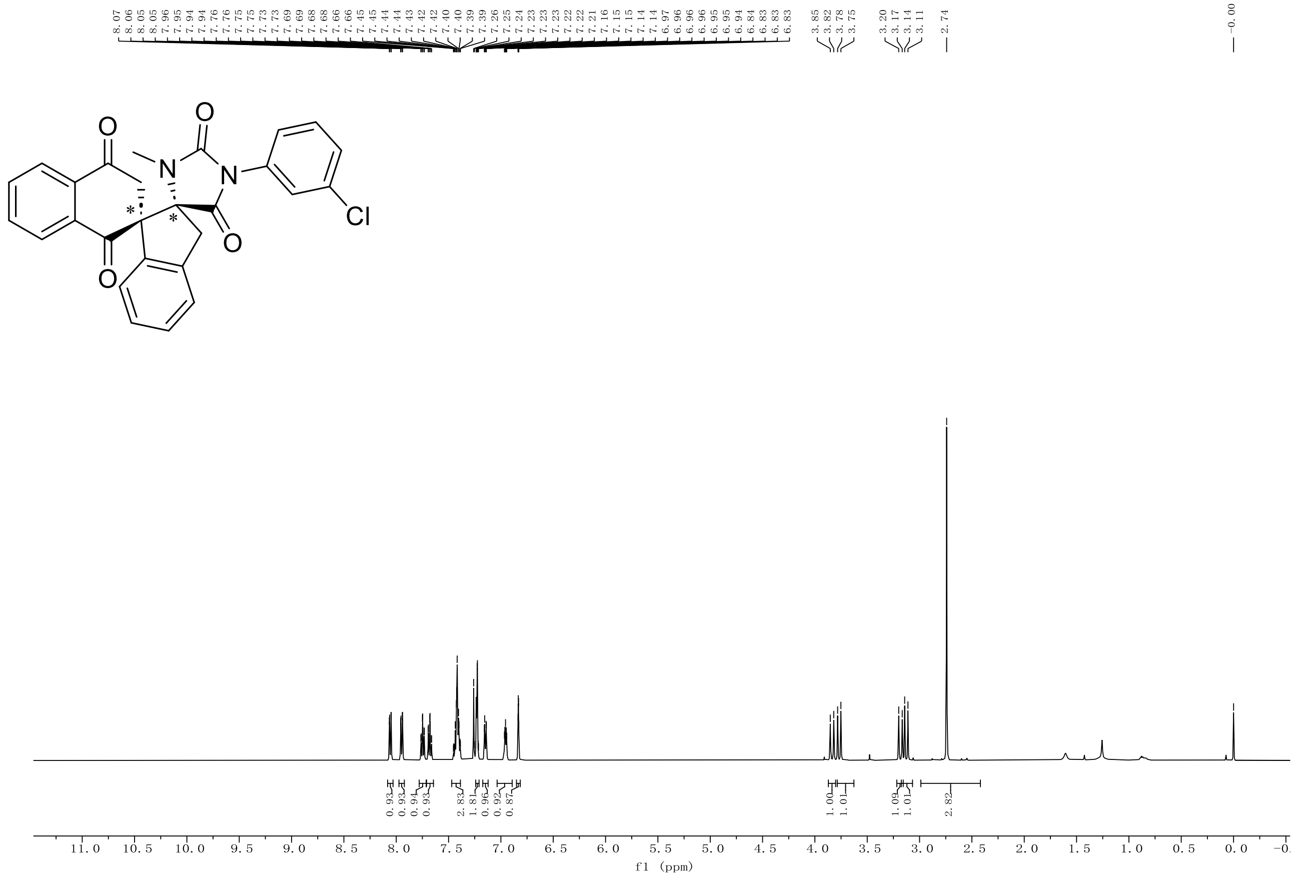

Figure S13: ^{1}H NMR spectra of compound **2d**

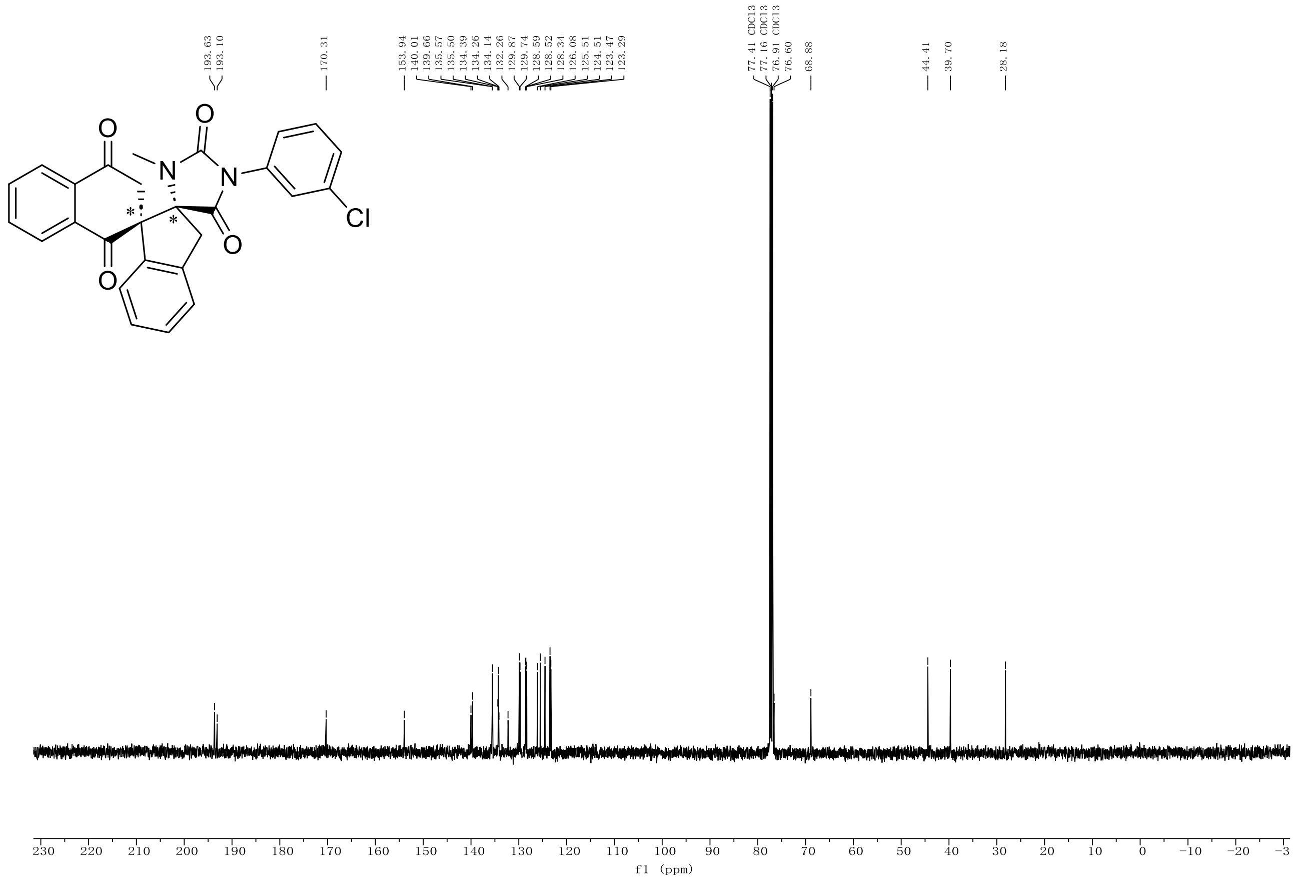

Figure S14: ^{13}C NMR spectra of compound **2d**

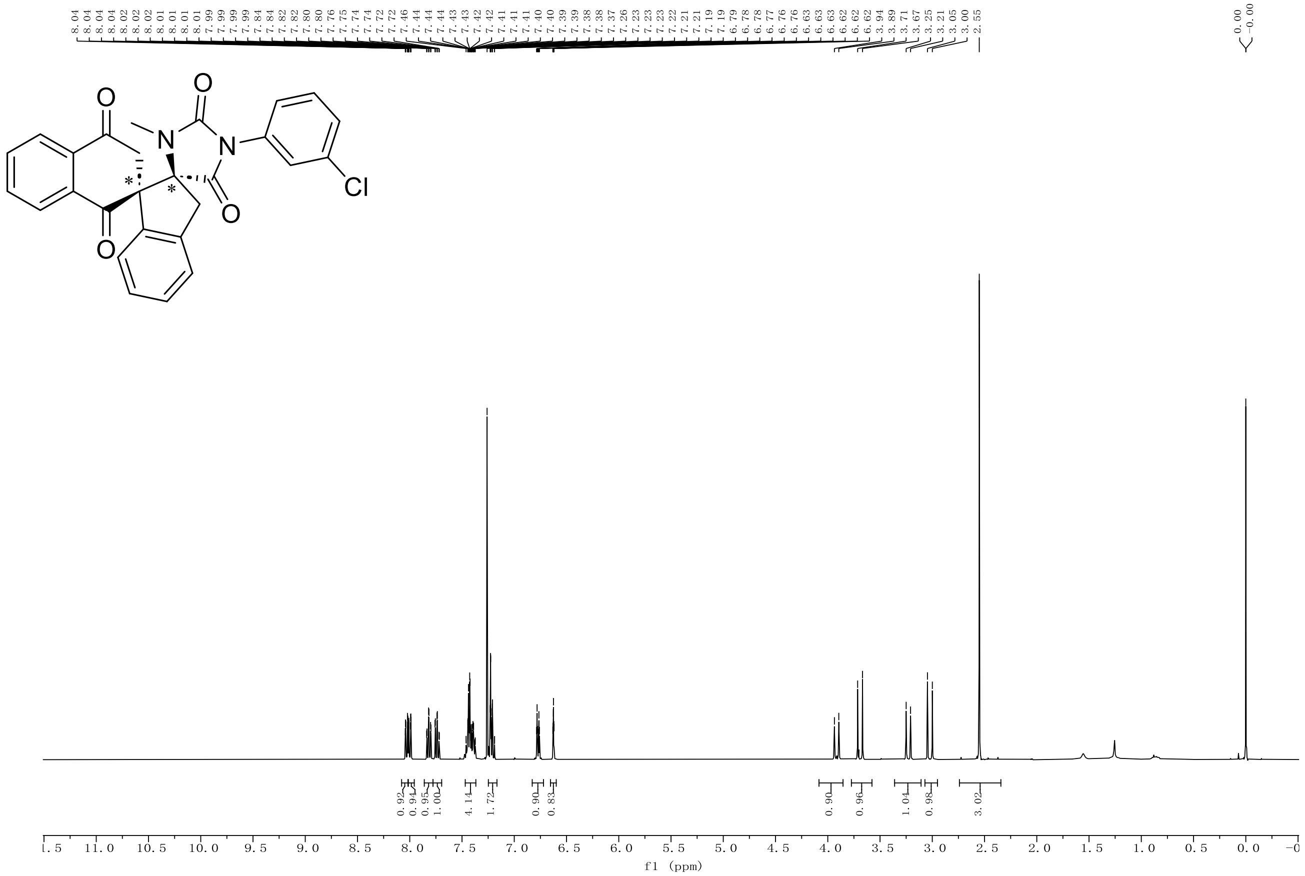

Figure S15: ^{1}H NMR spectra of compound **2'd**

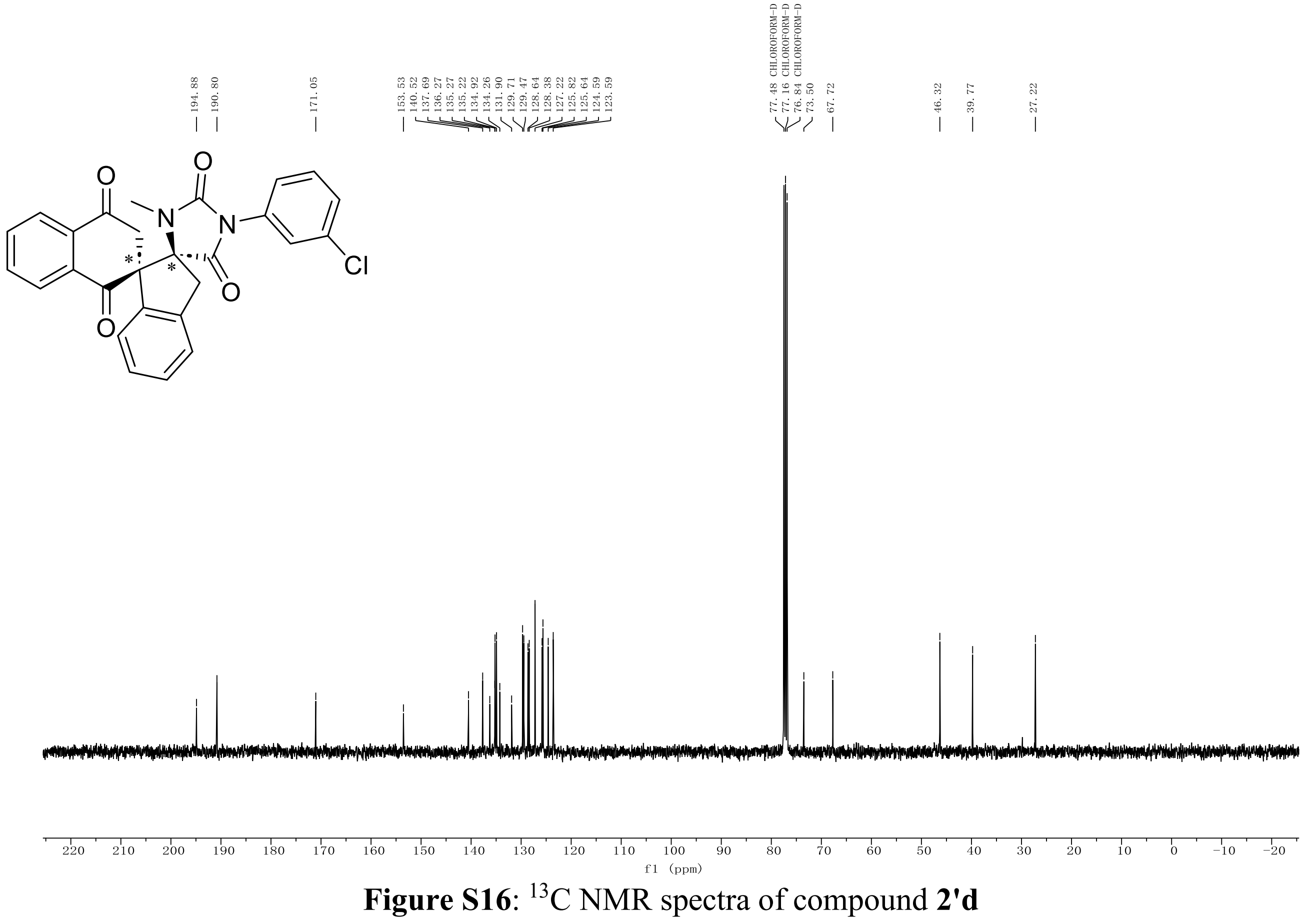

Figure S16: ^{13}C NMR spectra of compound **2'd**

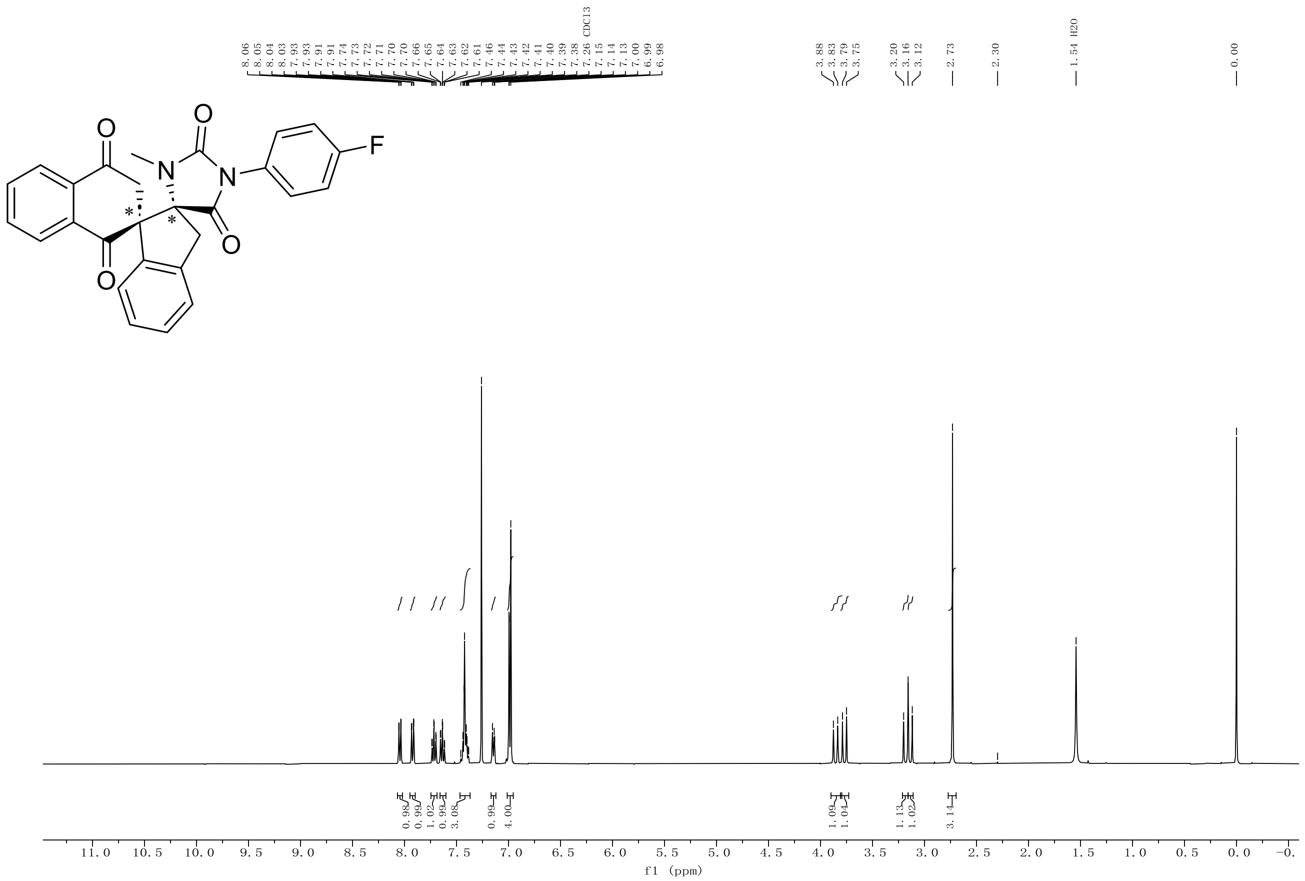

Figure S17: ^{1}H NMR spectra of compound **2e**

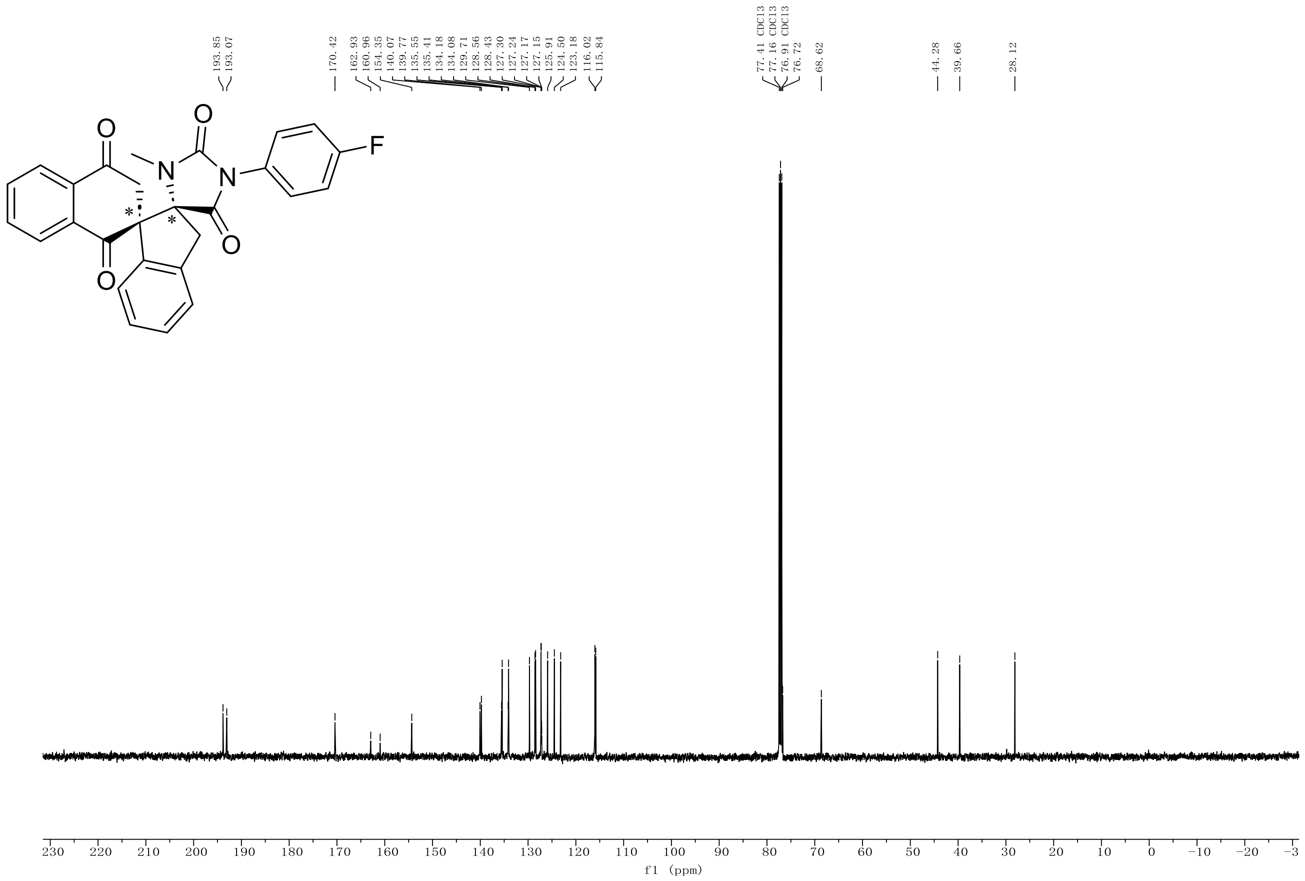

Figure S18: ^{13}C NMR spectra of compound **2e**

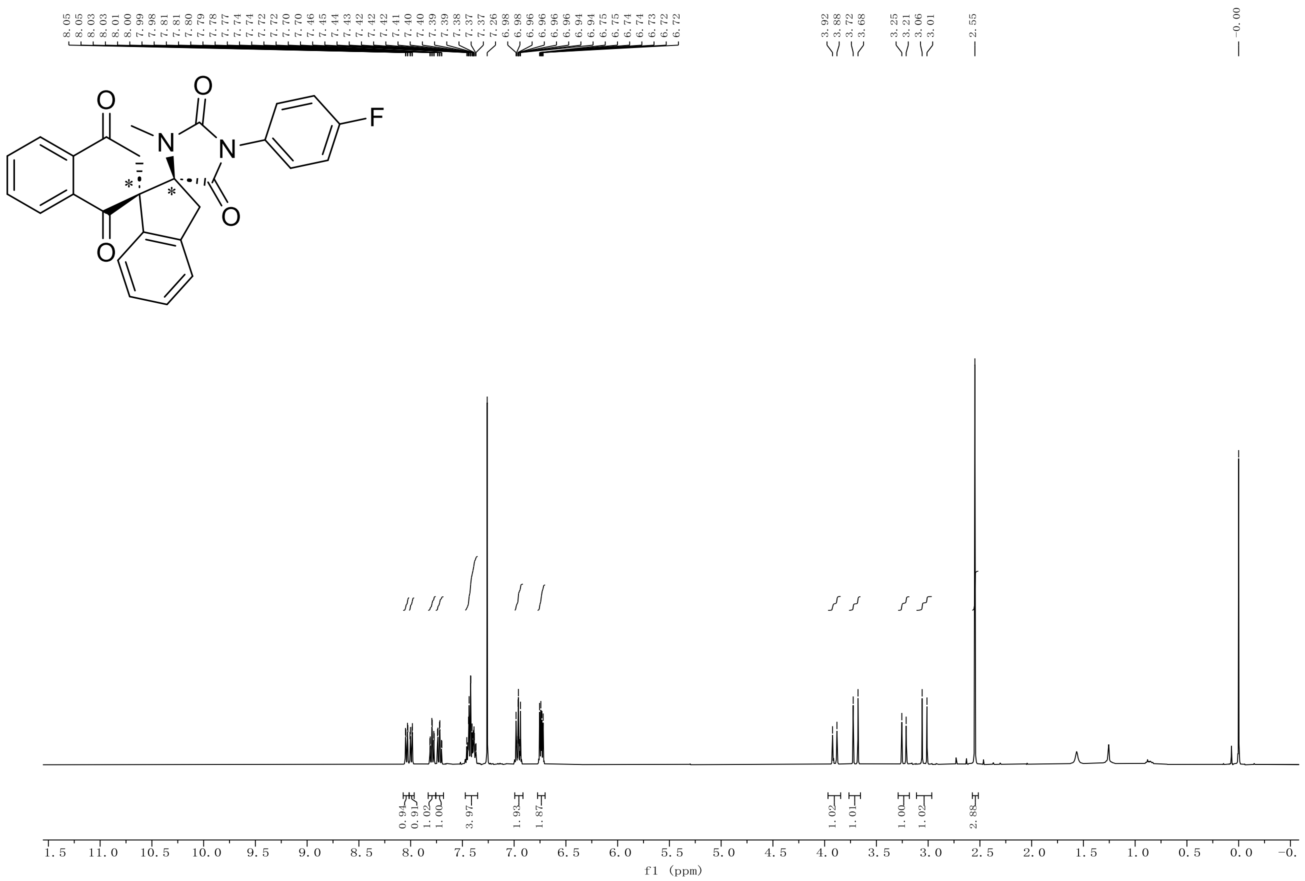

Figure S19: ^{1}H NMR spectra of compound **2'e**

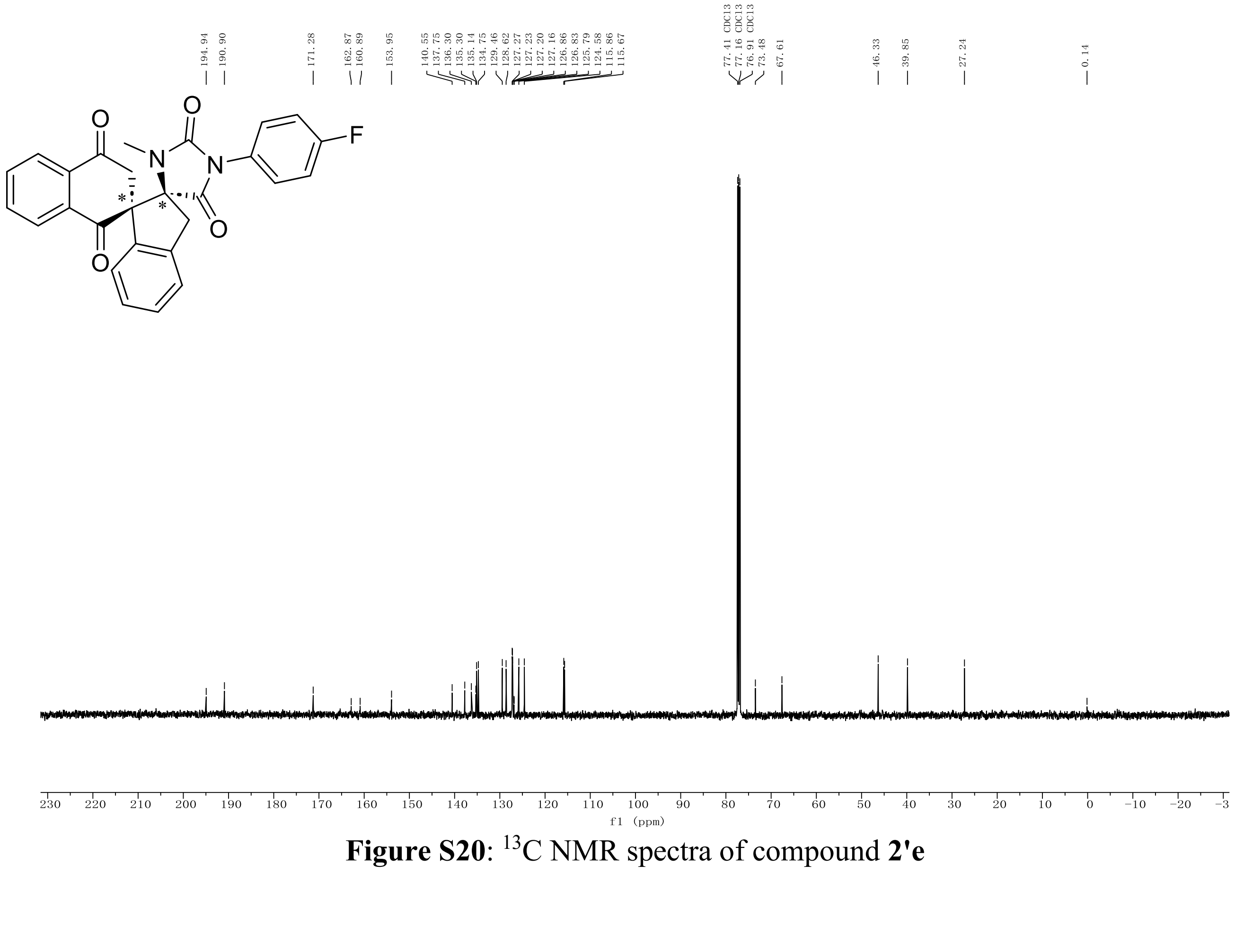

Figure S20: ^{13}C NMR spectra of compound **2'e**

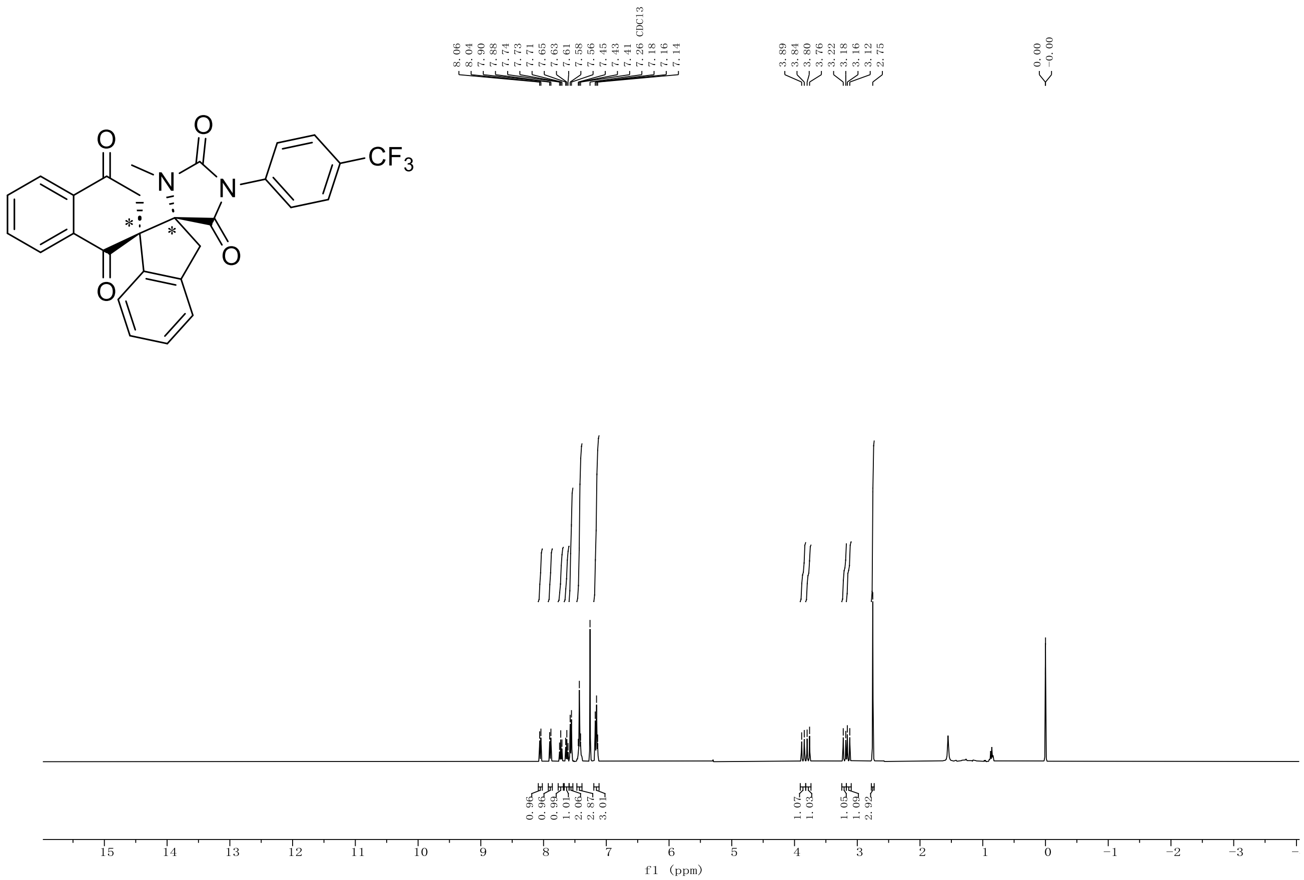

Figure S21: ^{1}H NMR spectra of compound **2f**

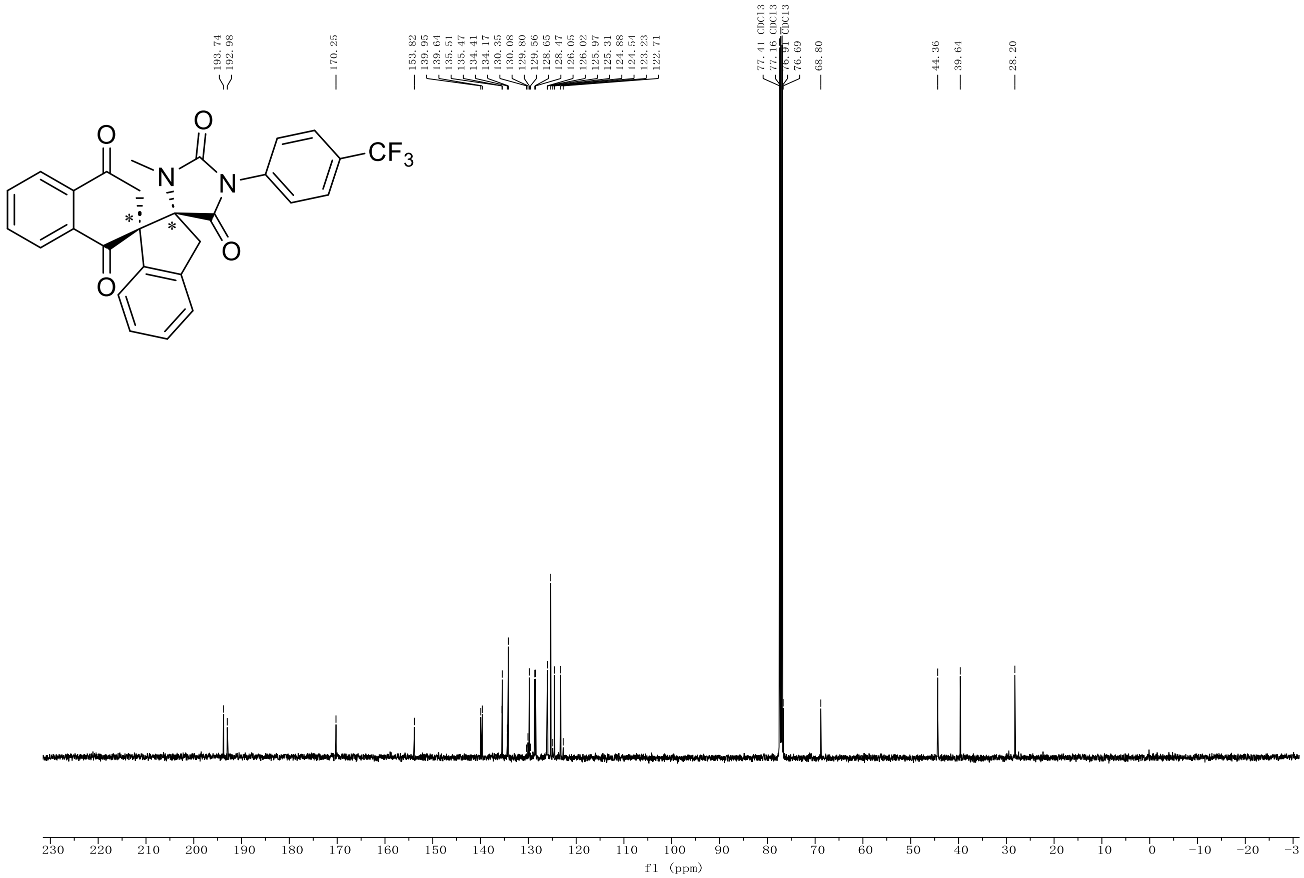

Figure S22: ^{13}C NMR spectra of compound **2f**

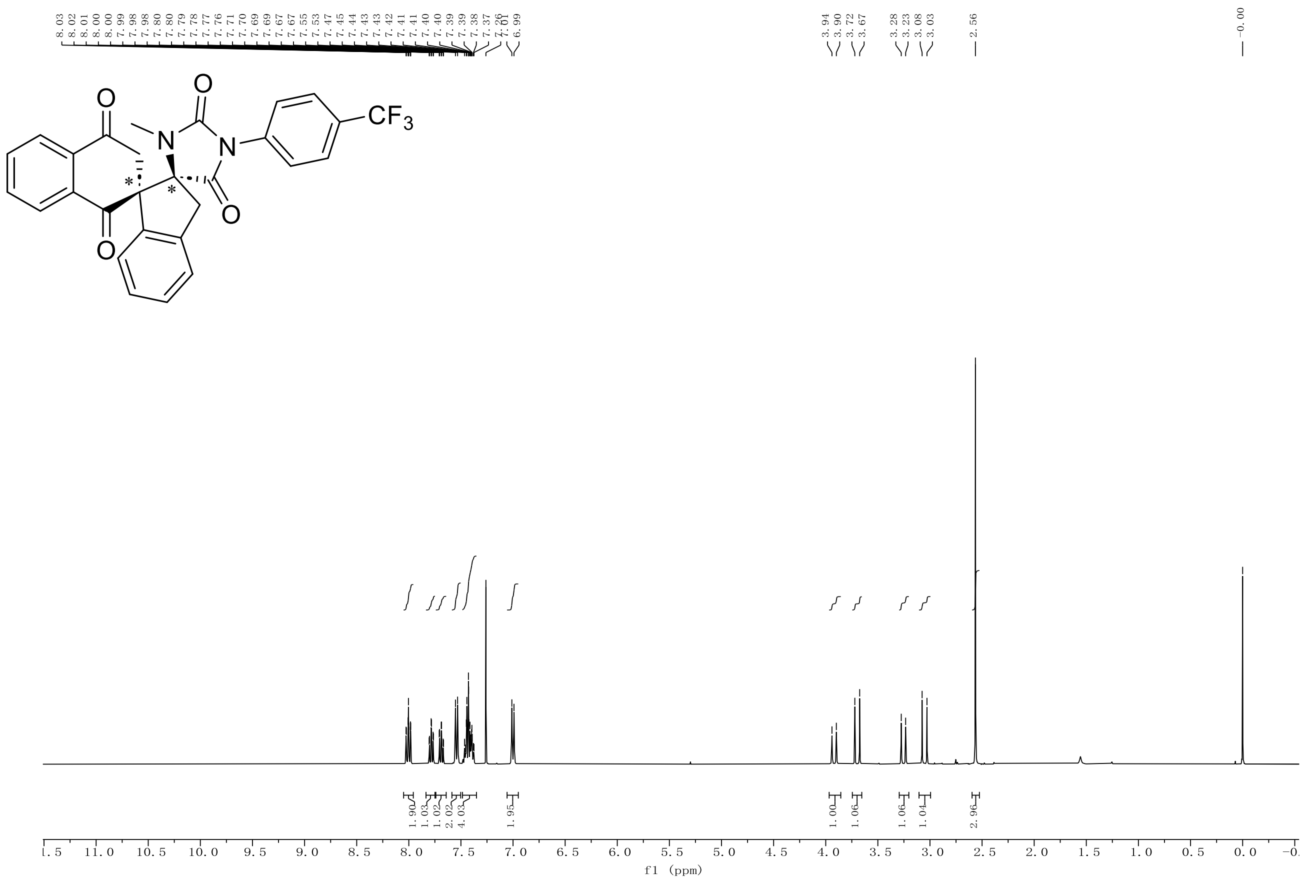

Figure S23: ^{1}H NMR spectra of compound **2'f**

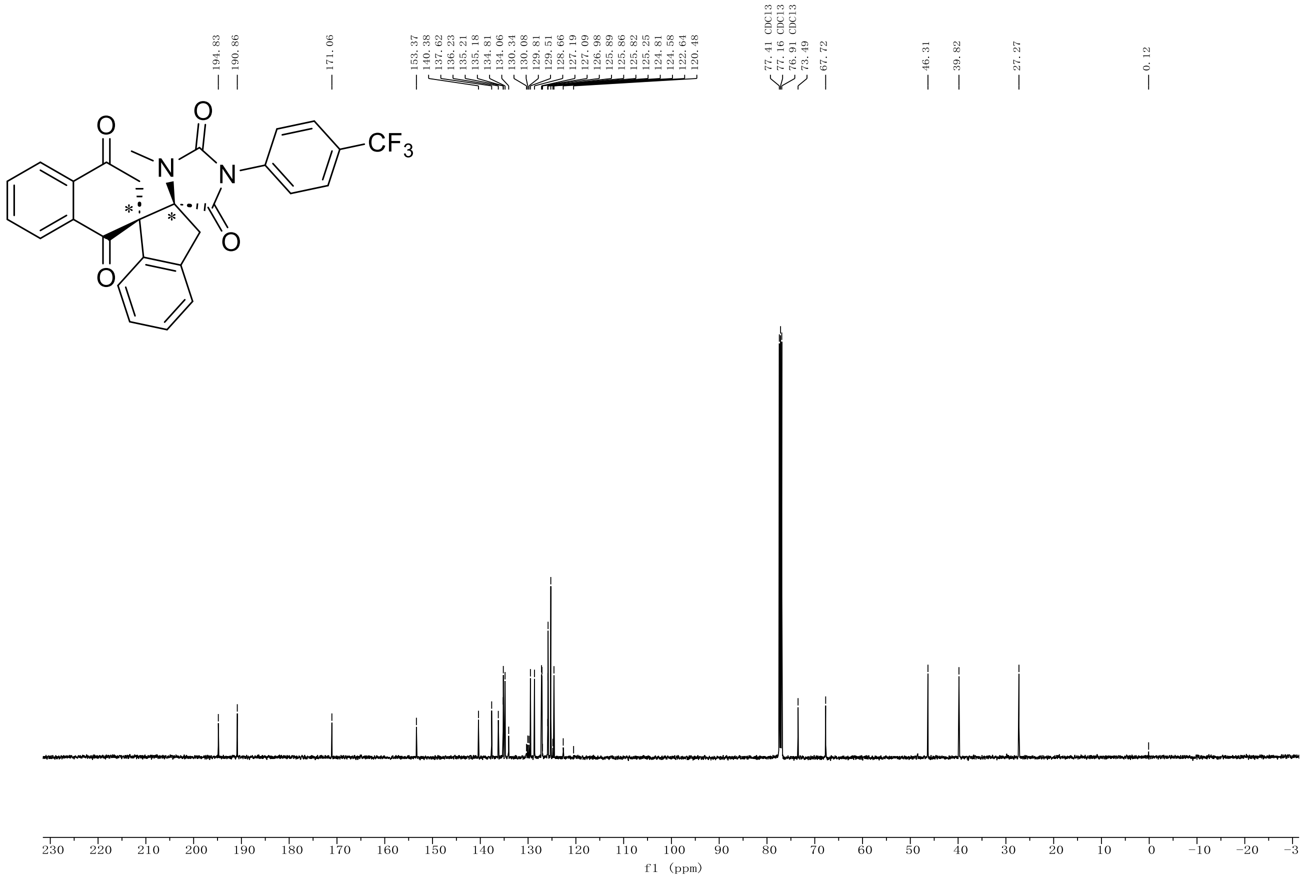

Figure S24: ^{13}C NMR spectra of compound **2'f**

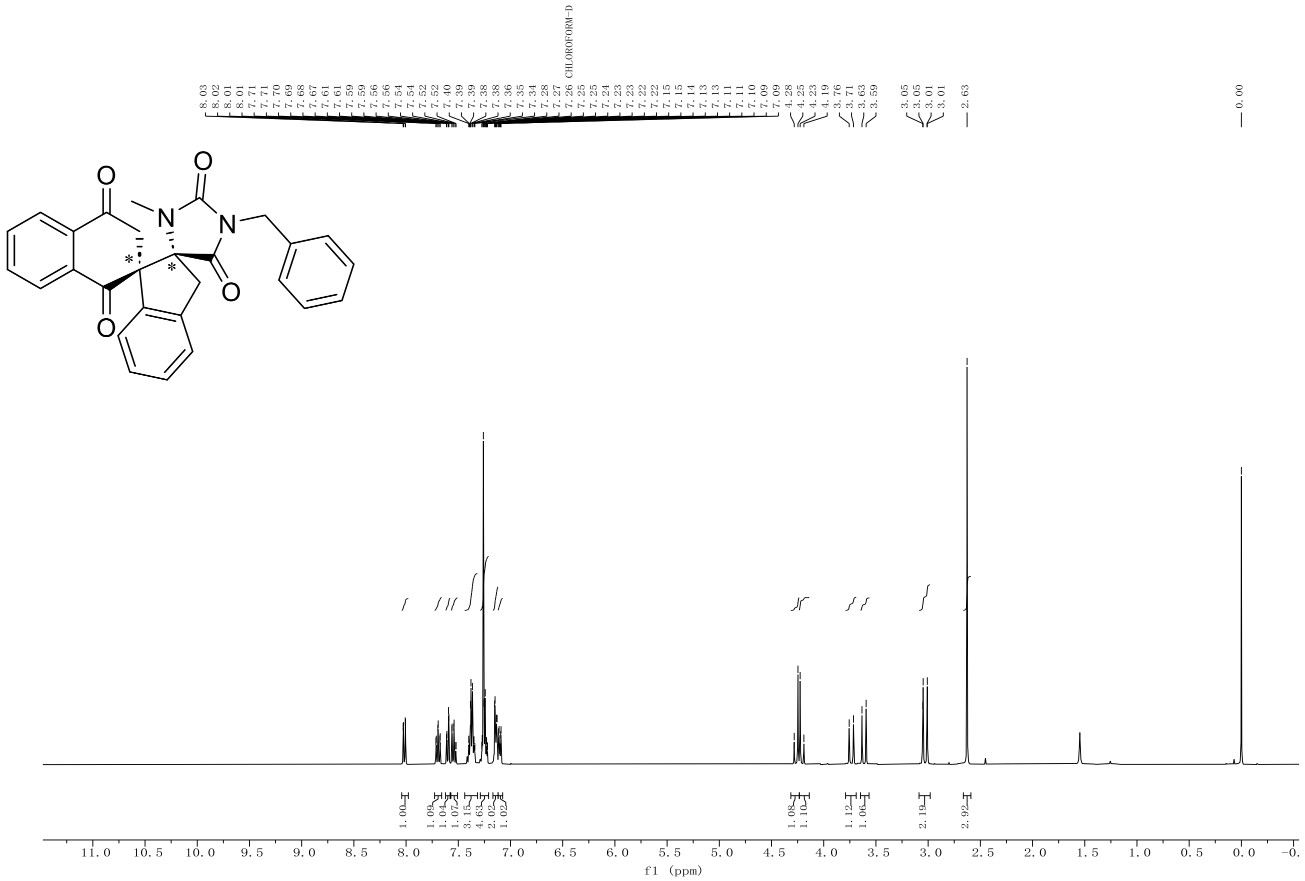

Figure S25: ^{1}H NMR spectra of compound **2g**

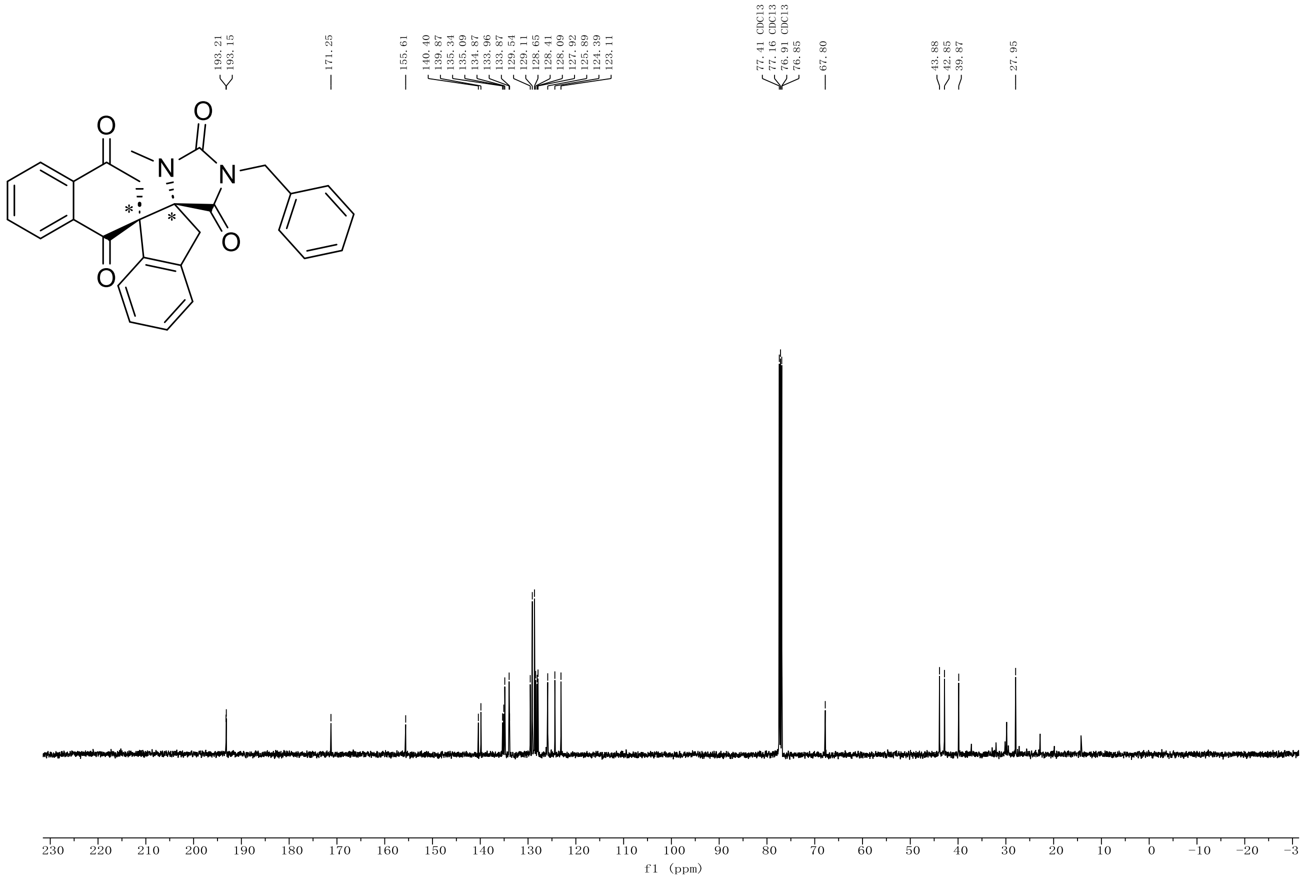

Figure S26: ^{13}C NMR spectra of compound **2g**

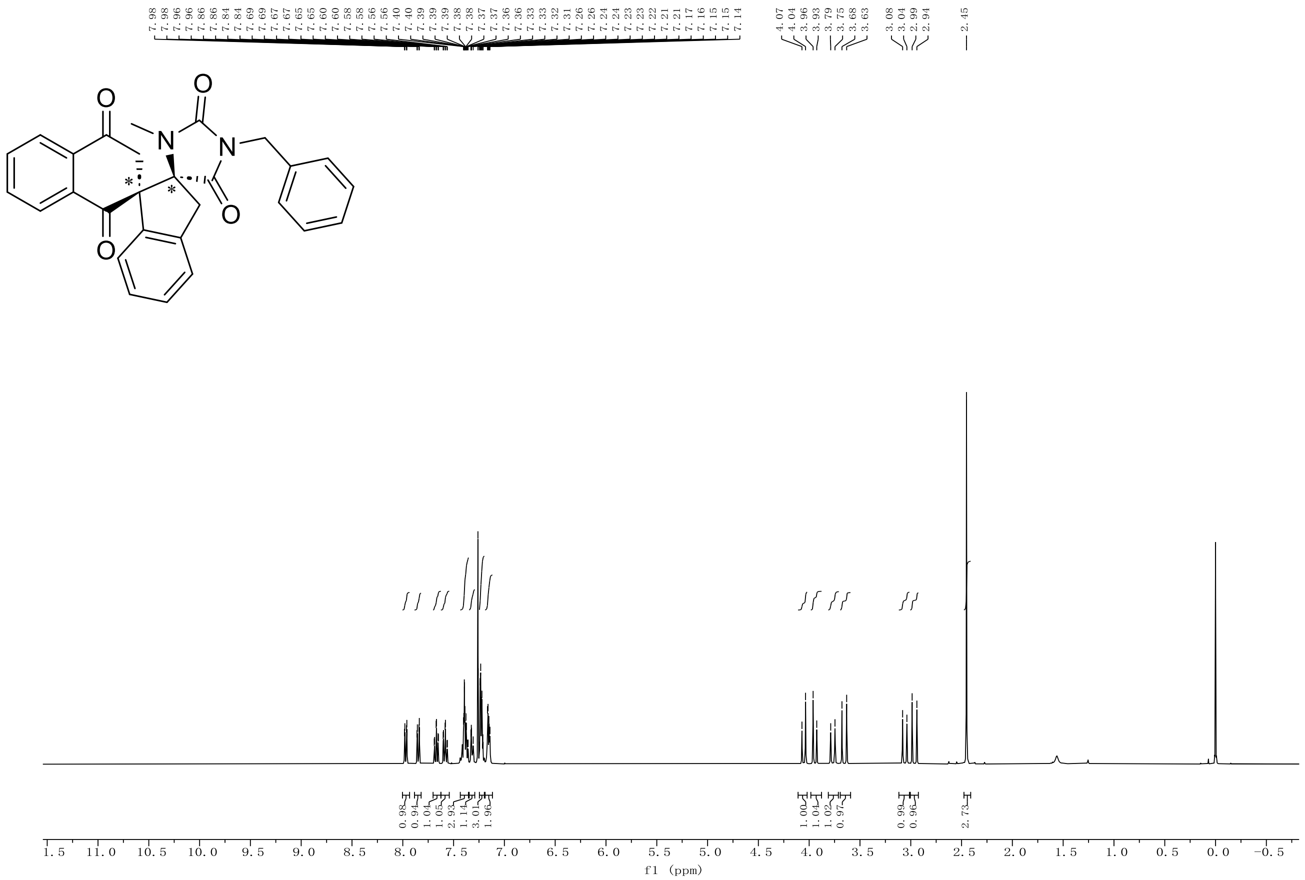

Figure S27: ^{1}H NMR spectra of compound **2'g**

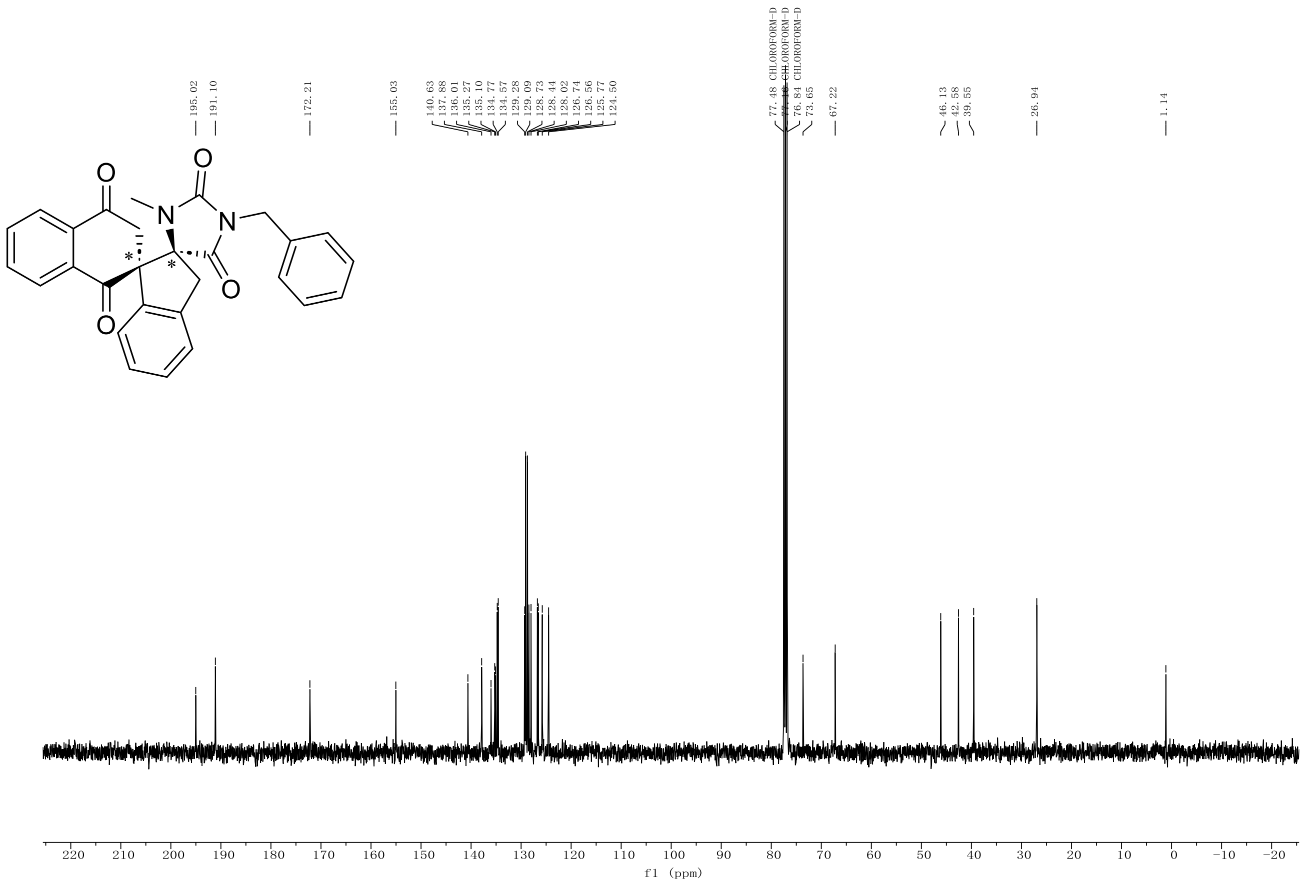

Figure S28: ^{13}C NMR spectra of compound **2'g**

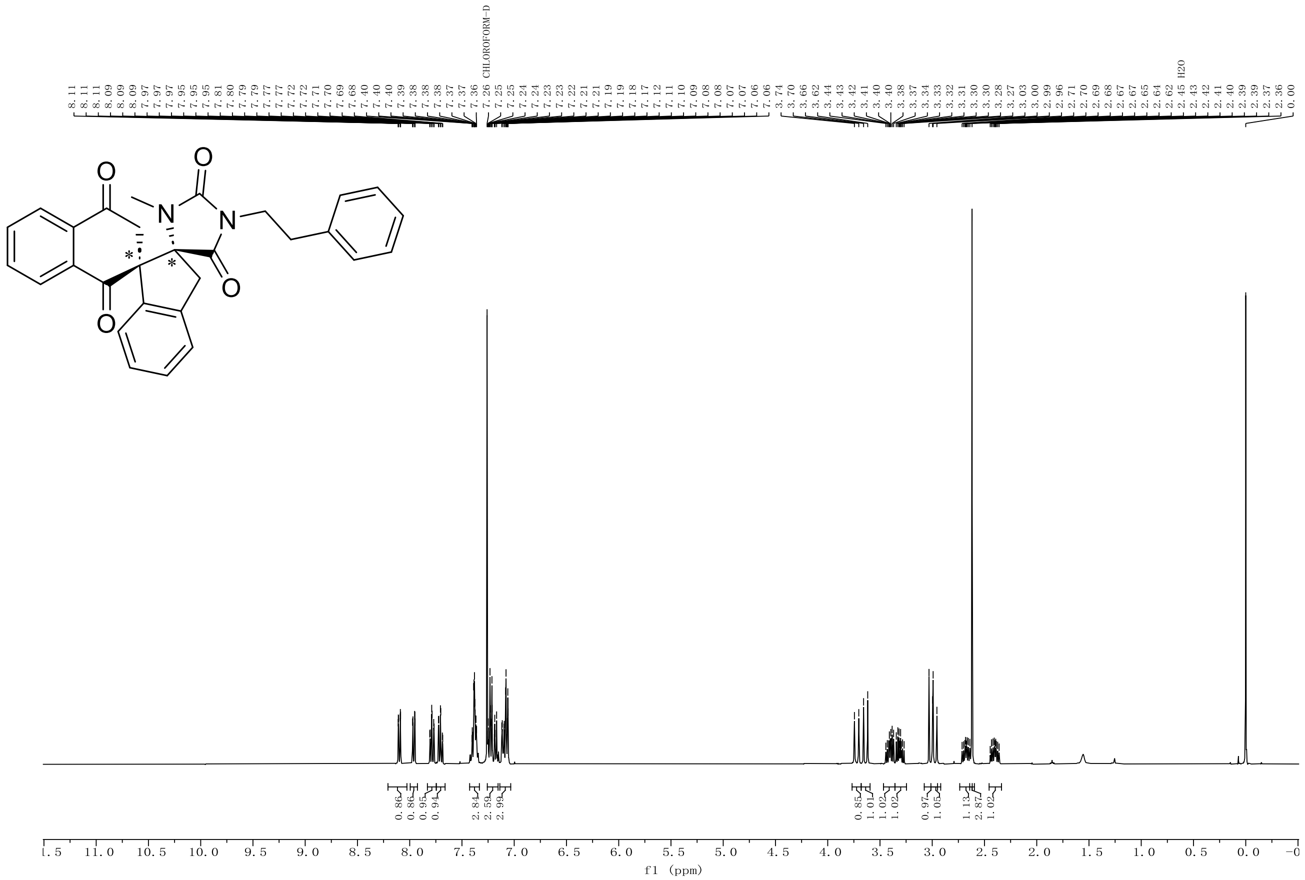

Figure S29: ^{1}H NMR spectra of compound **2h**

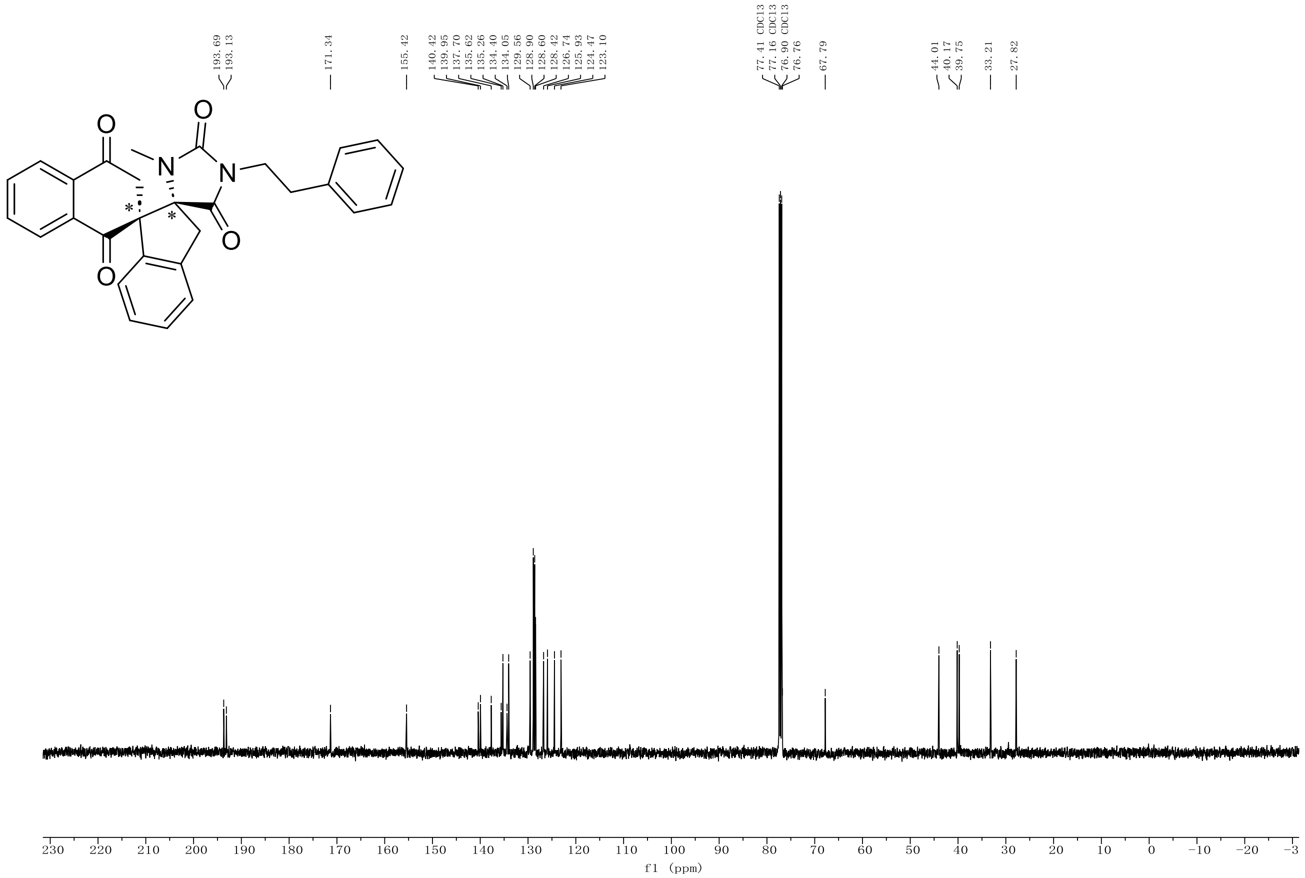

Figure S30: ^{13}C NMR spectra of compound **2h**

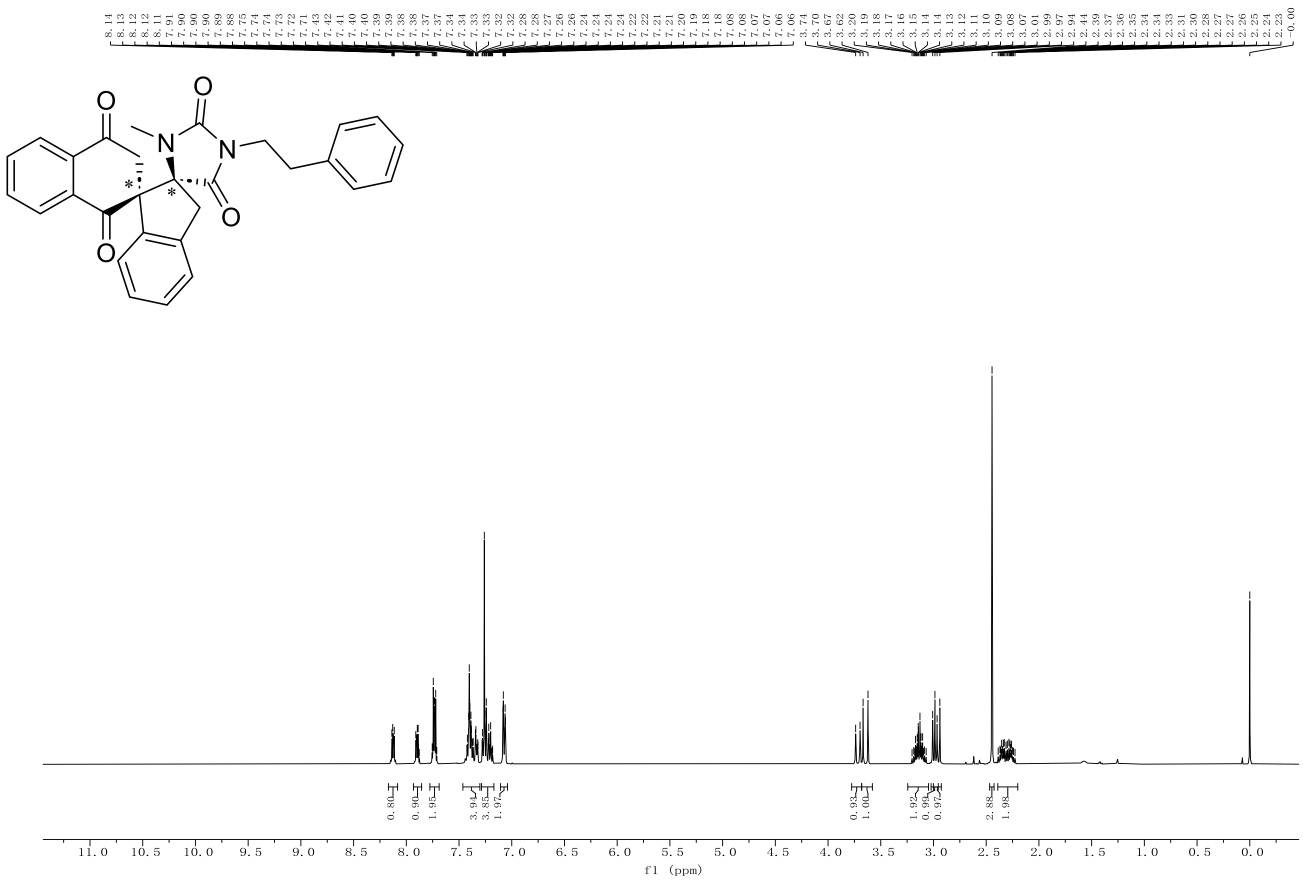

Figure S31: ^{1}H NMR spectra of compound **2'h**

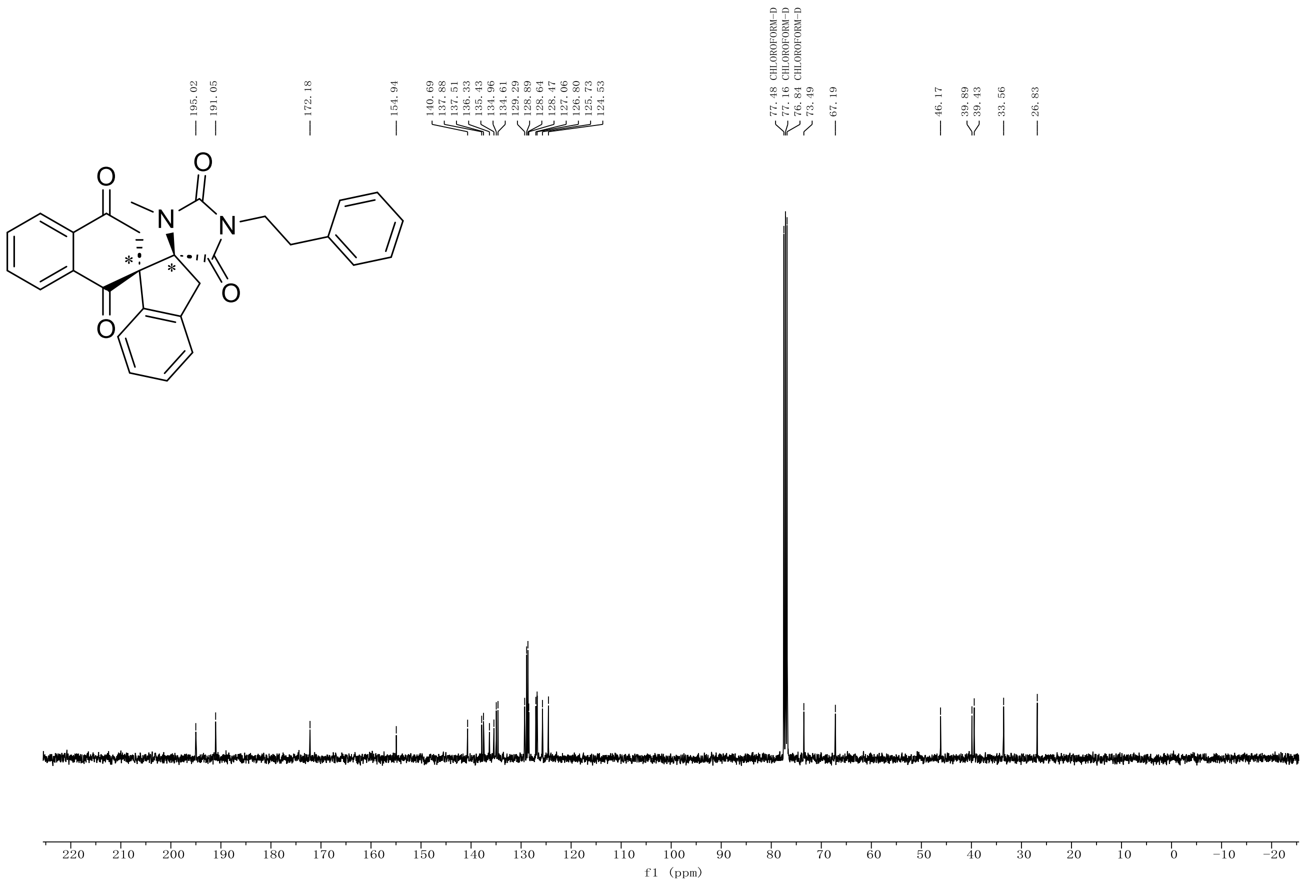

Figure S32: ^{13}C NMR spectra of compound **2'h**

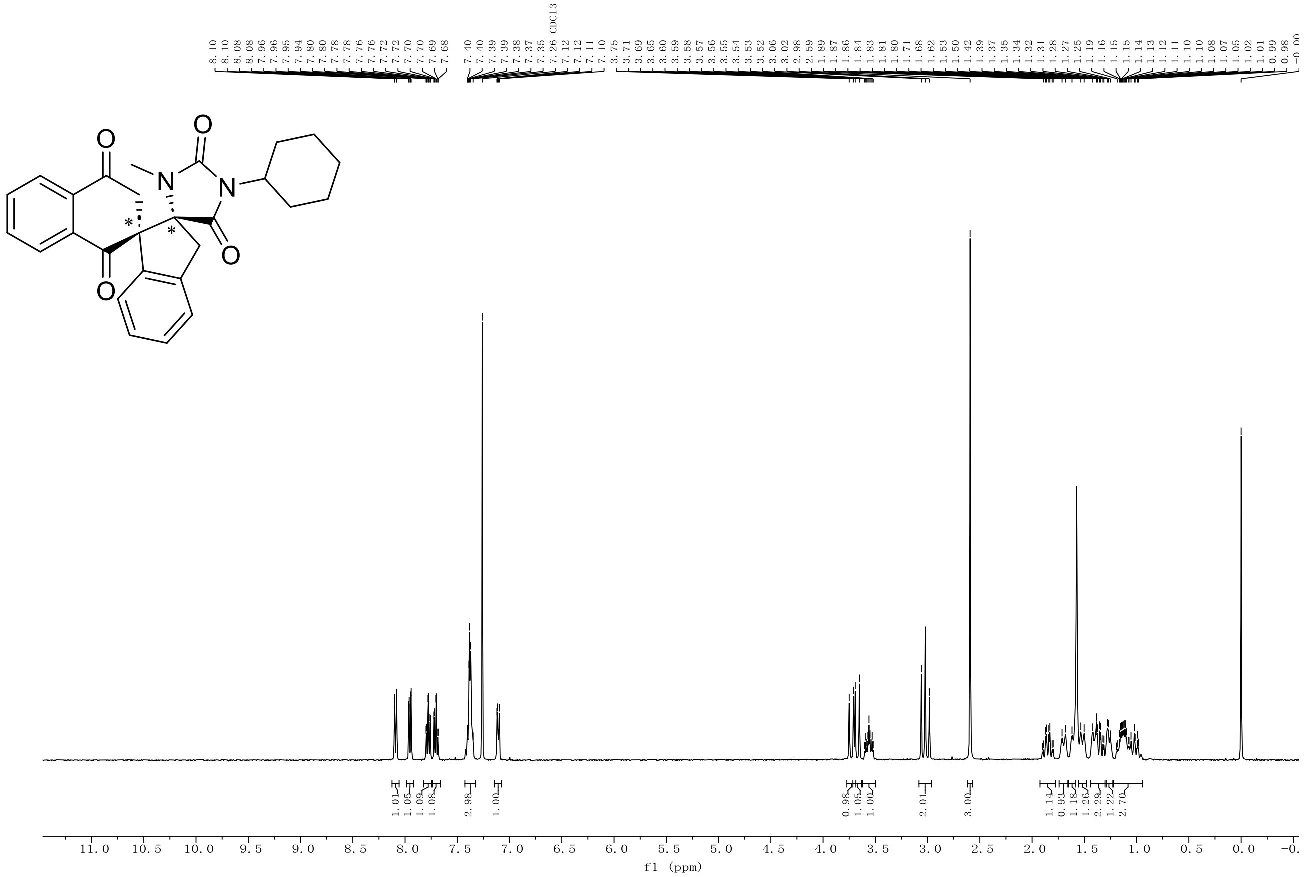

Figure S33: ^{1}H NMR spectra of compound **2i**

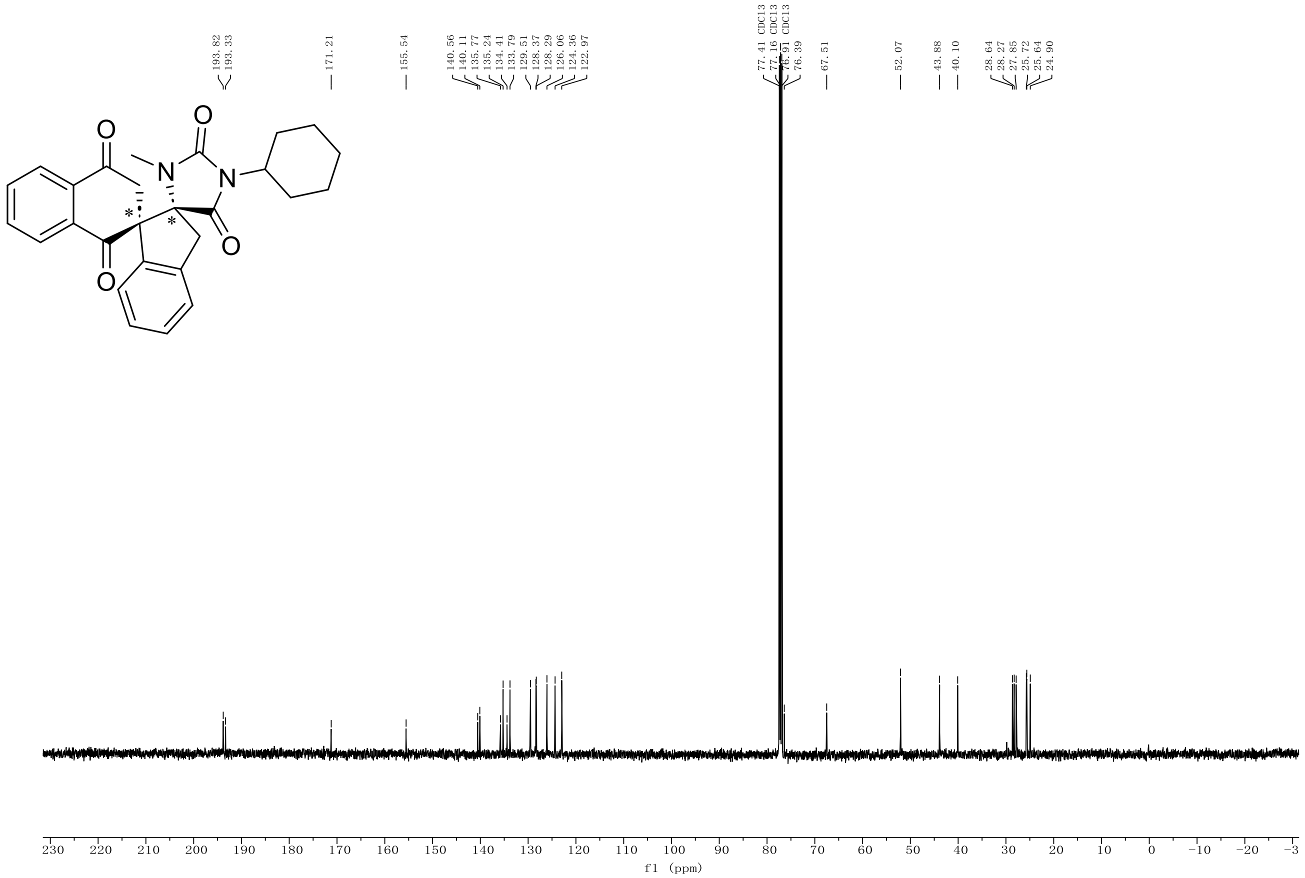

Figure S34: ^{13}C NMR spectra of compound **2i**

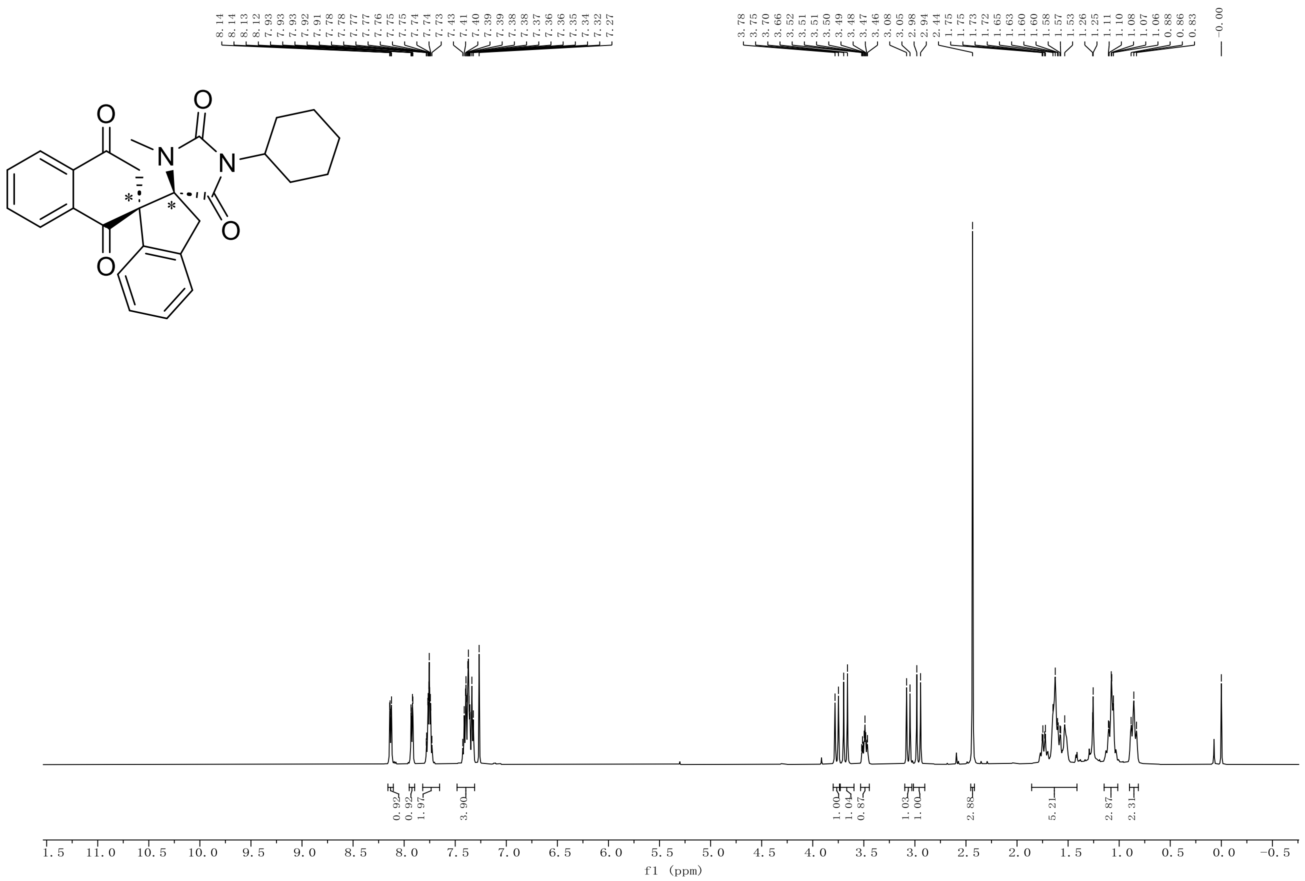

Figure S35: ^{1}H NMR spectra of compound **2'i**

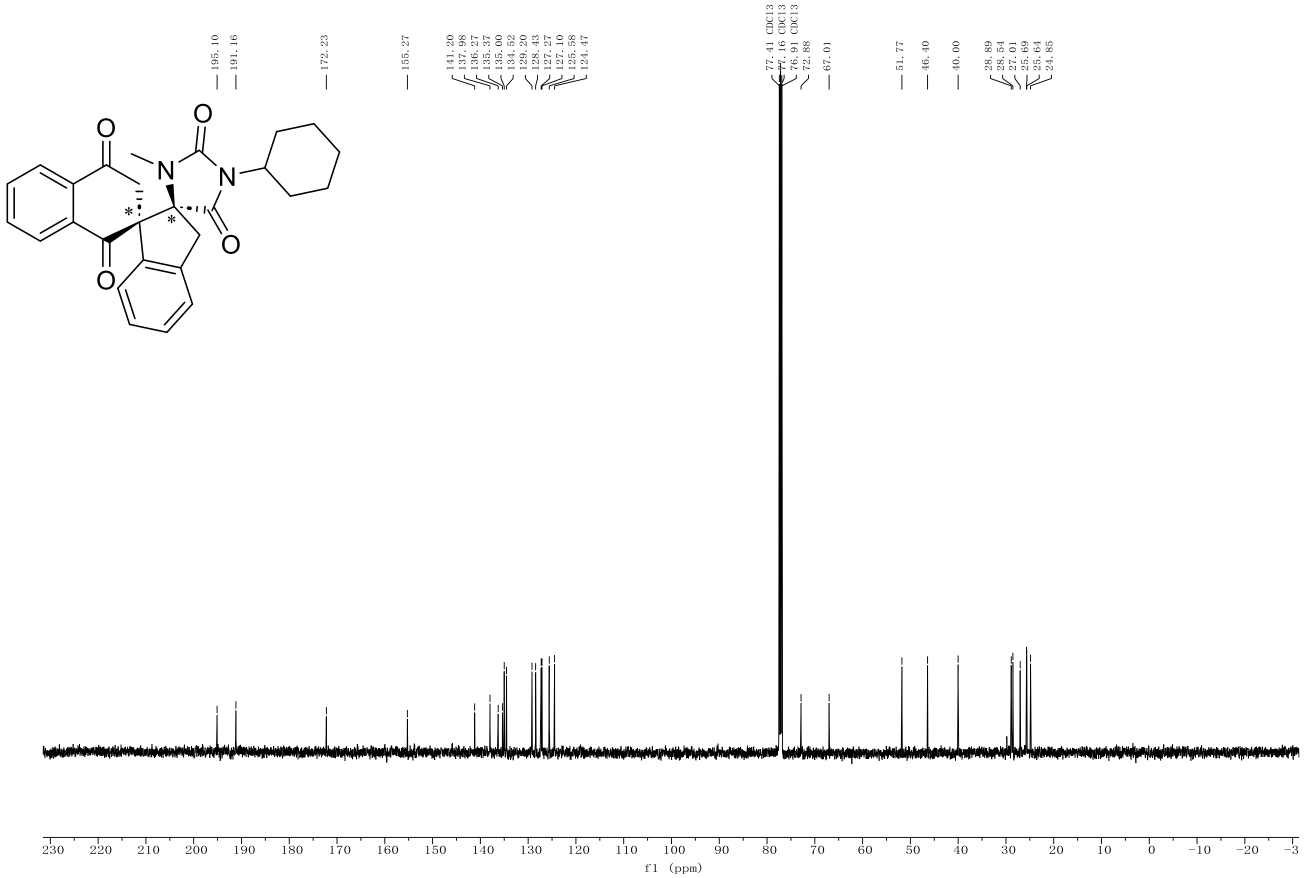

Figure S36: ^{13}C NMR spectra of compound **2'i**

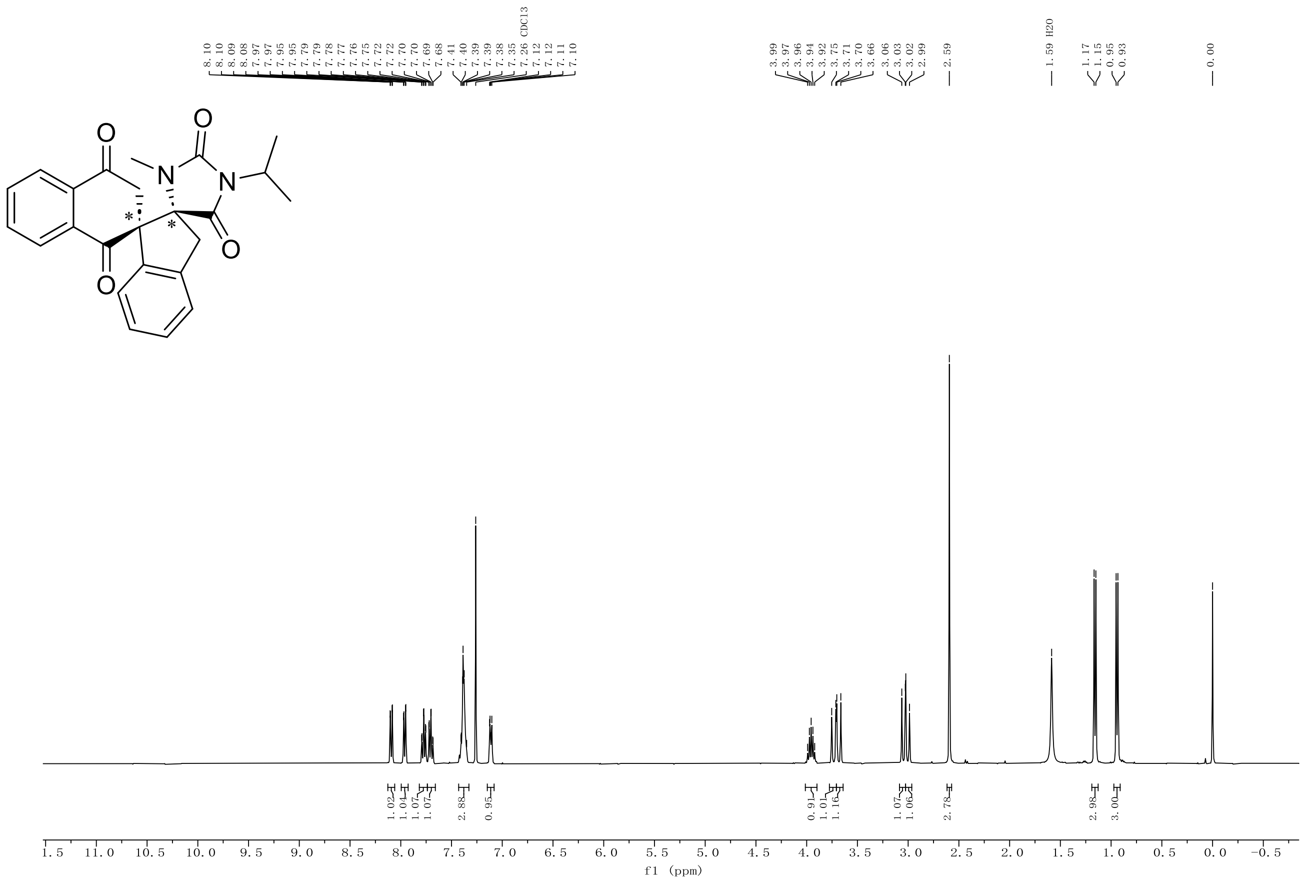

Figure S37: ^{1}H NMR spectra of compound **2j**

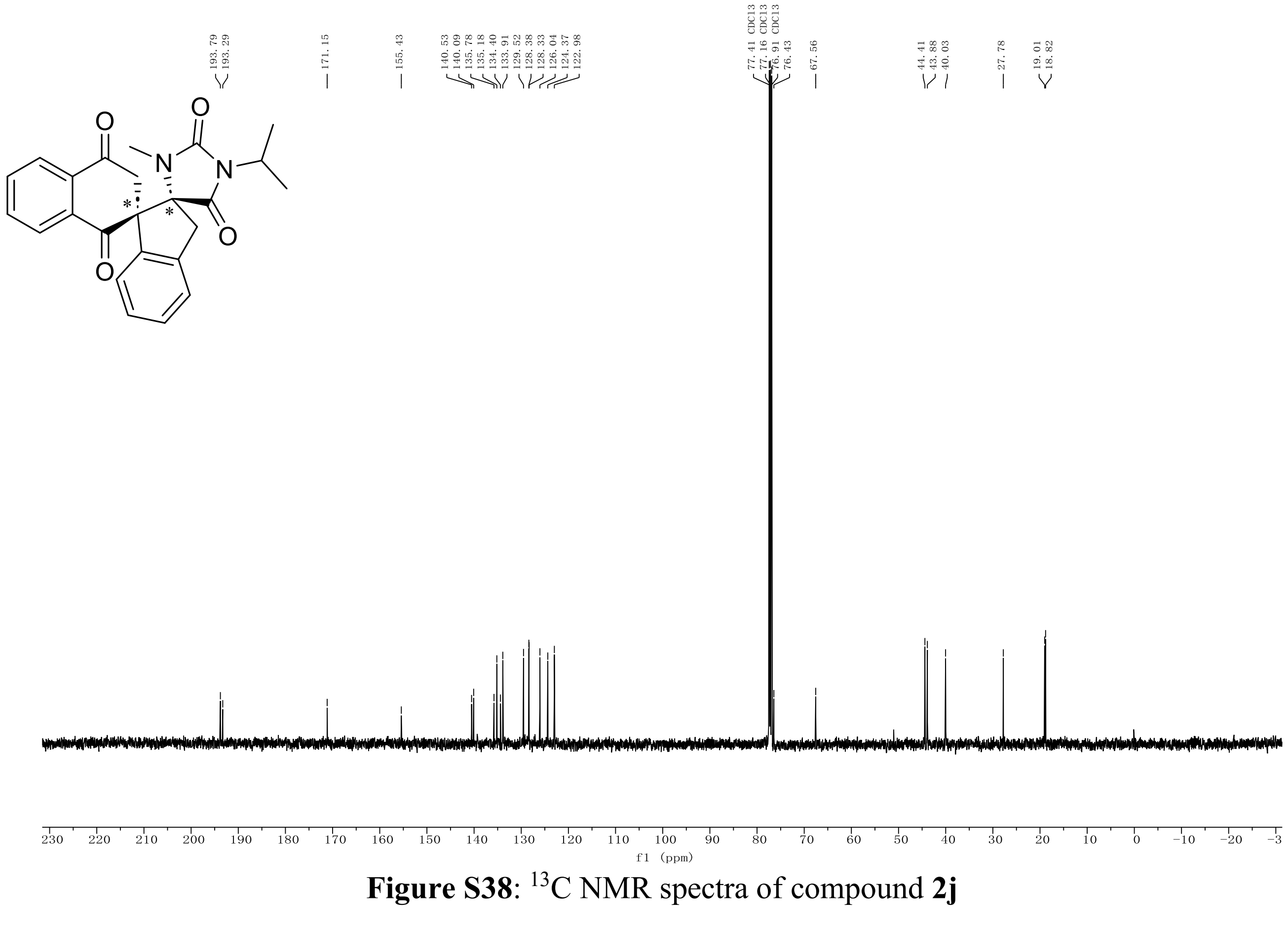

Figure S38: ^{13}C NMR spectra of compound **2j**

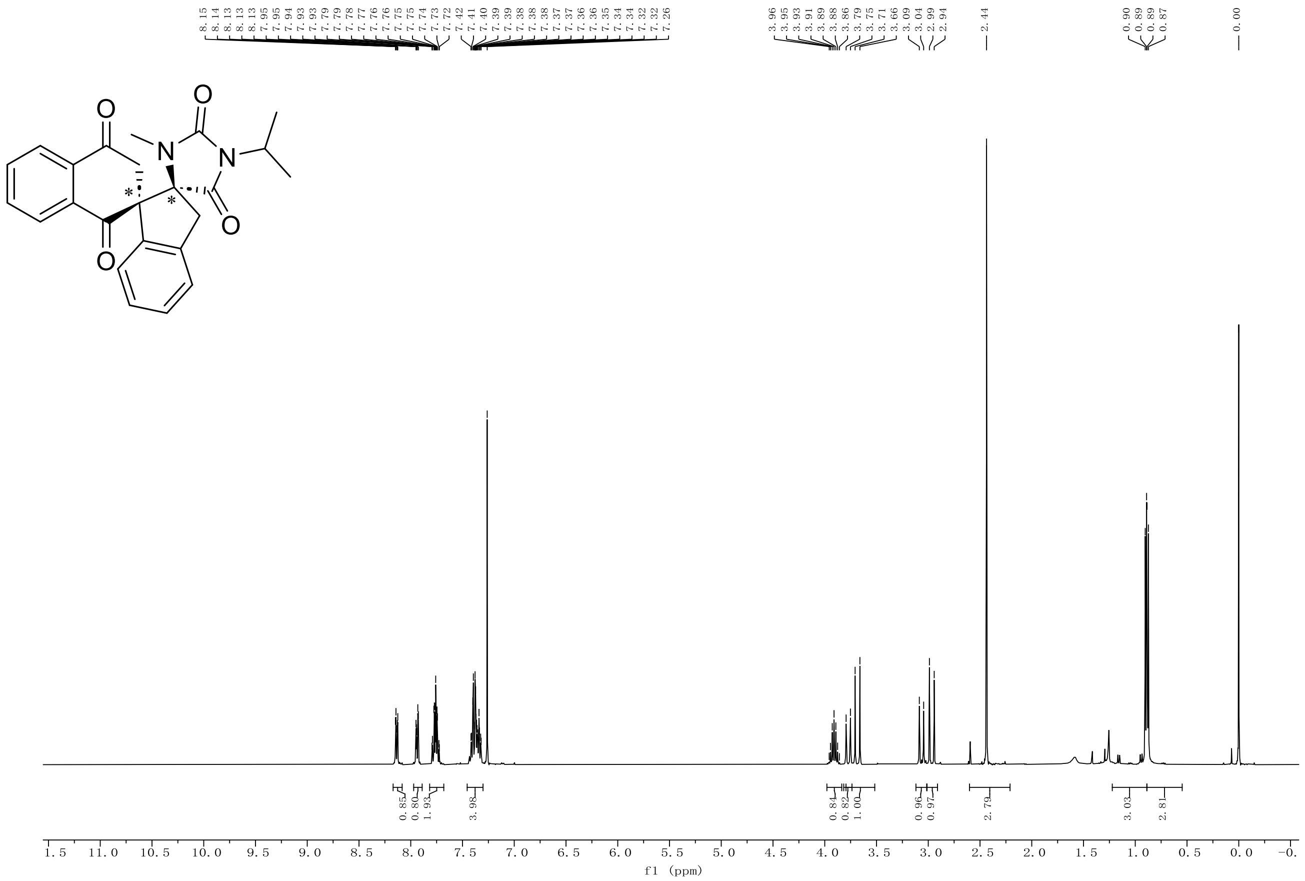

Figure S39: ^{1}H NMR spectra of compound **2'j**

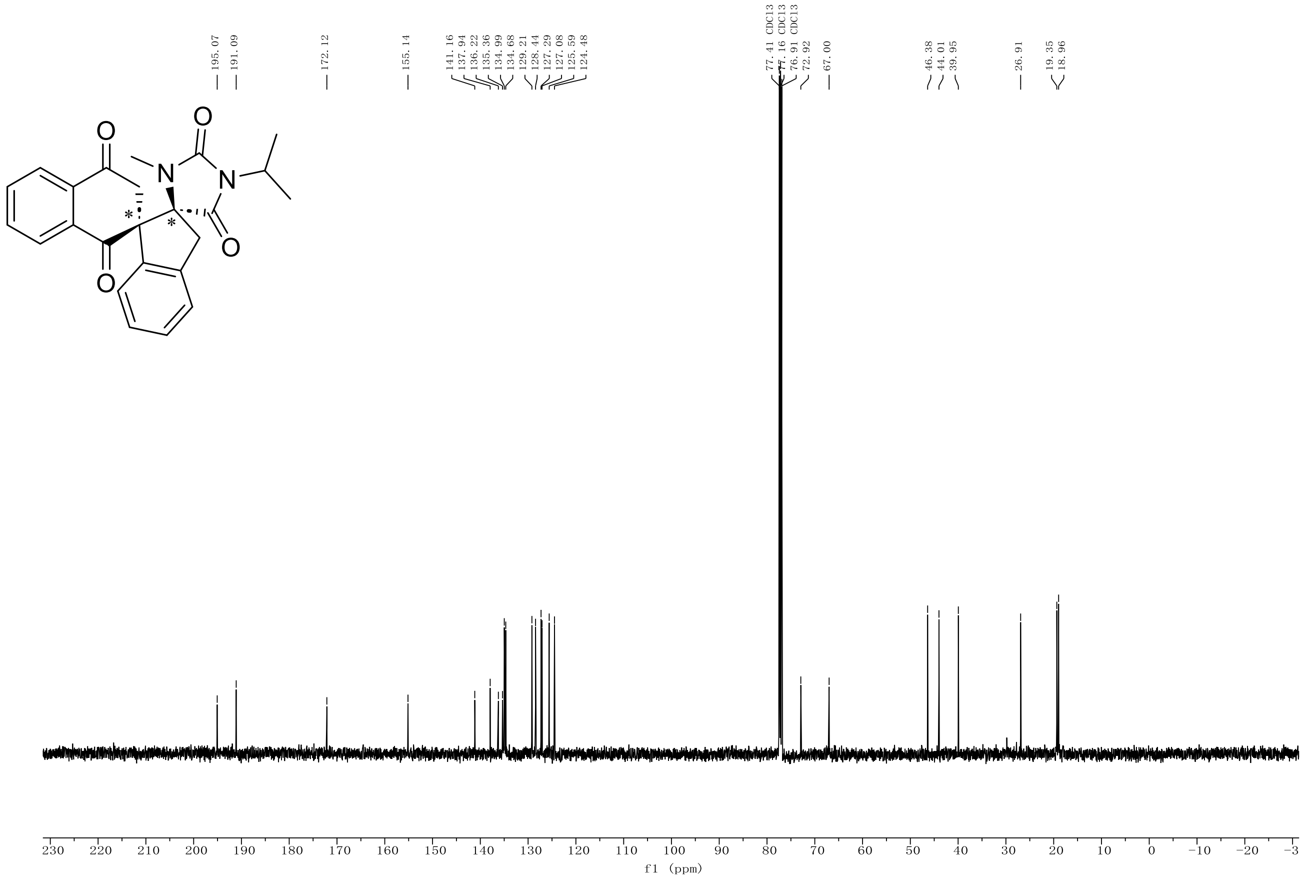

Figure S40: ^{13}C NMR spectra of compound **2'j**

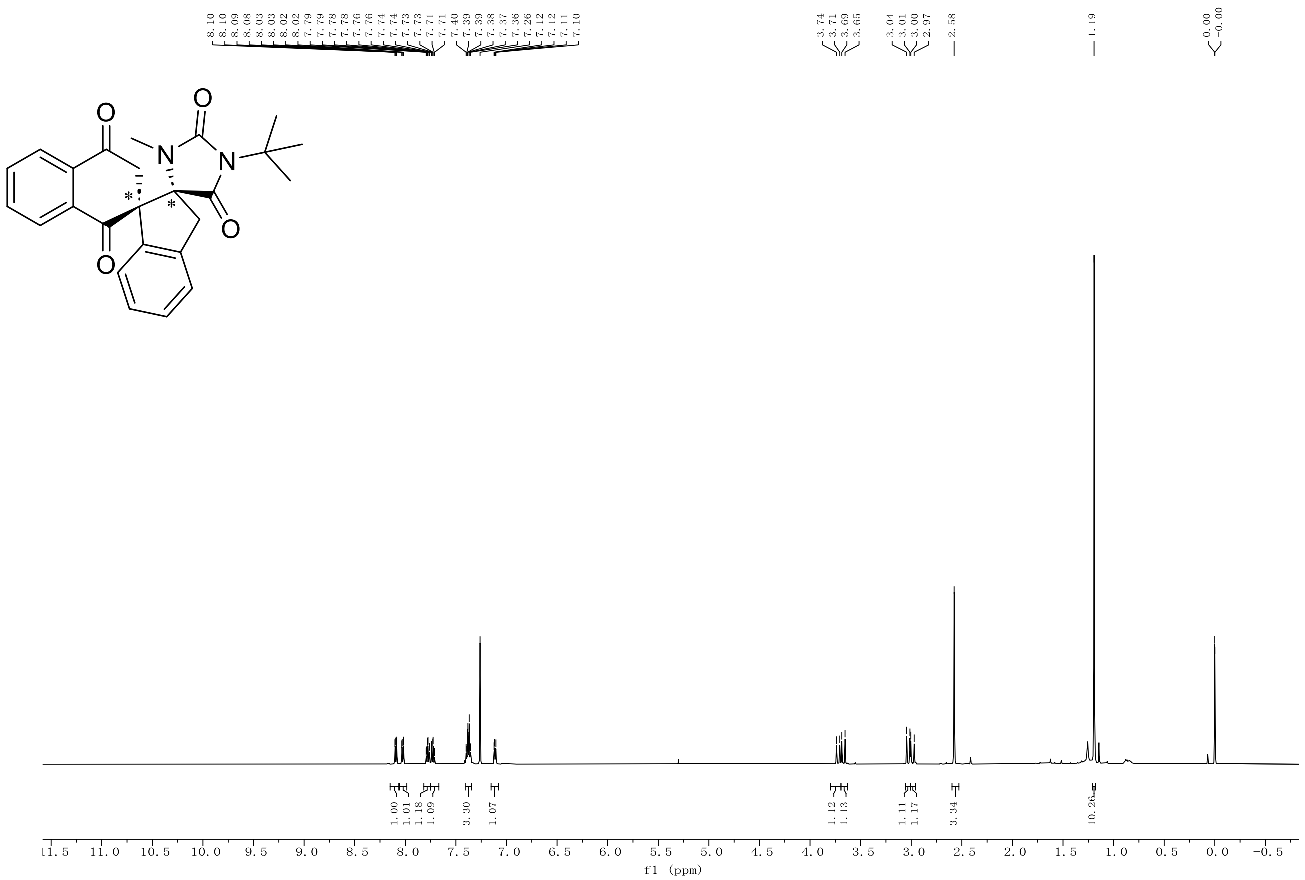

Figure S41: ^{1}H NMR spectra of compound **2k**

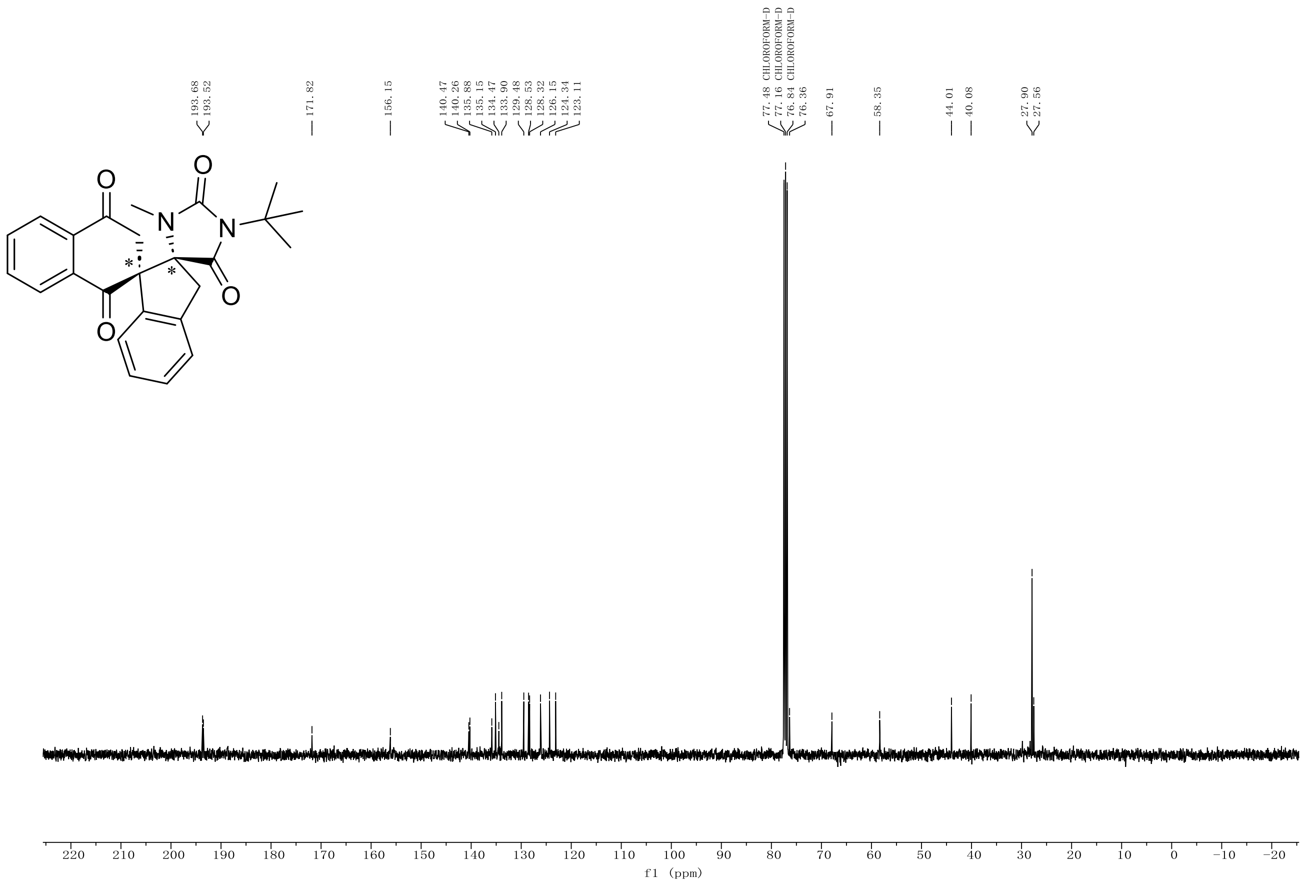

Figure S42: ^{13}C NMR spectra of compound **2k**

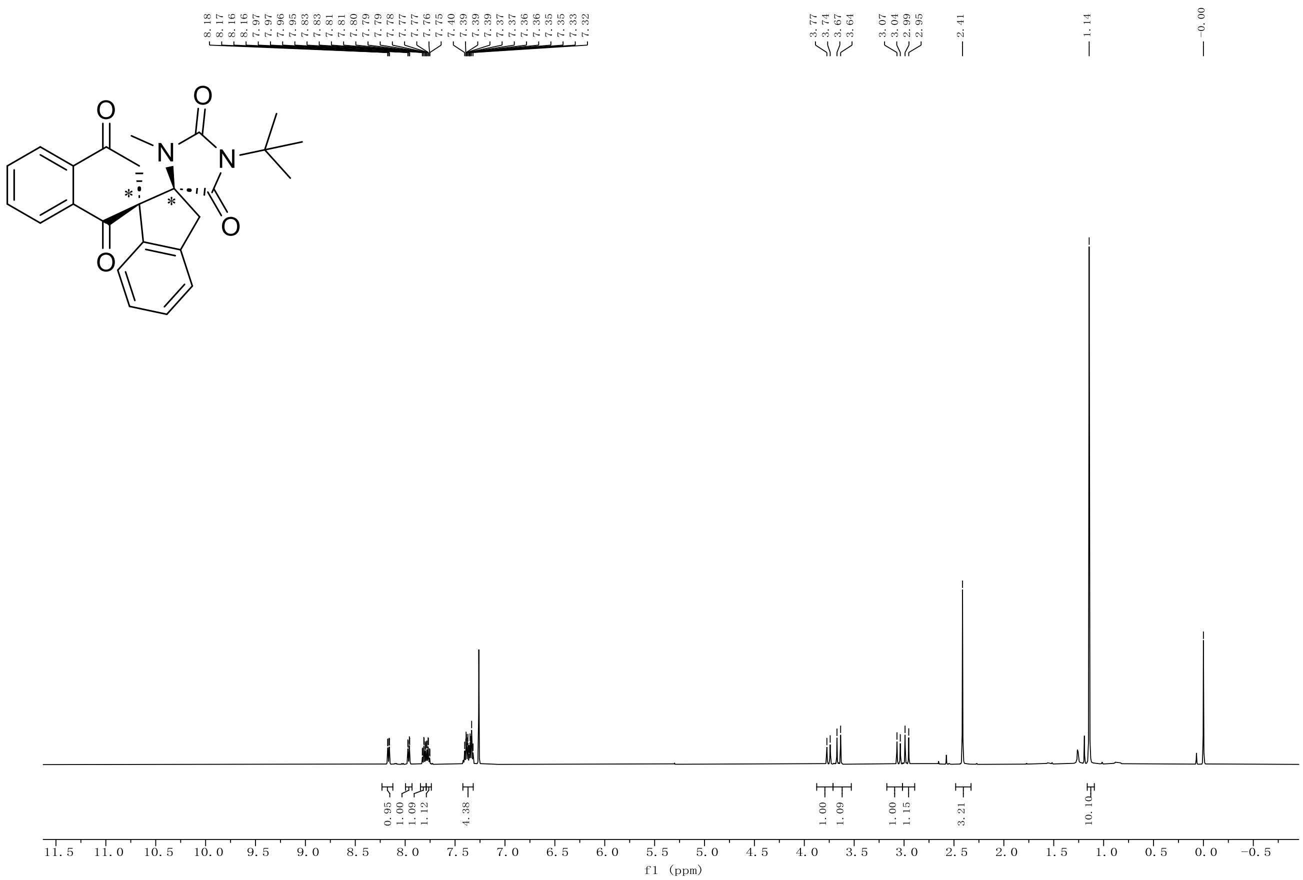

Figure S43: ^{1}H NMR spectra of compound **2'k**

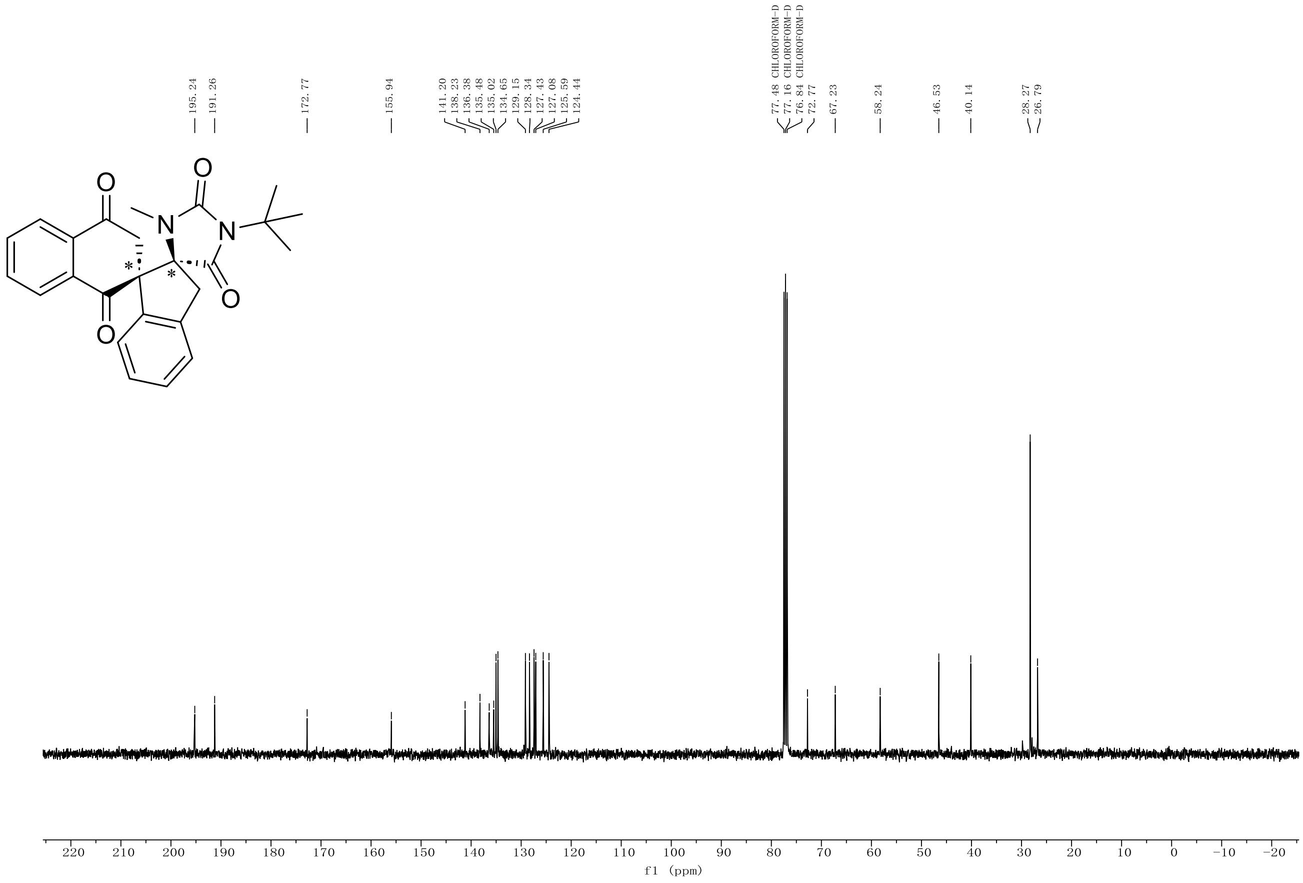

Figure S44: ^{13}C NMR spectra of compound **2'k**

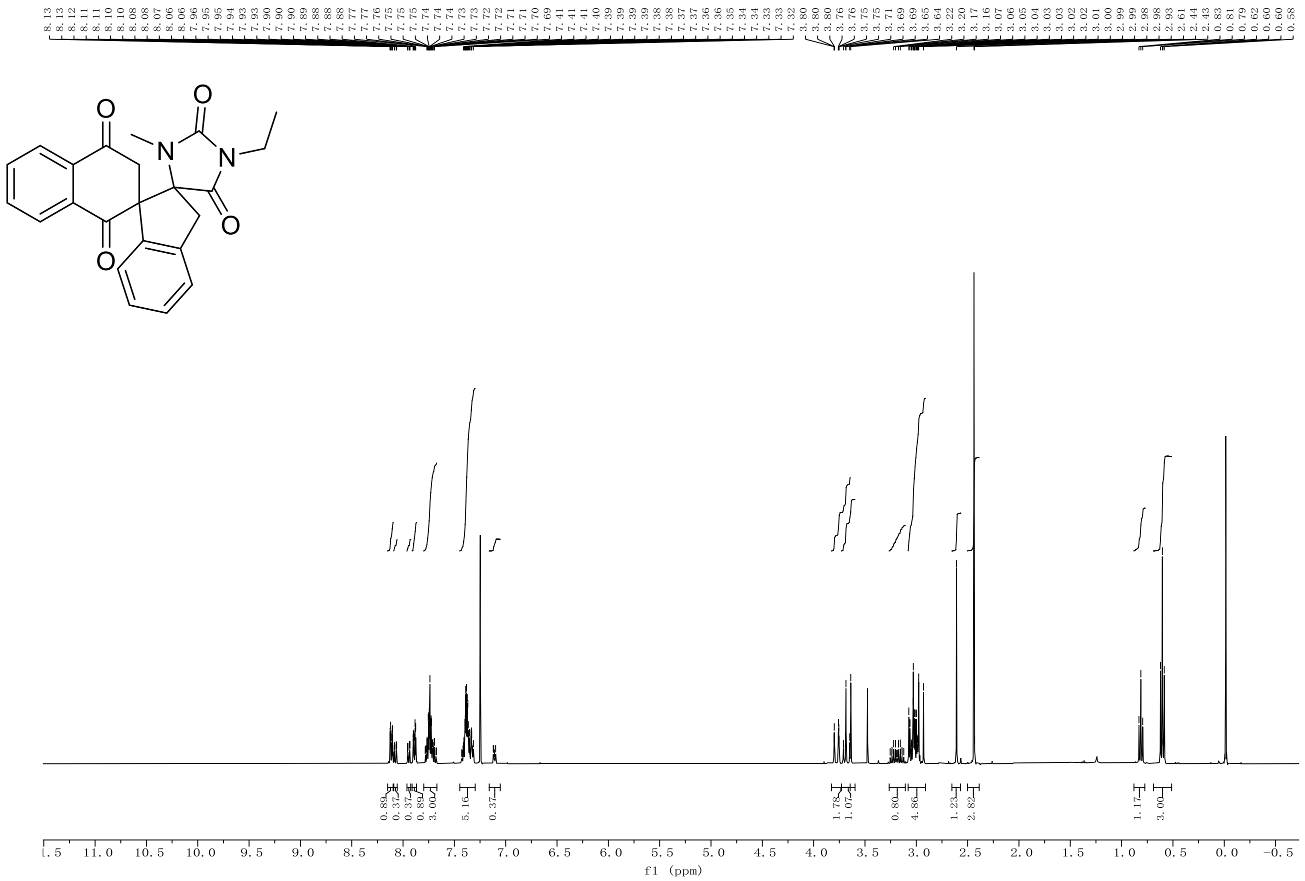

Figure S45: ^{1}H NMR spectra of compound **2l** and **2'l**

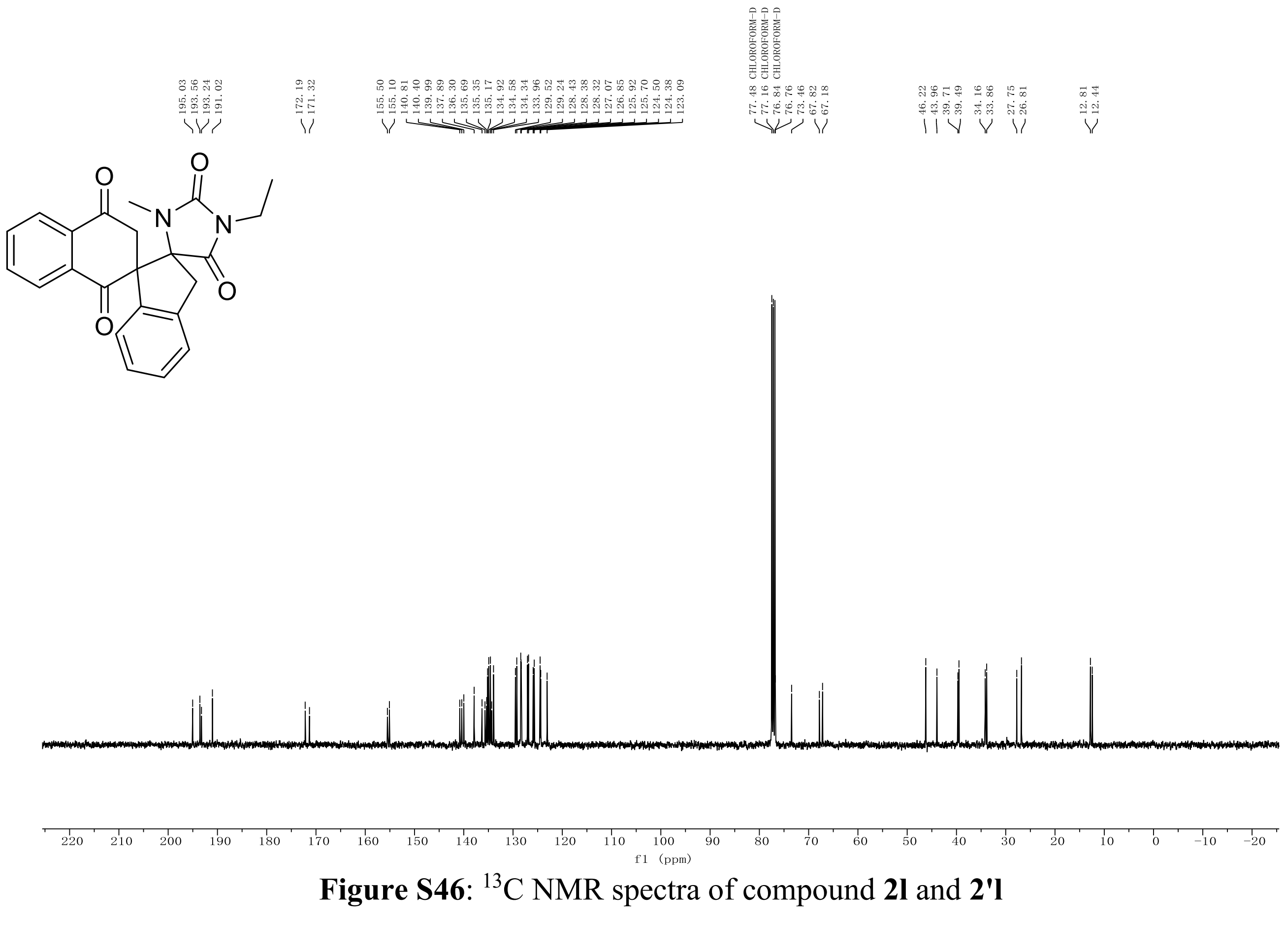

Figure S46: ^{13}C NMR spectra of compound **2l** and **2'l**

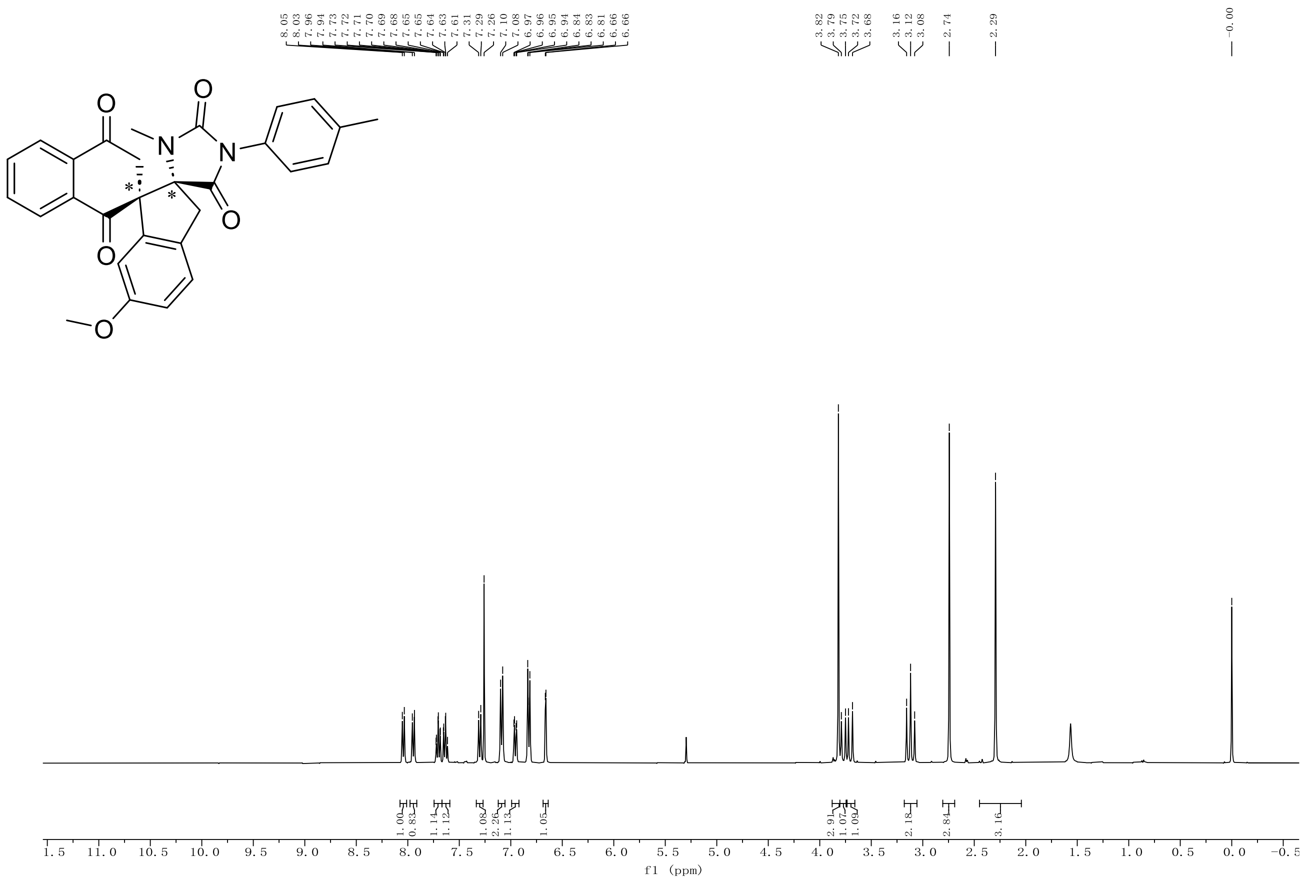

Figure S47: ^{1}H NMR spectra of compound **2m**

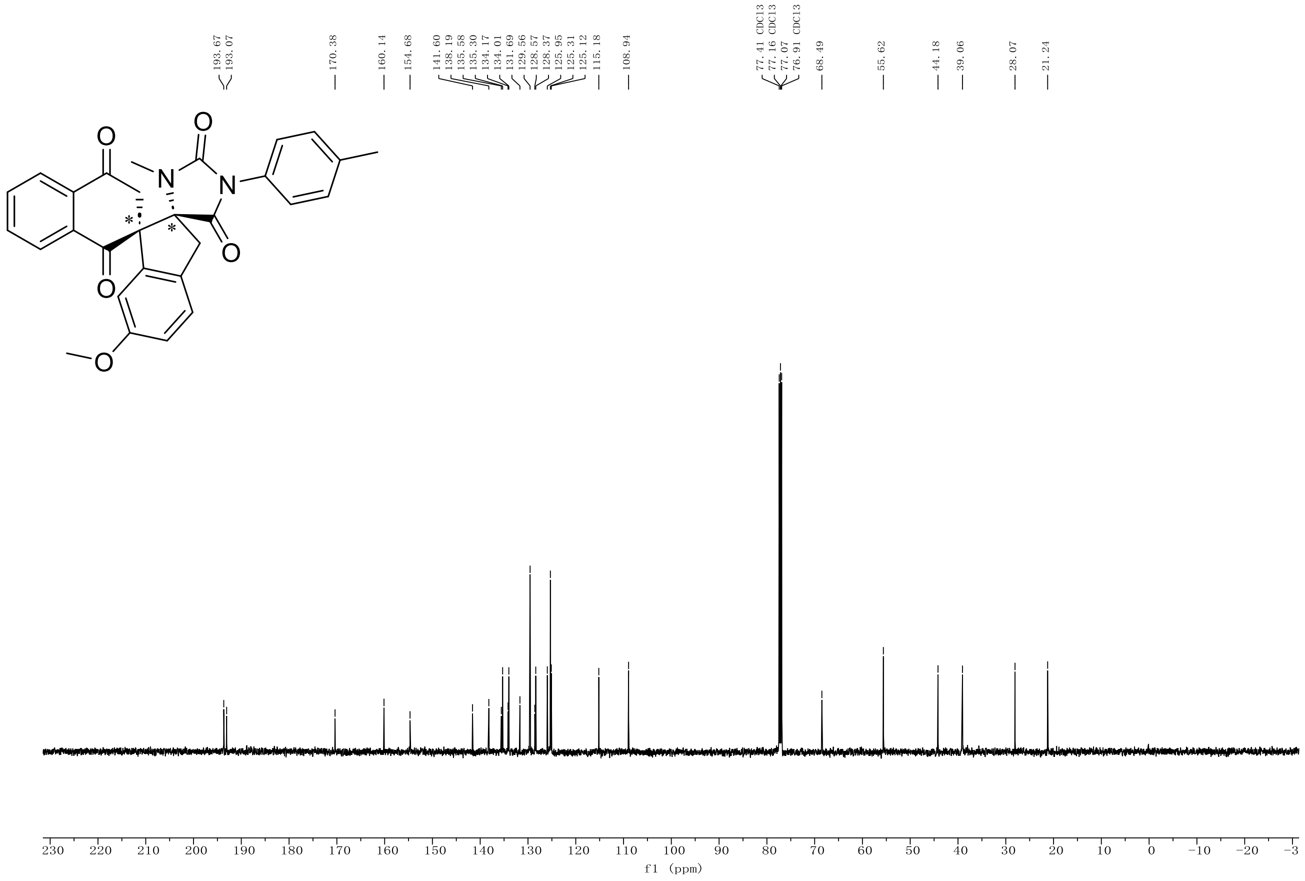

Figure S48: ^{13}C NMR spectra of compound **2m**

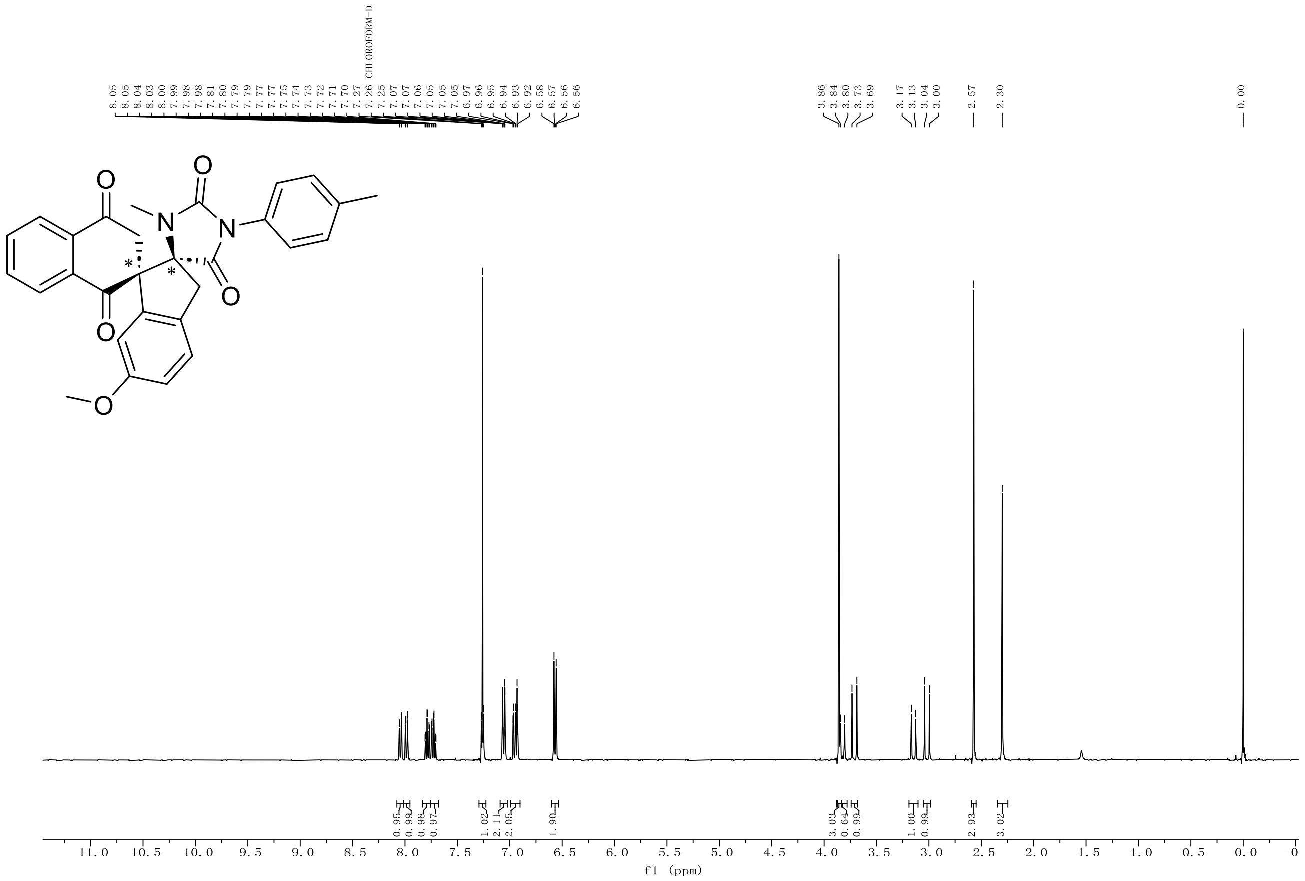

Figure S49: ^{1}H NMR spectra of compound **2'm**

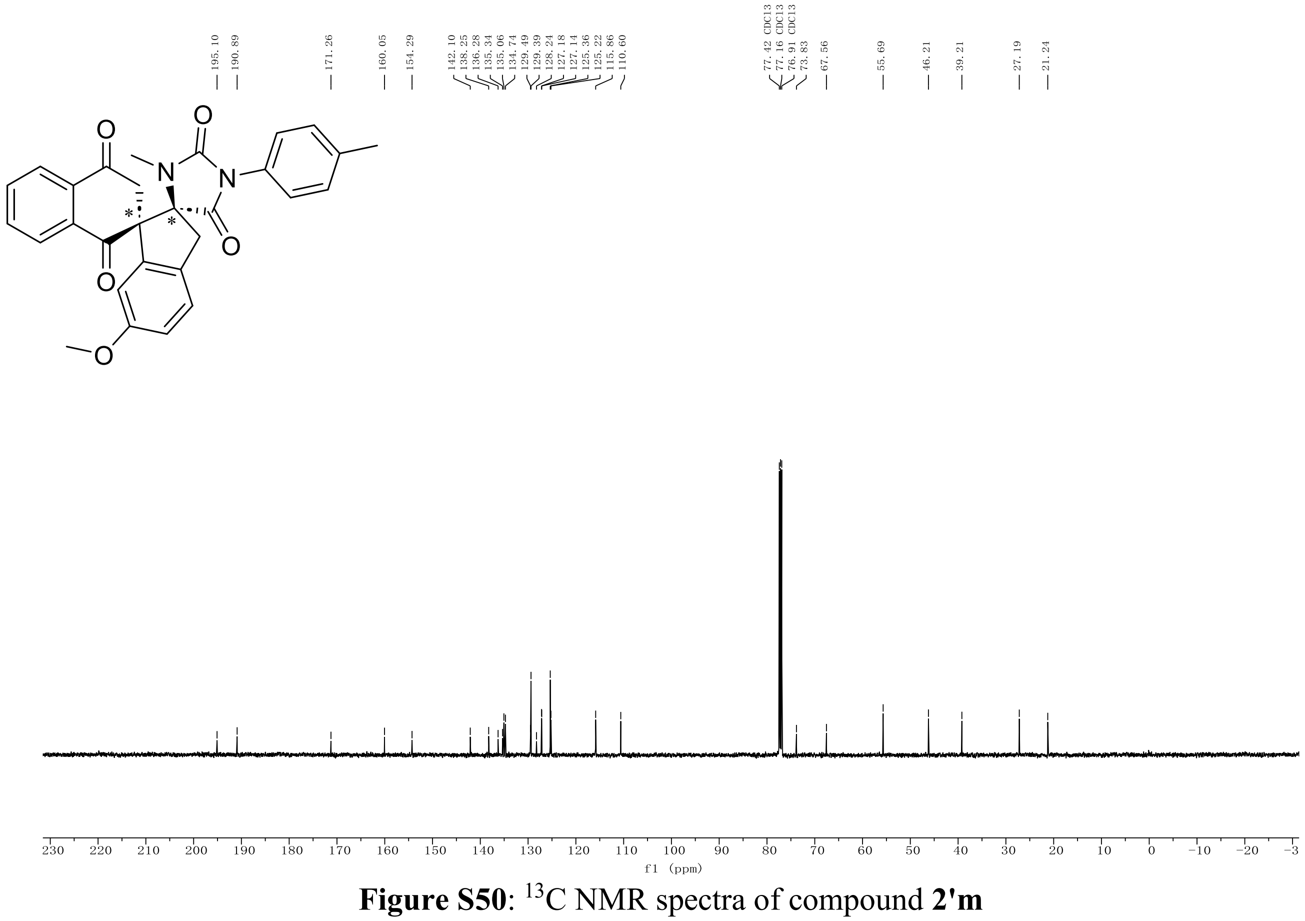

Figure S50: ^{13}C NMR spectra of compound **2'm**

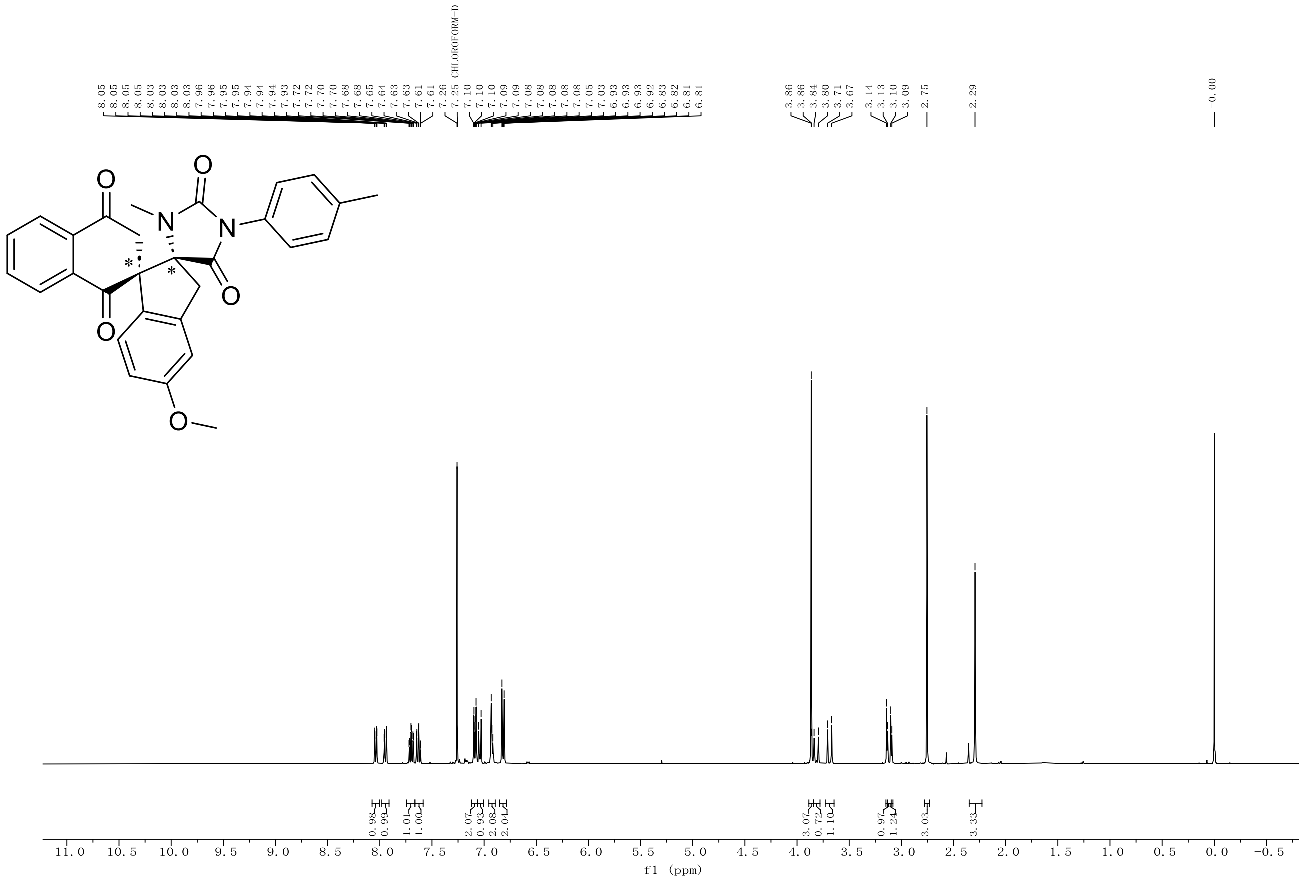

Figure S51: ^{1}H NMR spectra of compound **2n**

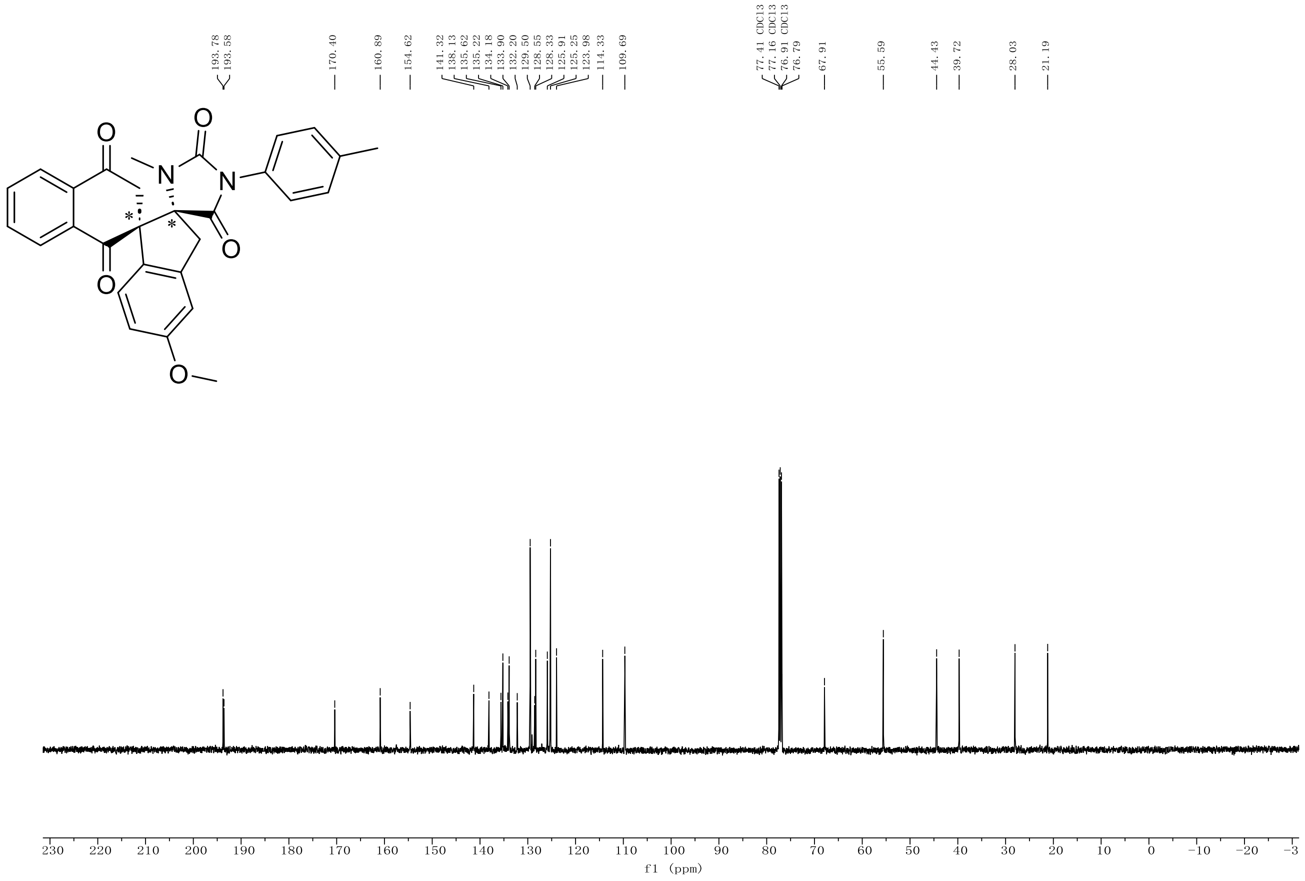

Figure S52: ^{13}C NMR spectra of compound **2n**

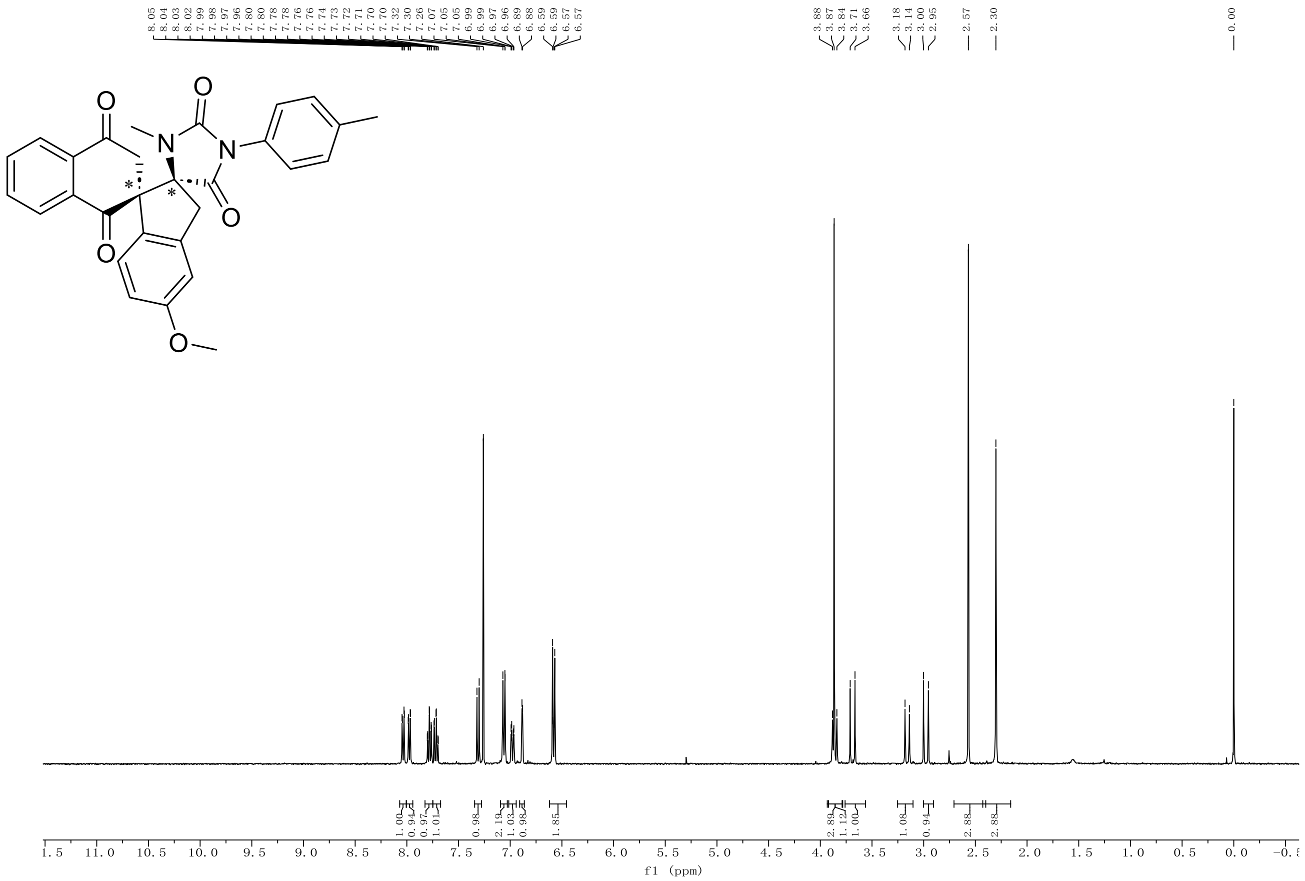

Figure S53: ^{1}H NMR spectra of compound **2'n**

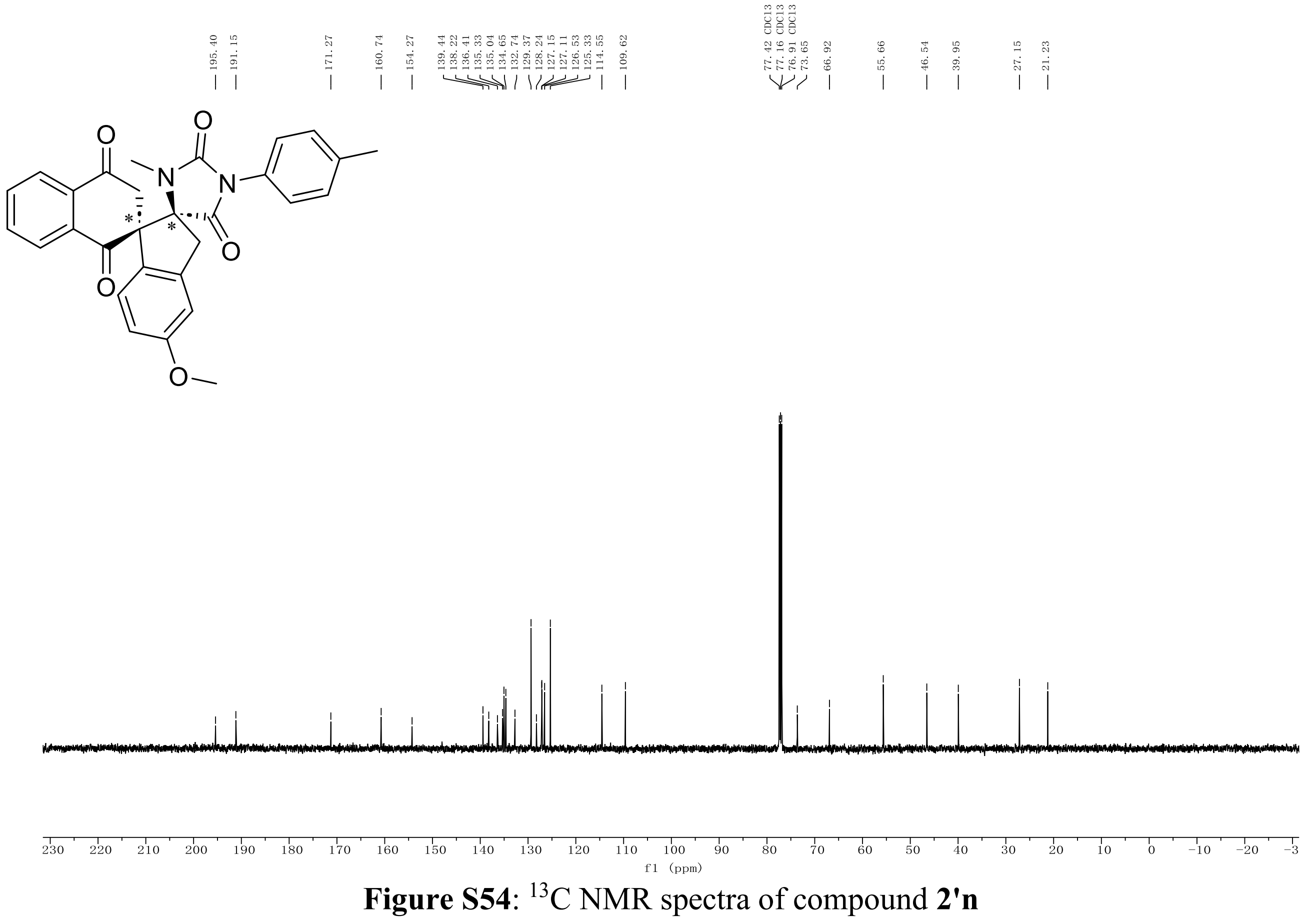

Figure S54: ^{13}C NMR spectra of compound **2'n**

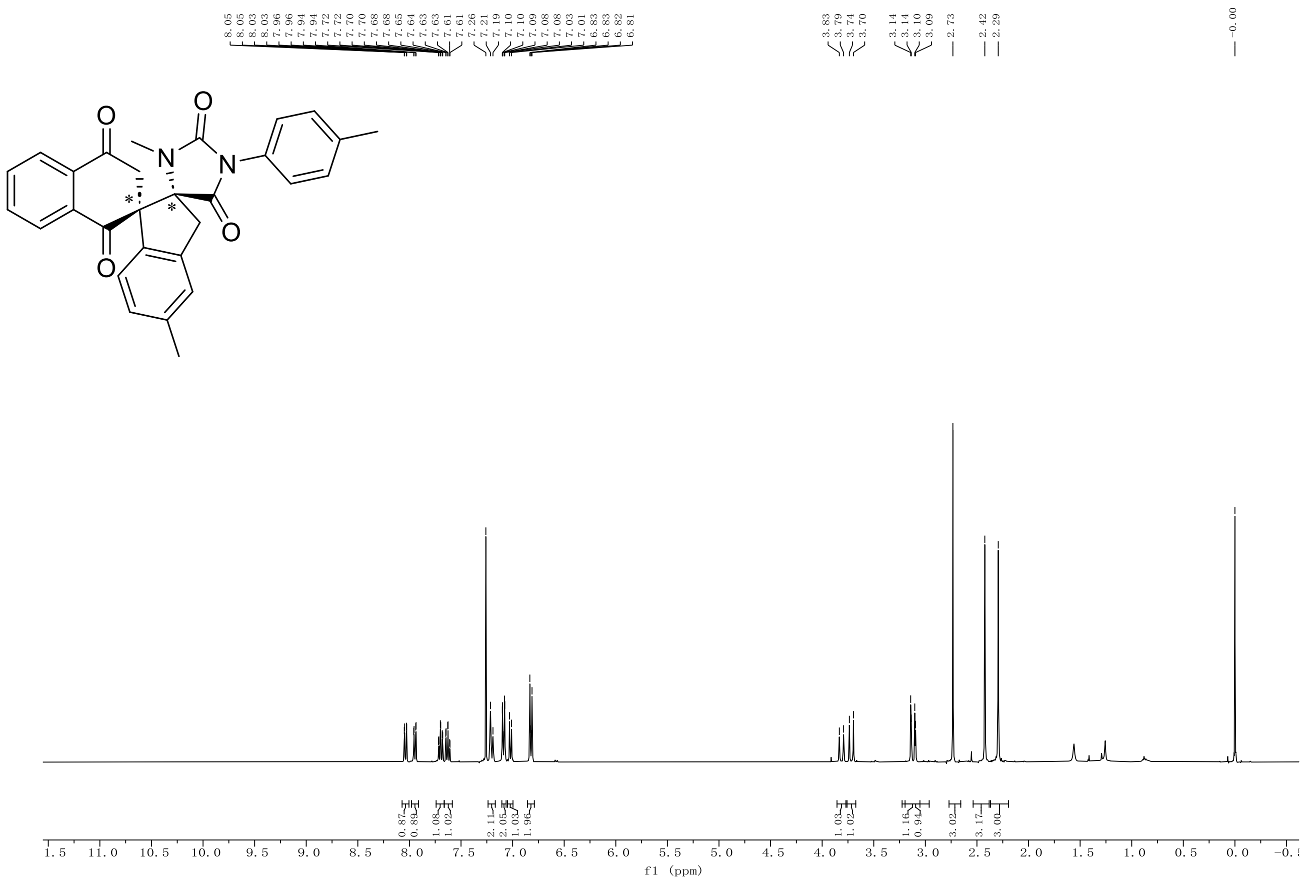

Figure S55: ^{1}H NMR spectra of compound **2o**

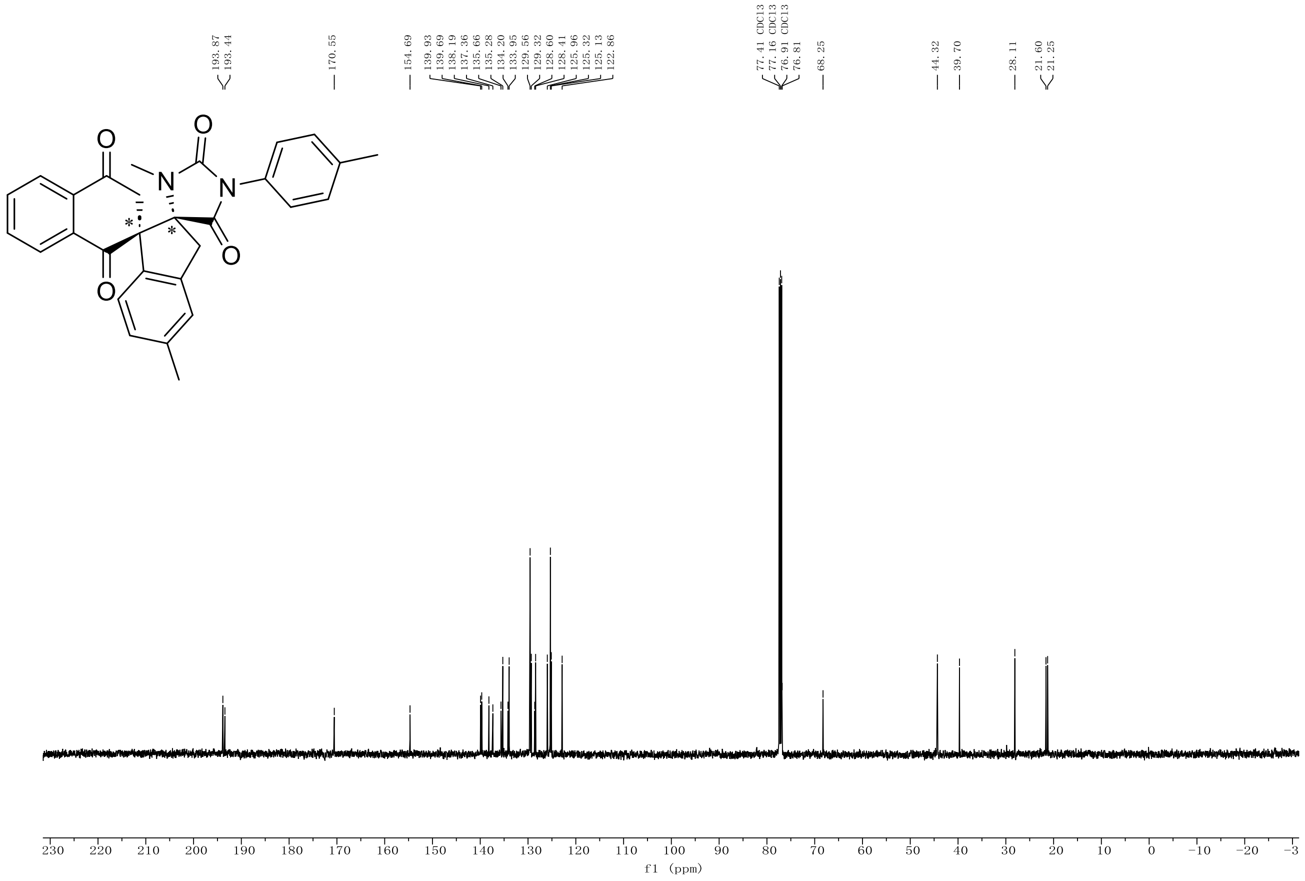

Figure S56: ^{13}C NMR spectra of compound **2o**

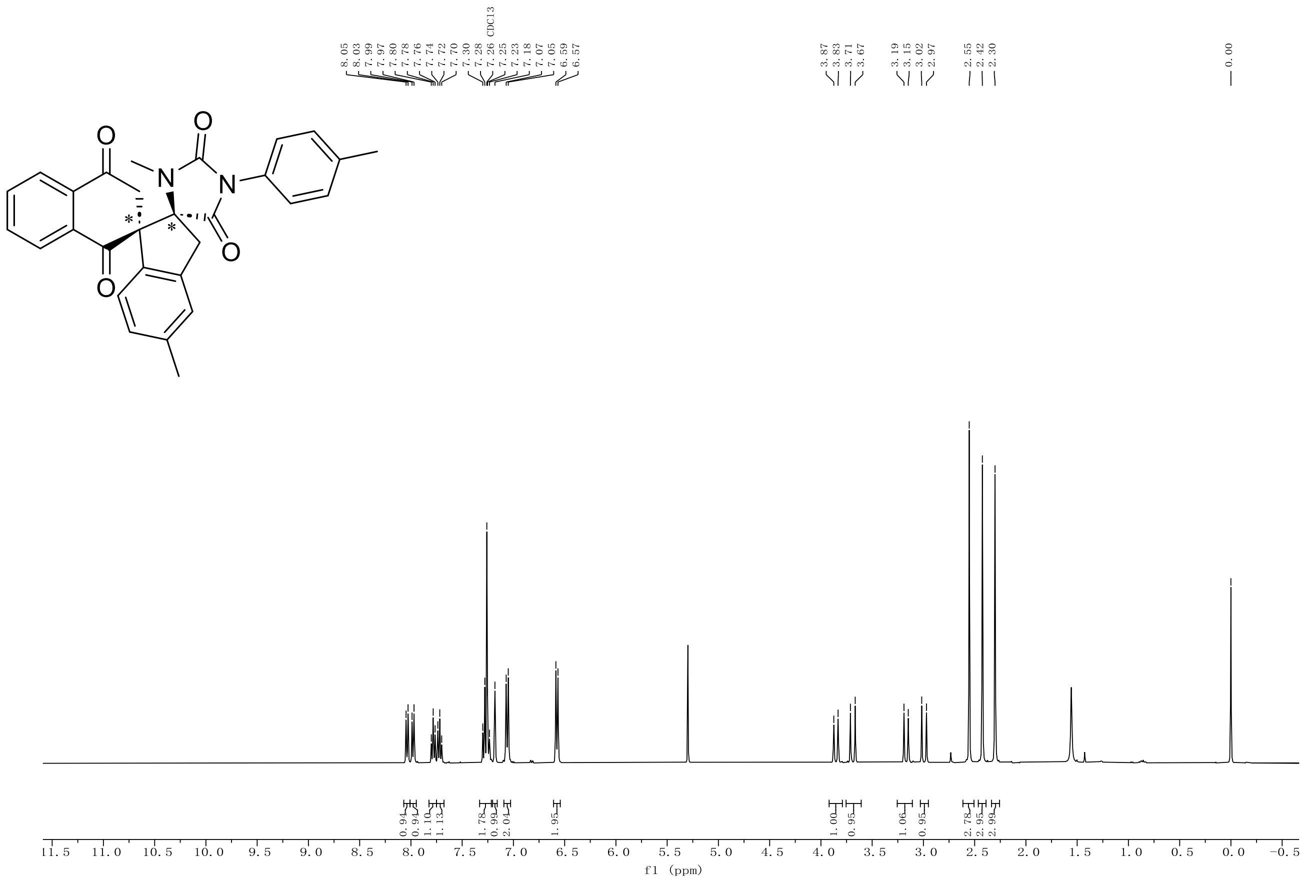

Figure S57: ^{1}H NMR spectra of compound **2'o**

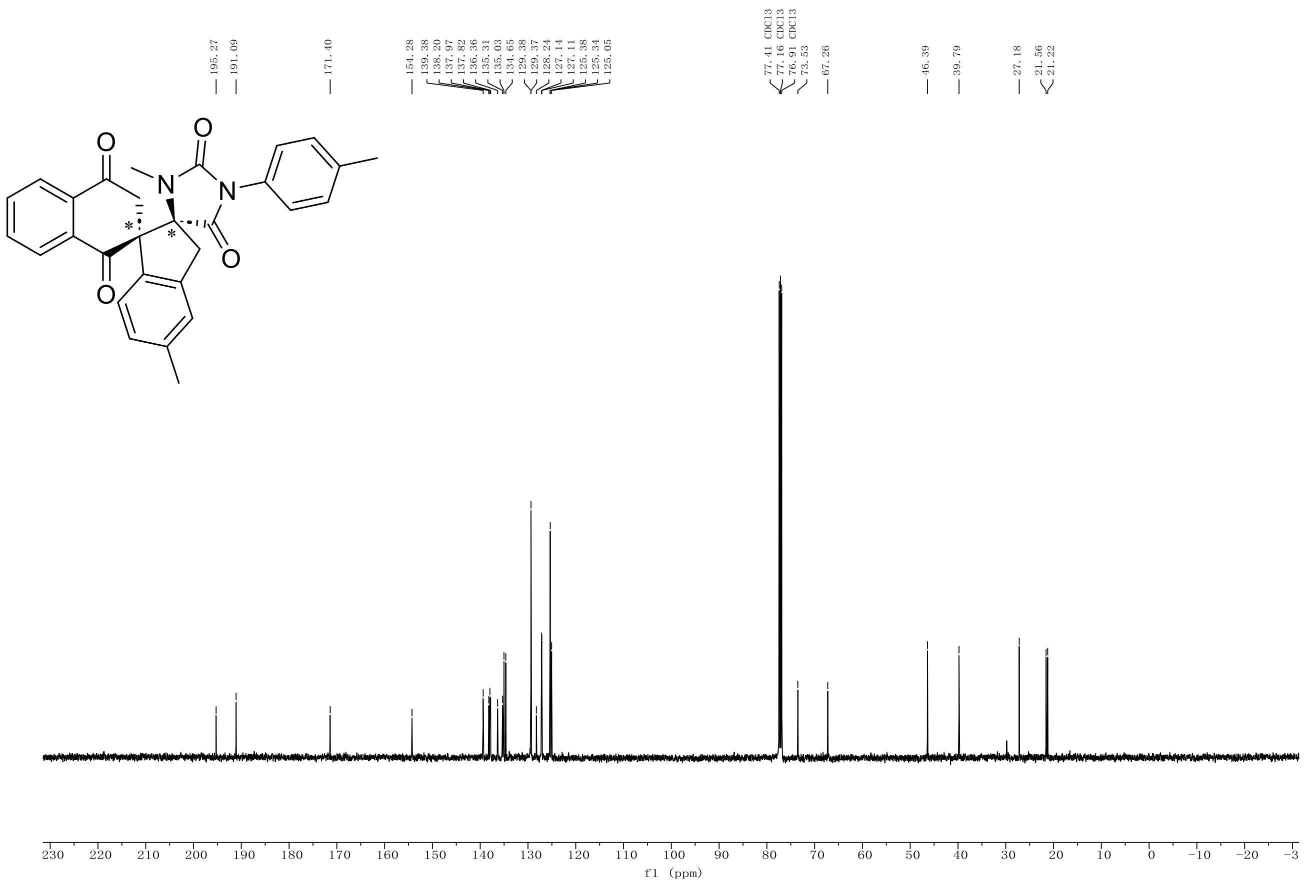

Figure S58: ^{13}C NMR spectra of compound **2'o**

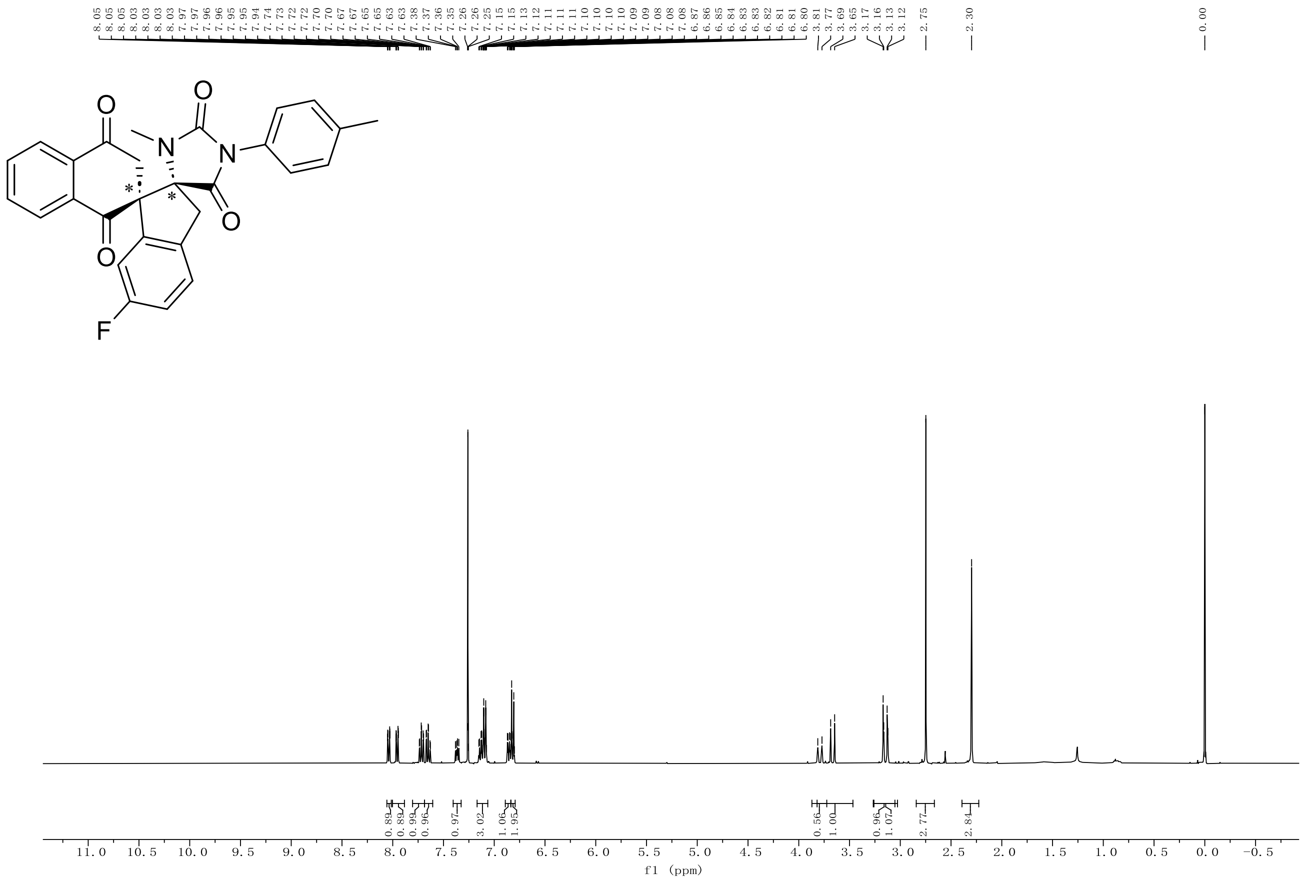

Figure S59: ^{1}H NMR spectra of compound **2p**

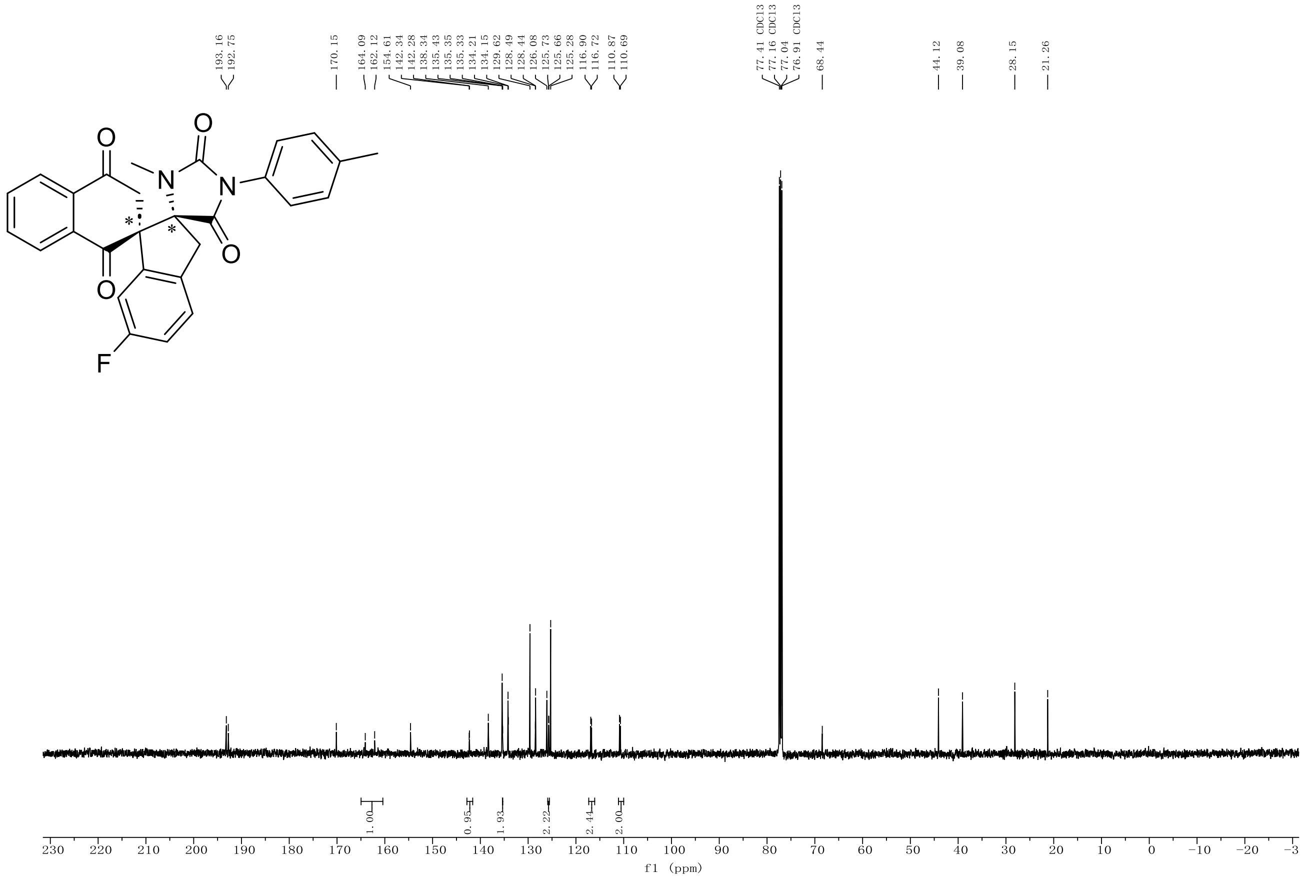

Figure S60: ^{13}C NMR spectra of compound **2p**

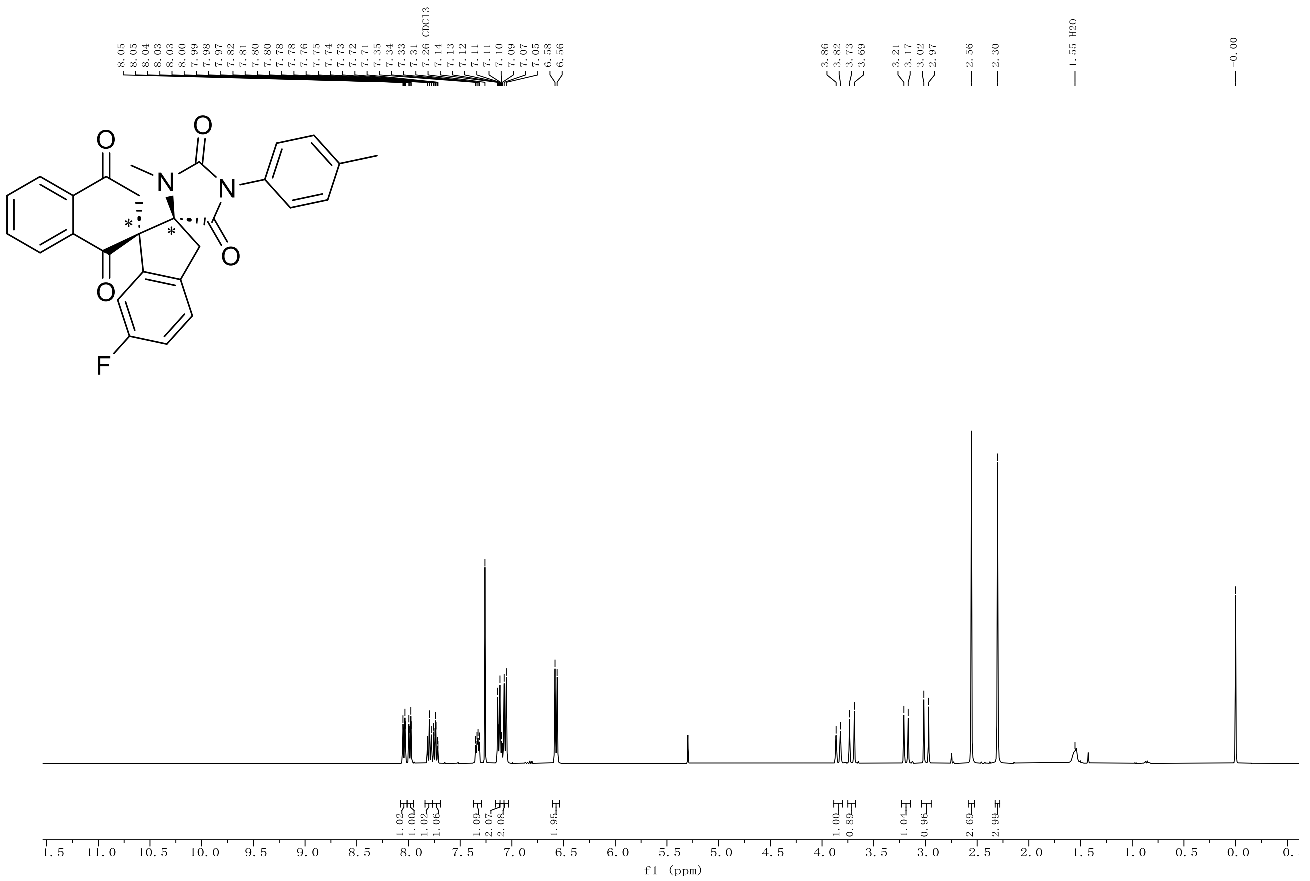

Figure S61: ^{1}H NMR spectra of compound **2'p**

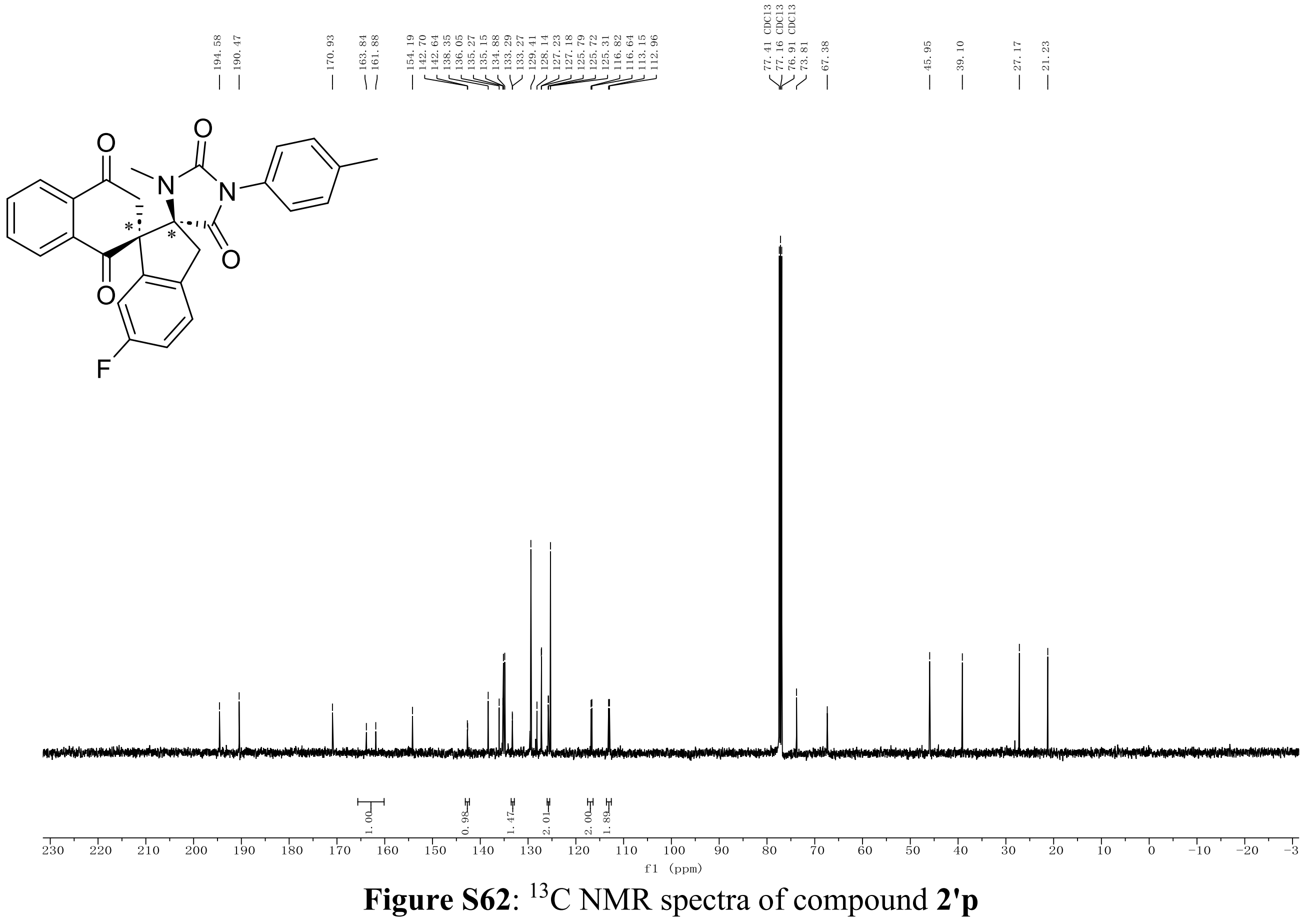

Figure S62: ^{13}C NMR spectra of compound **2'p**

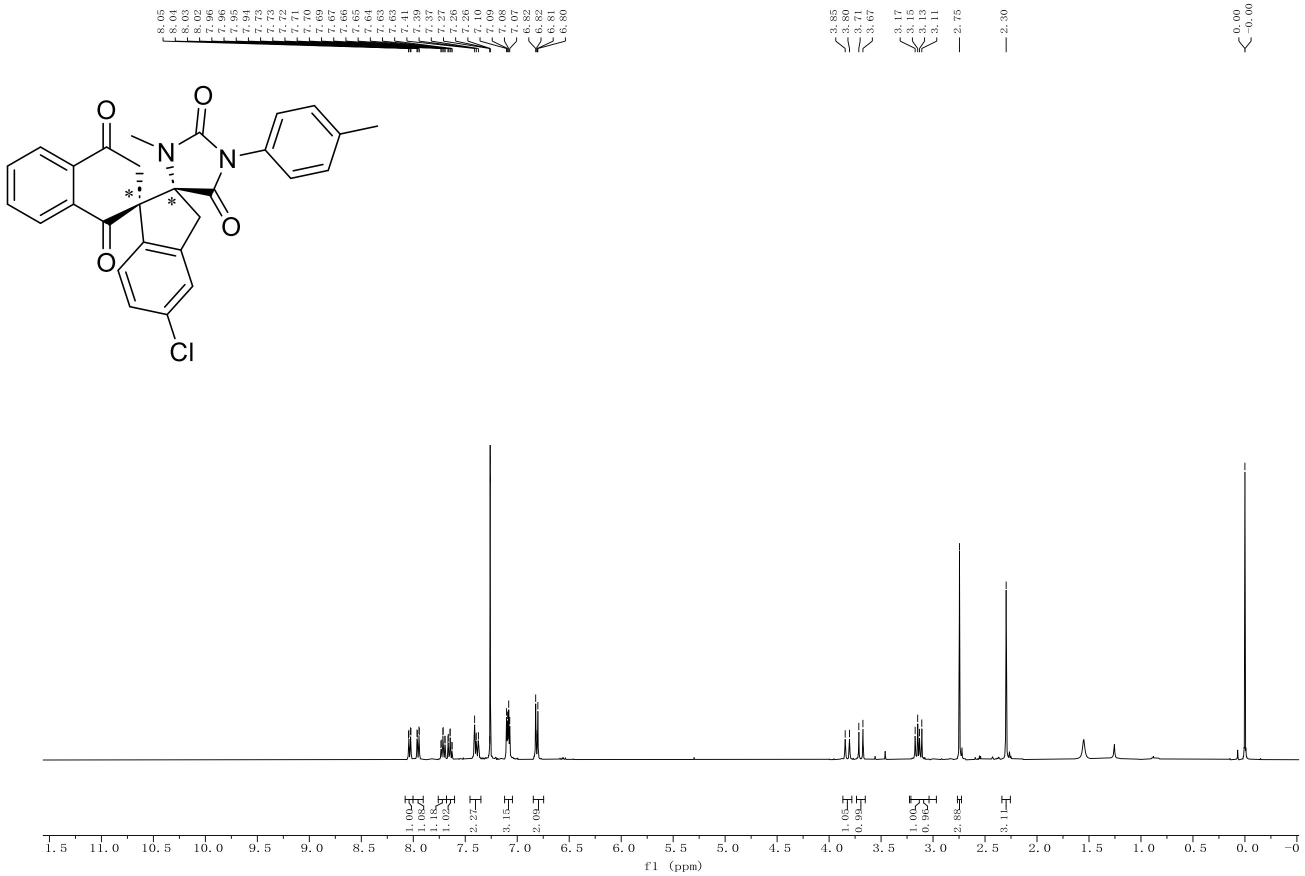

Figure S63: ^{1}H NMR spectra of compound **2q**

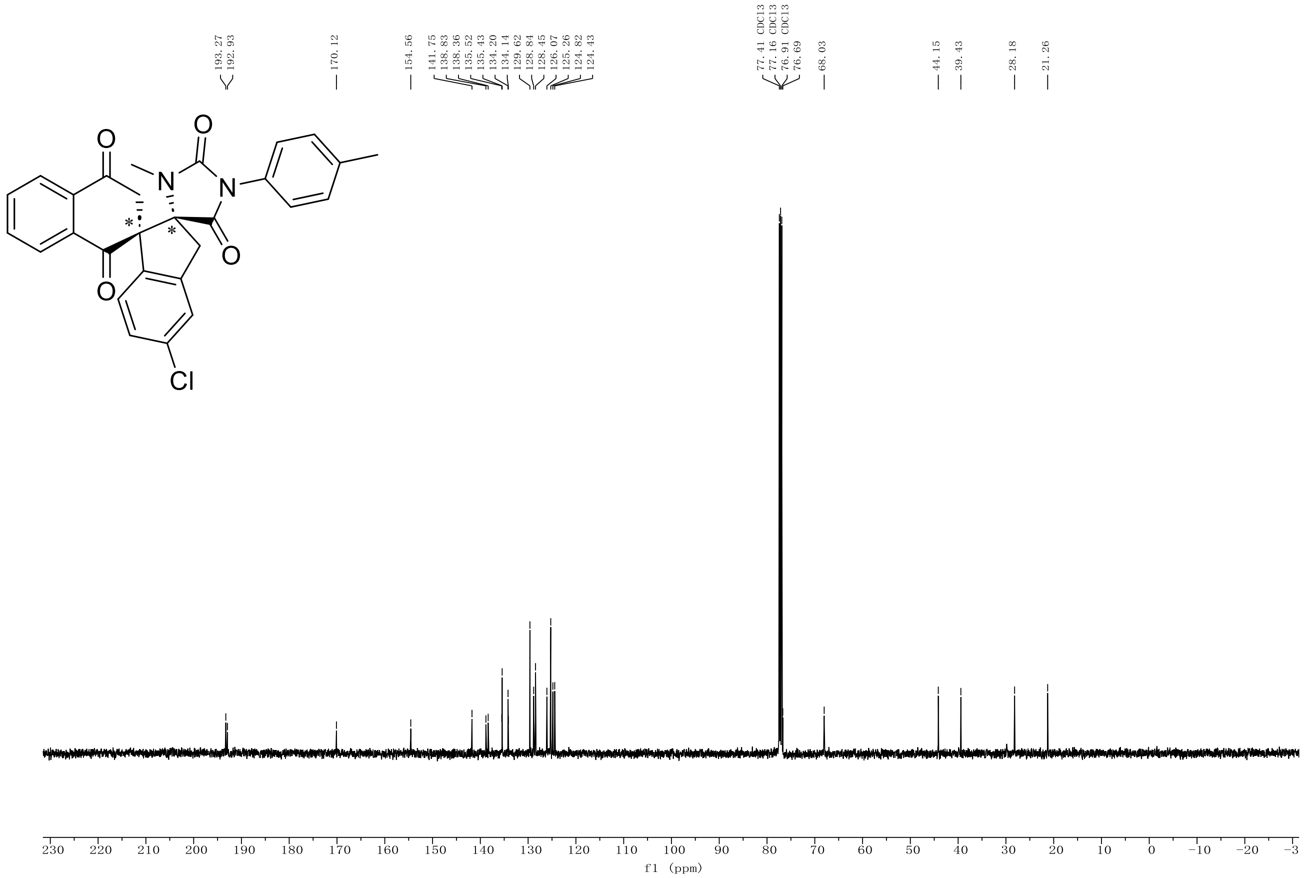

Figure S64: ^{13}C NMR spectra of compound **2q**

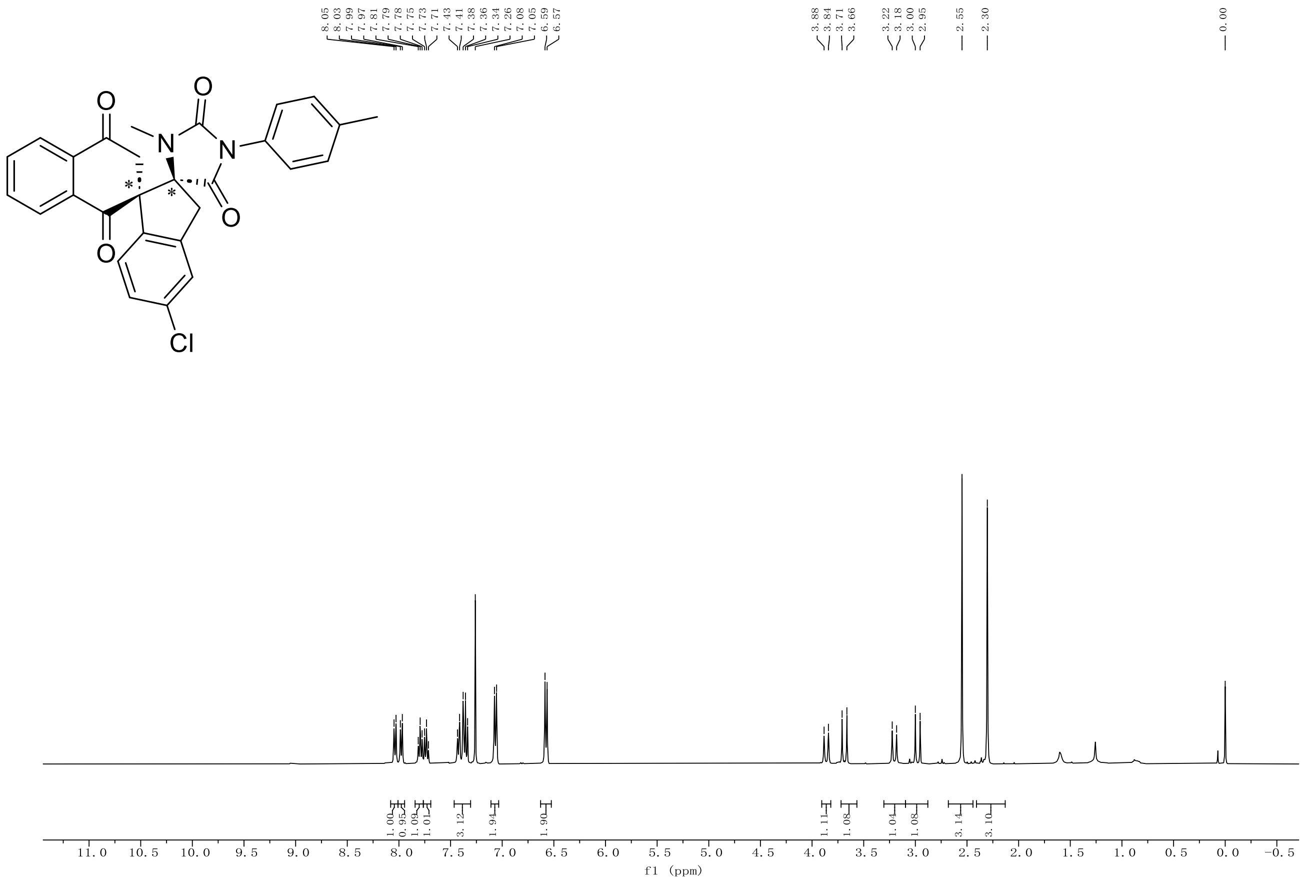

Figure S65: ^{1}H NMR spectra of compound **2'q**

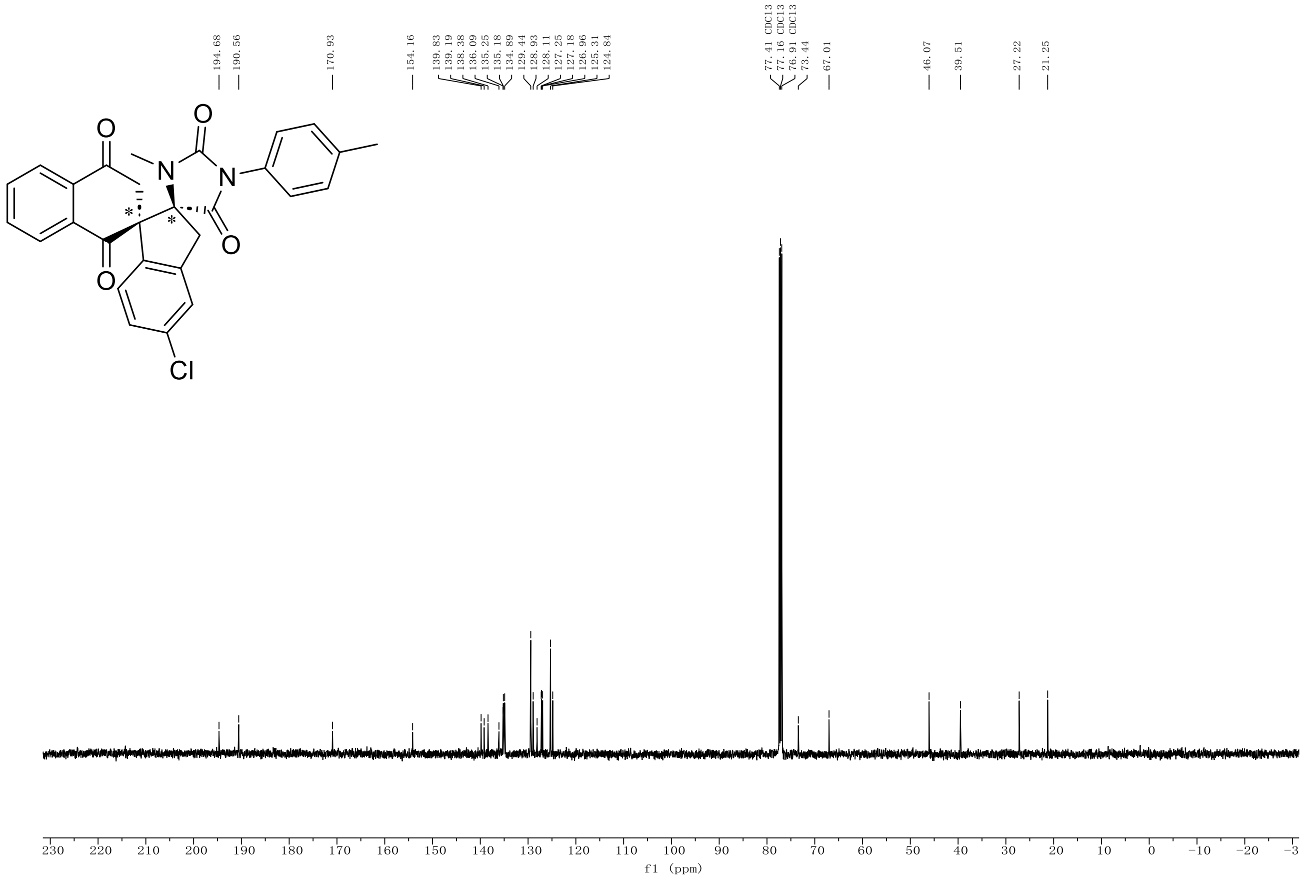

Figure S66: ^{13}C NMR spectra of compound **2'q**

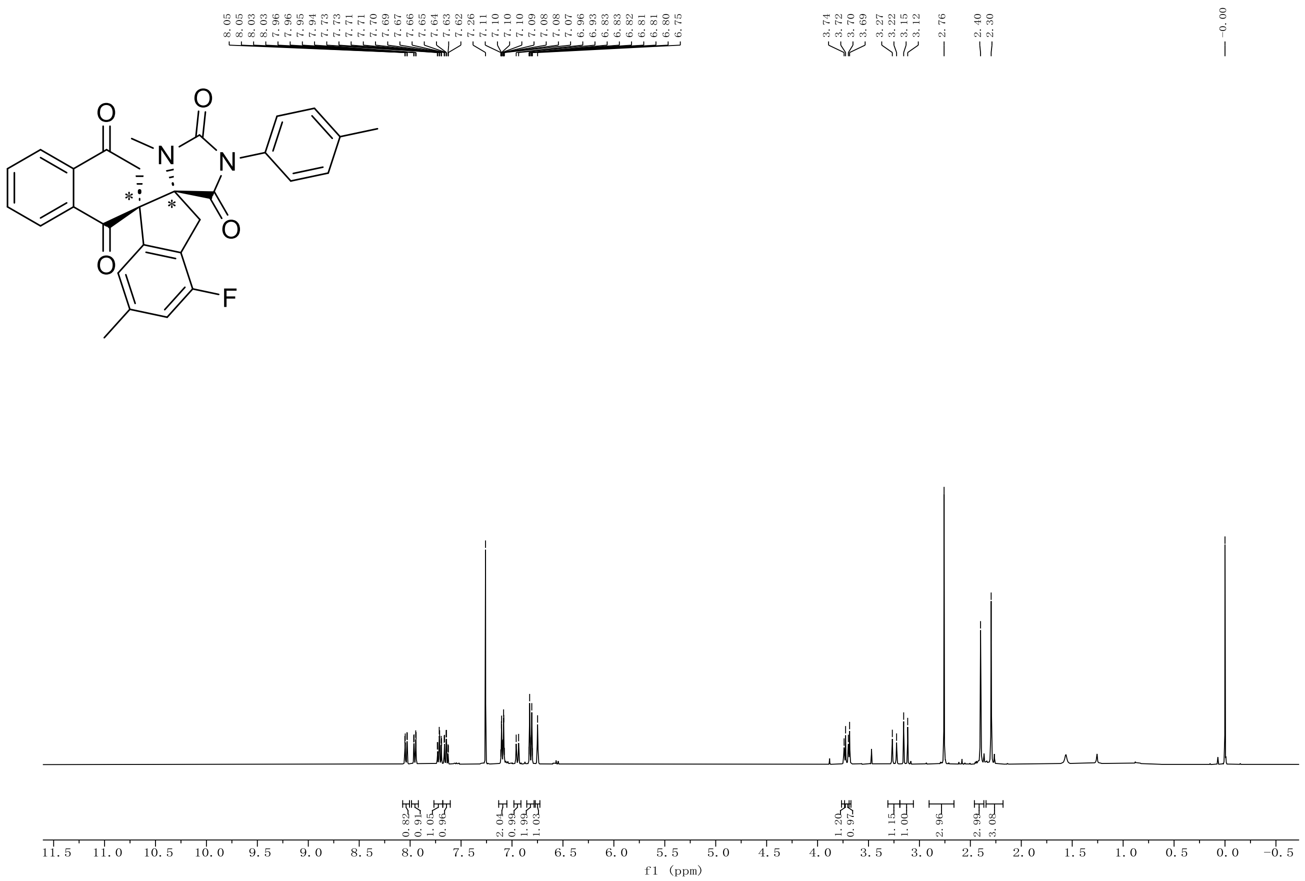

Figure S67: ^{1}H NMR spectra of compound **2r**

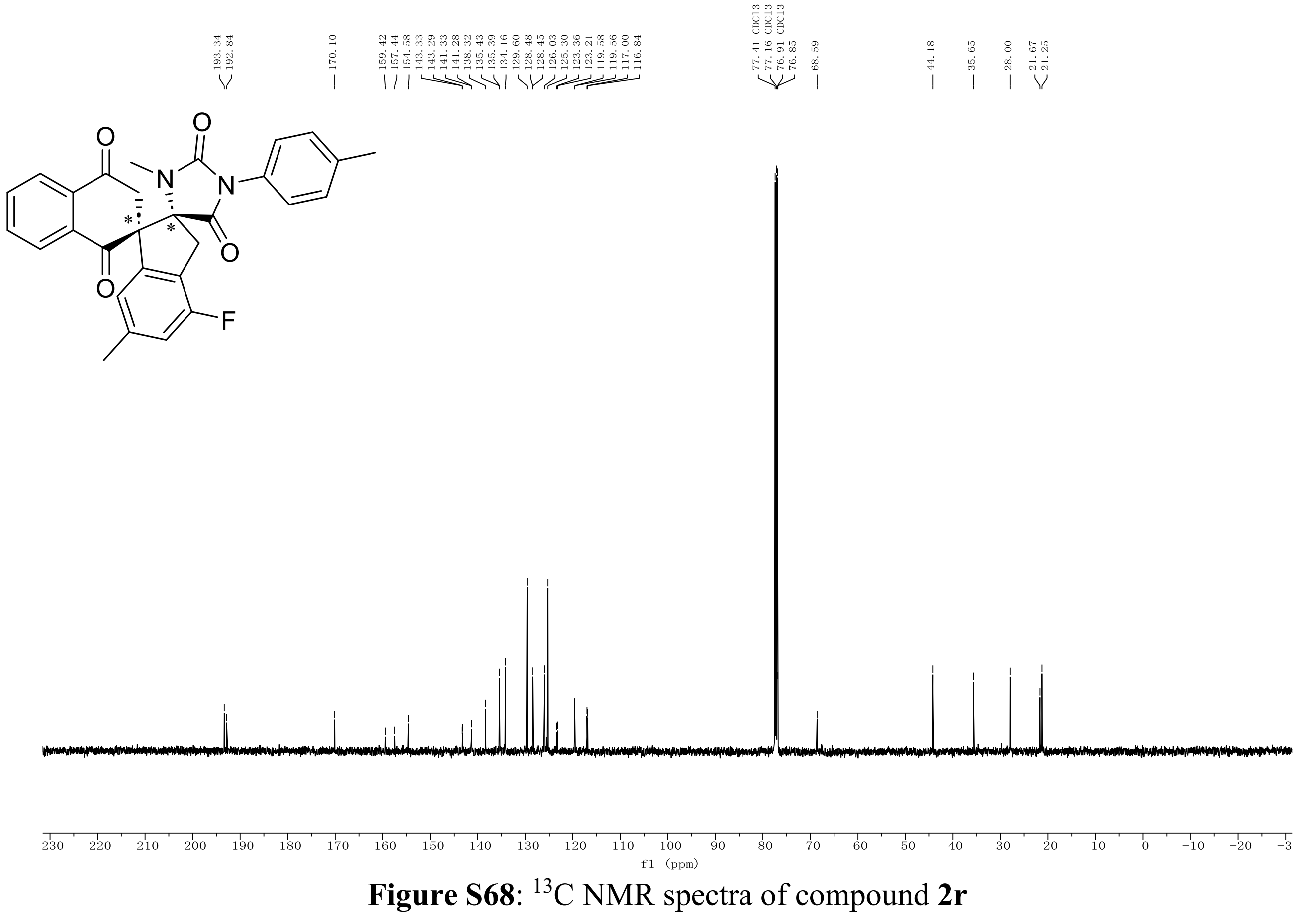

Figure S68: ^{13}C NMR spectra of compound **2r**

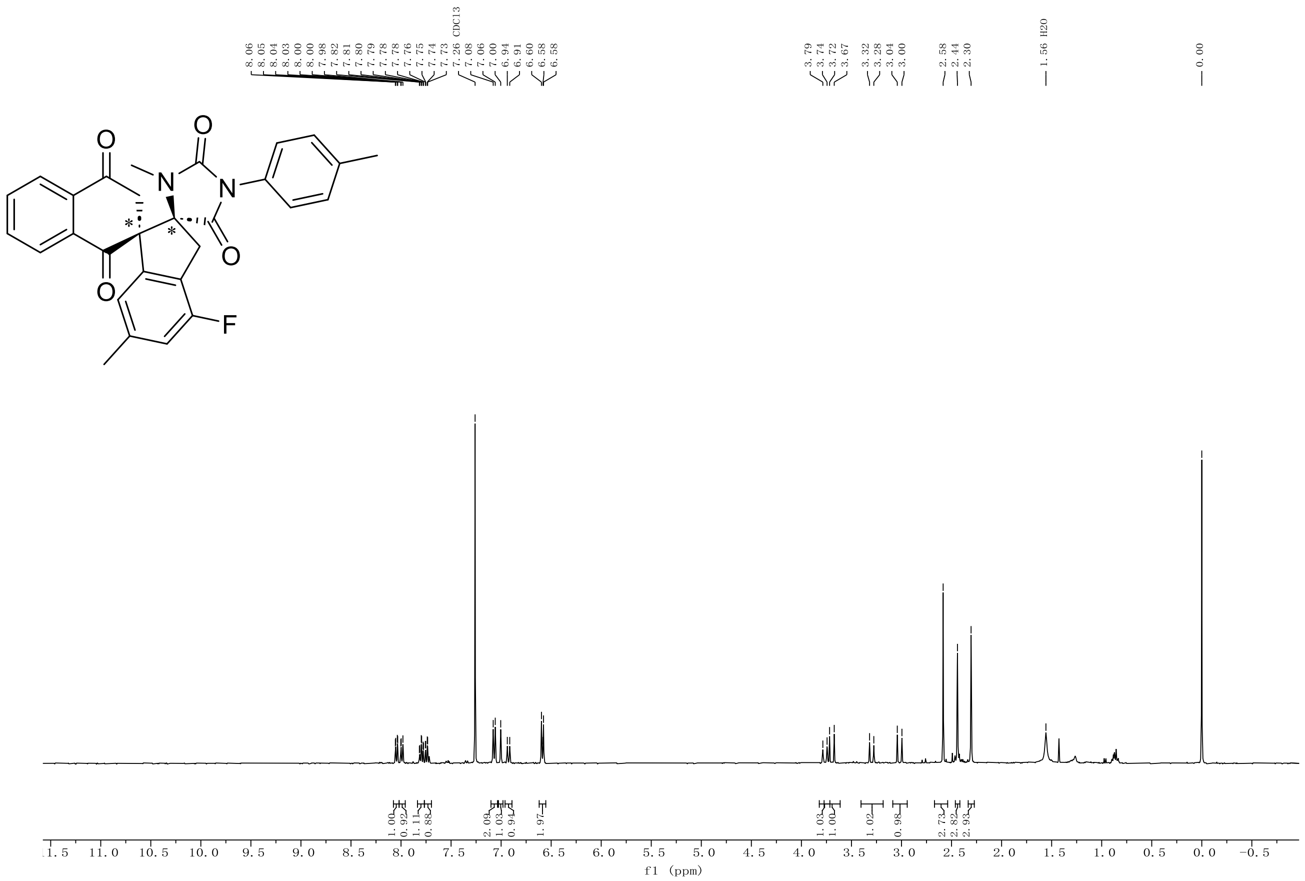

Figure S69: ^{1}H NMR spectra of compound **2'r**

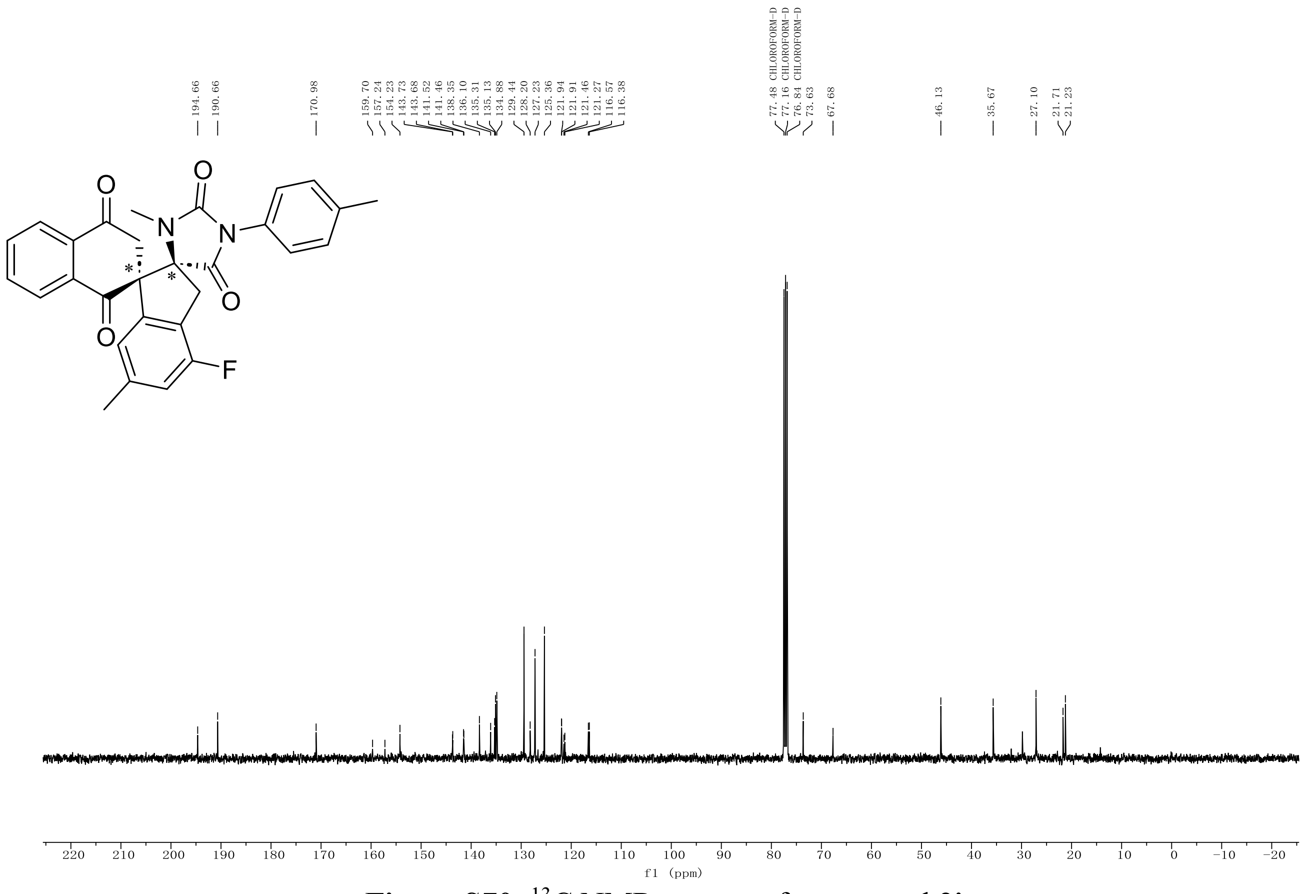

Figure S70: ^{13}C NMR spectra of compound **2'r**

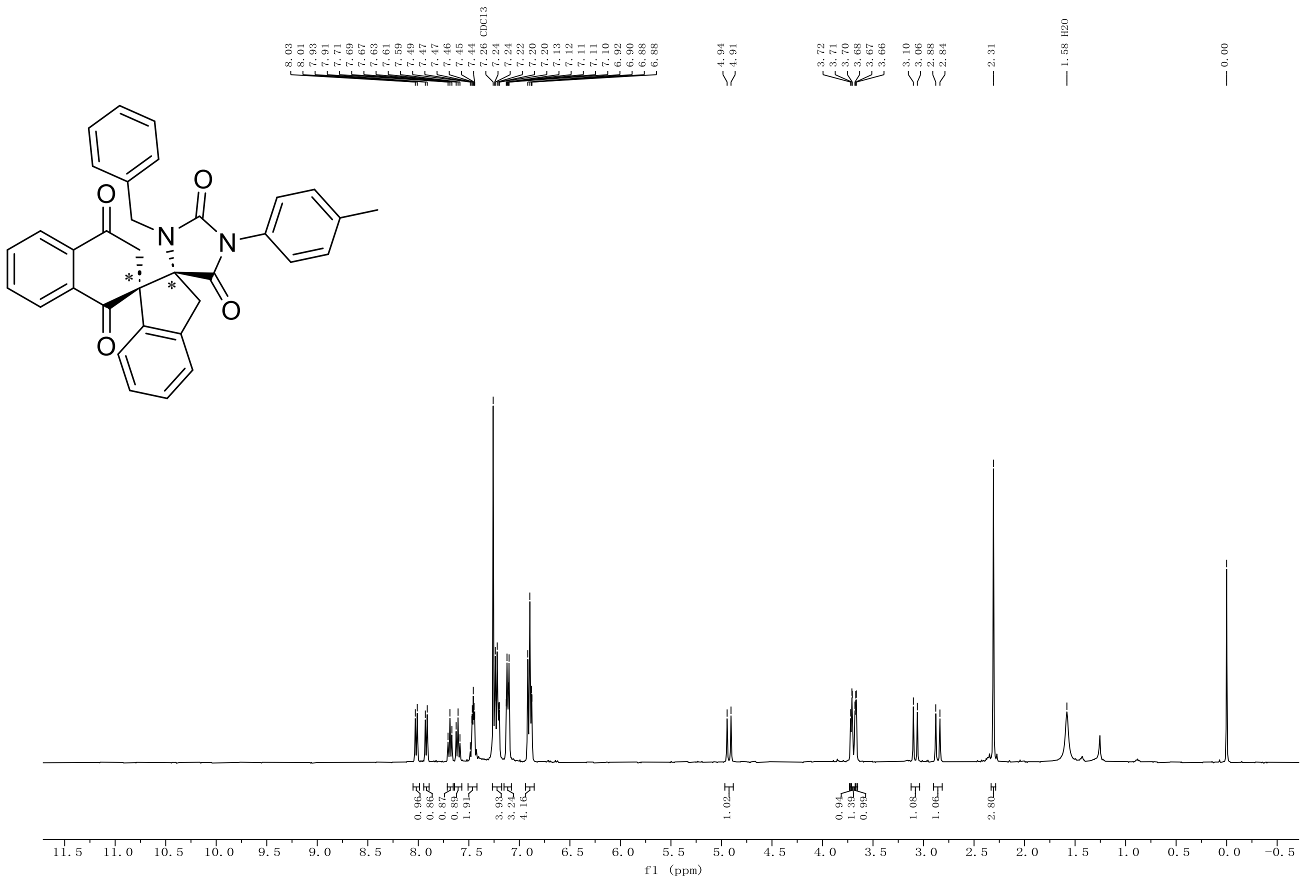

Figure S71: ^{1}H NMR spectra of compound **2s**

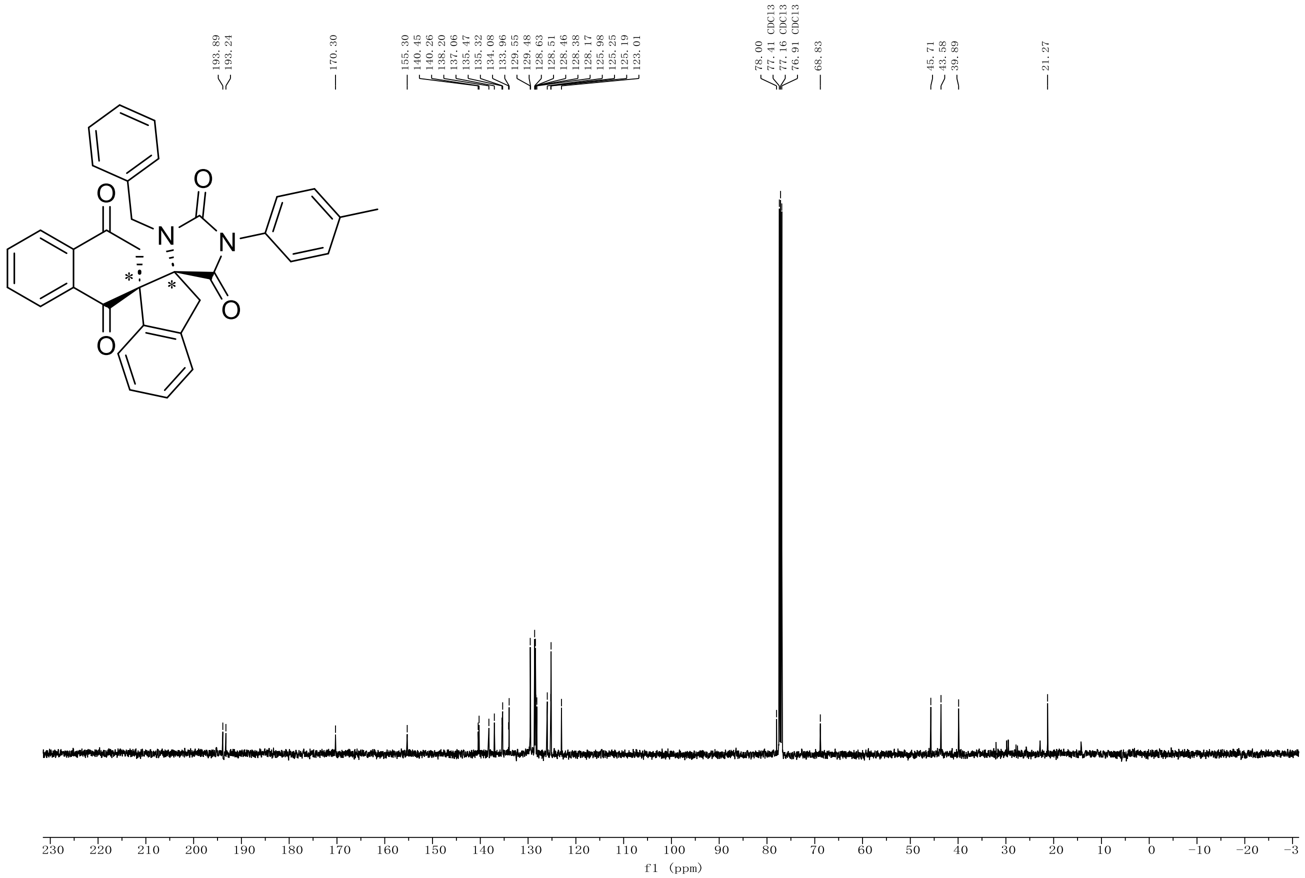

Figure S72: ^{13}C NMR spectra of compound **2s**

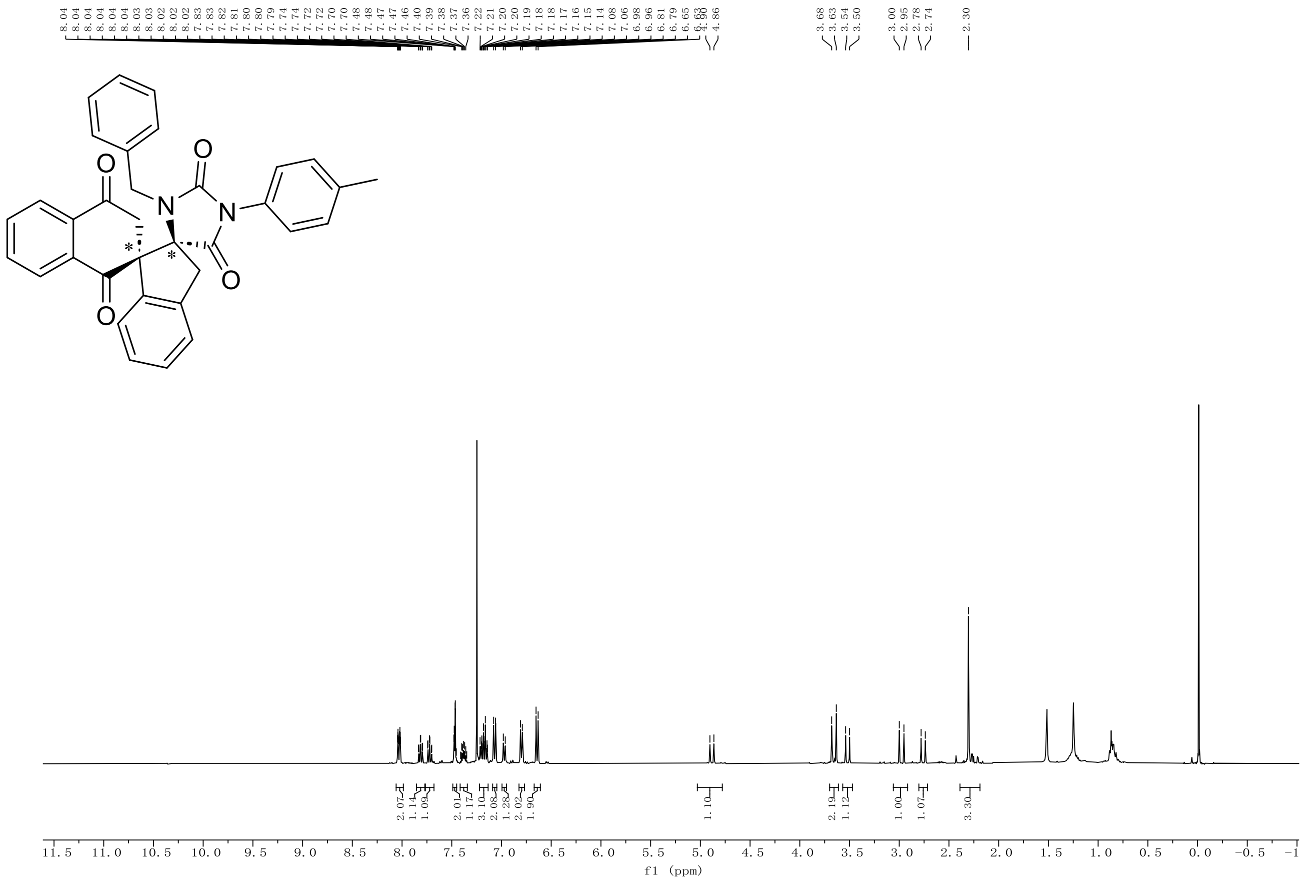

Figure S73: ^{1}H NMR spectra of compound **2's**

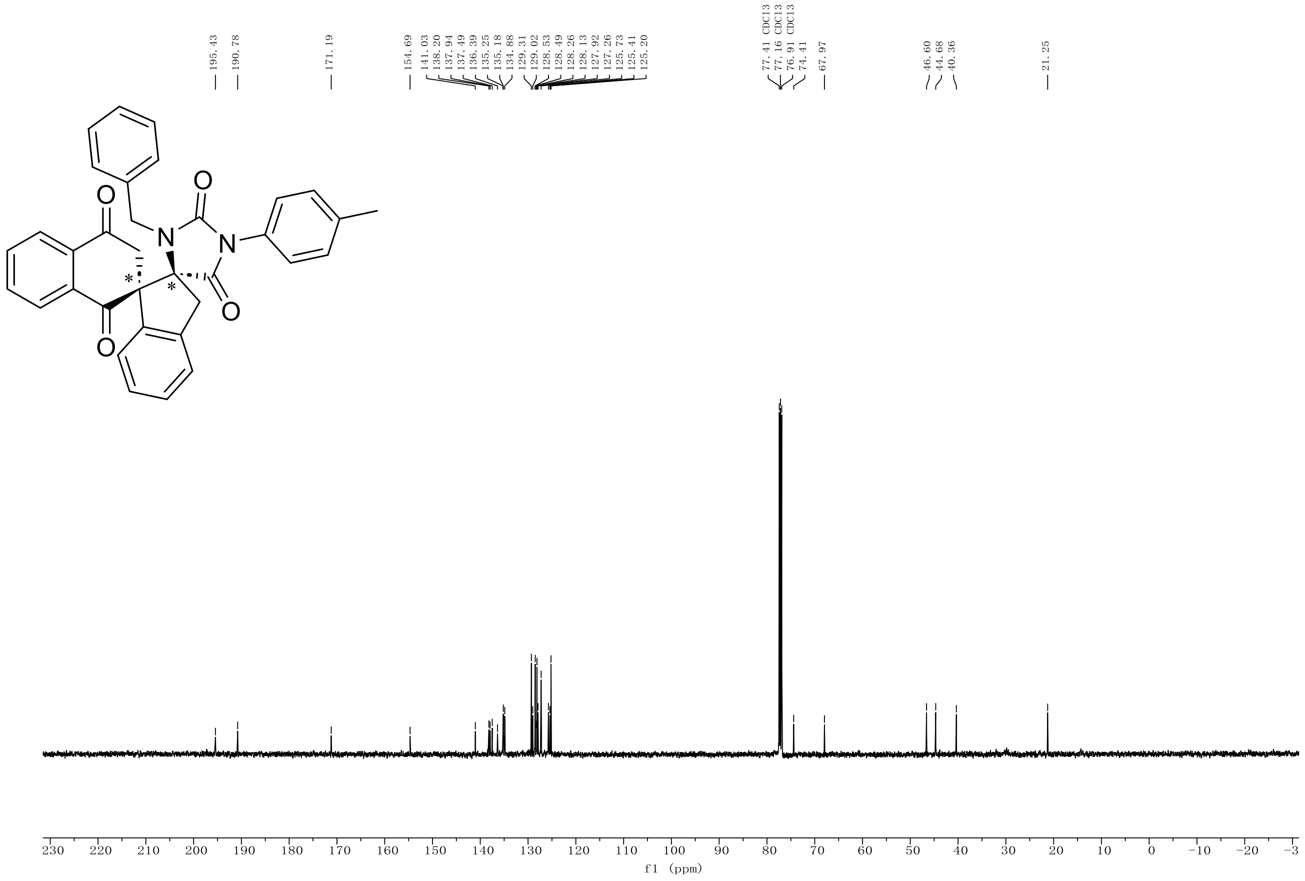

Figure S74: ^{13}C NMR spectra of compound **2's**

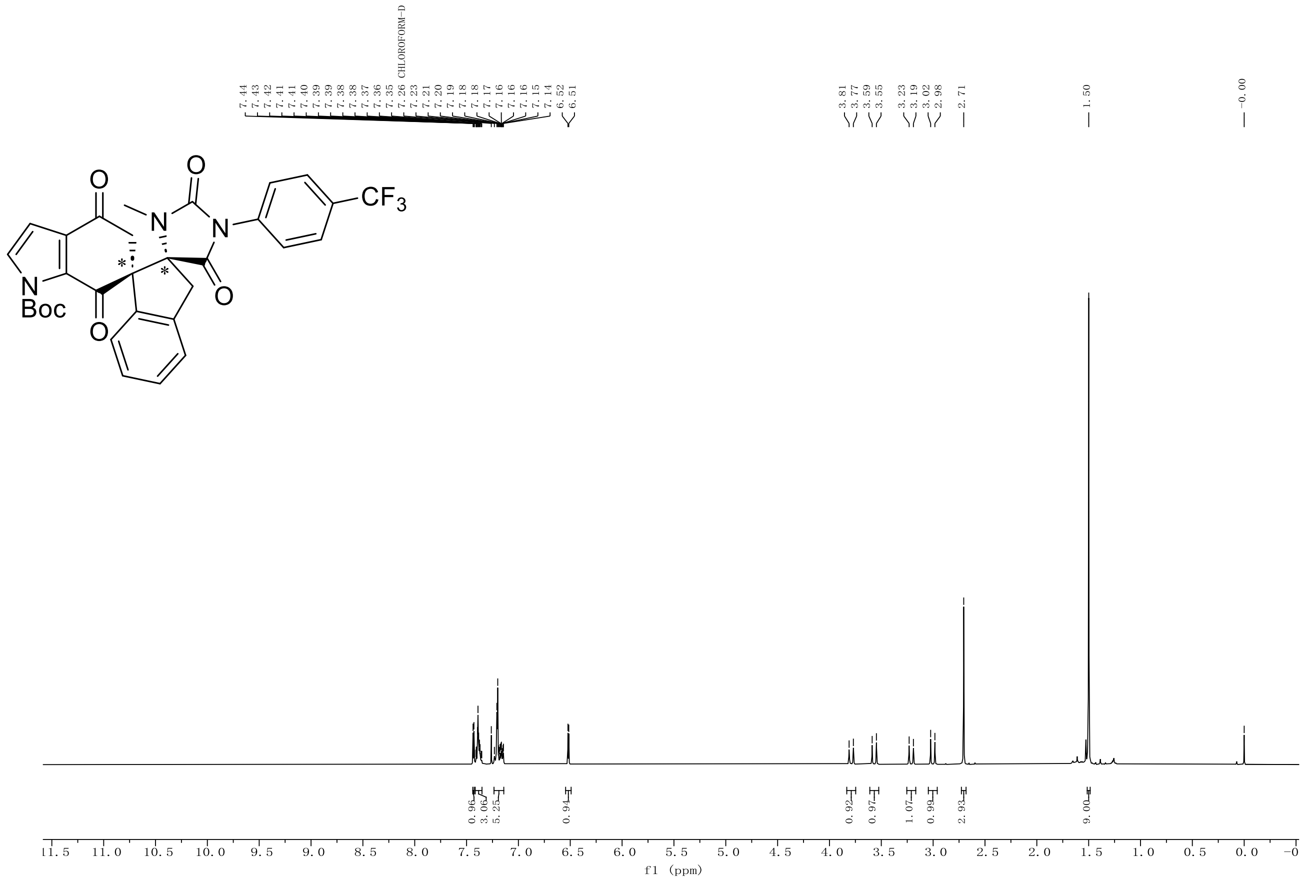

Figure S75: ^{1}H NMR spectra of compound **5a**

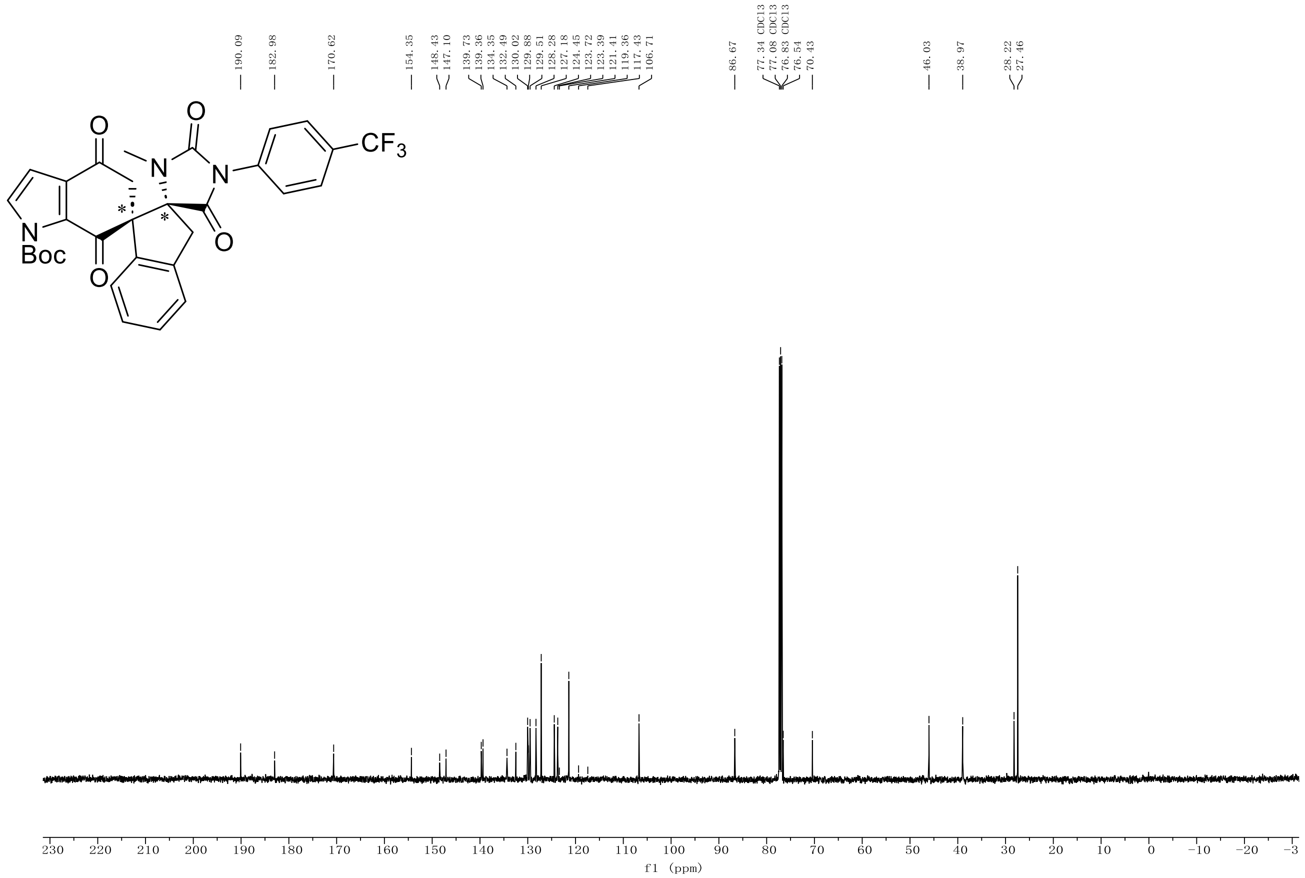

Figure S76: ^{13}C NMR spectra of compound **5a**

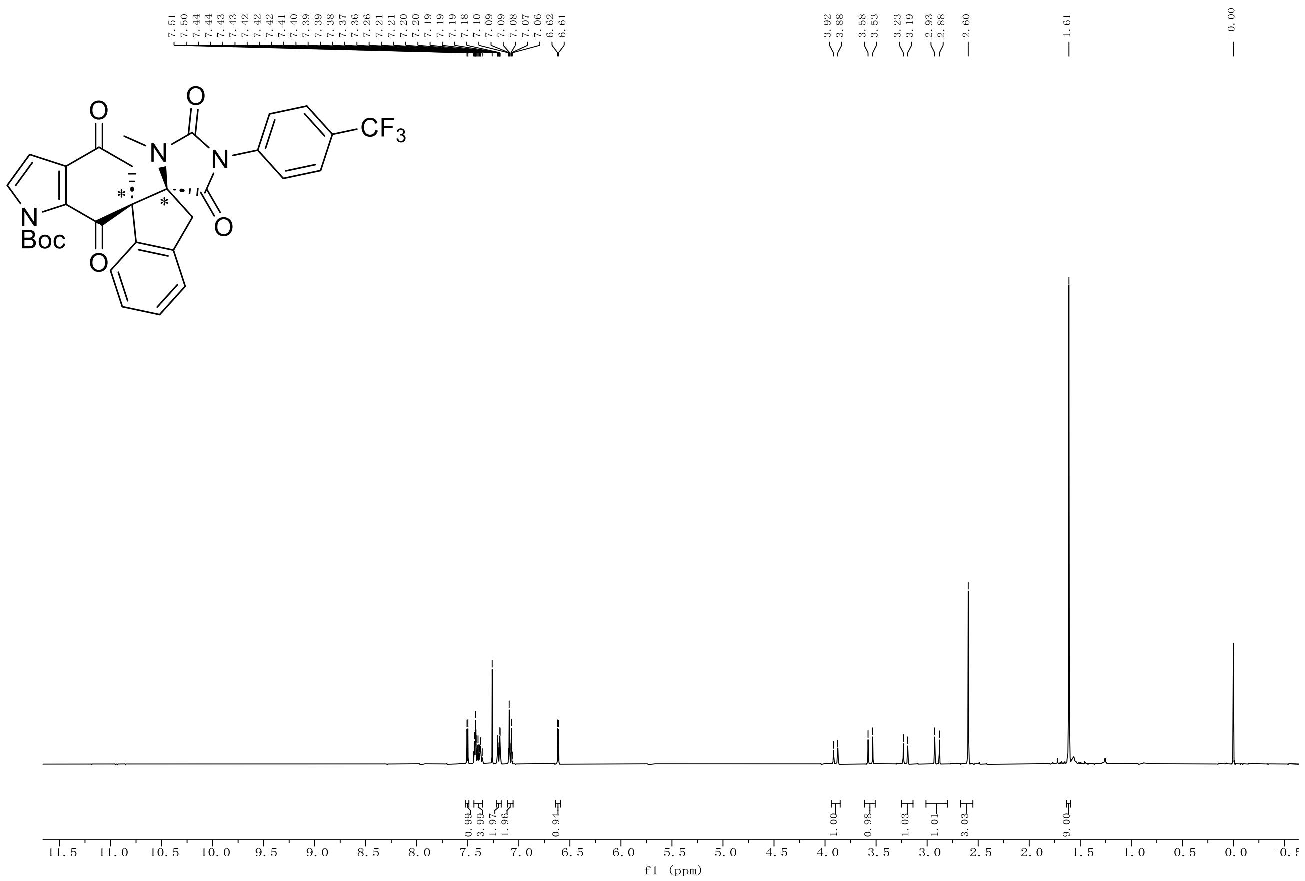

Figure S77: ^{1}H NMR spectra of compound **5'a**

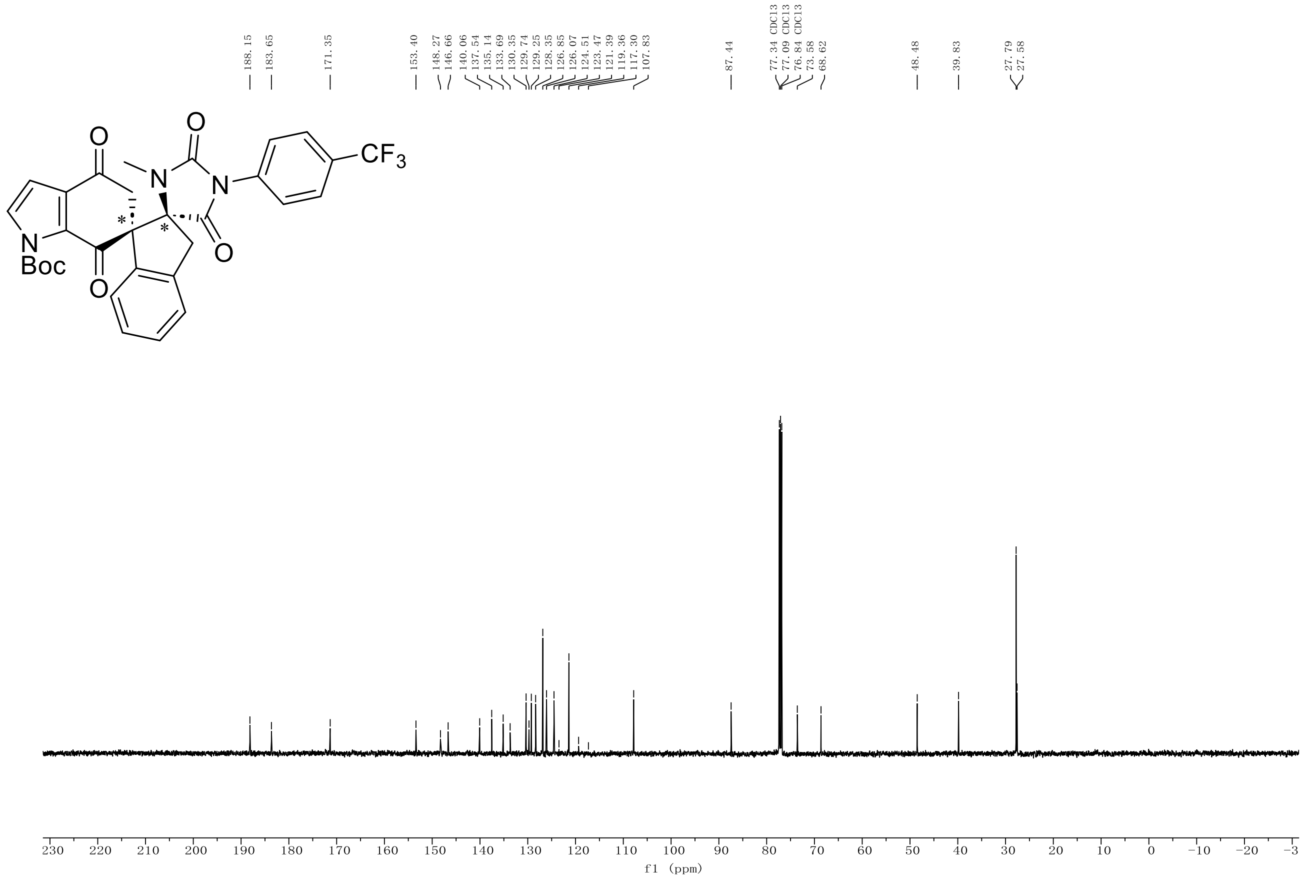

Figure S78: ^{13}C NMR spectra of compound **5'a**

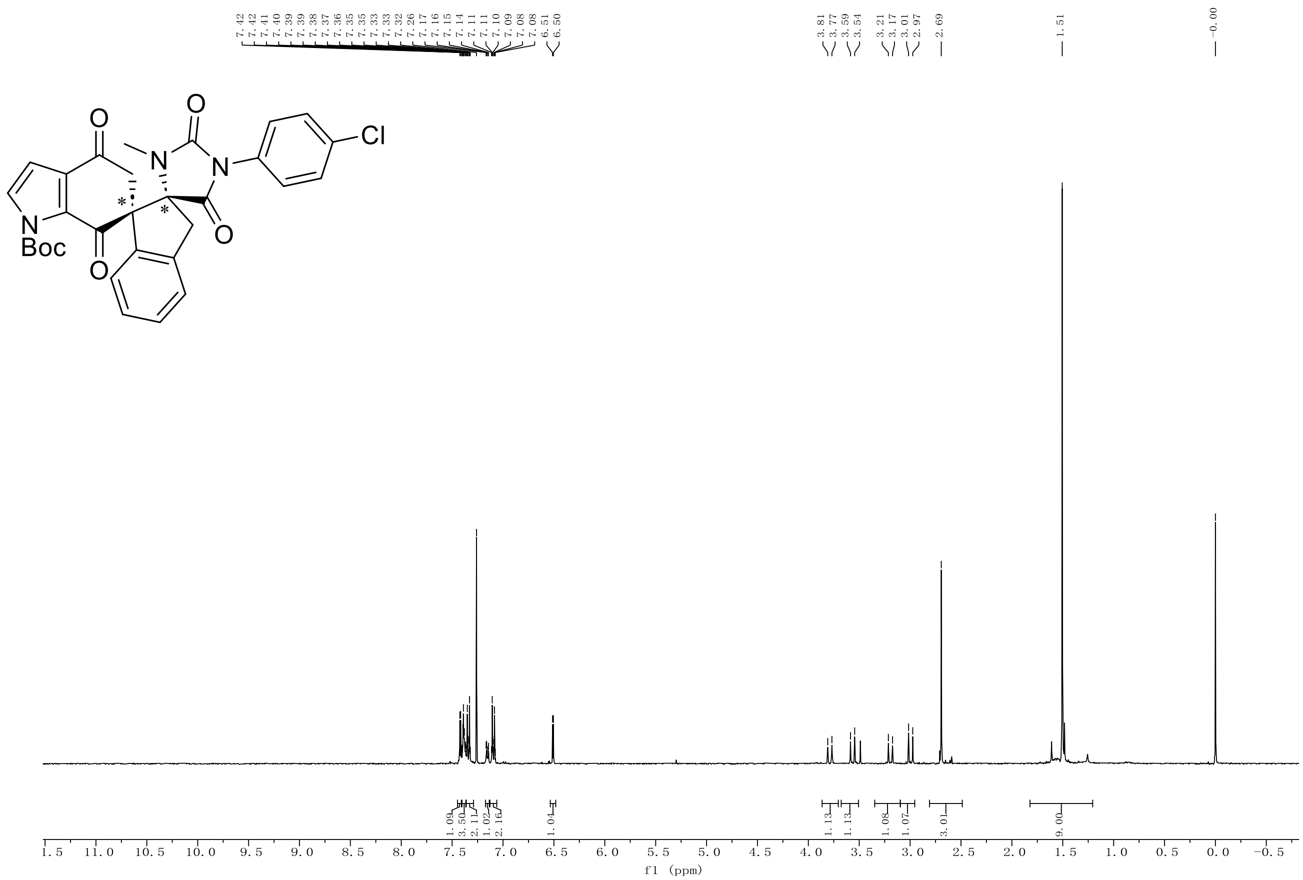

Figure S79: ^{1}H NMR spectra of compound **5b**

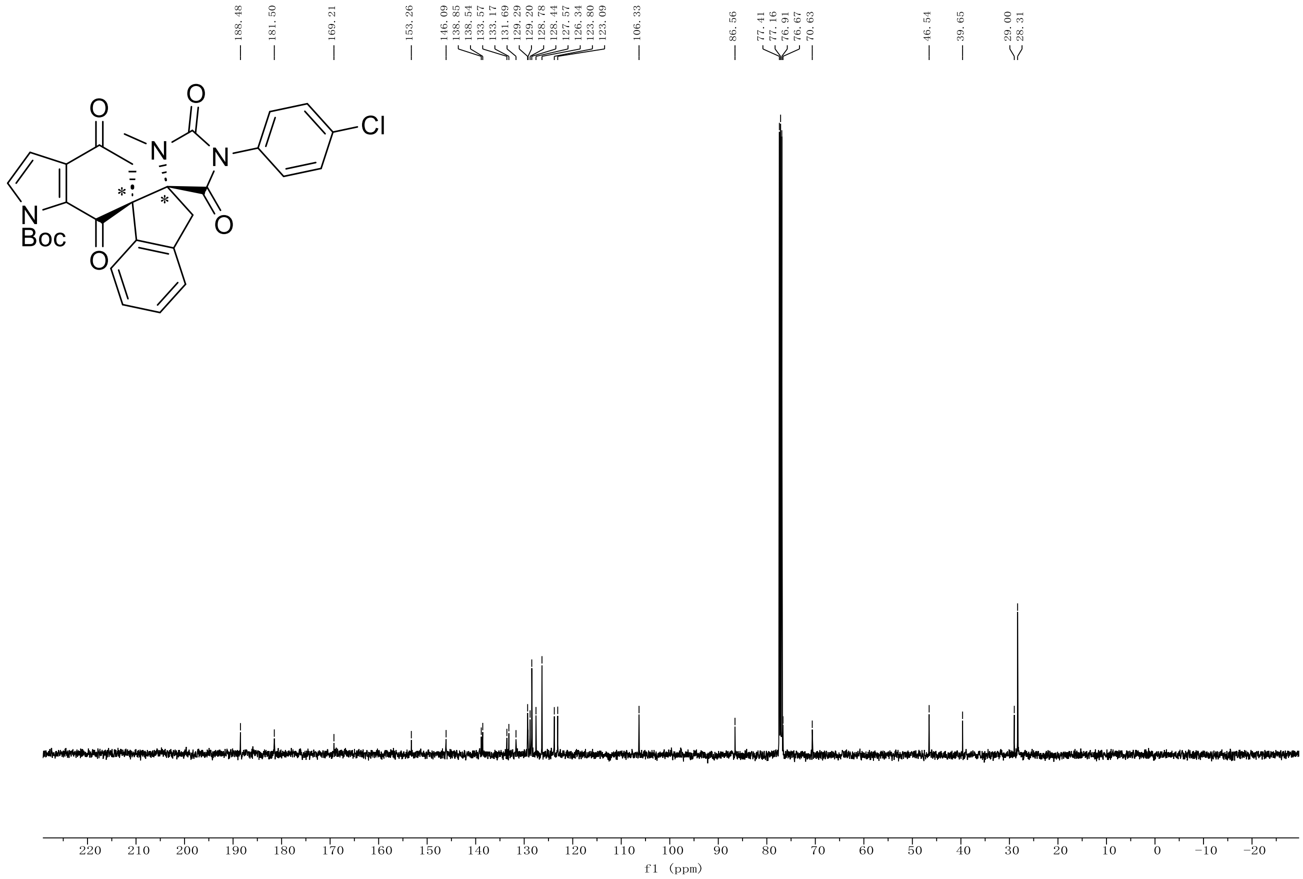

Figure S80: ^{13}C NMR spectra of compound **5b**

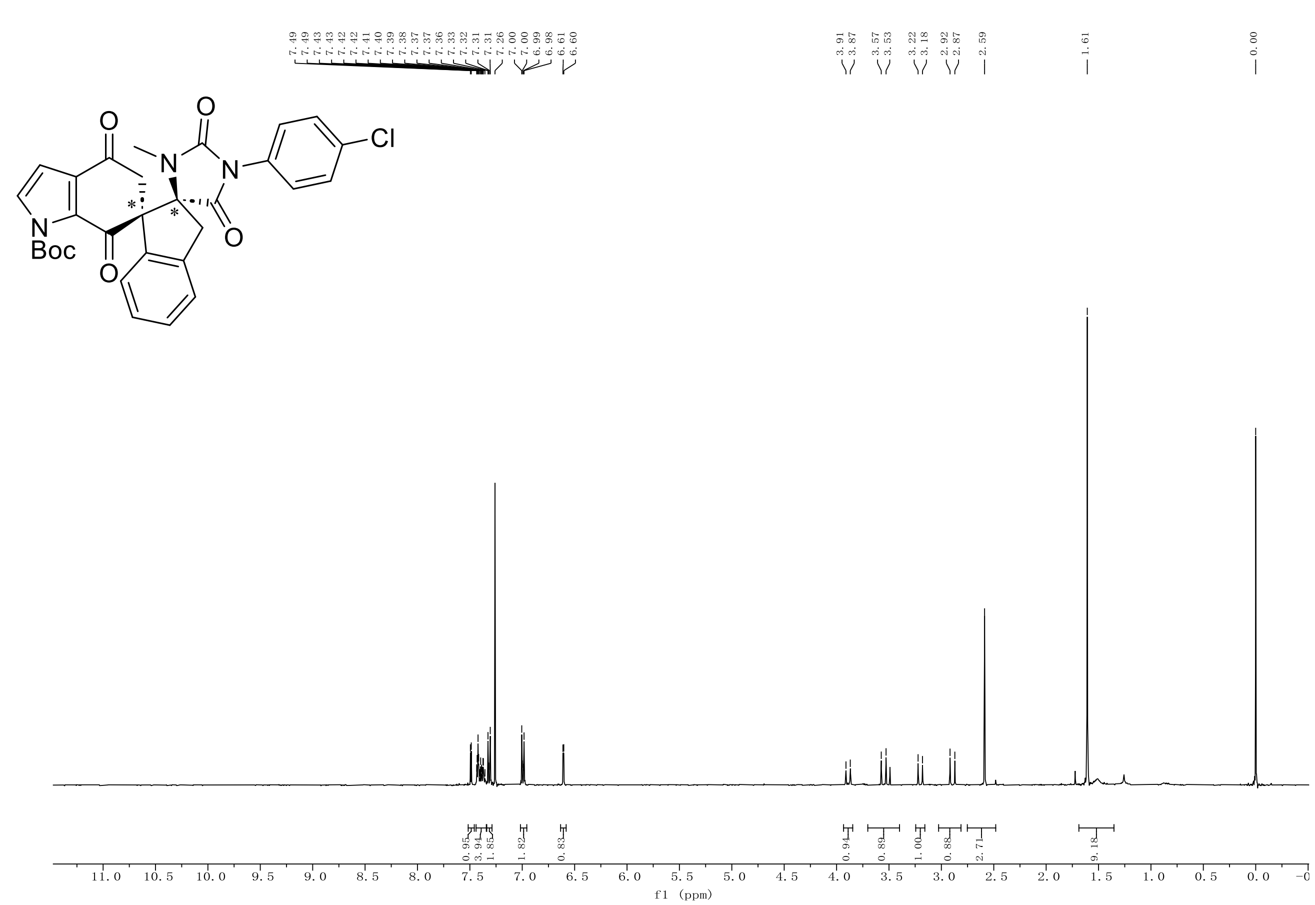

Figure S81: ^{1}H NMR spectra of compound **5'b**

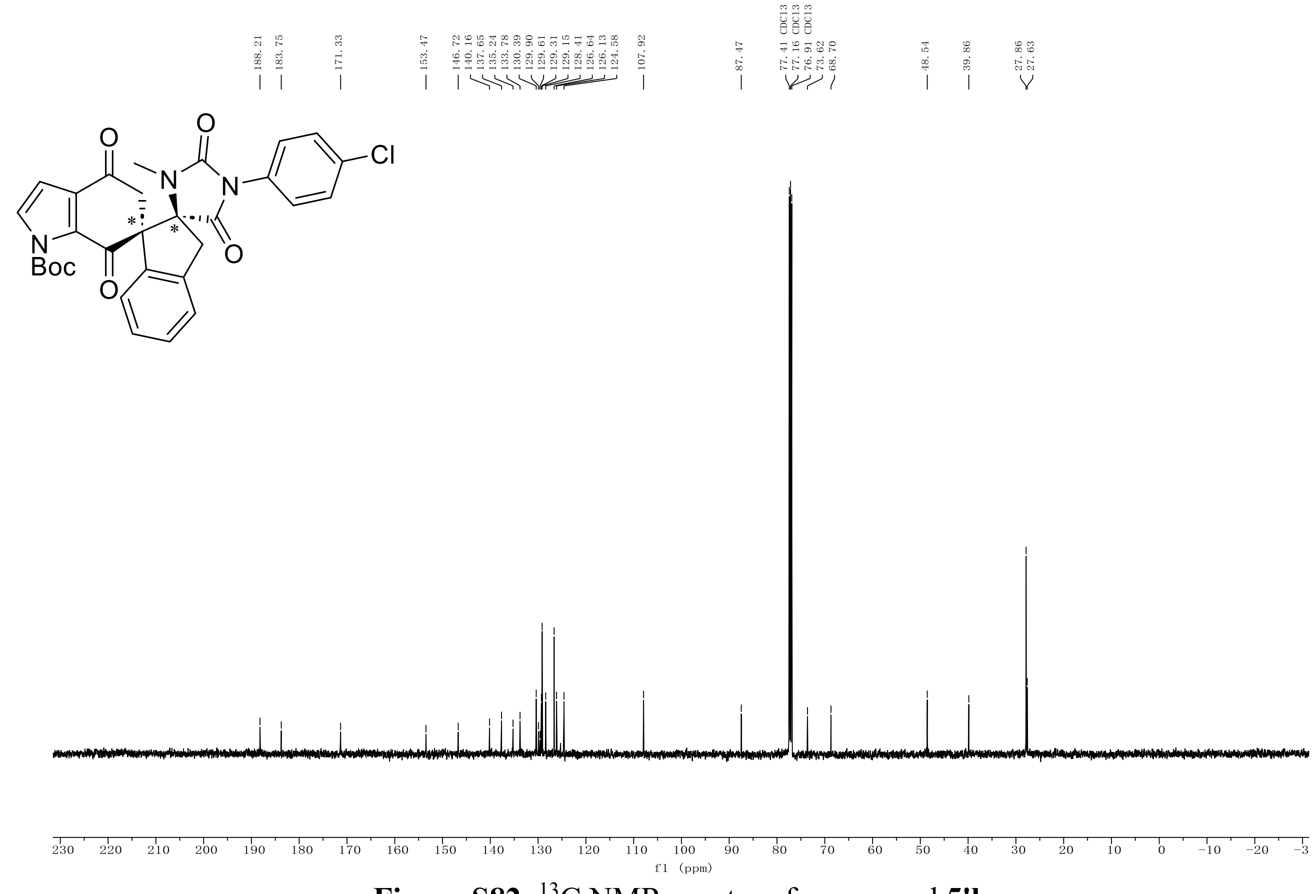

Figure S82: ^{13}C NMR spectra of compound **5'b**

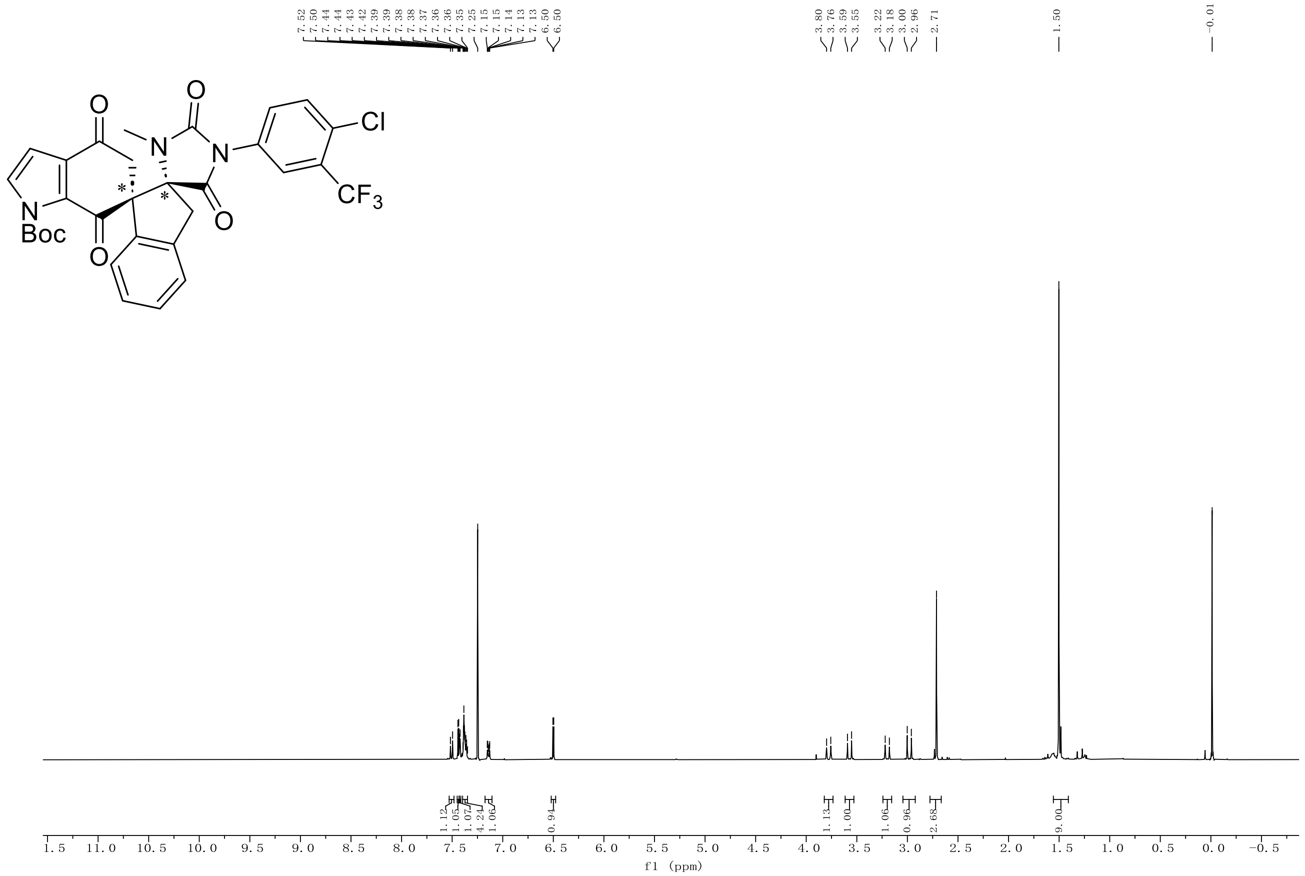

Figure S83: ^{1}H NMR spectra of compound **5c**

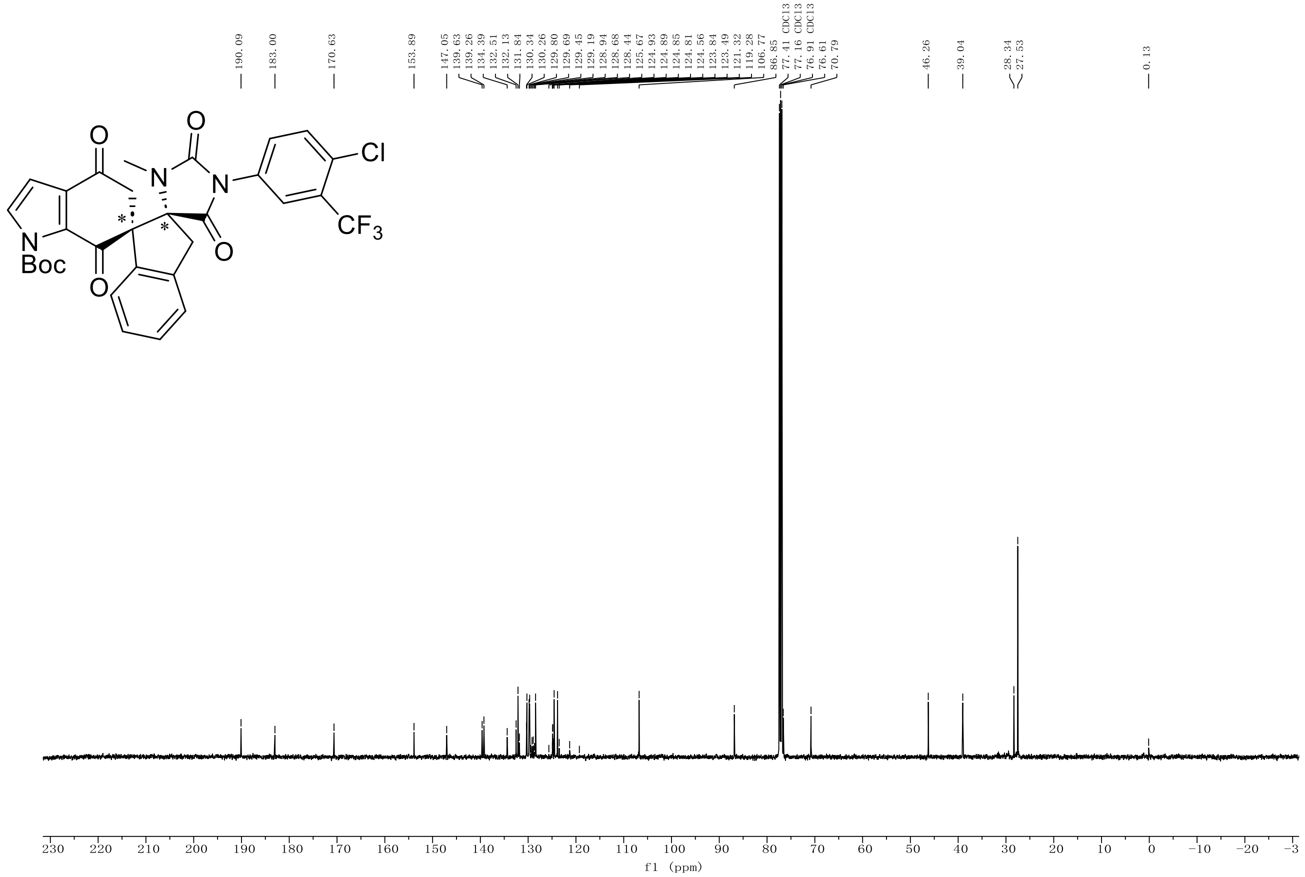

Figure S84: ^{13}C NMR spectra of compound **5c**

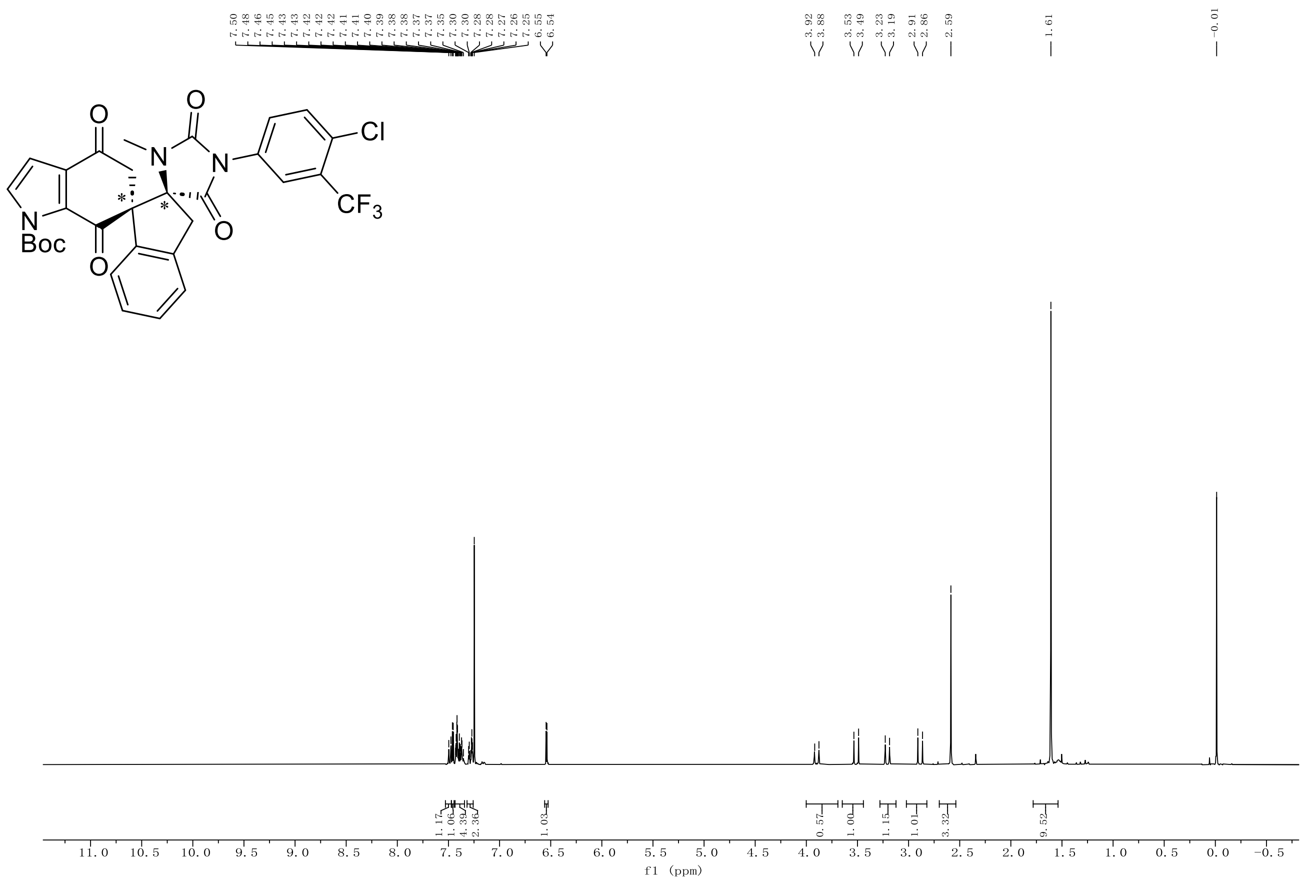

Figure S85: ^{1}H NMR spectra of compound **5'c**

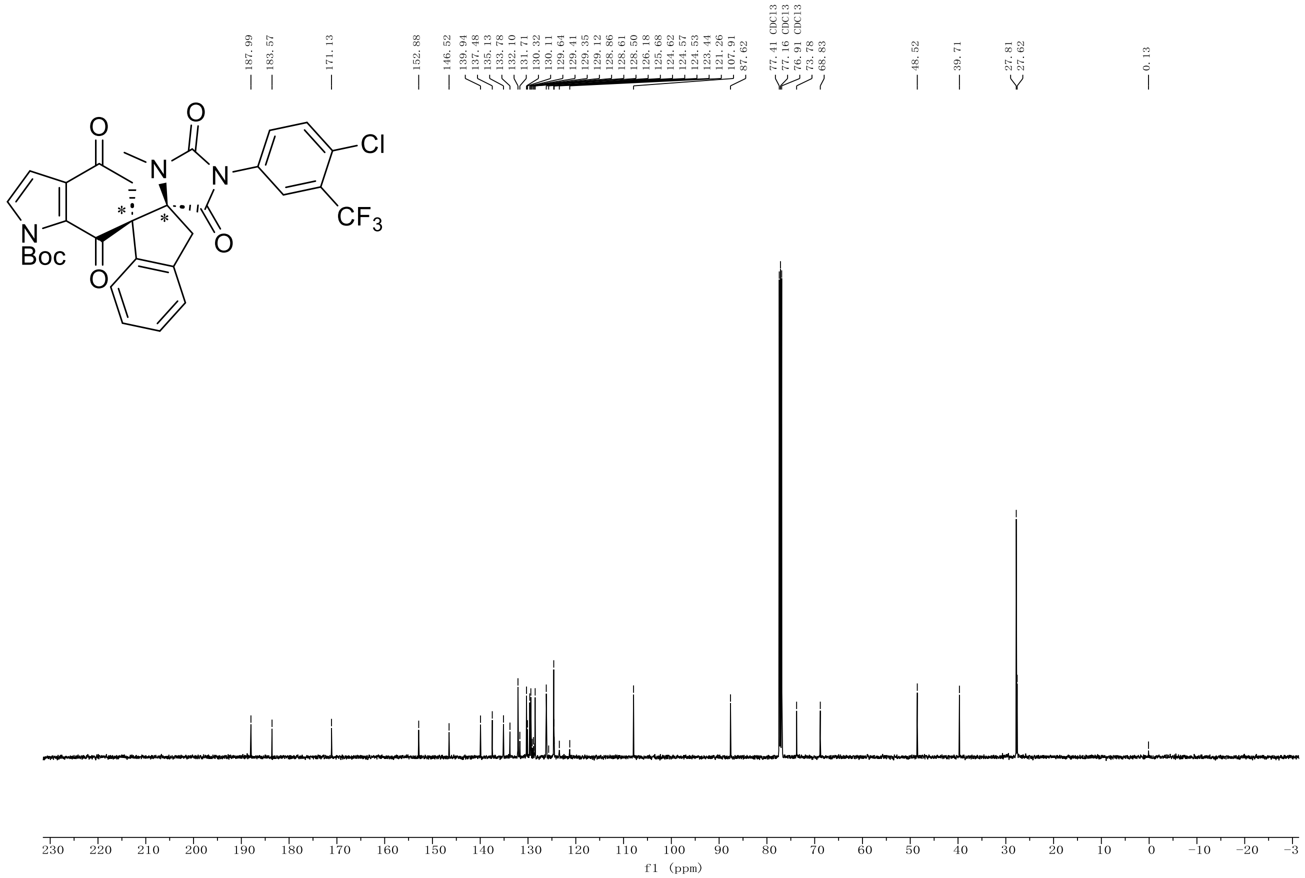

Figure S86: ^{13}C NMR spectra of compound **5'c**

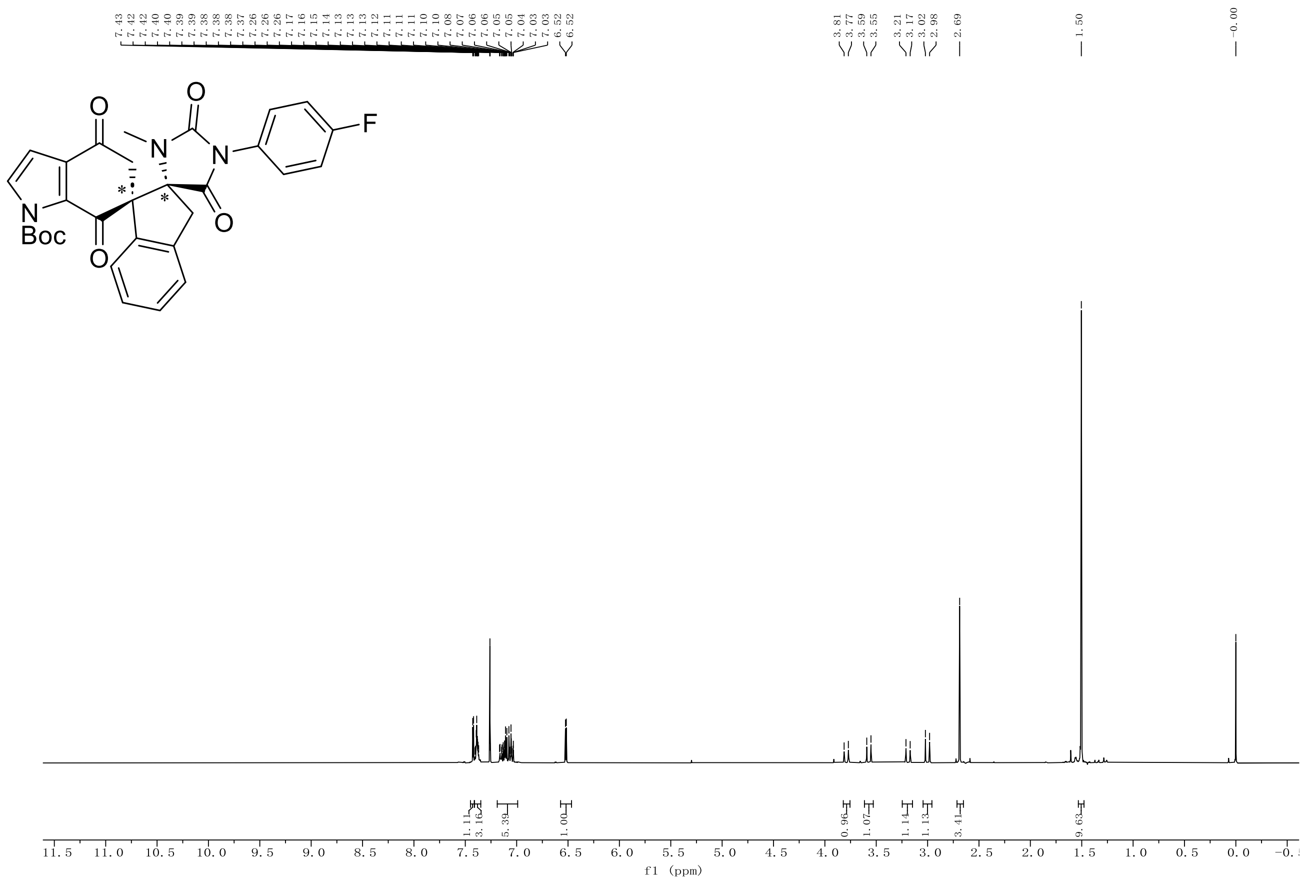

Figure S87: ^{1}H NMR spectra of compound **5d**

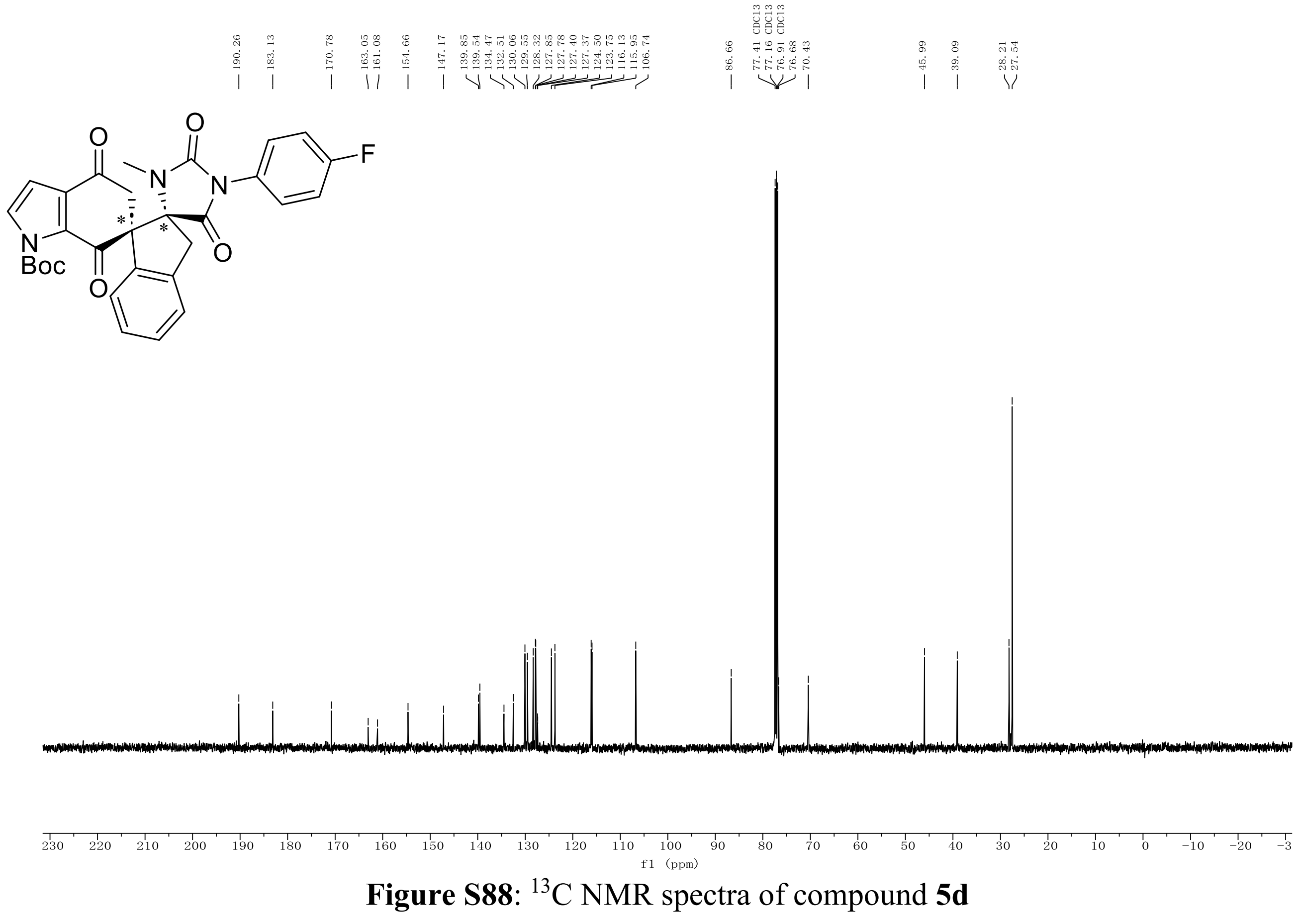

Figure S88: ^{13}C NMR spectra of compound **5d**

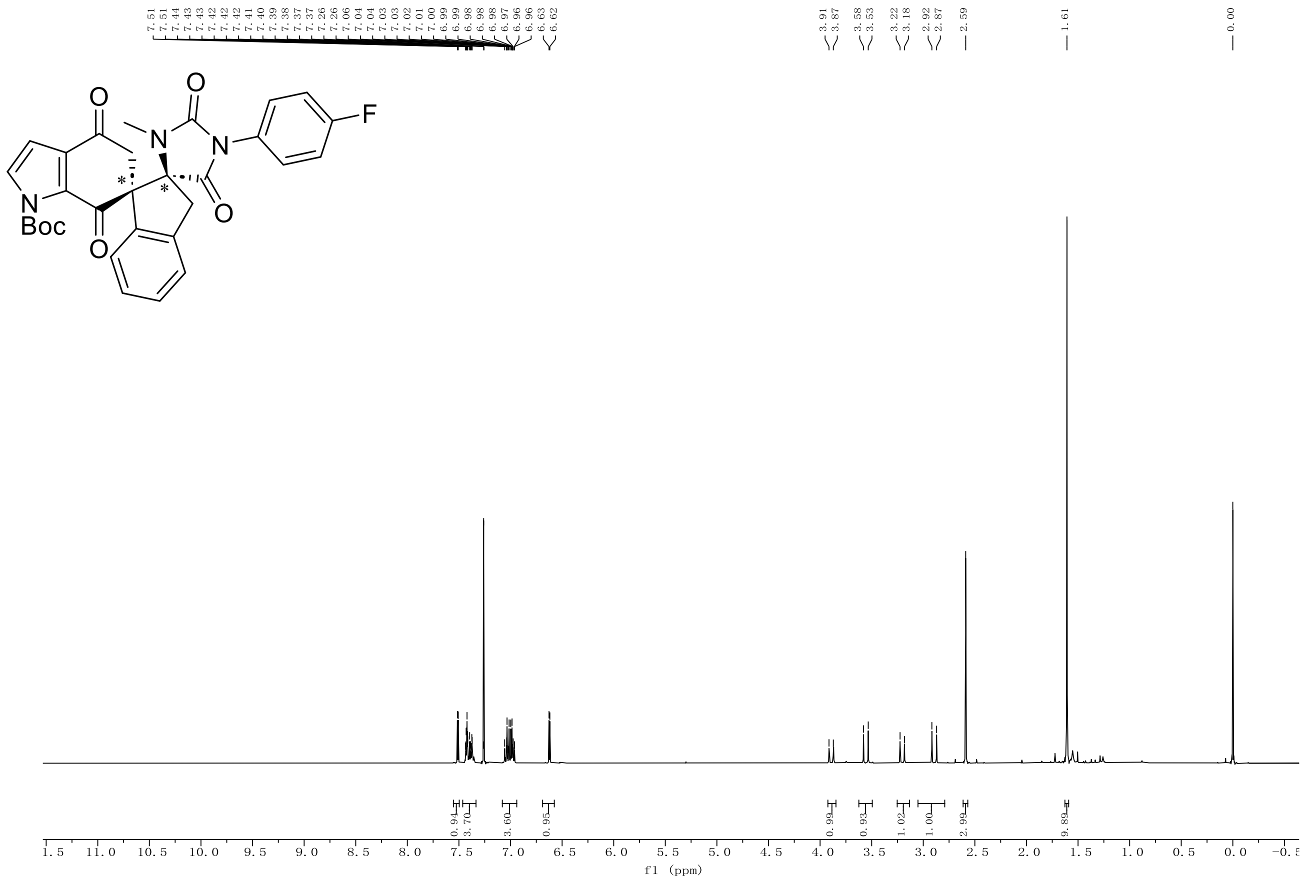

Figure S89: ^{1}H NMR spectra of compound **5'd**

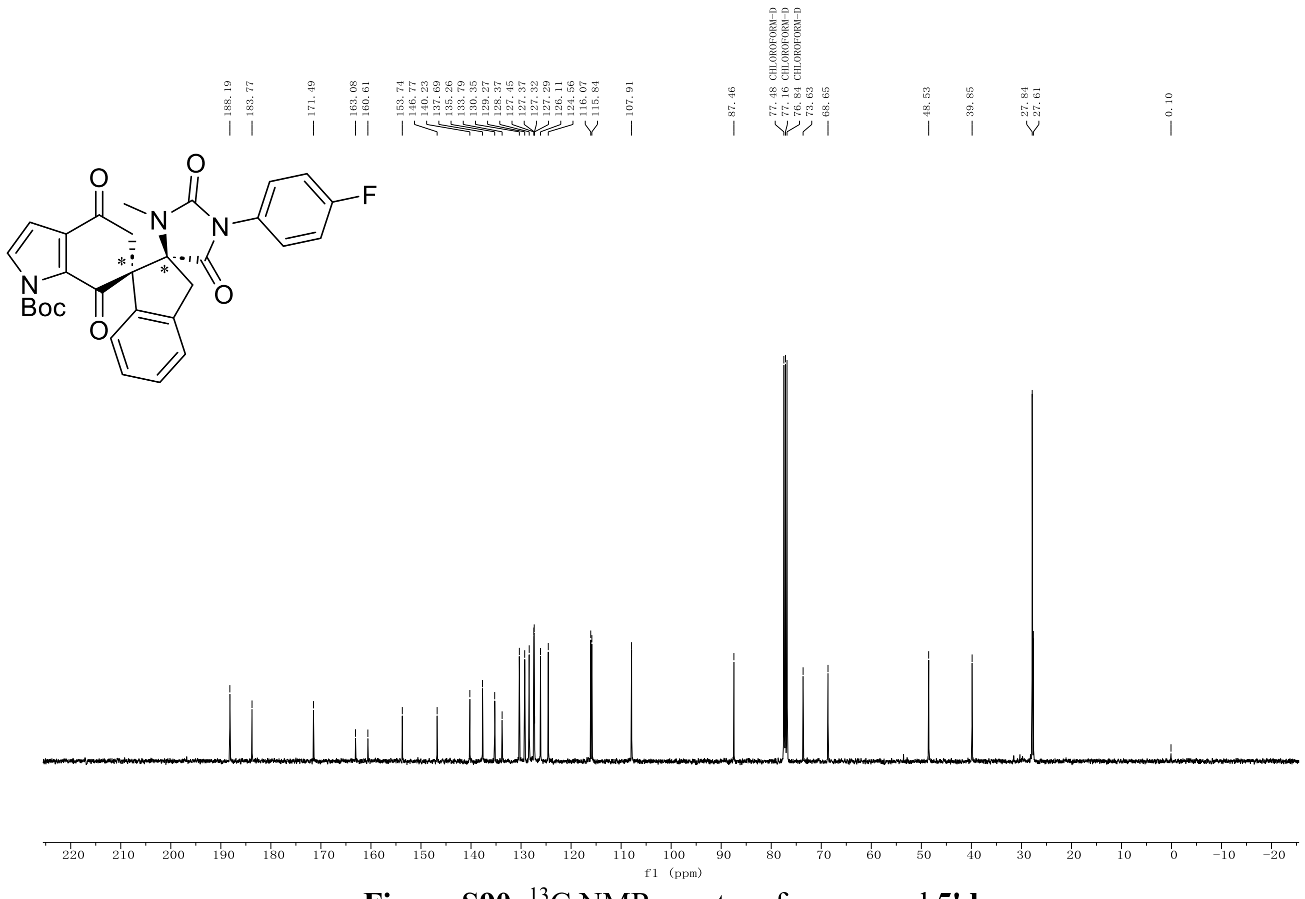

Figure S90: ^{13}C NMR spectra of compound **5'd**

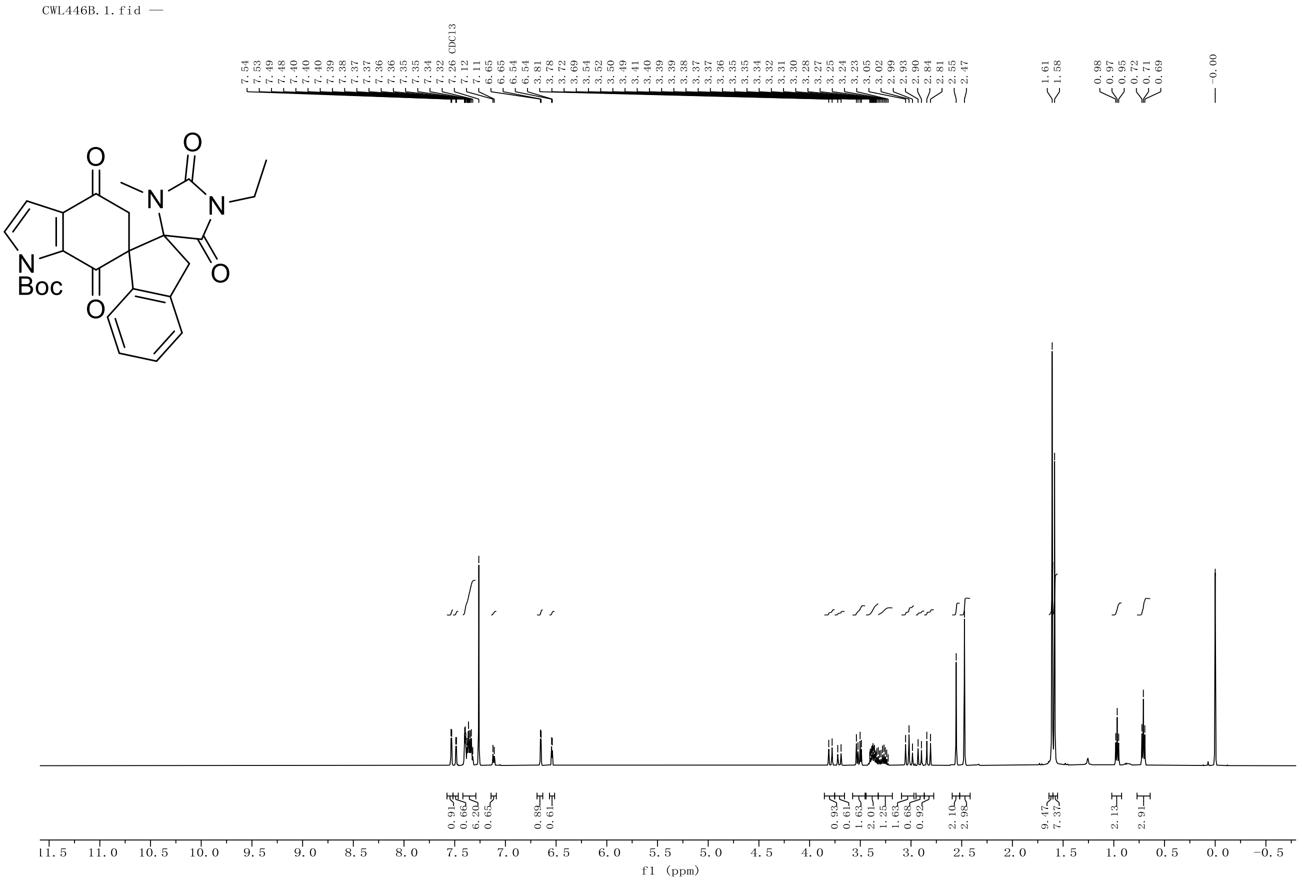

Figure S91: ^{1}H NMR spectra of compound **5e and 5'e**

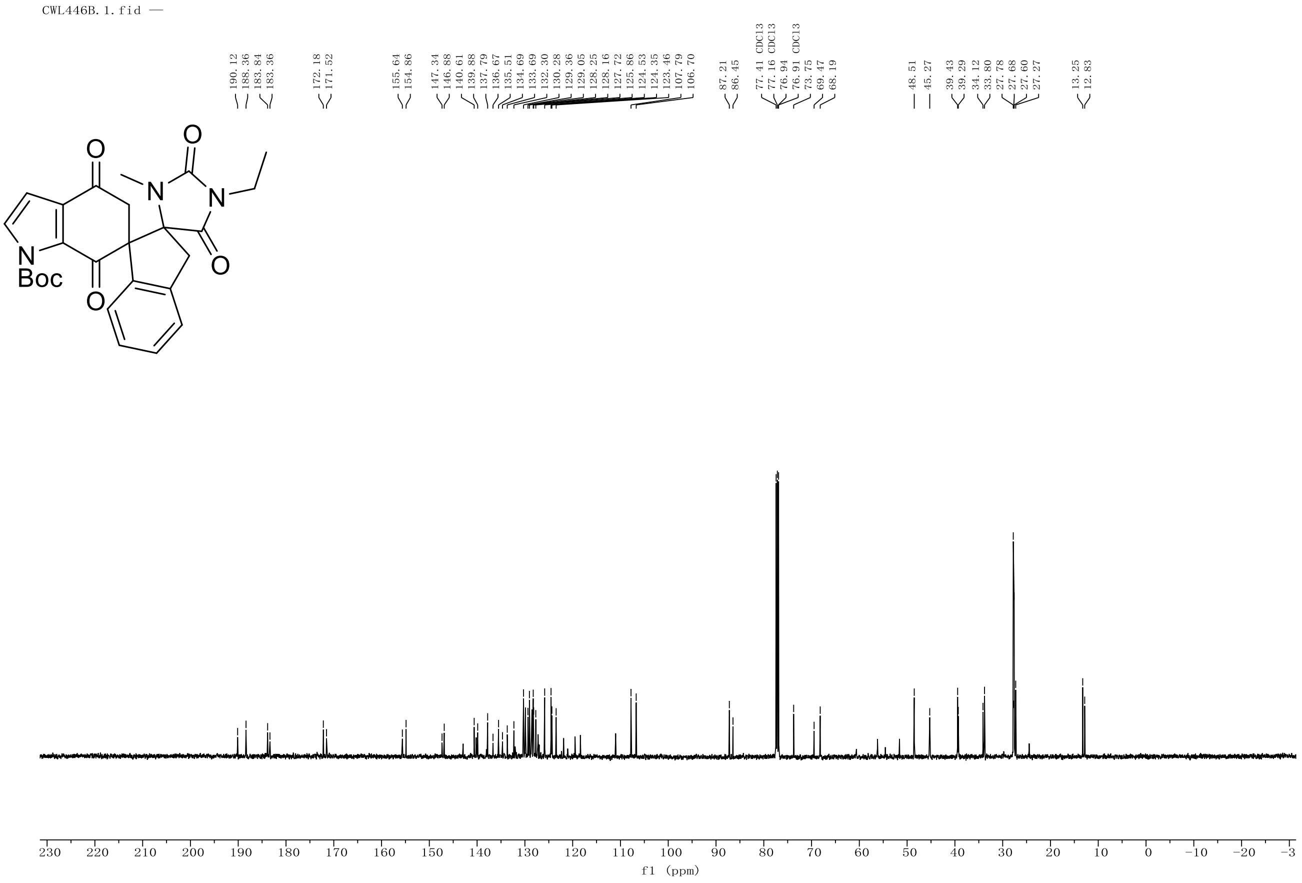

Figure S92: ^{13}C NMR spectra of compound **5e and 5'e**

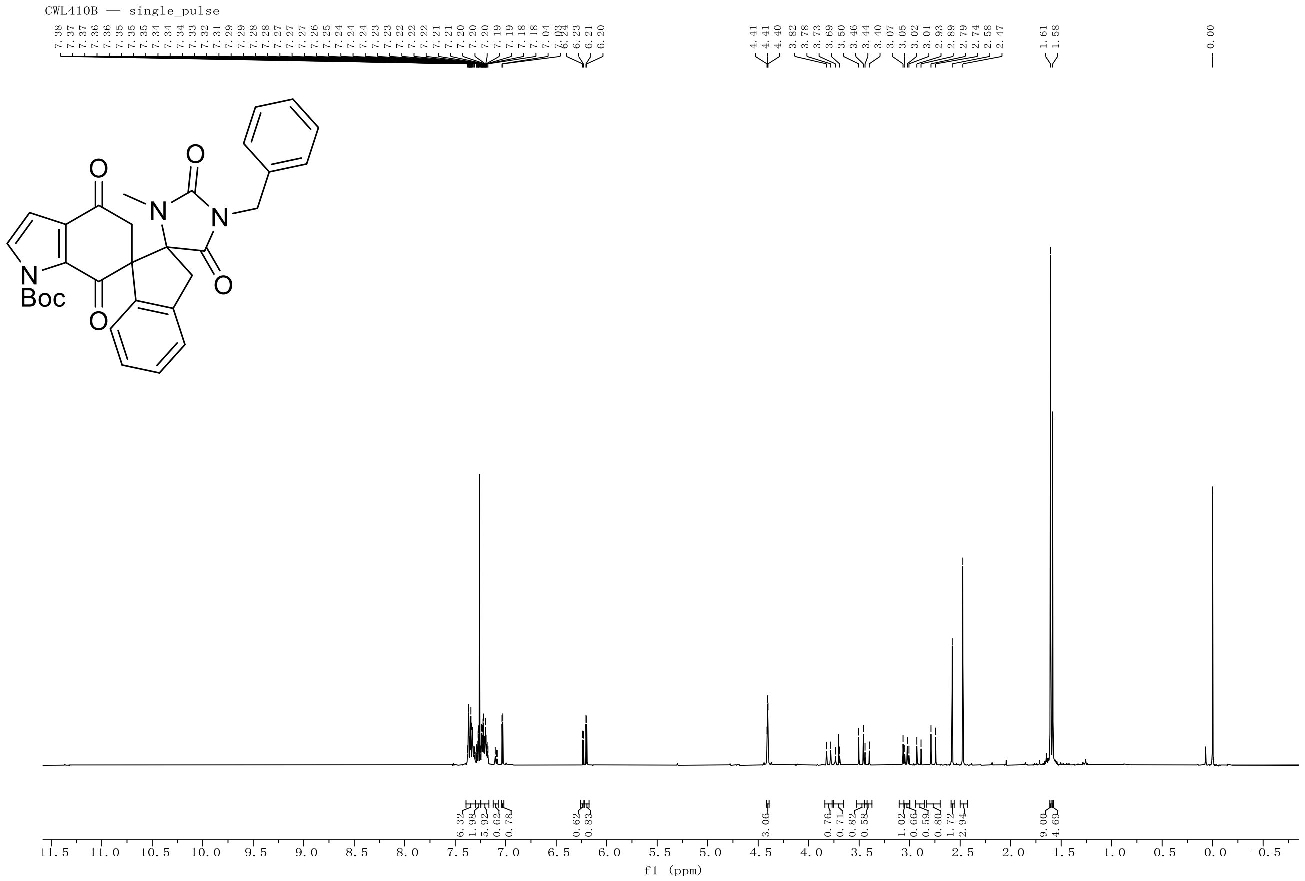

Figure S93: ^{1}H NMR spectra of compound **5f and 5'f**

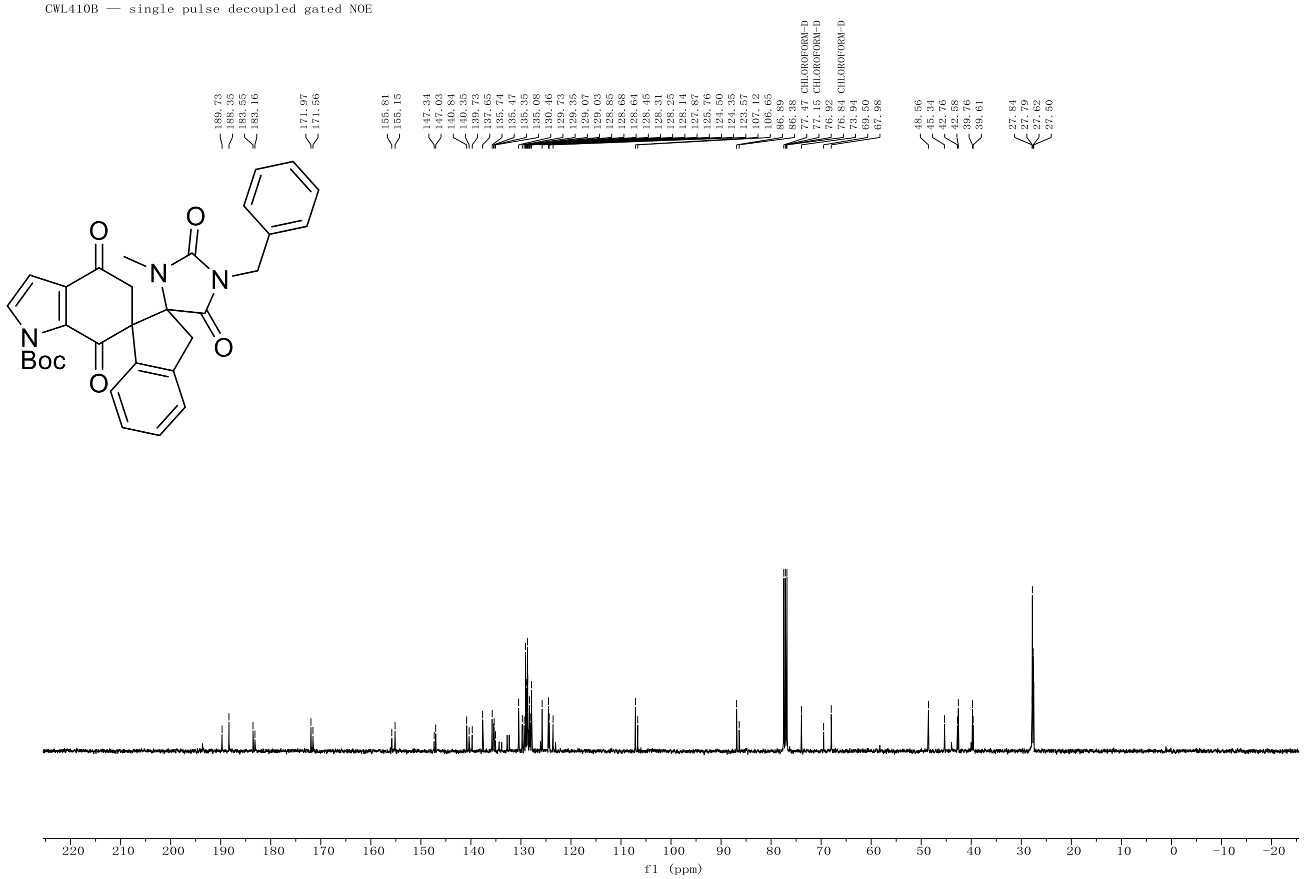

Figure S94: ^{13}C NMR spectra of compound **5f and 5'f**

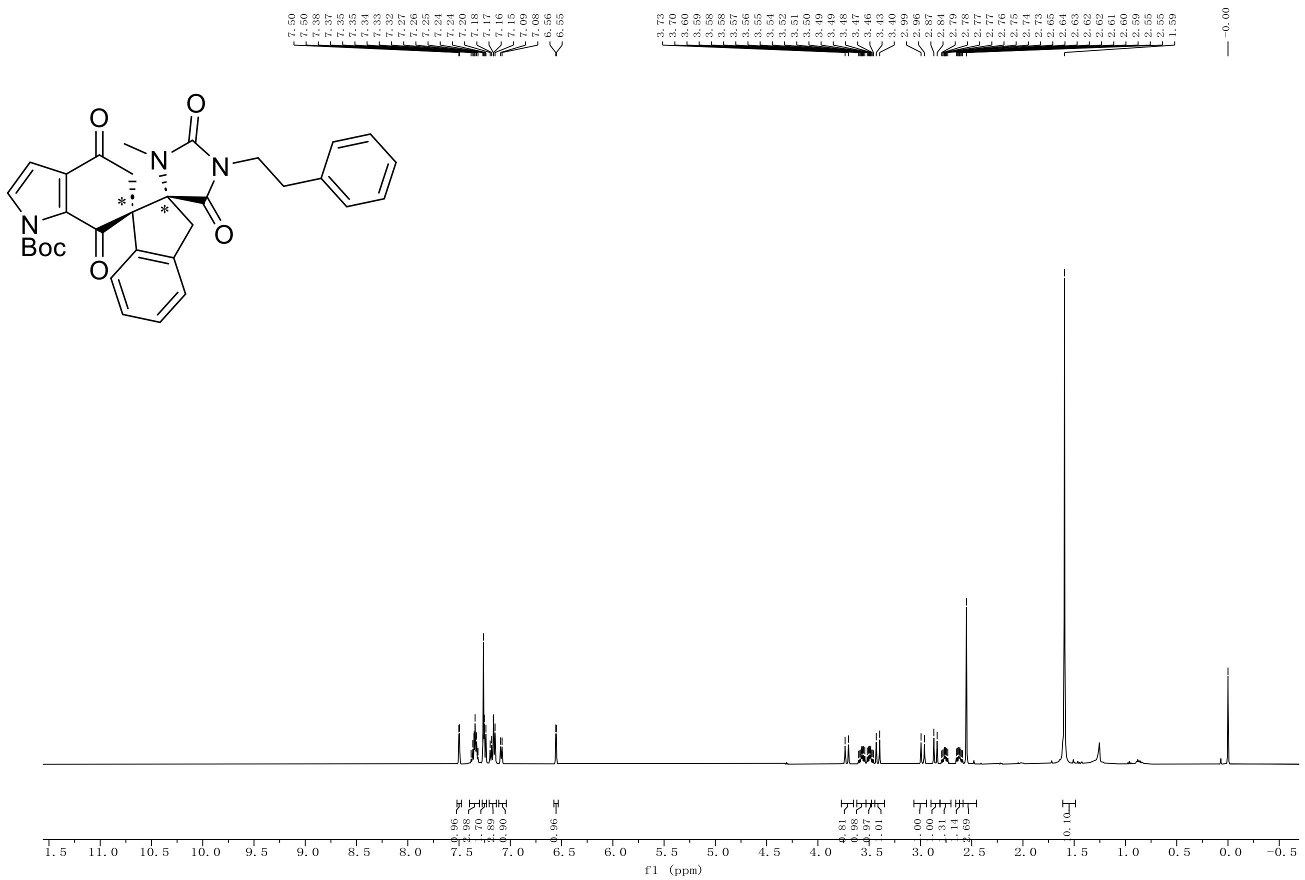

Figure S95: ^{1}H NMR spectra of compound **5g**

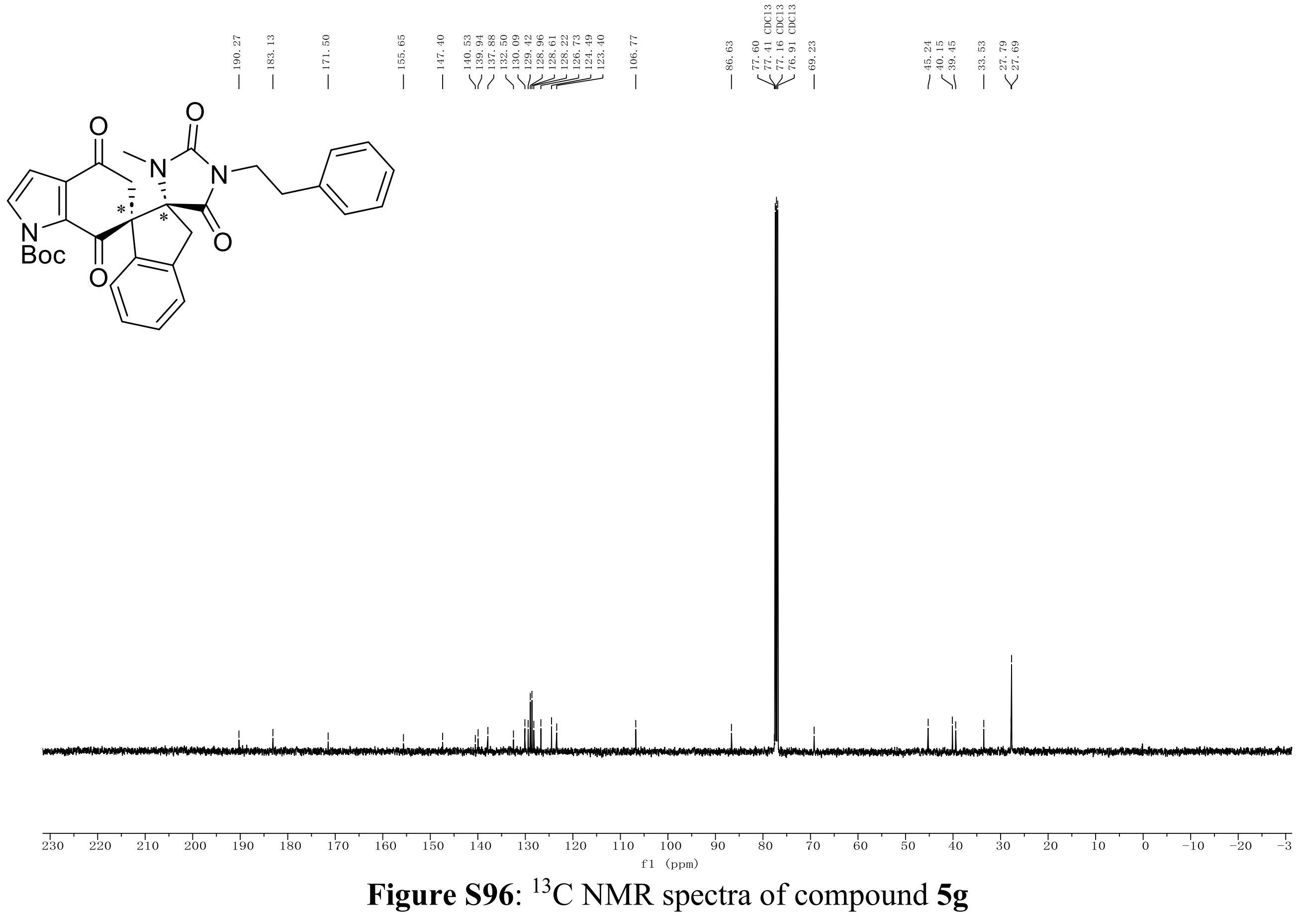

Figure S96: ^{13}C NMR spectra of compound **5g**

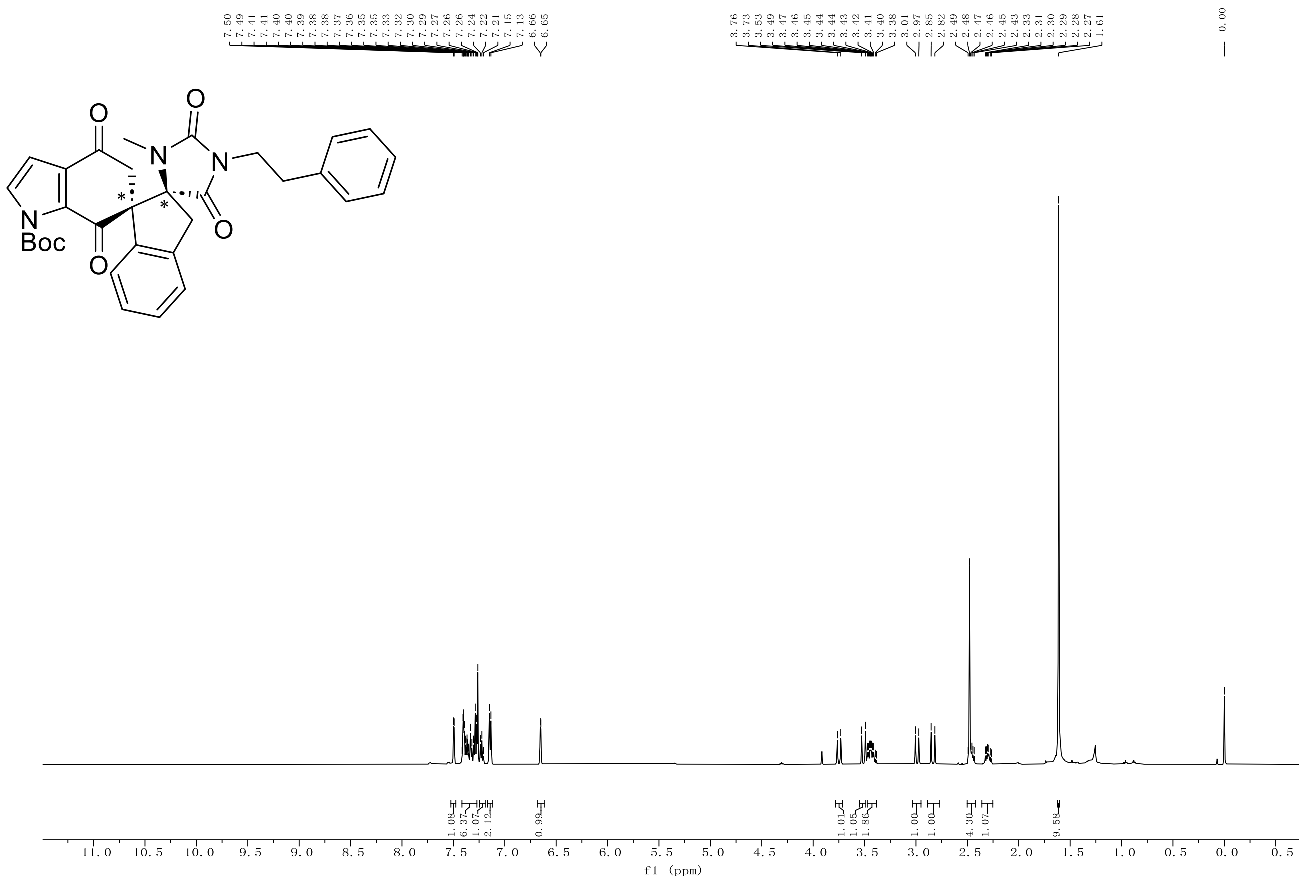

Figure S97: ^{1}H NMR spectra of compound **5'g**

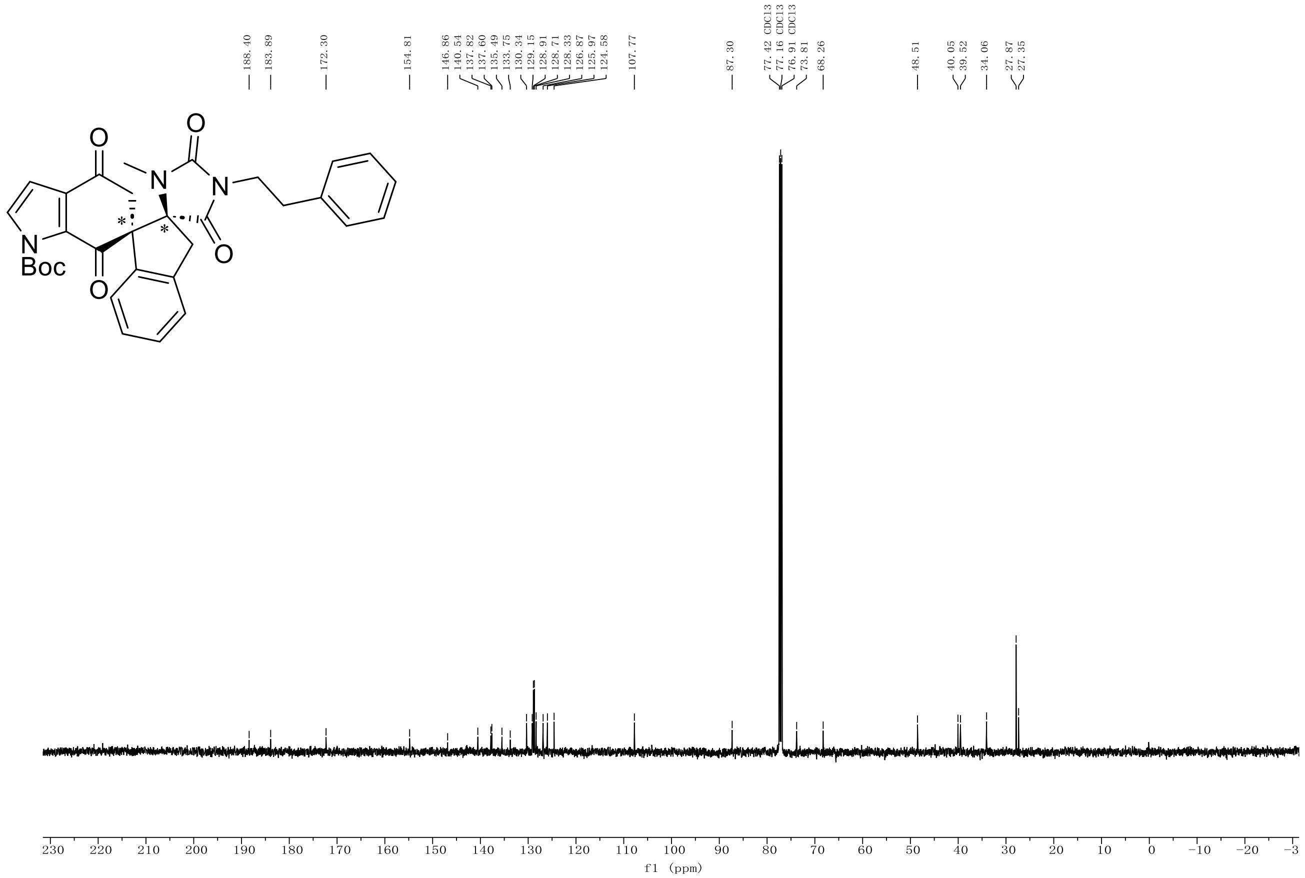

Figure S98: ^{13}C NMR spectra of compound **5'g**

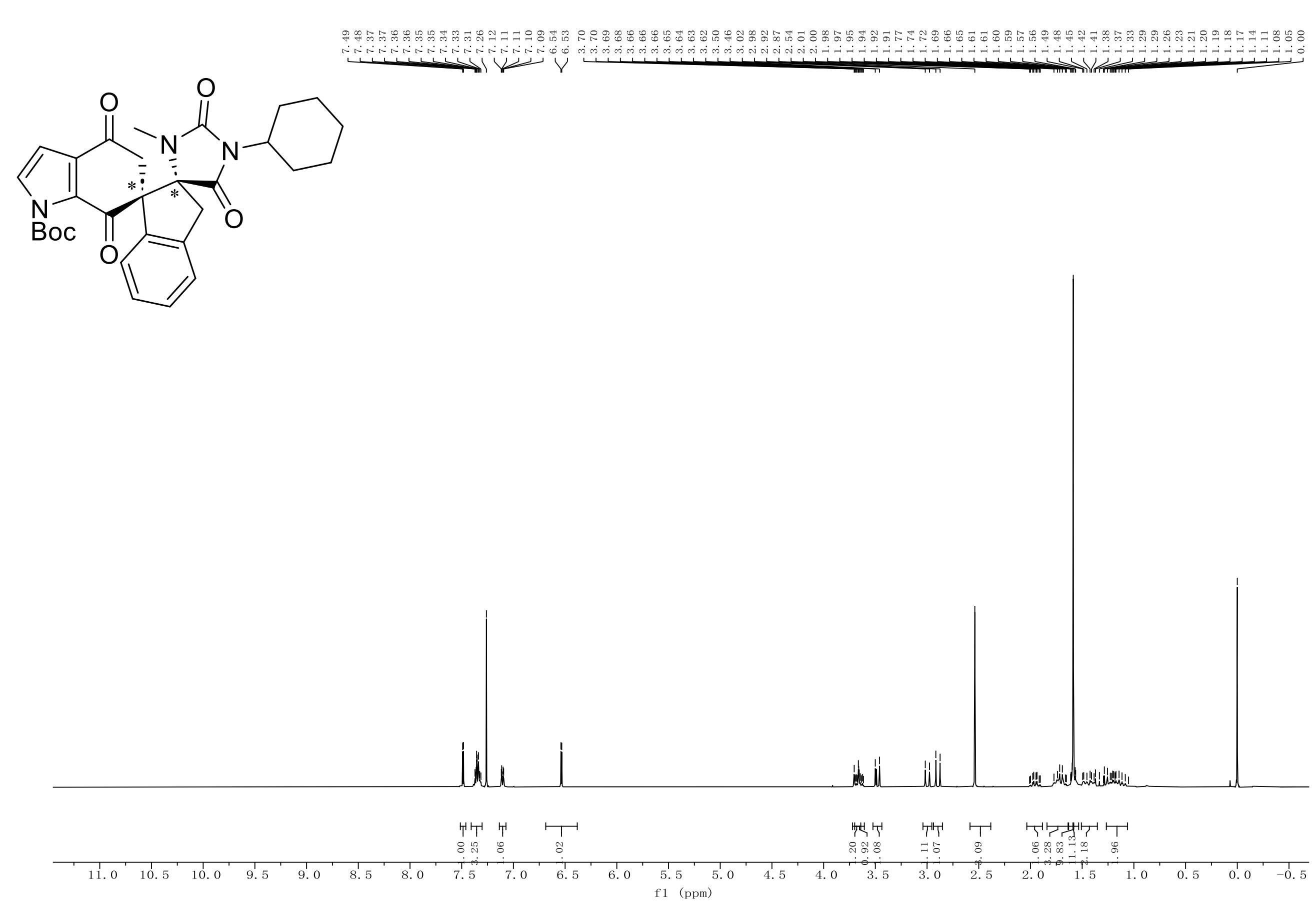

Figure S99: ^{1}H NMR spectra of compound **5h**

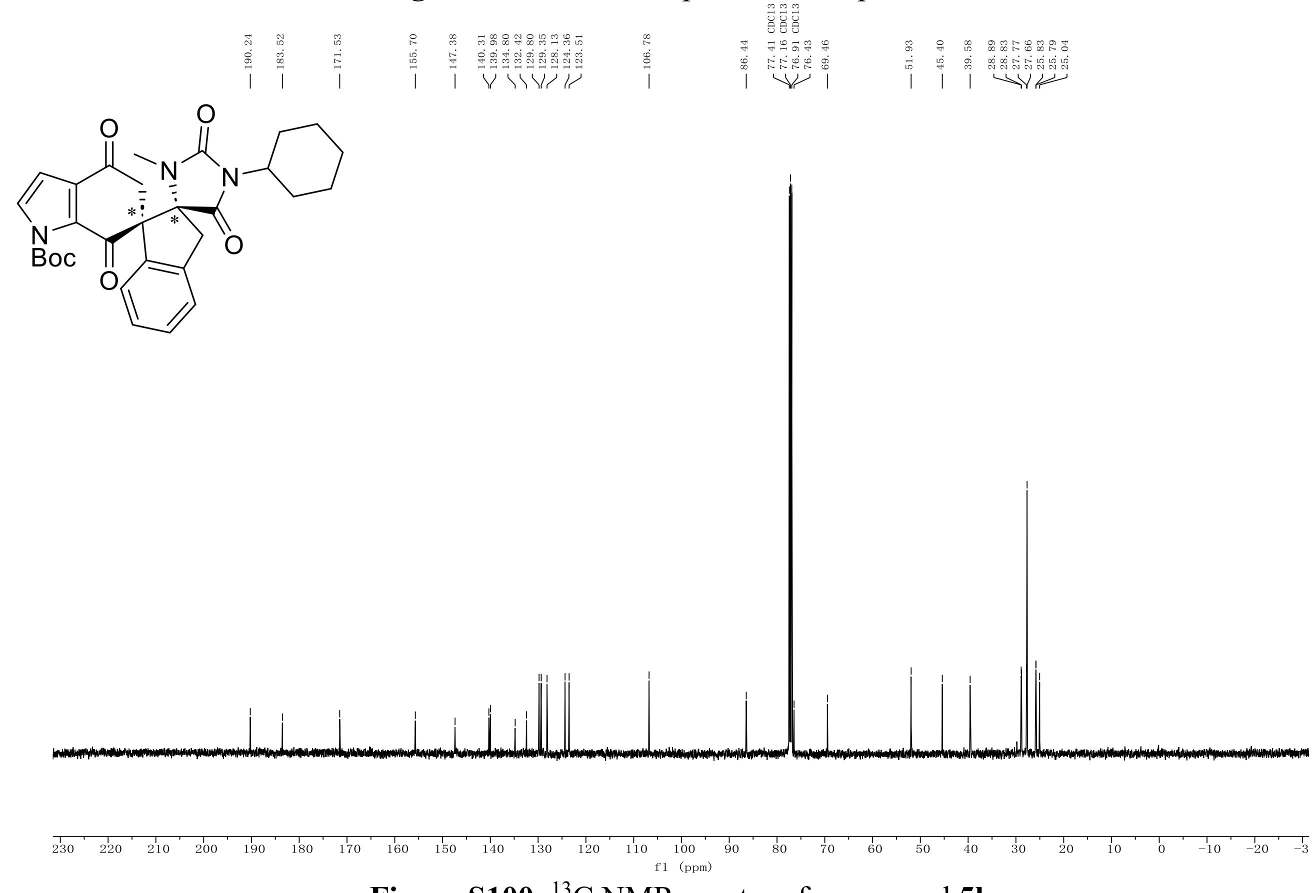

Figure S100: ^{13}C NMR spectra of compound **5h**

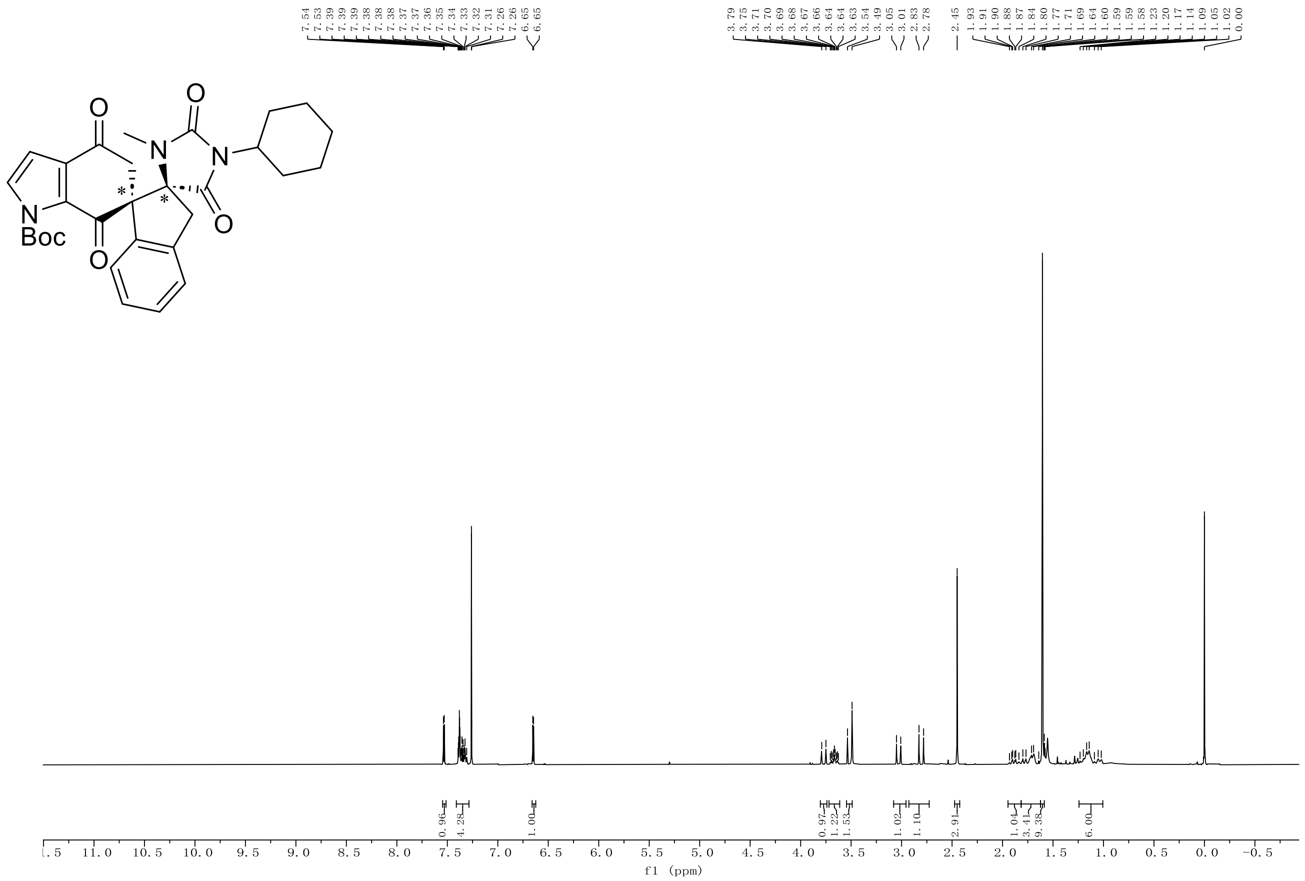

Figure S101: ^{1}H NMR spectra of compound **5'h**

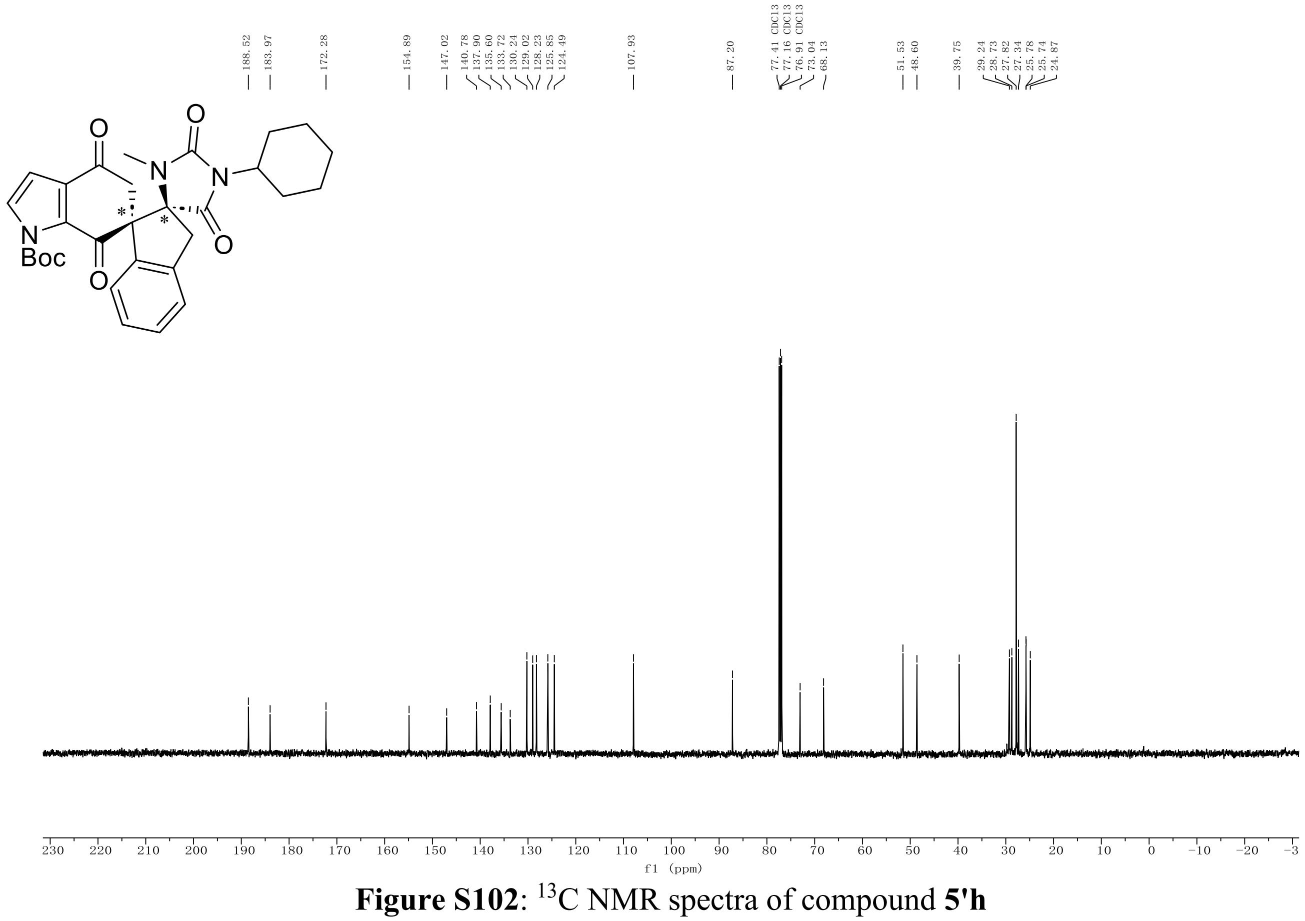

Figure S102: ^{13}C NMR spectra of compound **5'h**

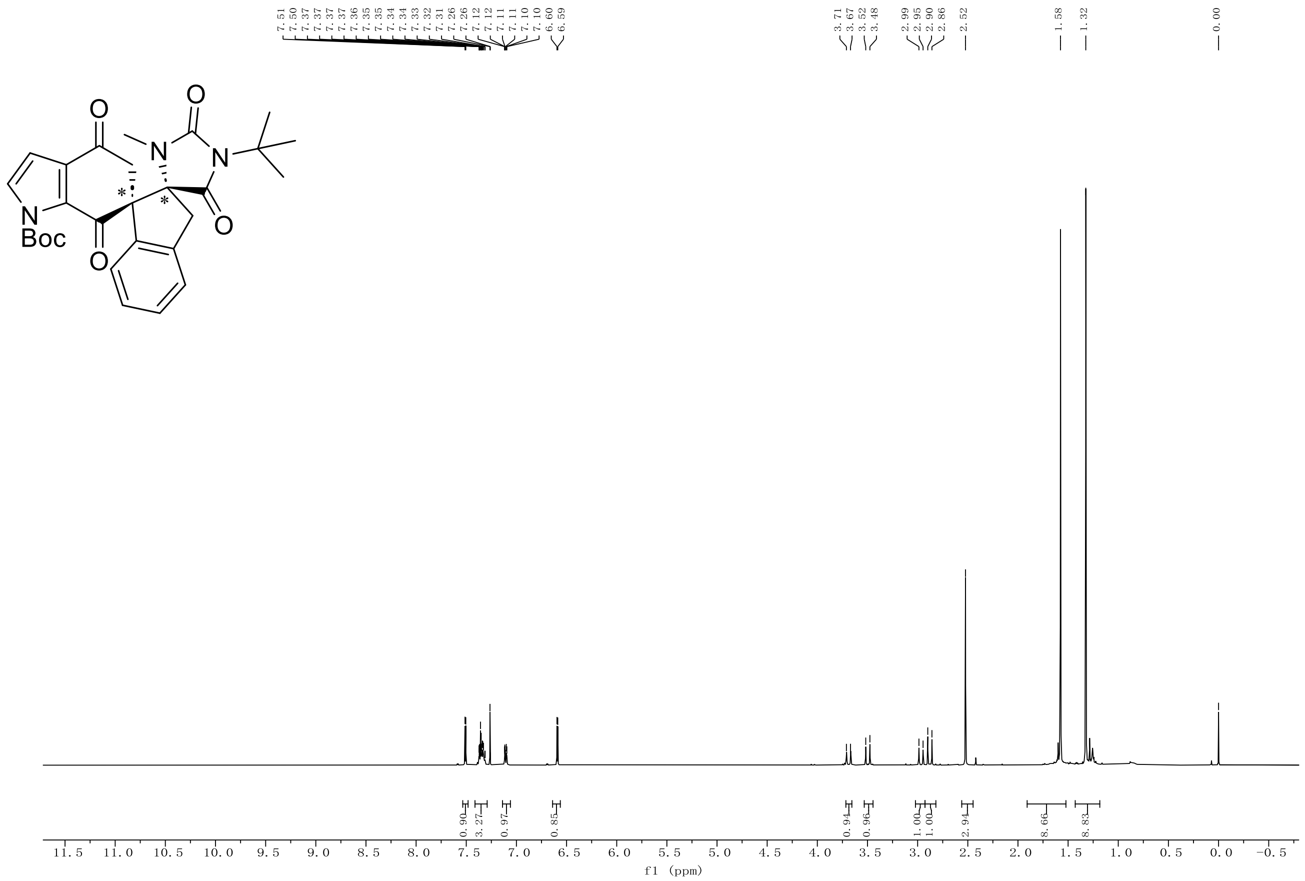

Figure S103: ^{1}H NMR spectra of compound **5i**

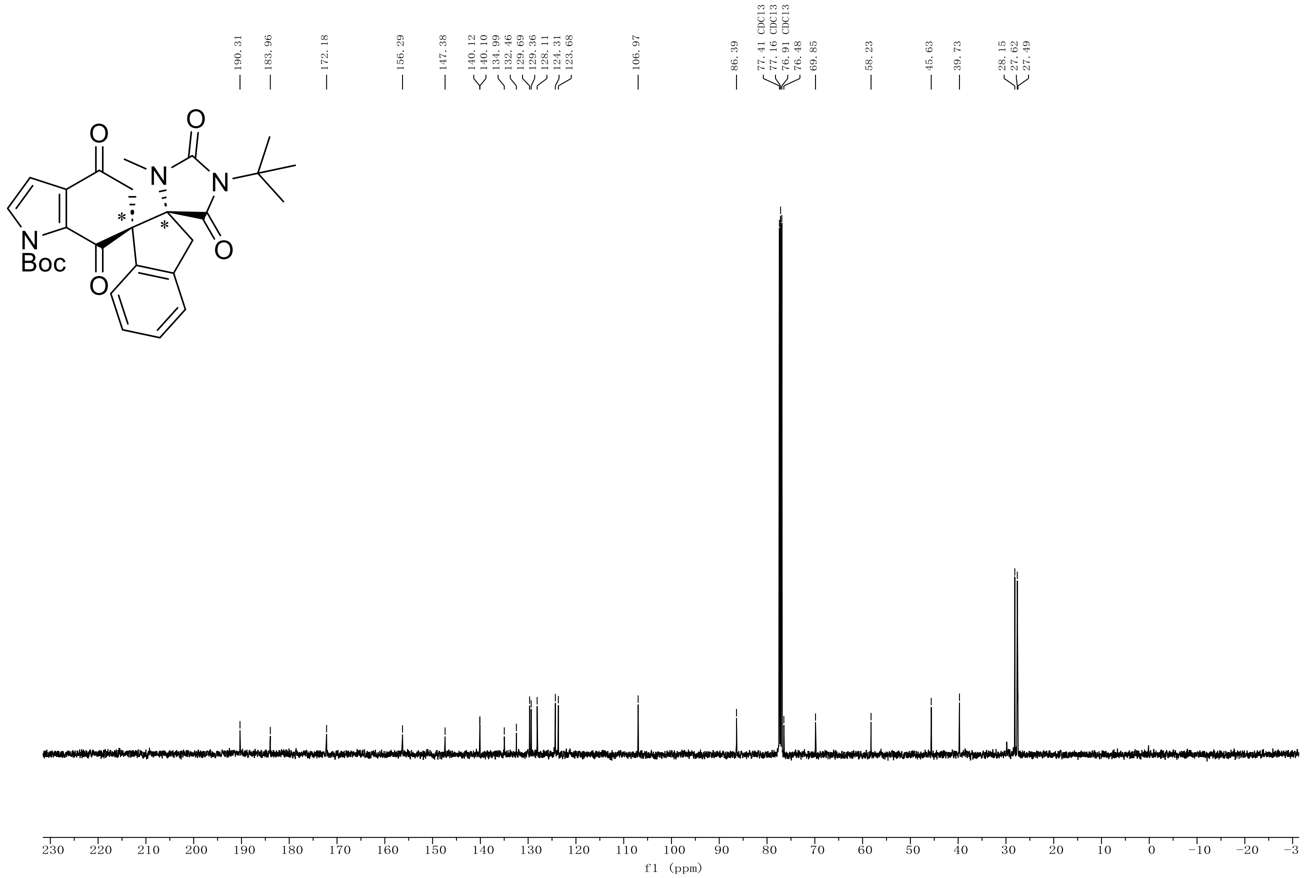

Figure S104: ^{13}C NMR spectra of compound **5i**

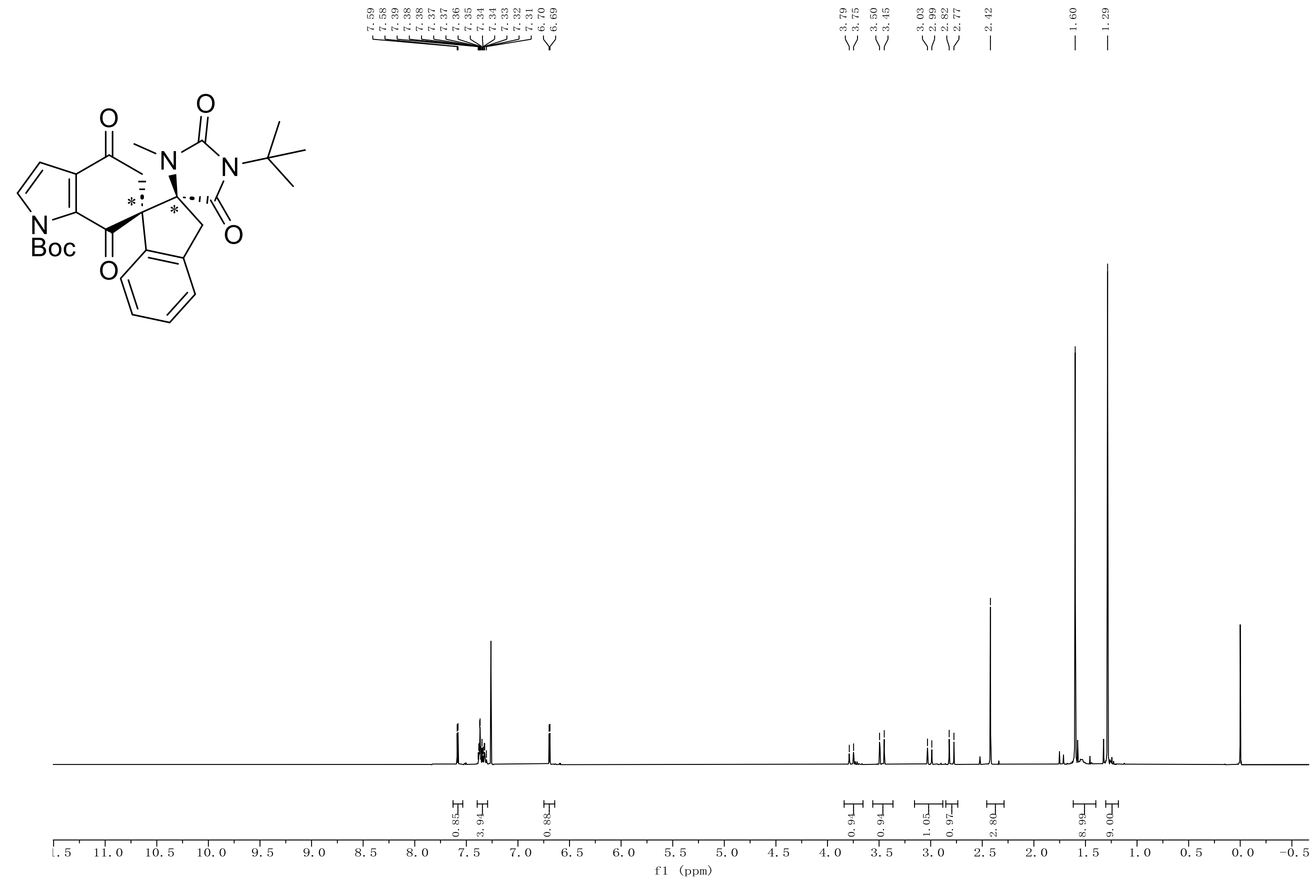

Figure S105: ^{1}H NMR spectra of compound **5'i**

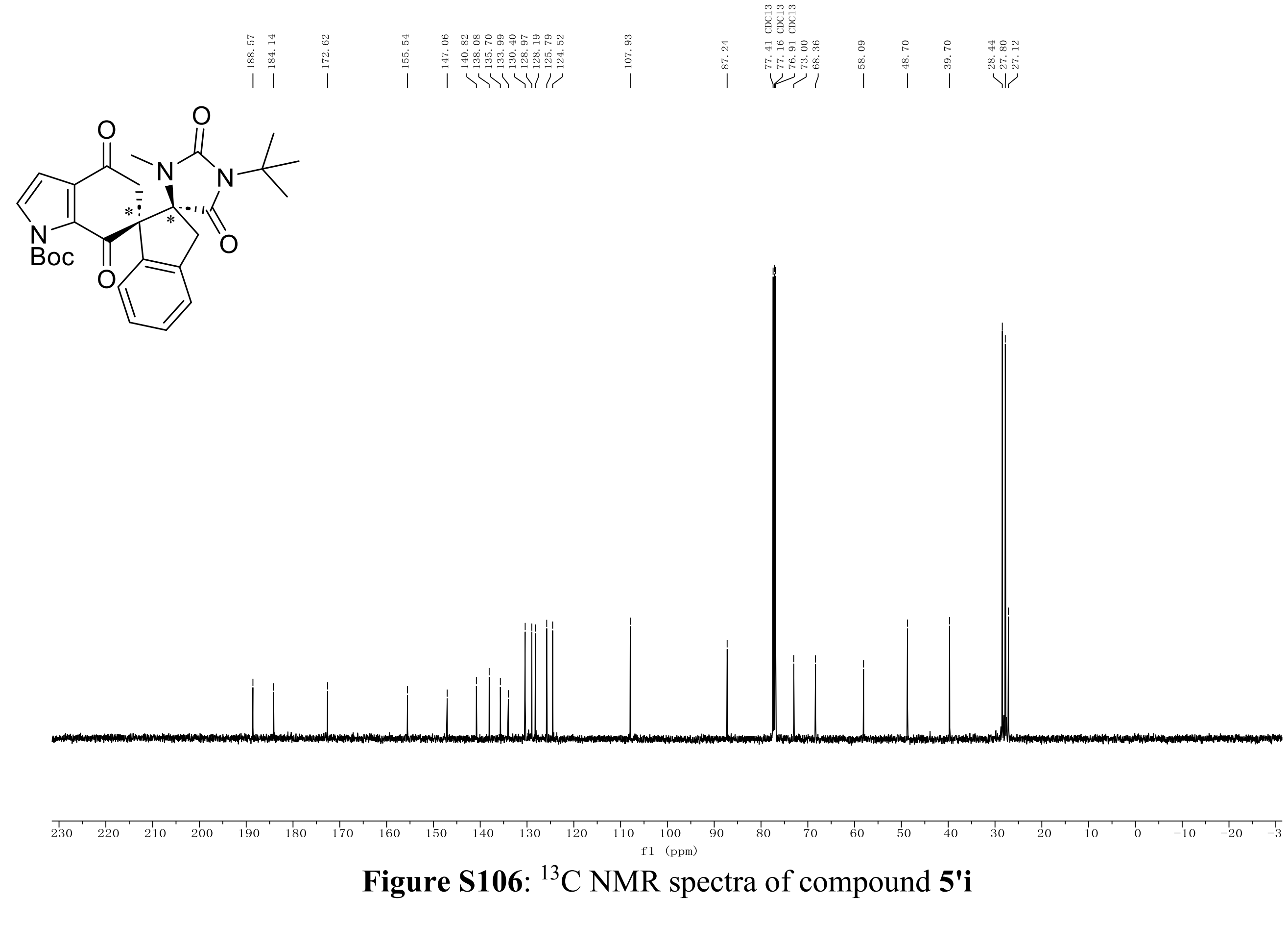

Figure S106: ^{13}C NMR spectra of compound **5'i**

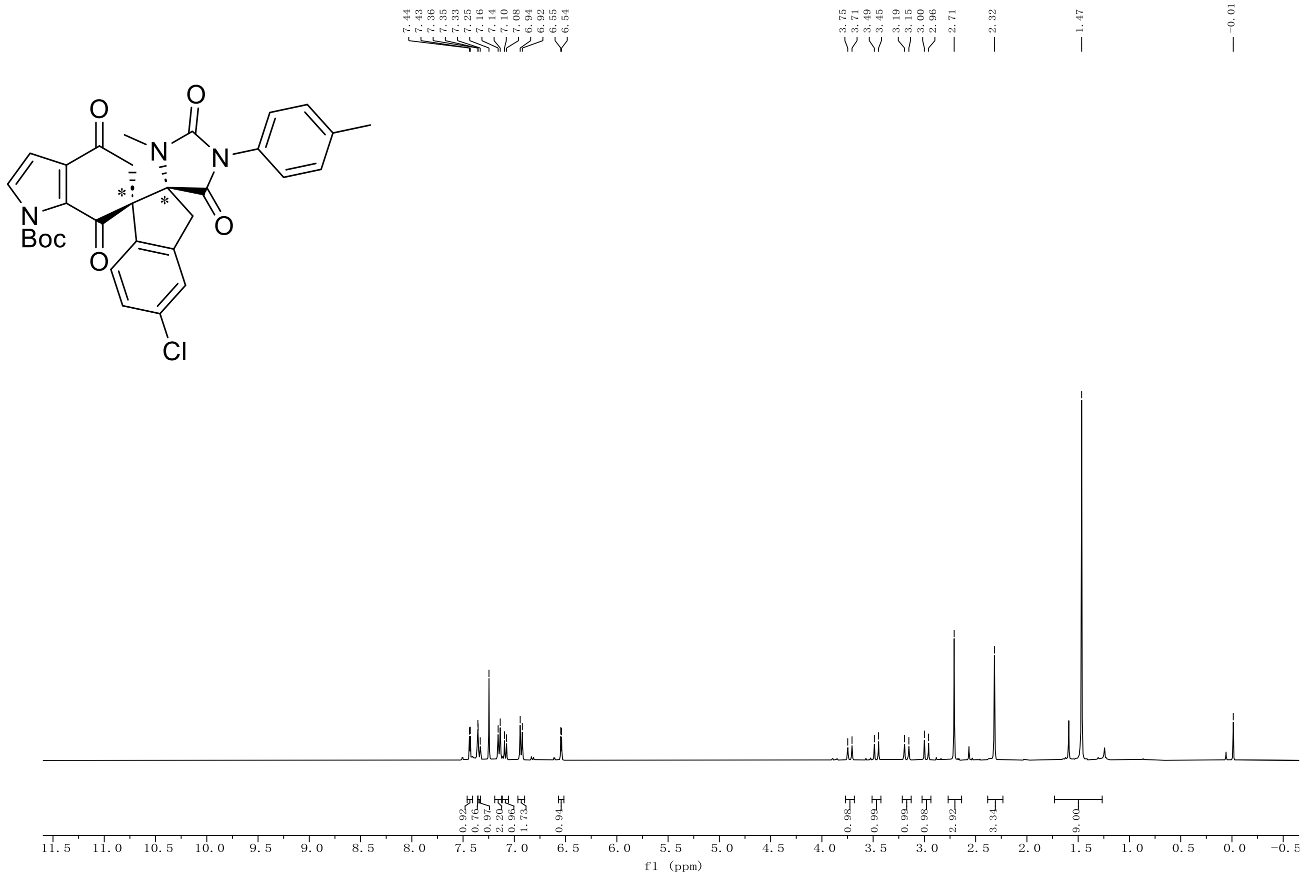

Figure S107: ^{1}H NMR spectra of compound **5j**

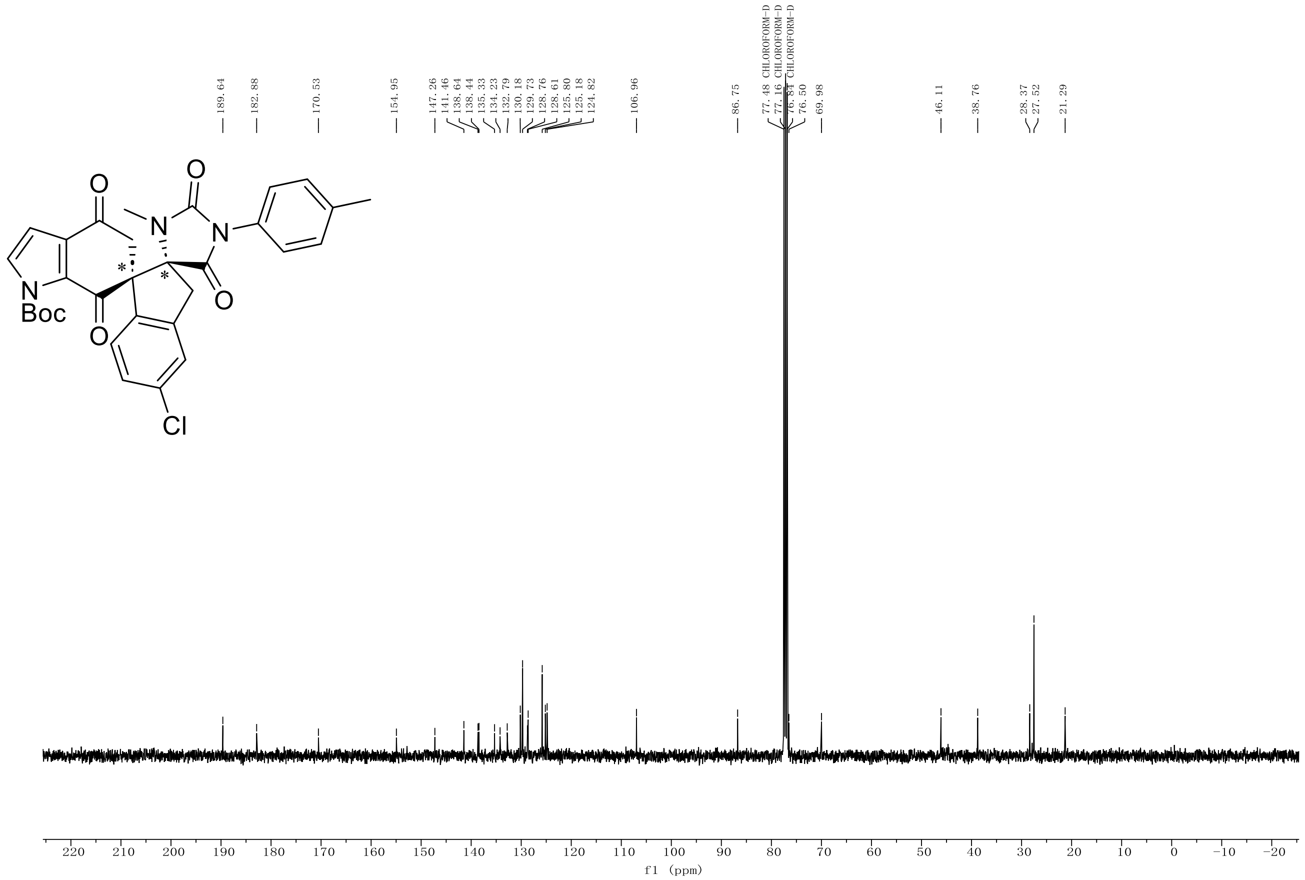

Figure S108: ^{13}C NMR spectra of compound **5j**

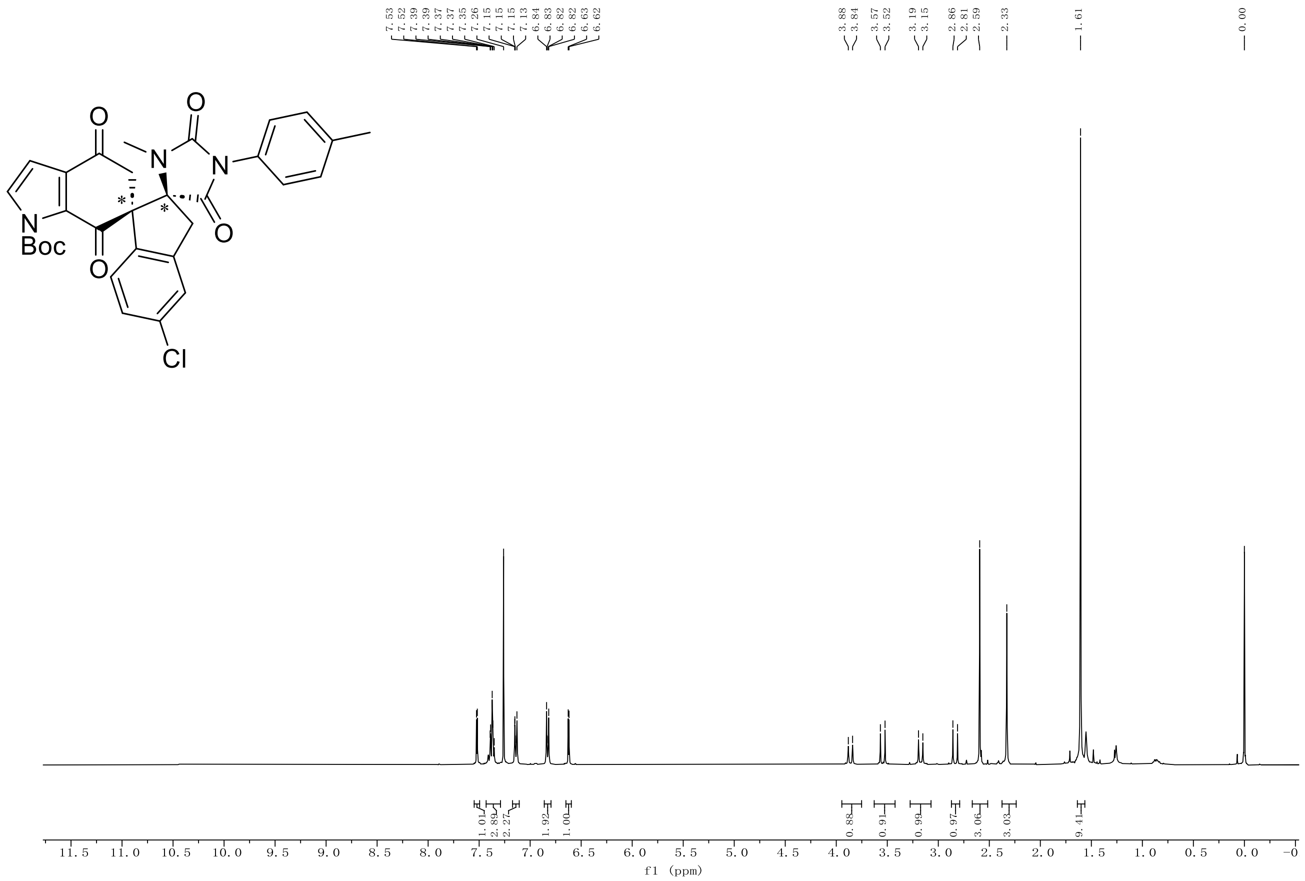

Figure S109: ^{1}H NMR spectra of compound **5'j**

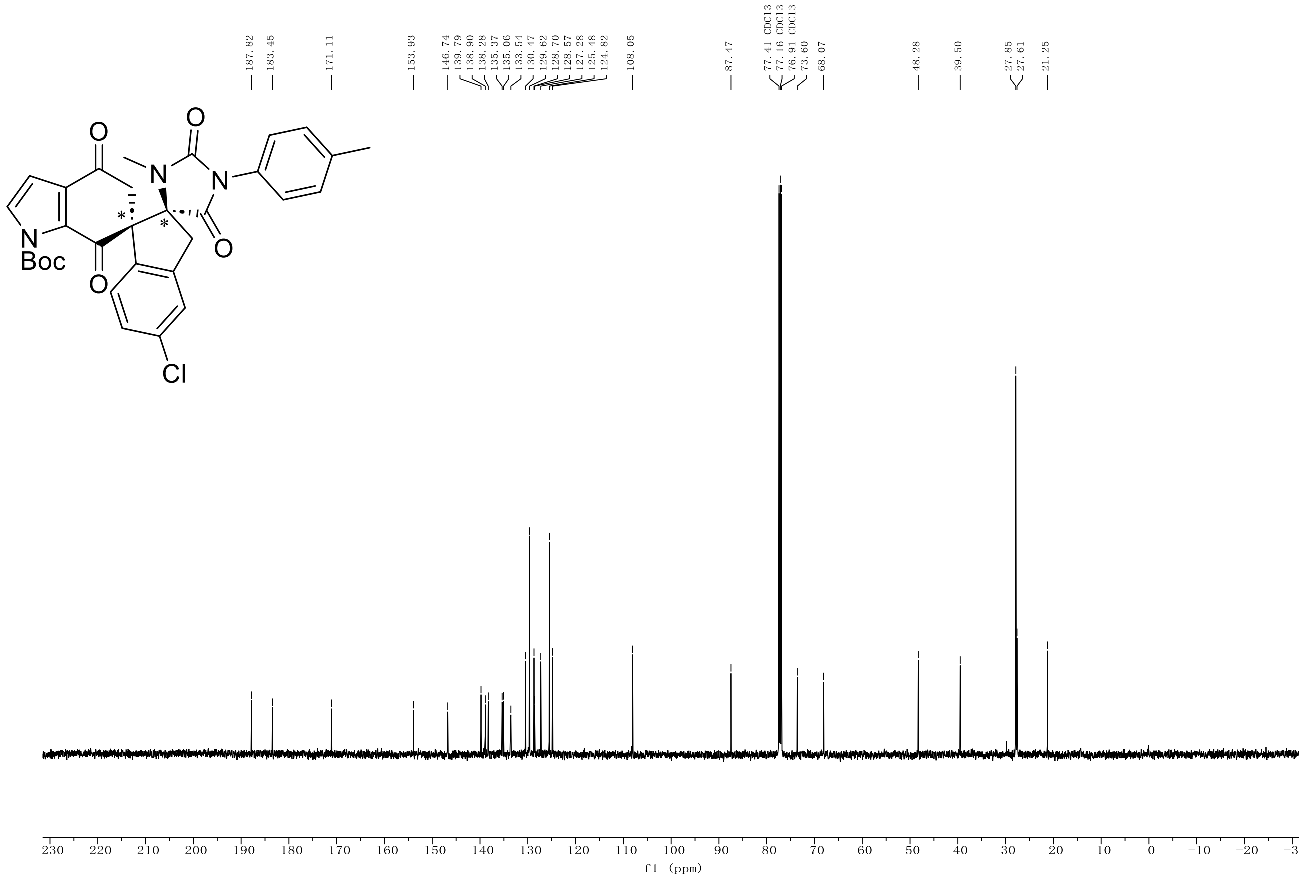

Figure S110: ^{13}C NMR spectra of compound **5'j**

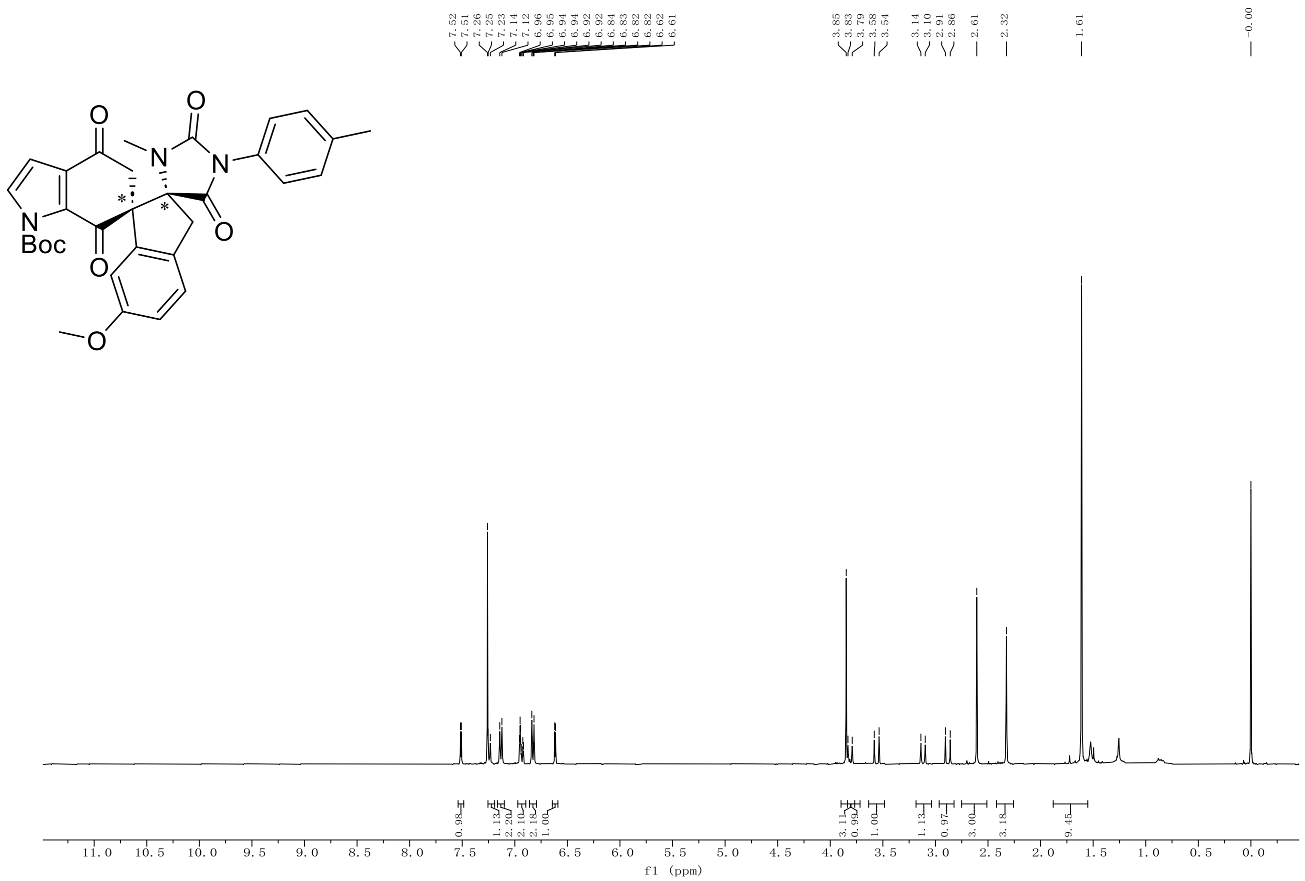

Figure S111: ^{1}H NMR spectra of compound **5k**

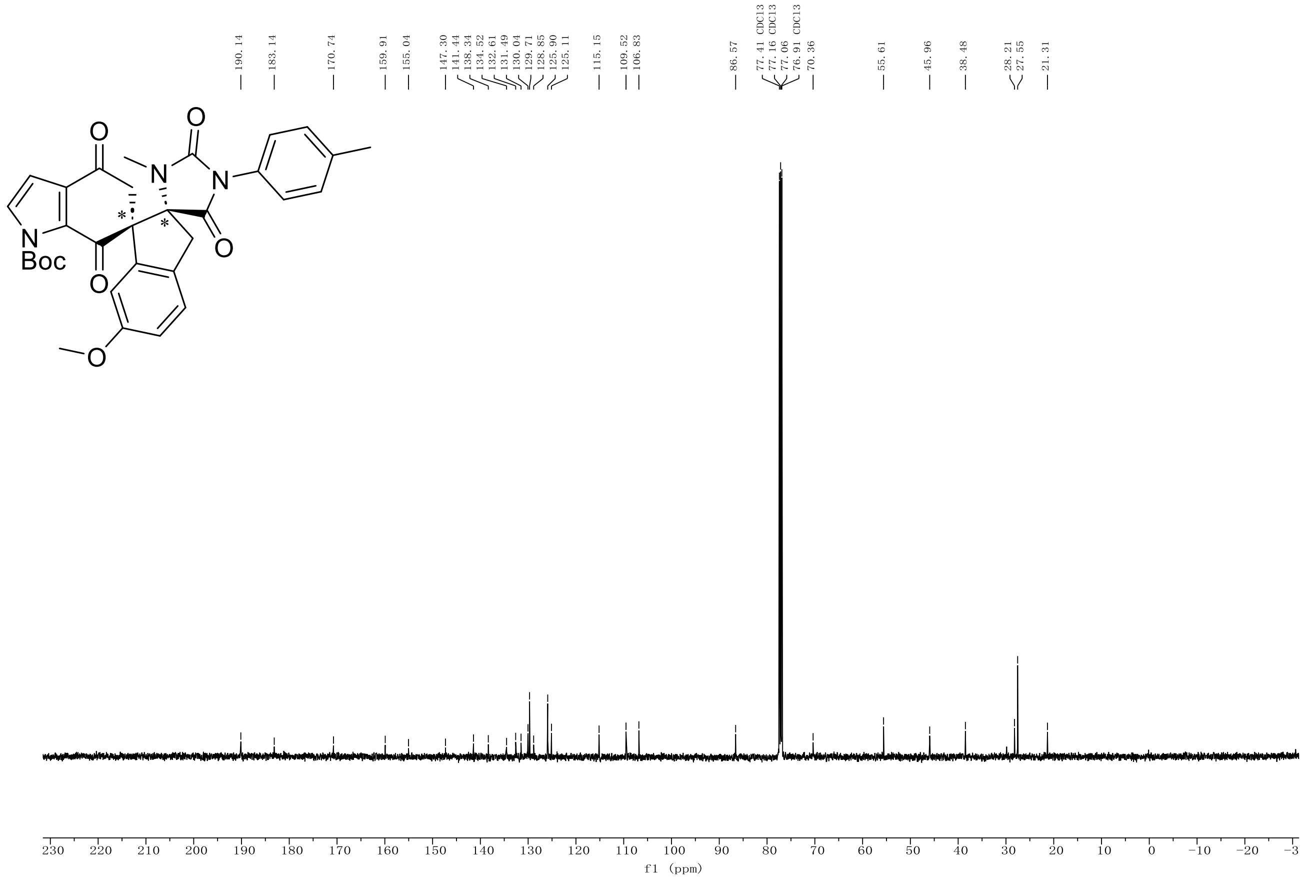

Figure S112: ^{13}C NMR spectra of compound **5k**

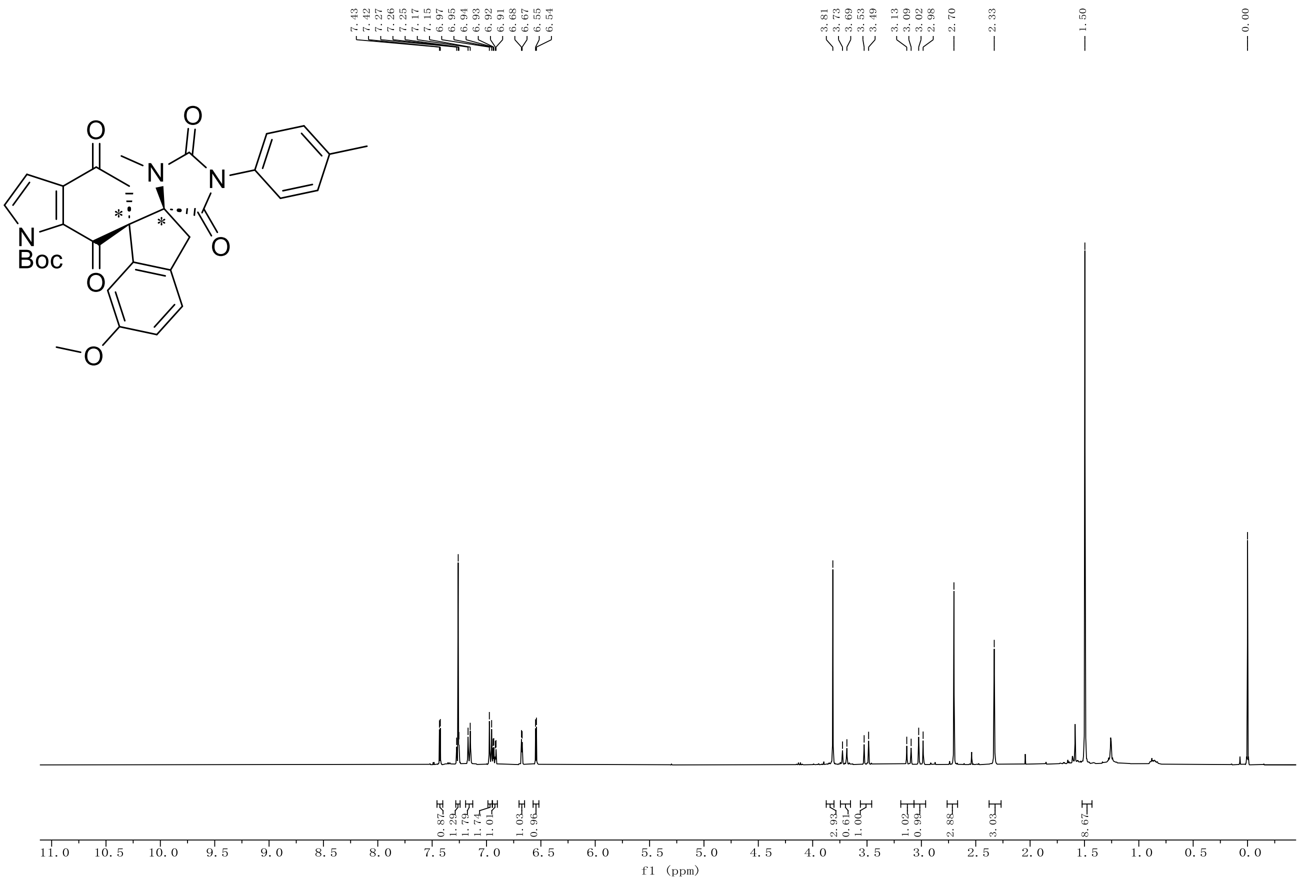

Figure S113: ^{1}H NMR spectra of compound **5'k**

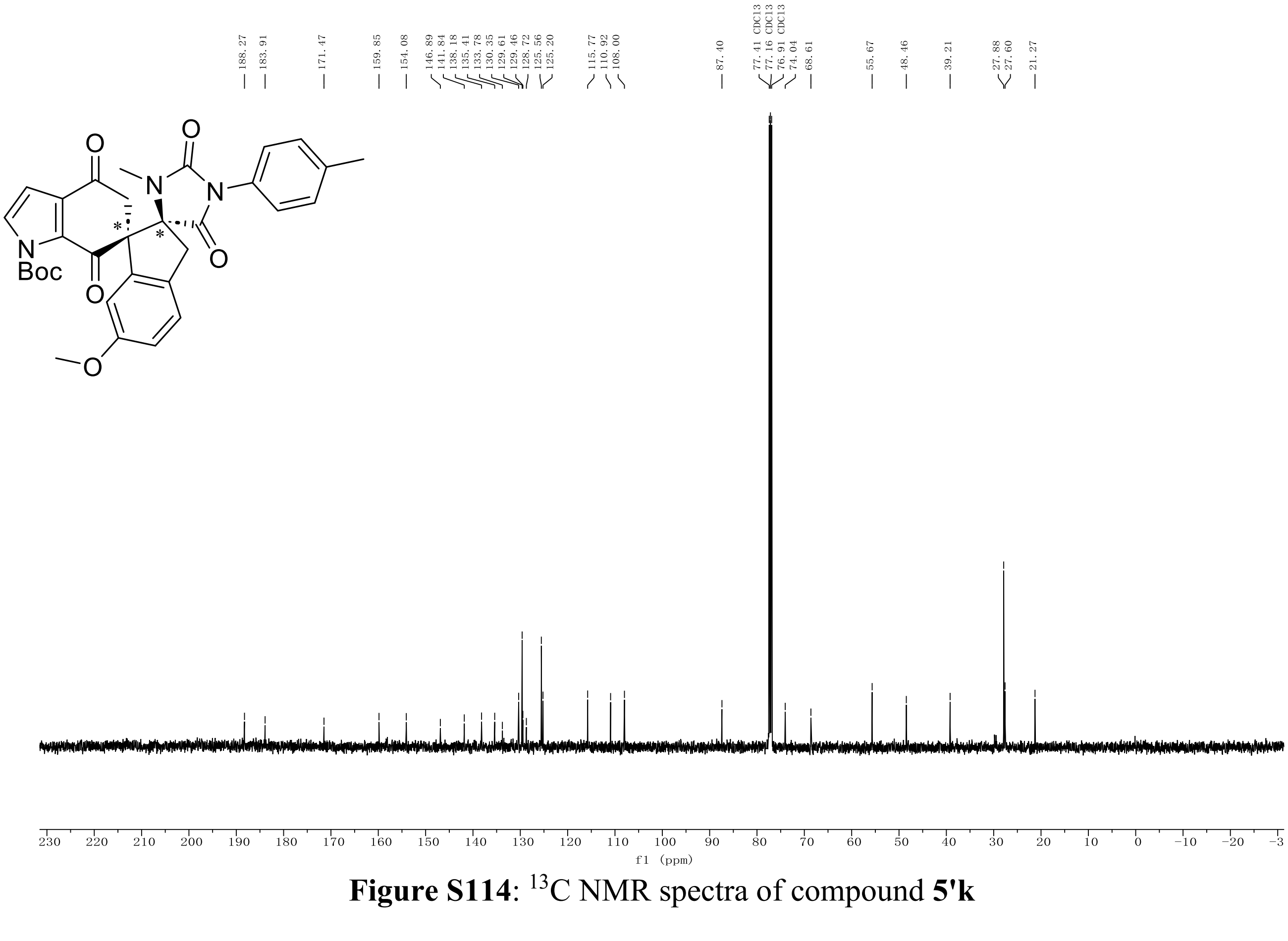

Figure S114: ^{13}C NMR spectra of compound **5'k**

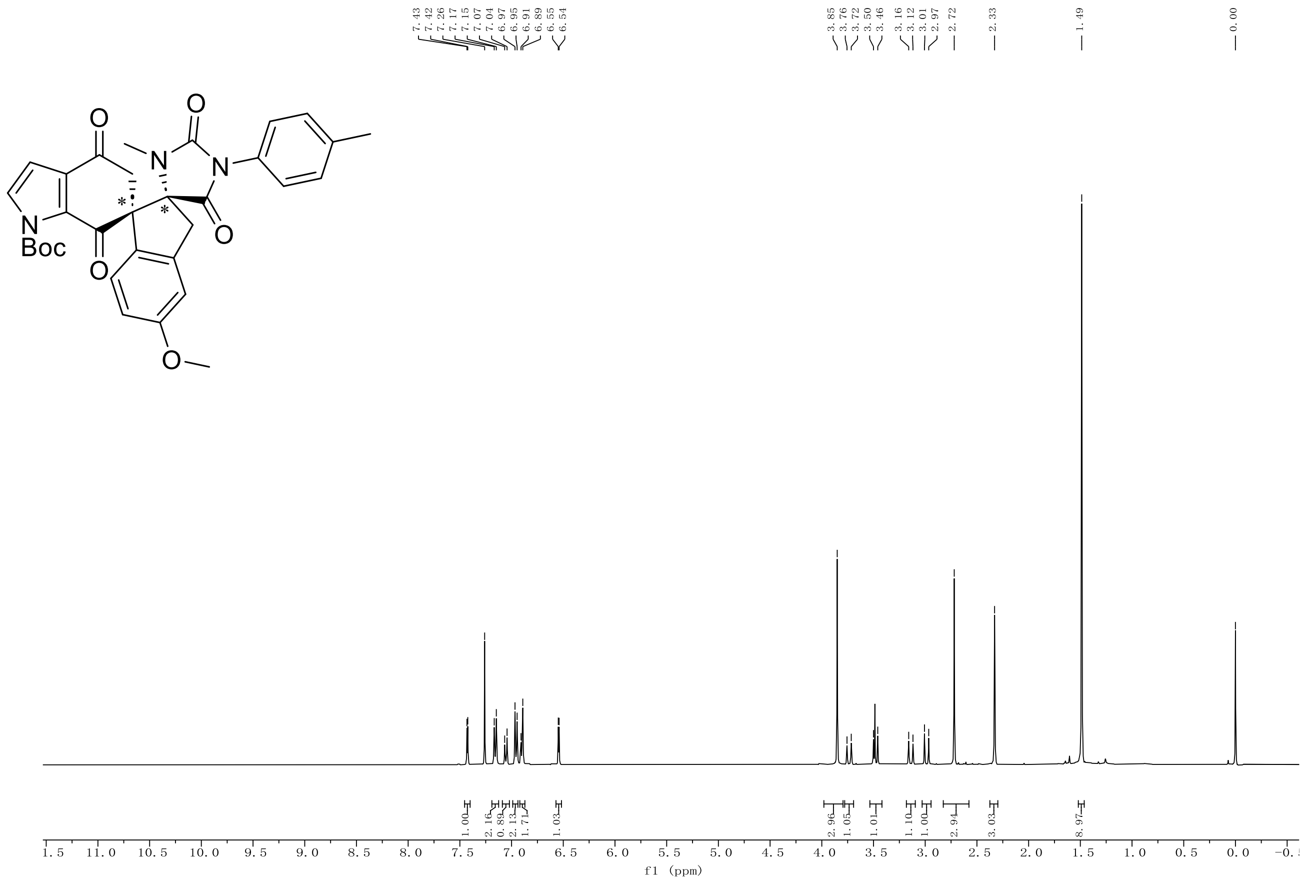

Figure S115: ^{1}H NMR spectra of compound **5l**

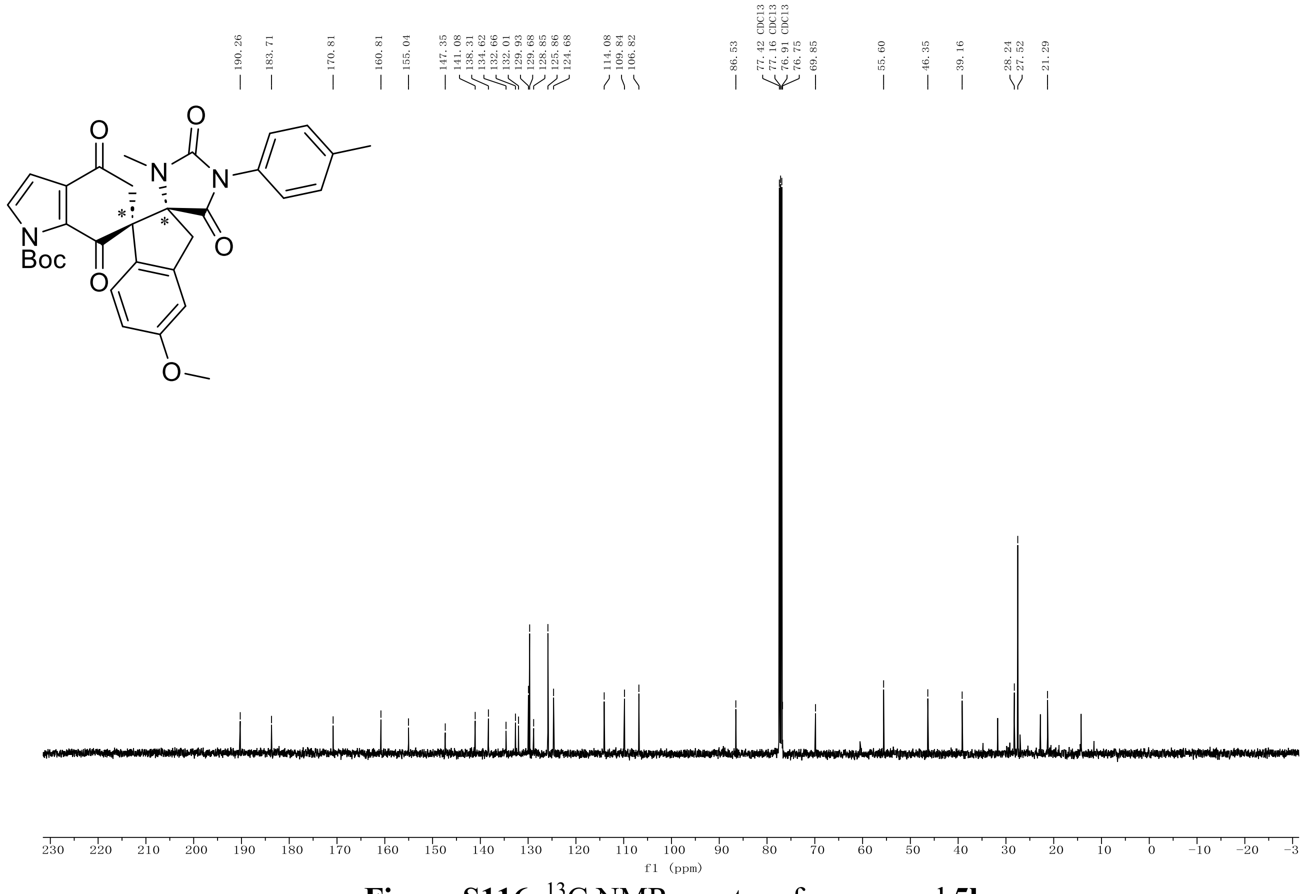

Figure S116: ^{13}C NMR spectra of compound **5l**

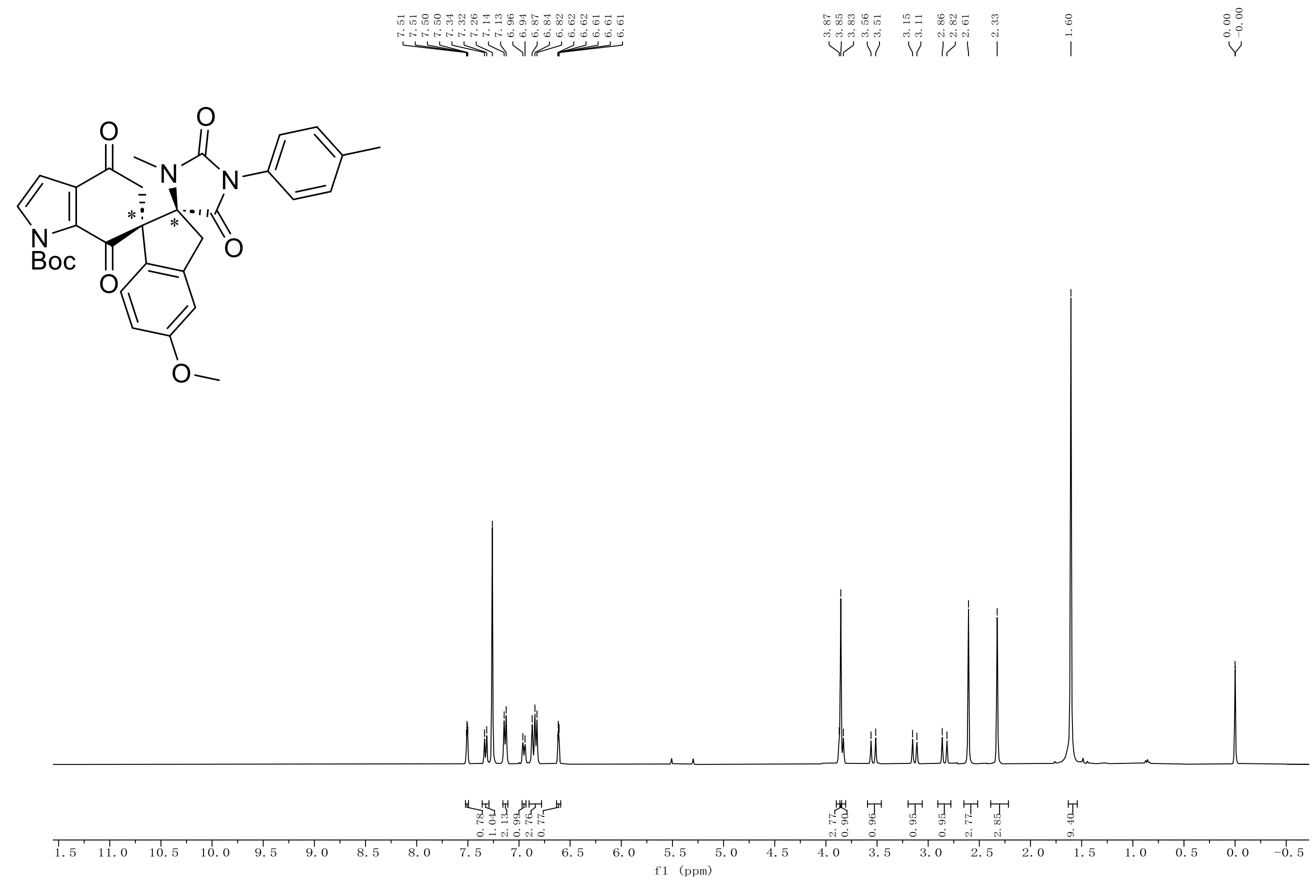

Figure S117: ^{1}H NMR spectra of compound **5'l**

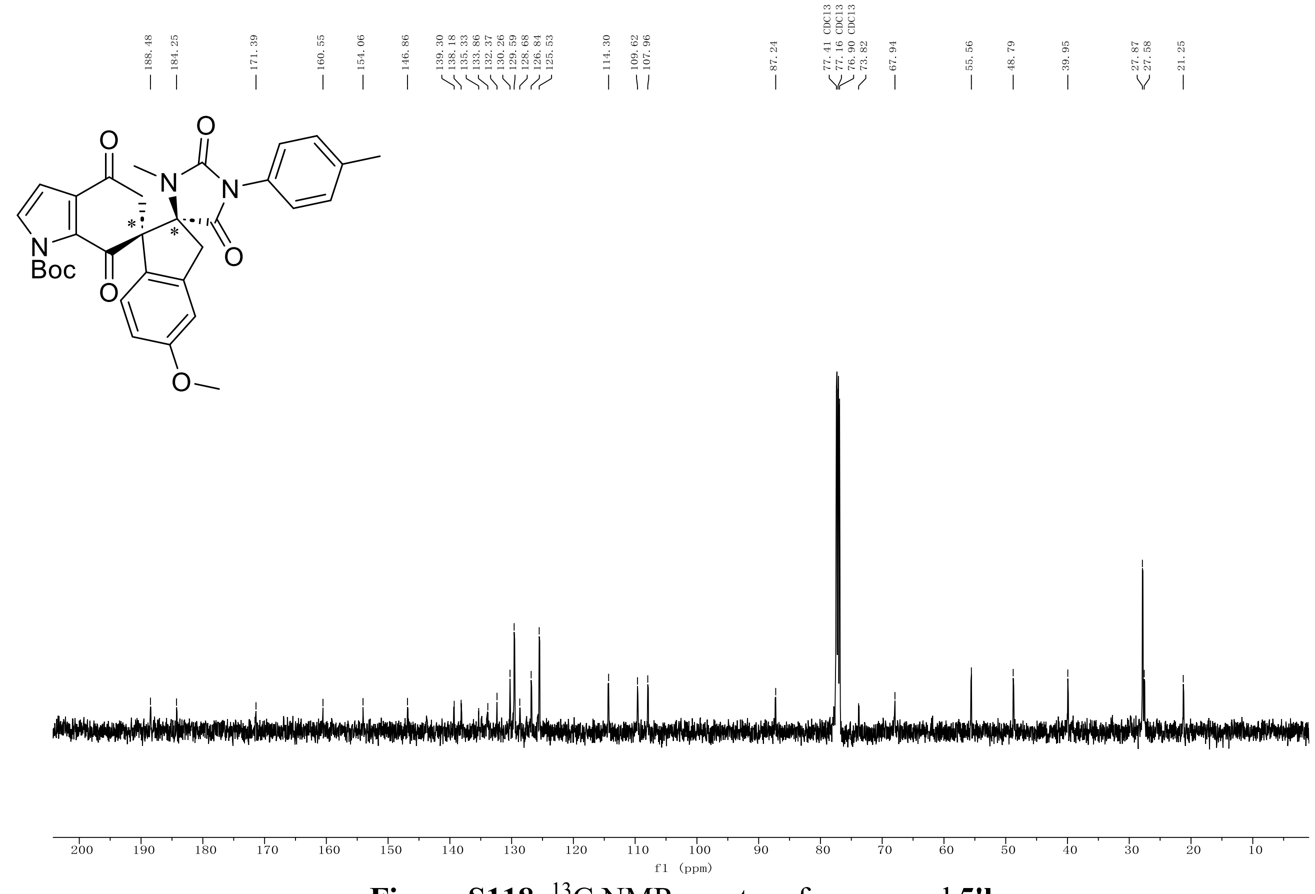

Figure S118: ^{13}C NMR spectra of compound **5'l**

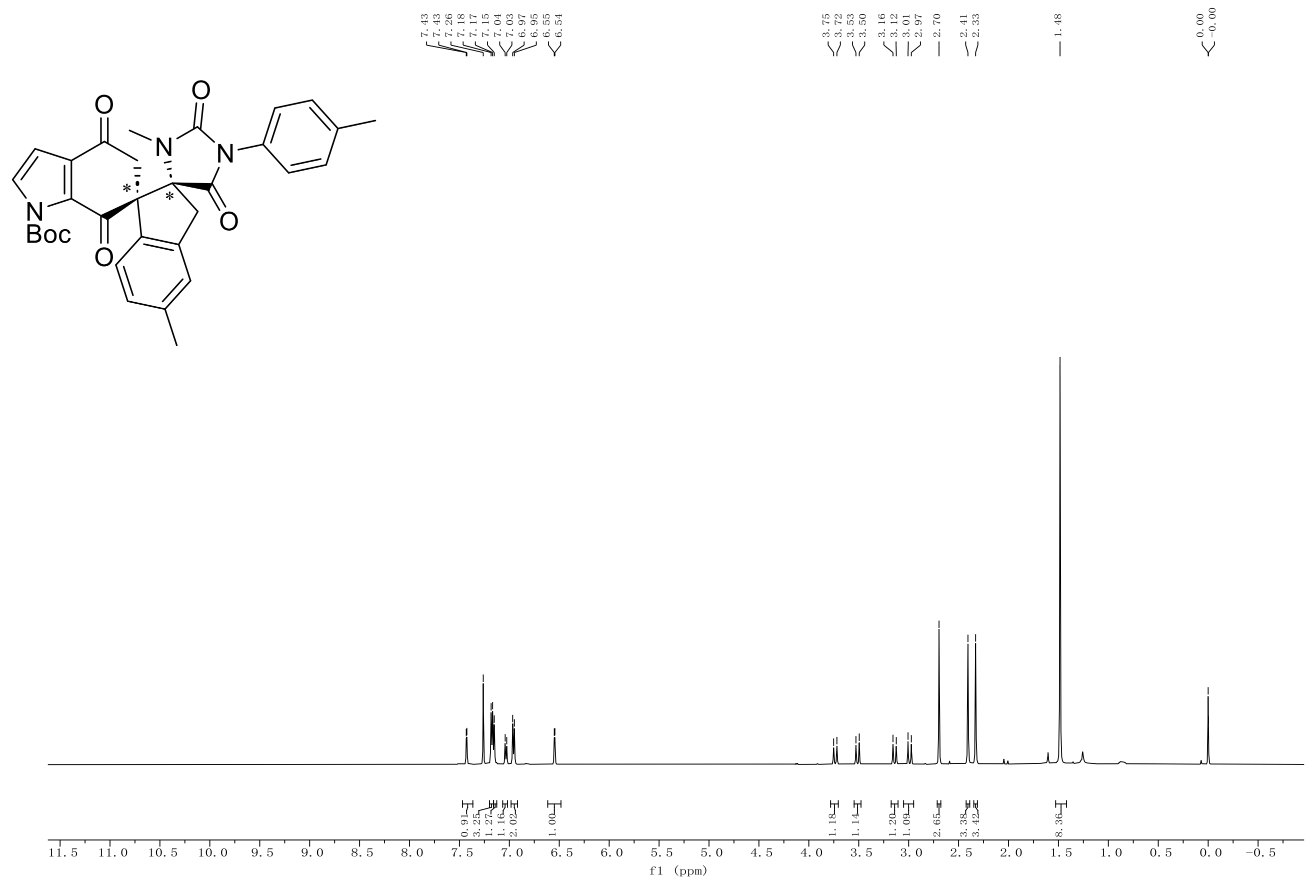

Figure S119: ^{1}H NMR spectra of compound **5m**

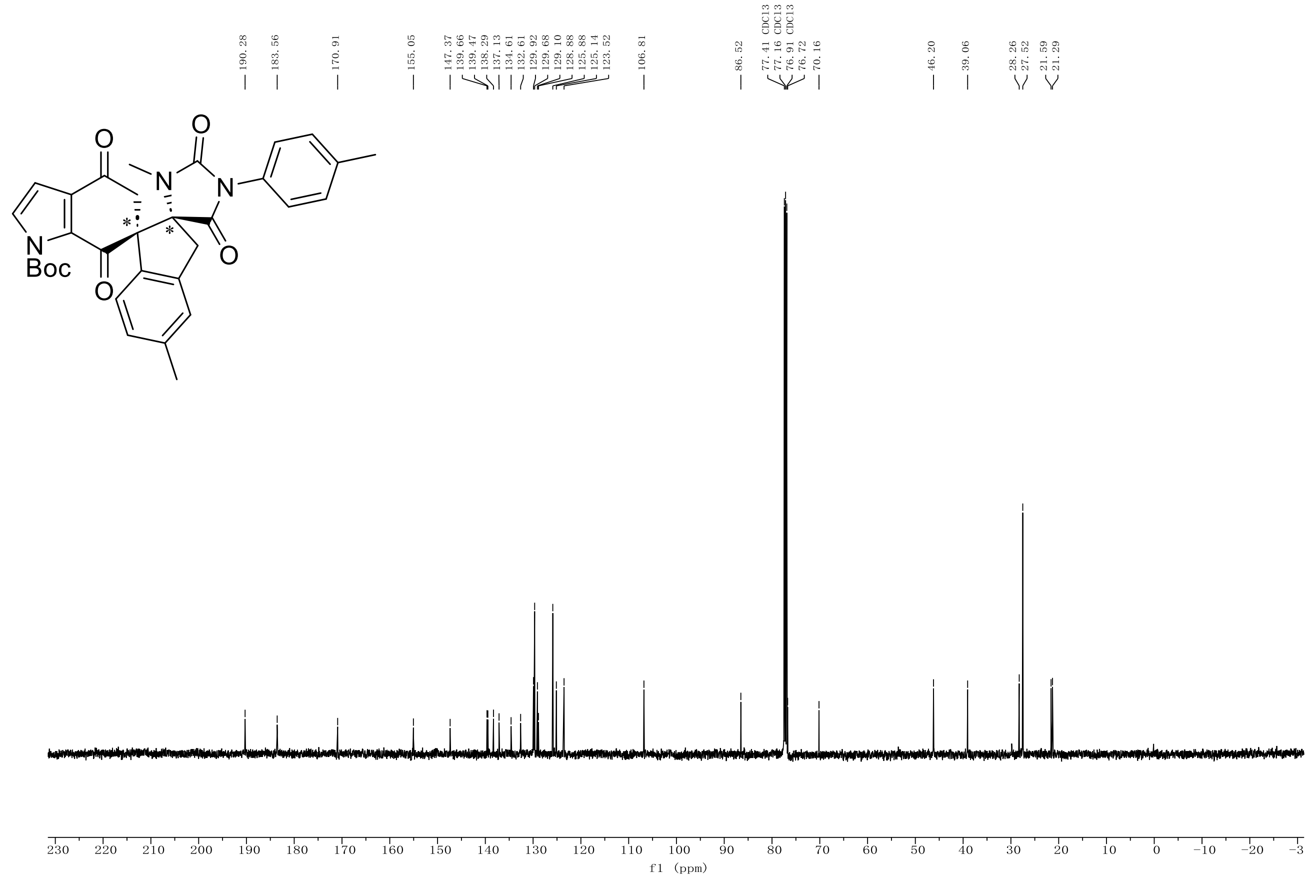

Figure S120: ^{13}C NMR spectra of compound **5m**

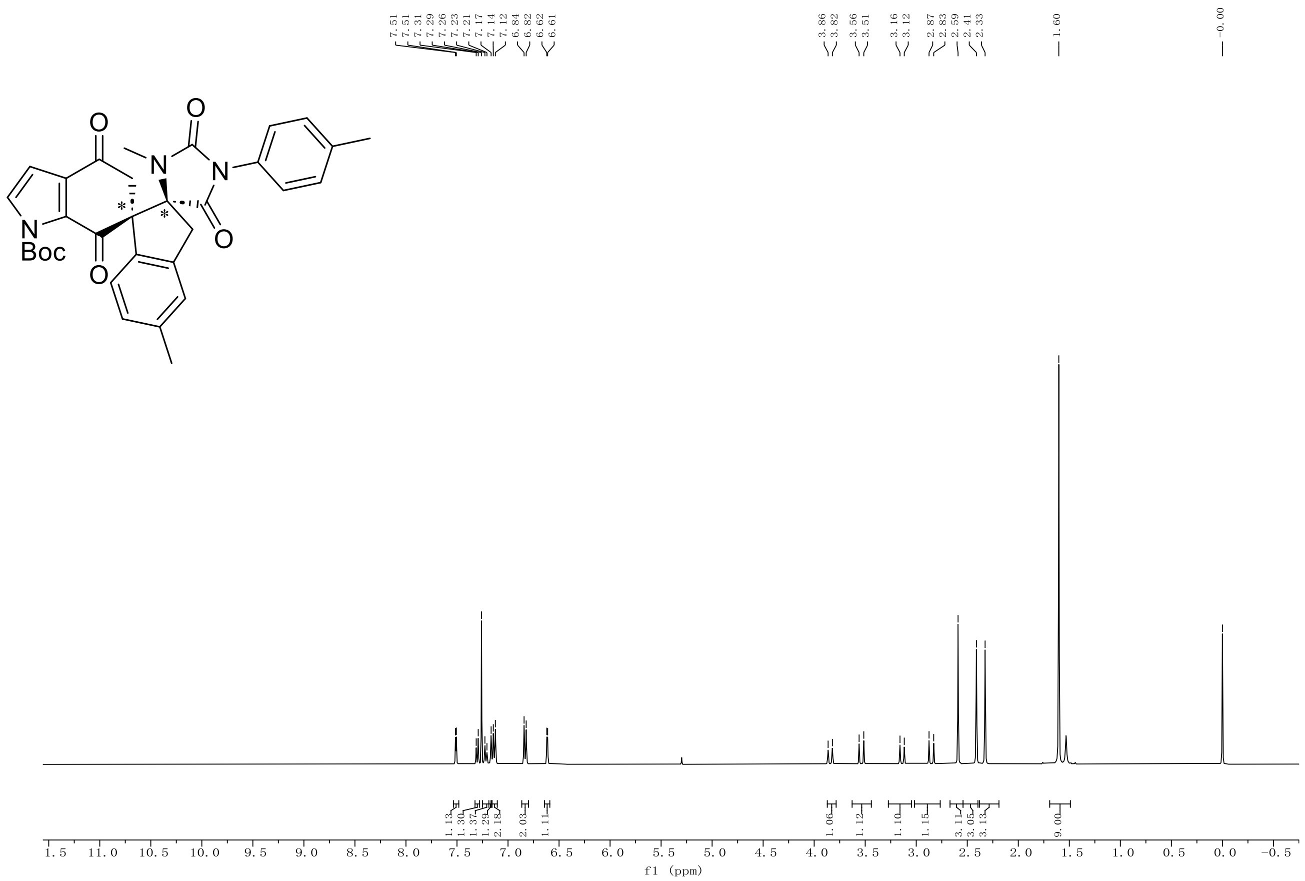

Figure S121: ^{1}H NMR spectra of compound **5'm**

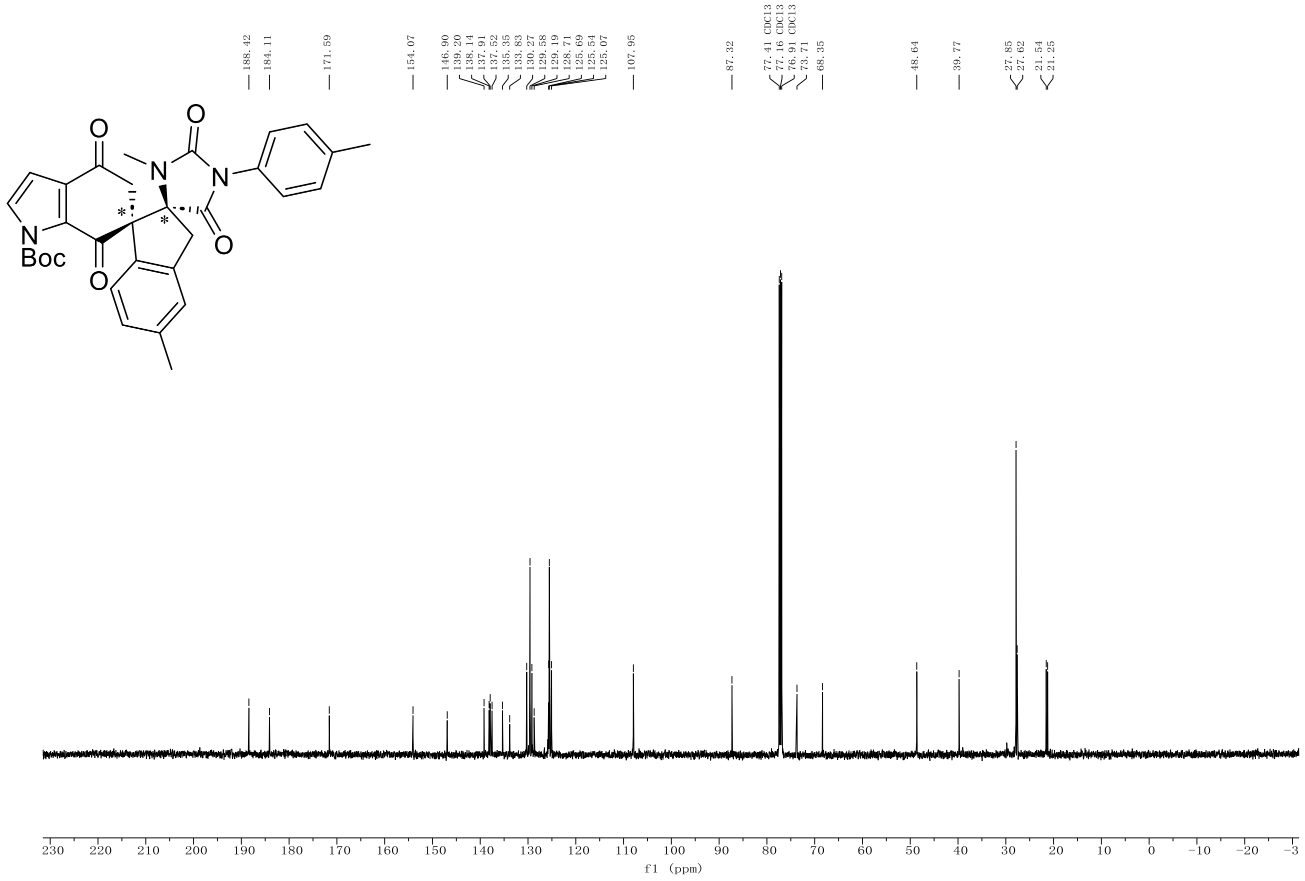

Figure S122: ^{13}C NMR spectra of compound **5'm**

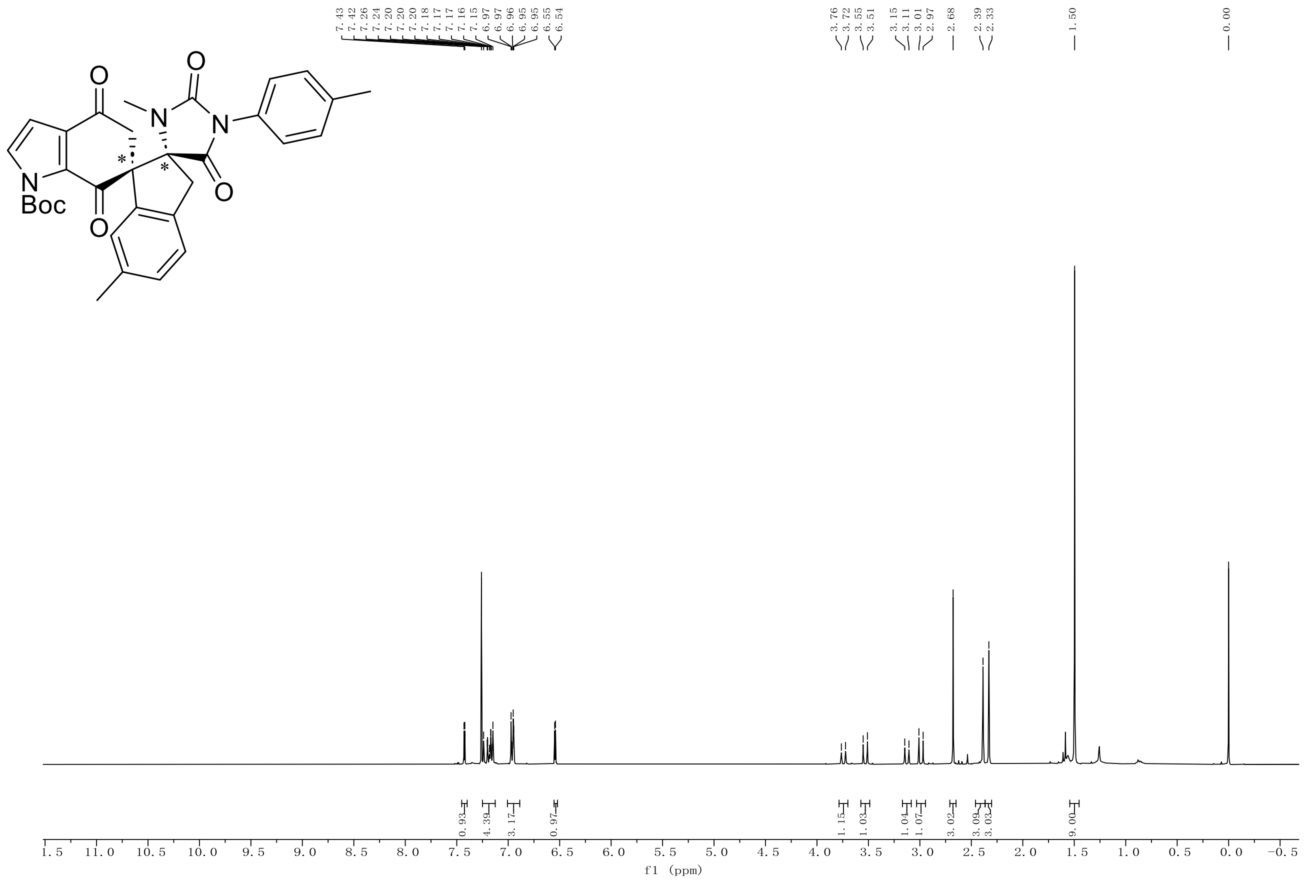

Figure S123: ^{1}H NMR spectra of compound **5n**

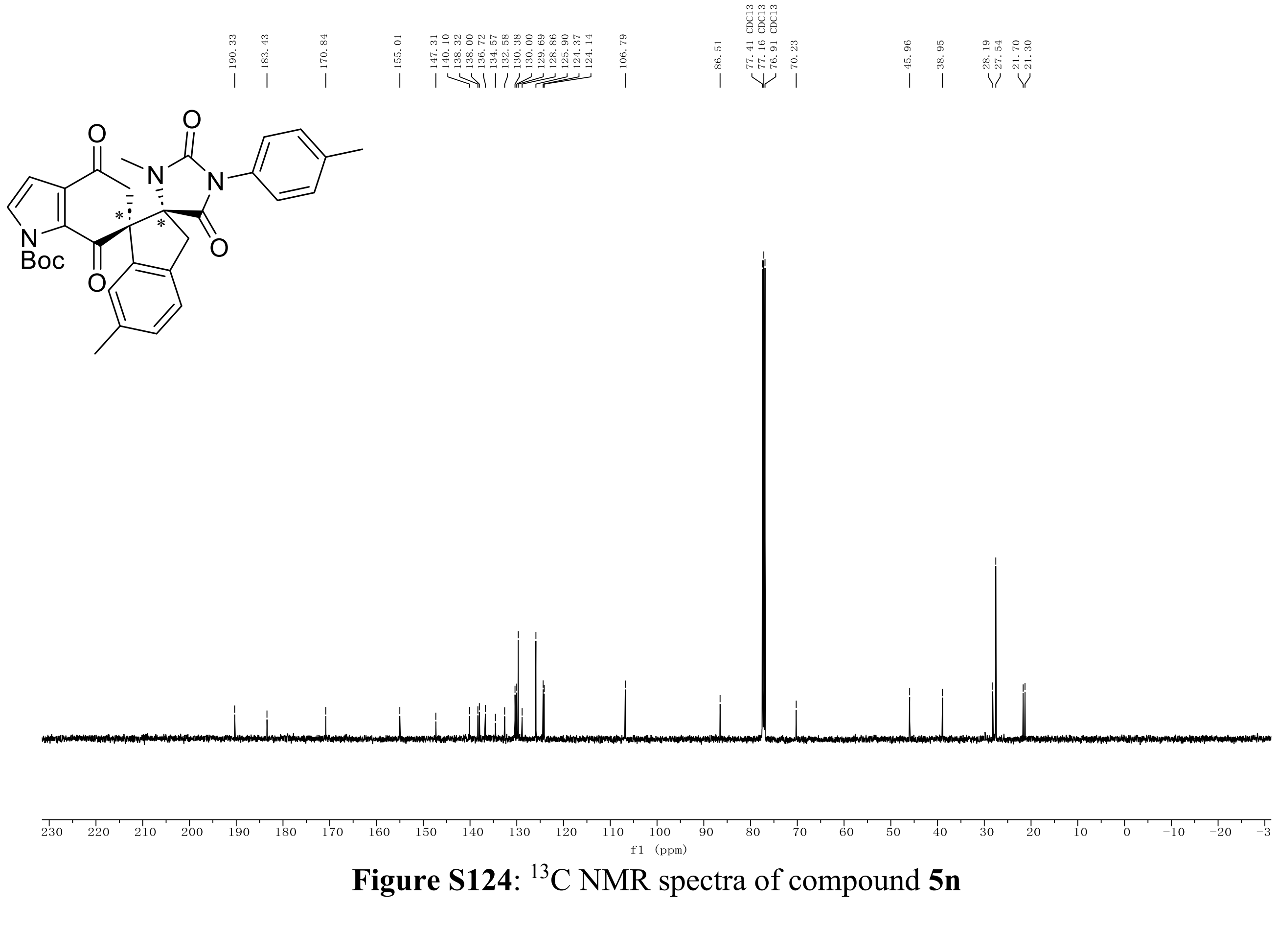

Figure S124: ^{13}C NMR spectra of compound **5n**

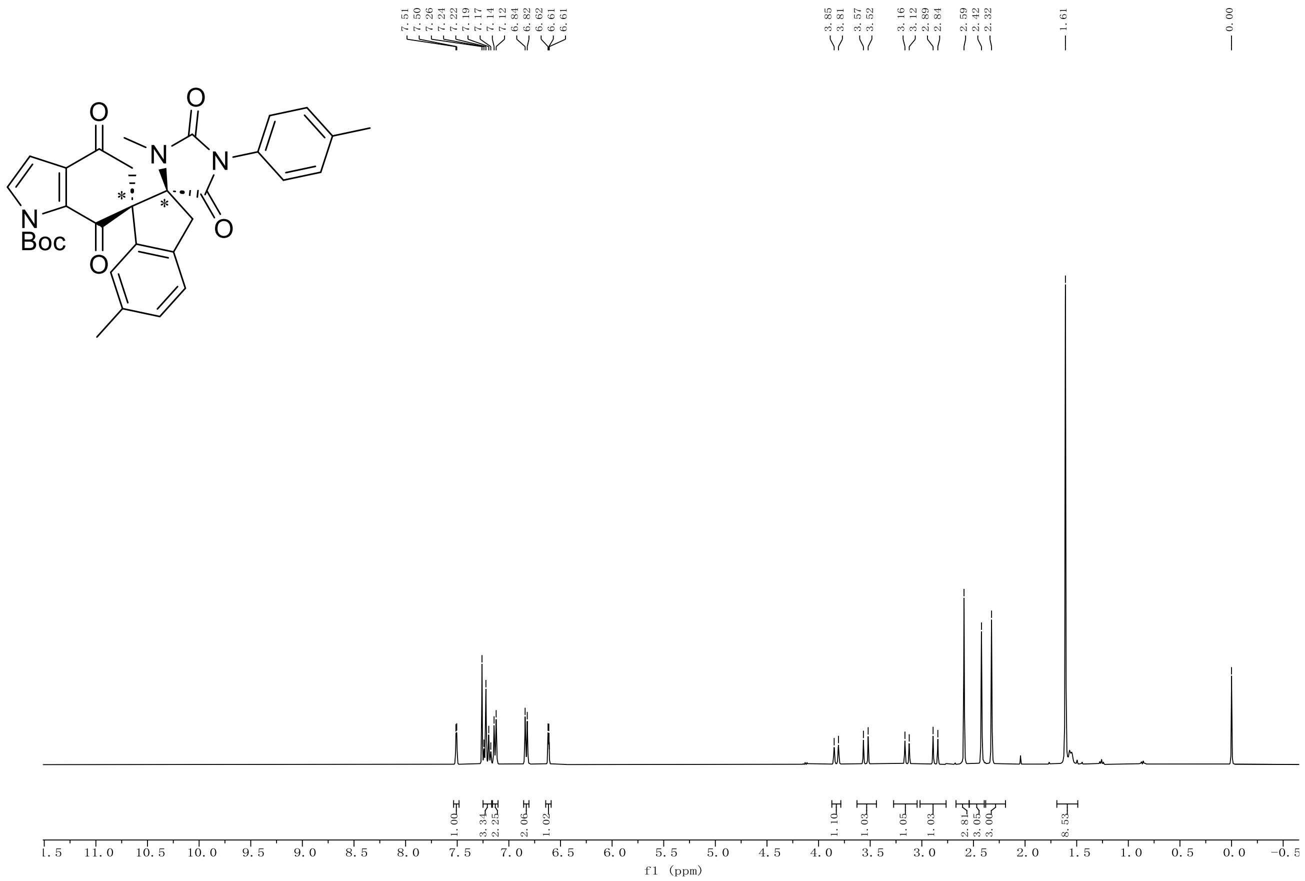

Figure S125: ^{1}H NMR spectra of compound **5'n**

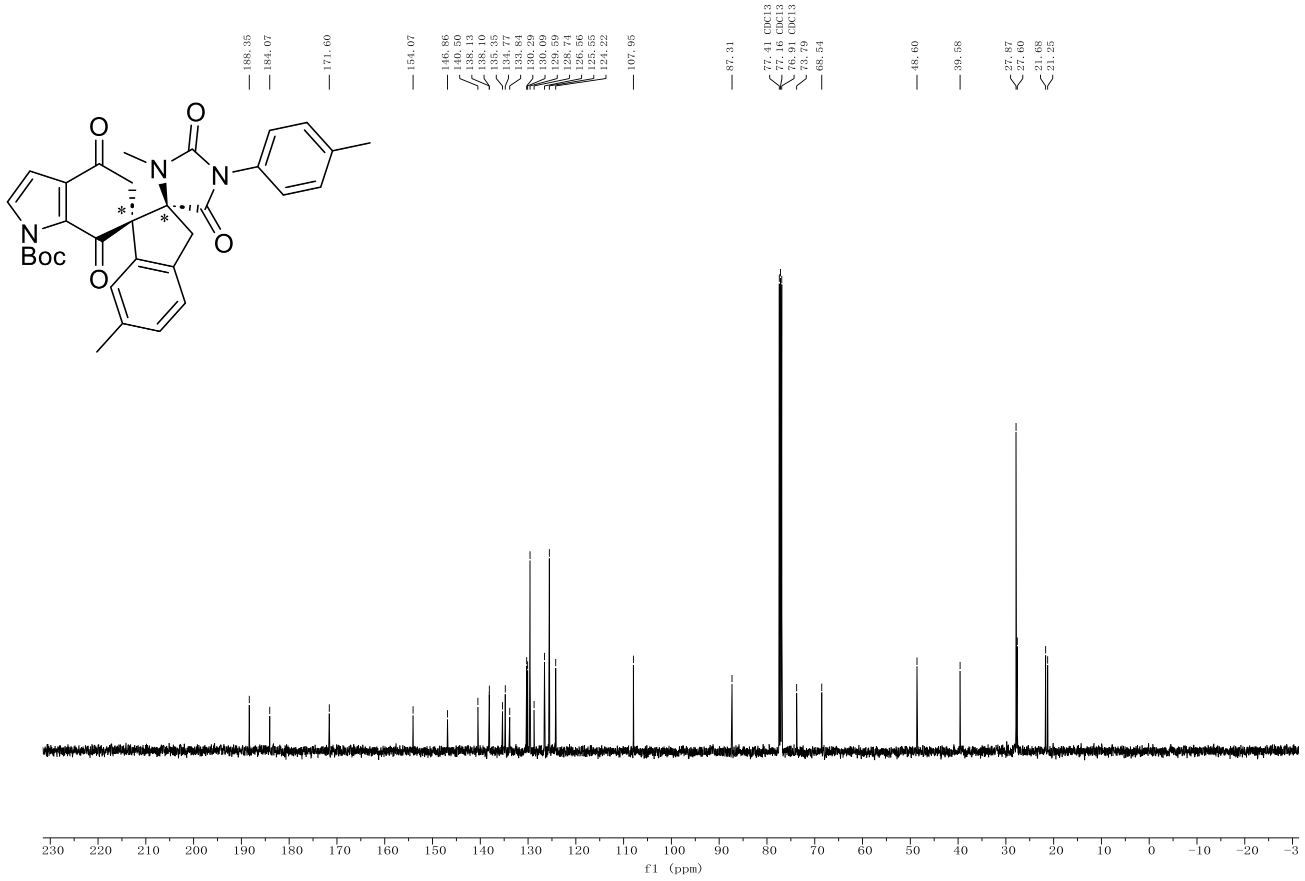

Figure S126: ^{13}C NMR spectra of compound **5'n**

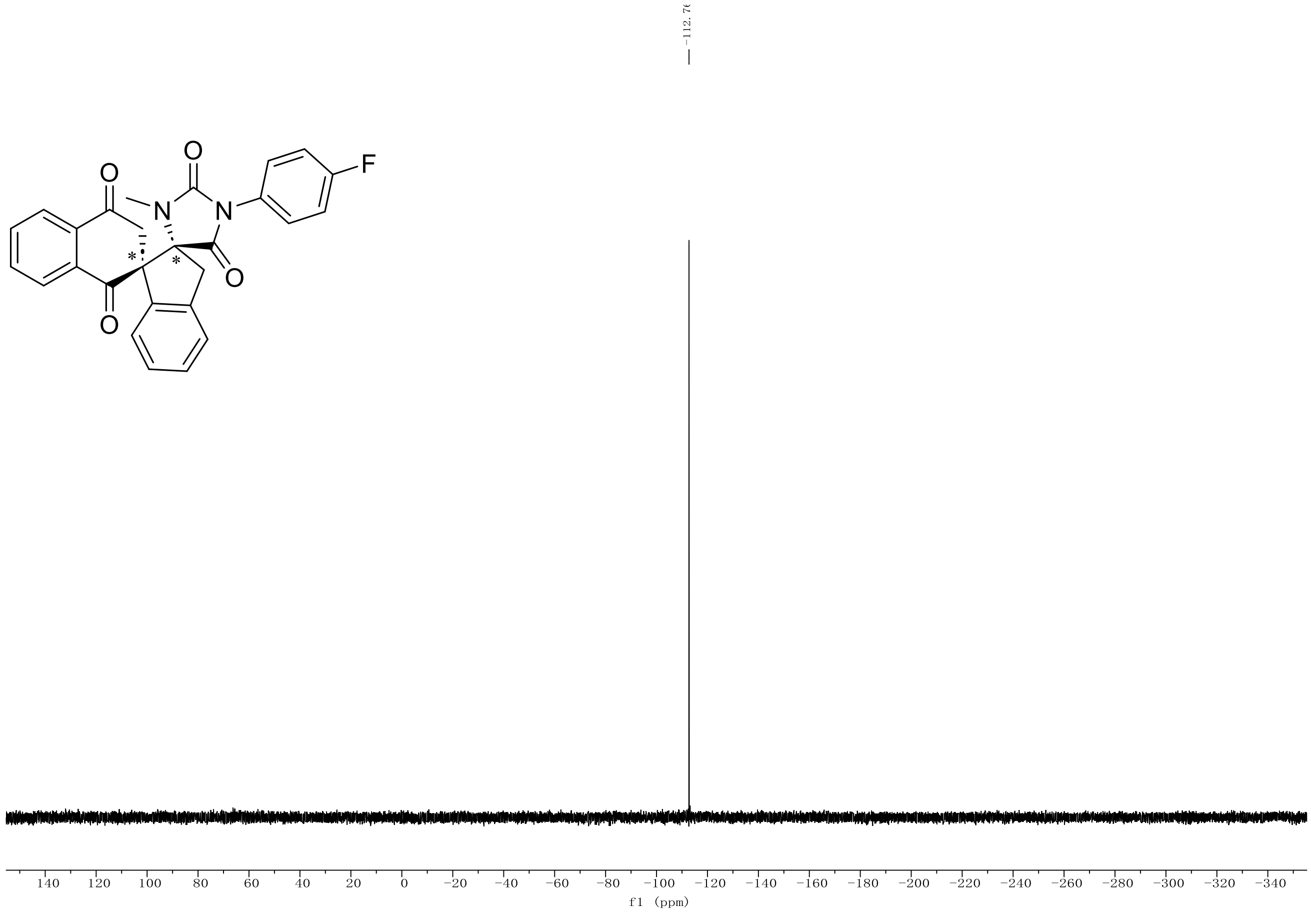

Figure S127: ^{19}F{H} NMR spectra of compound **2e**

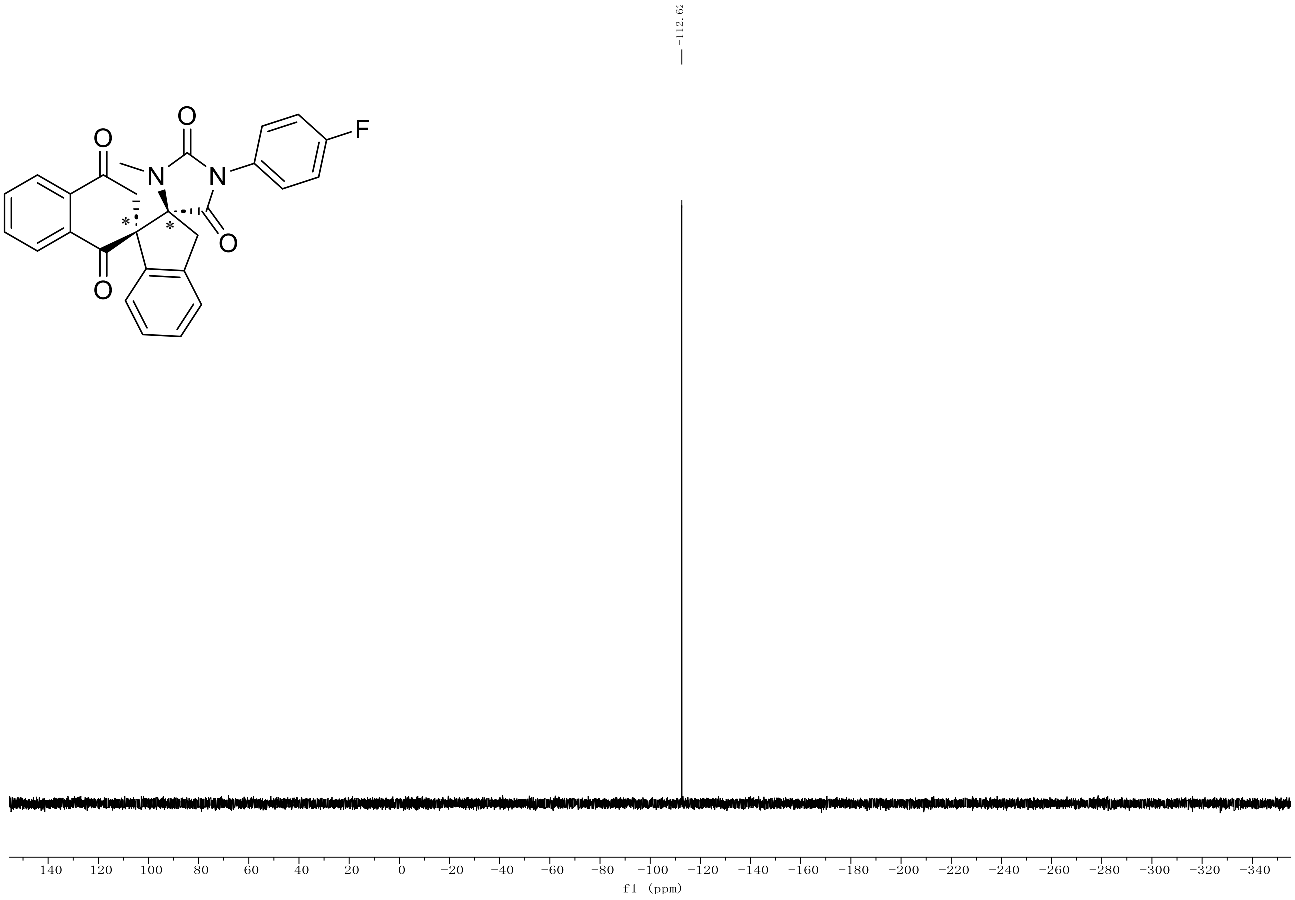

Figure S128: ^{19}F{H} NMR spectra of compound **2'e**

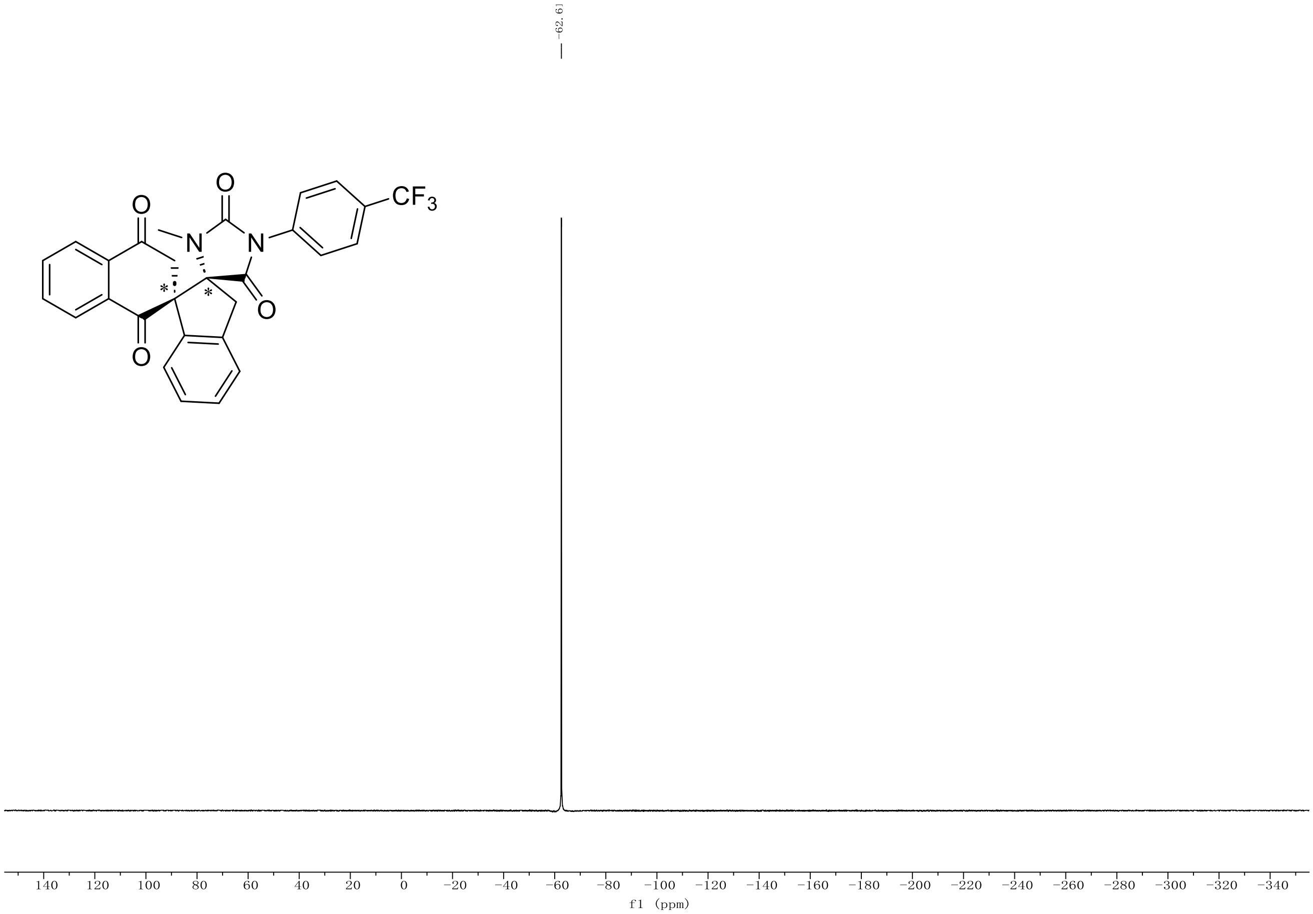

Figure S129: ^{19}F{H} NMR spectra of compound **2f**

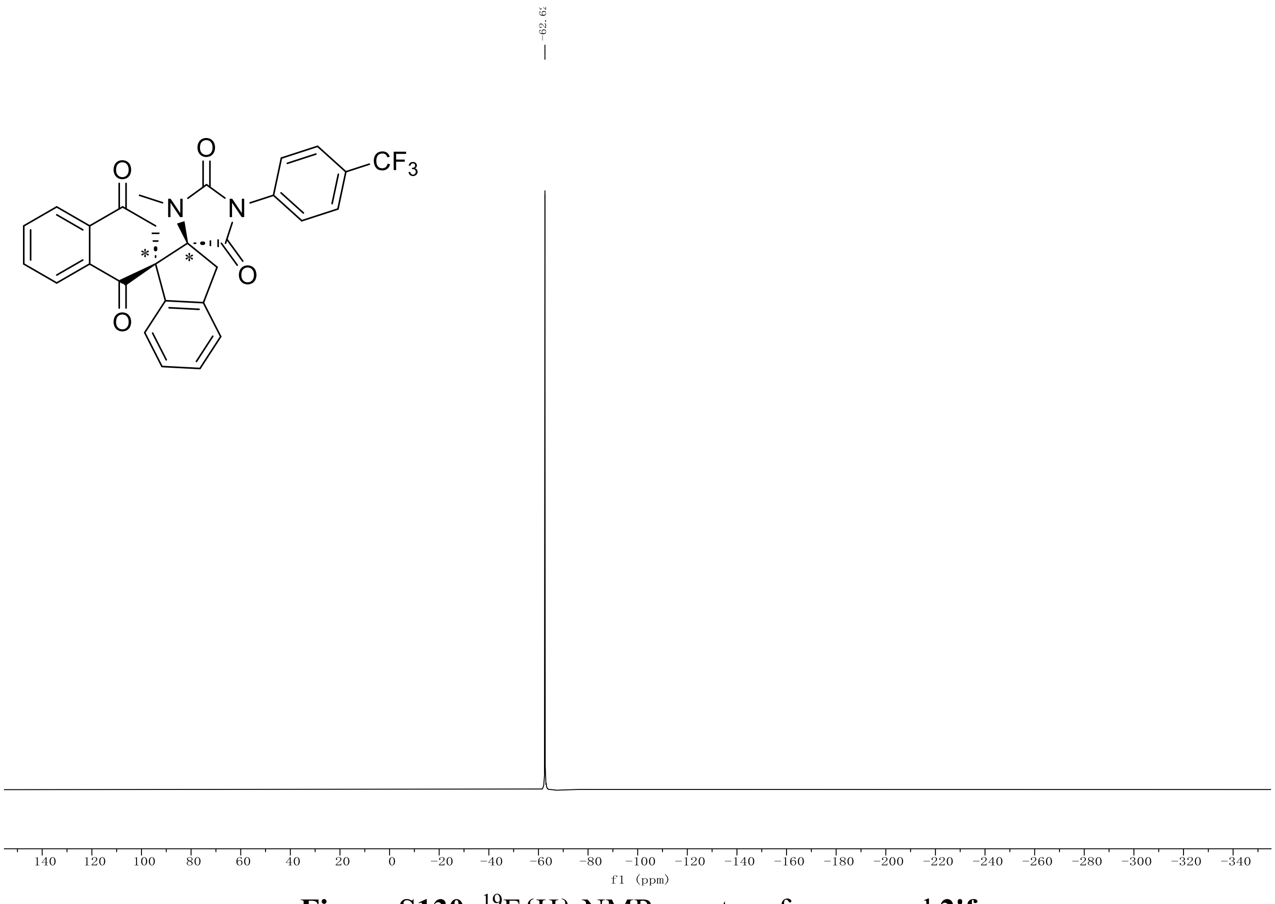

Figure S130: ^{19}F{H} NMR spectra of compound **2'f**

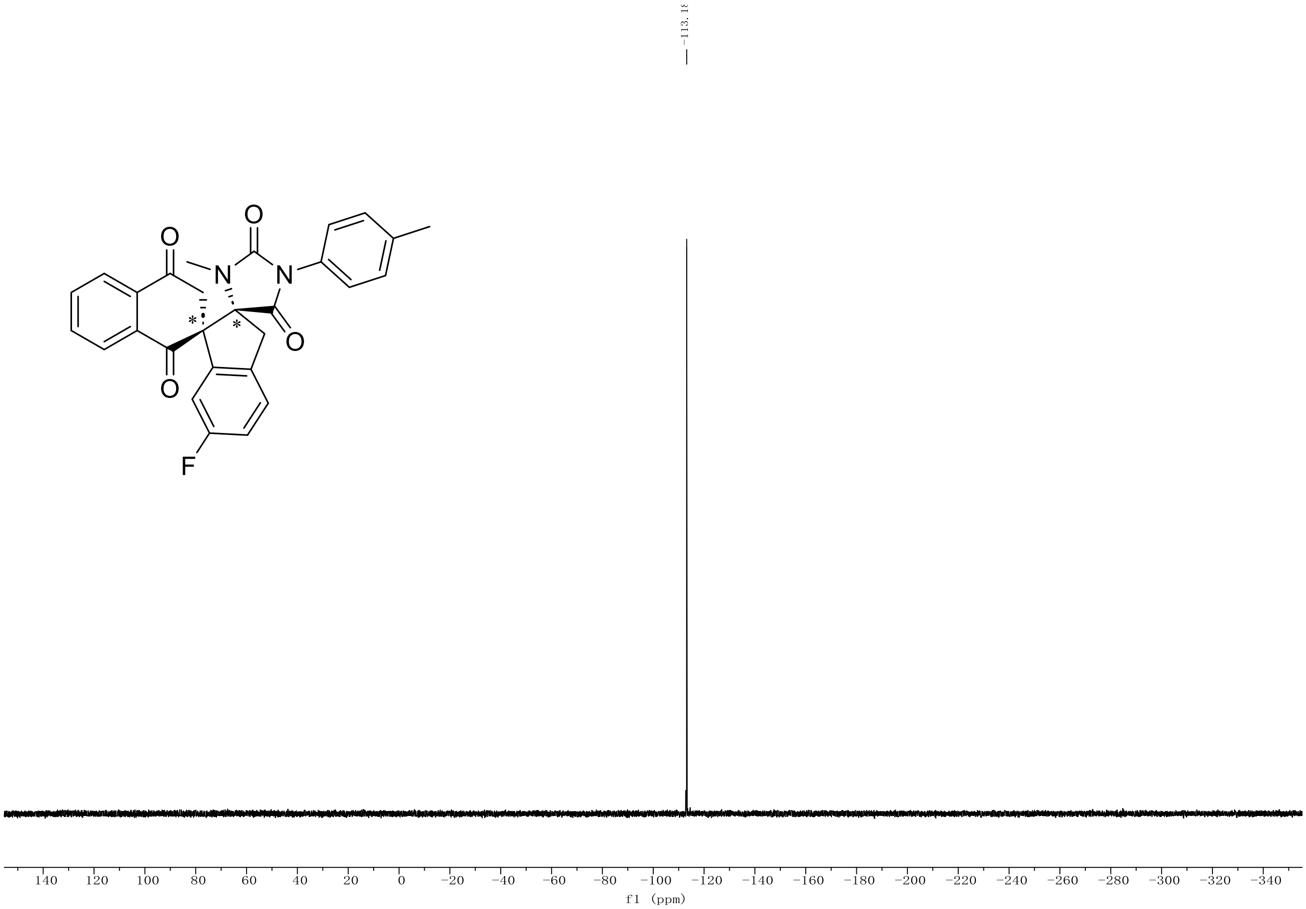

Figure S131: $^{19}F\{H\}$ NMR spectra of compound **2p**

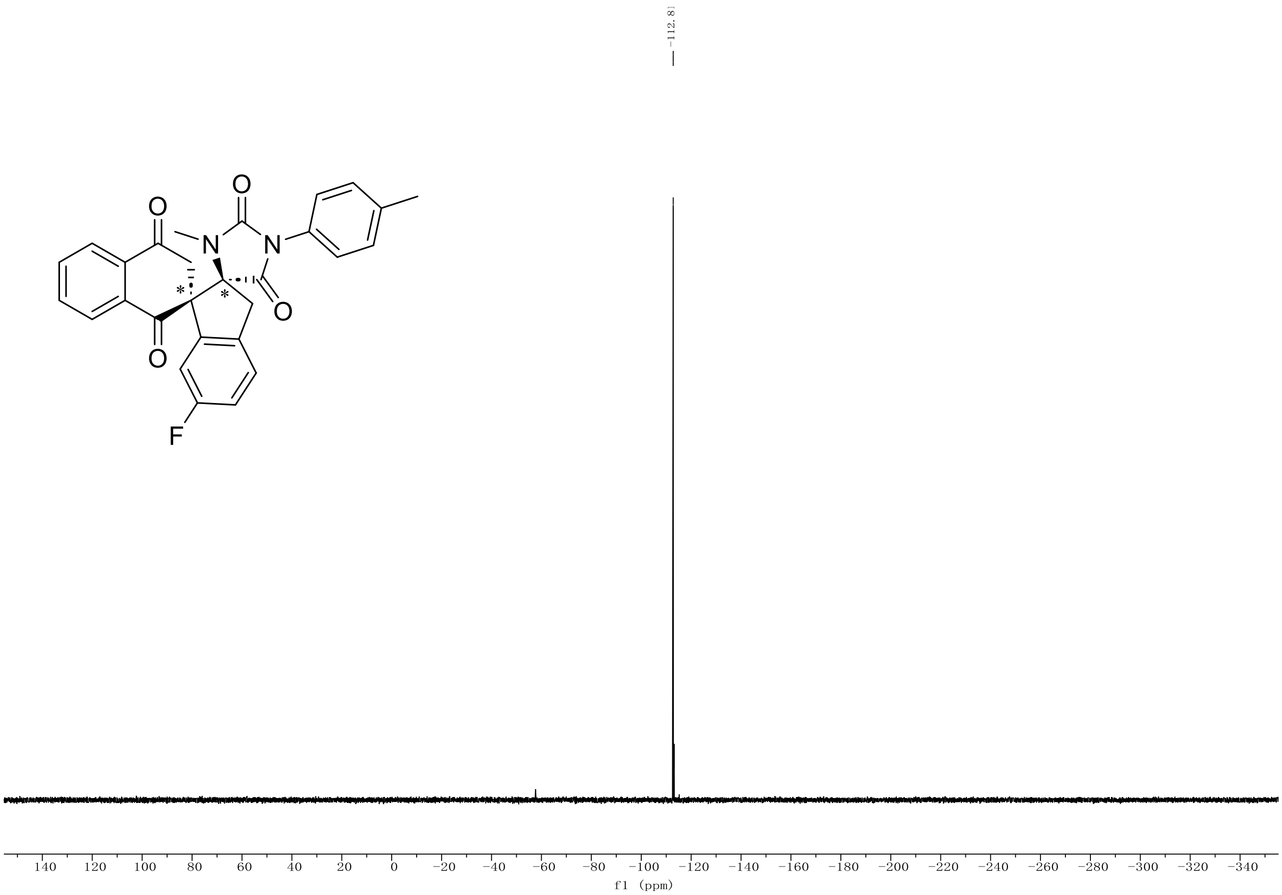

Figure S132: $^{19}F\{H\}$ NMR spectra of compound **2'p**

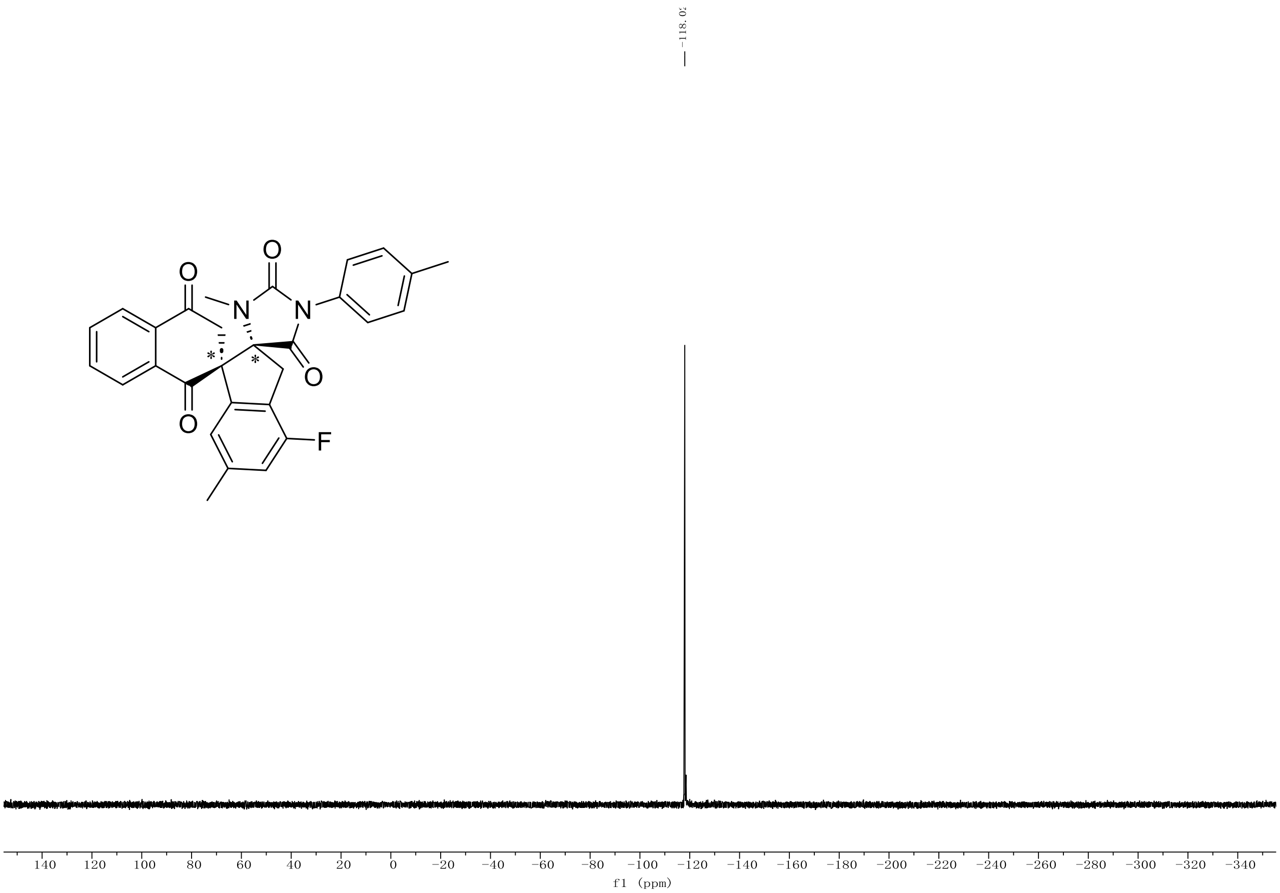

Figure S133: $^{19}F\{H\}$ NMR spectra of compound **2r**

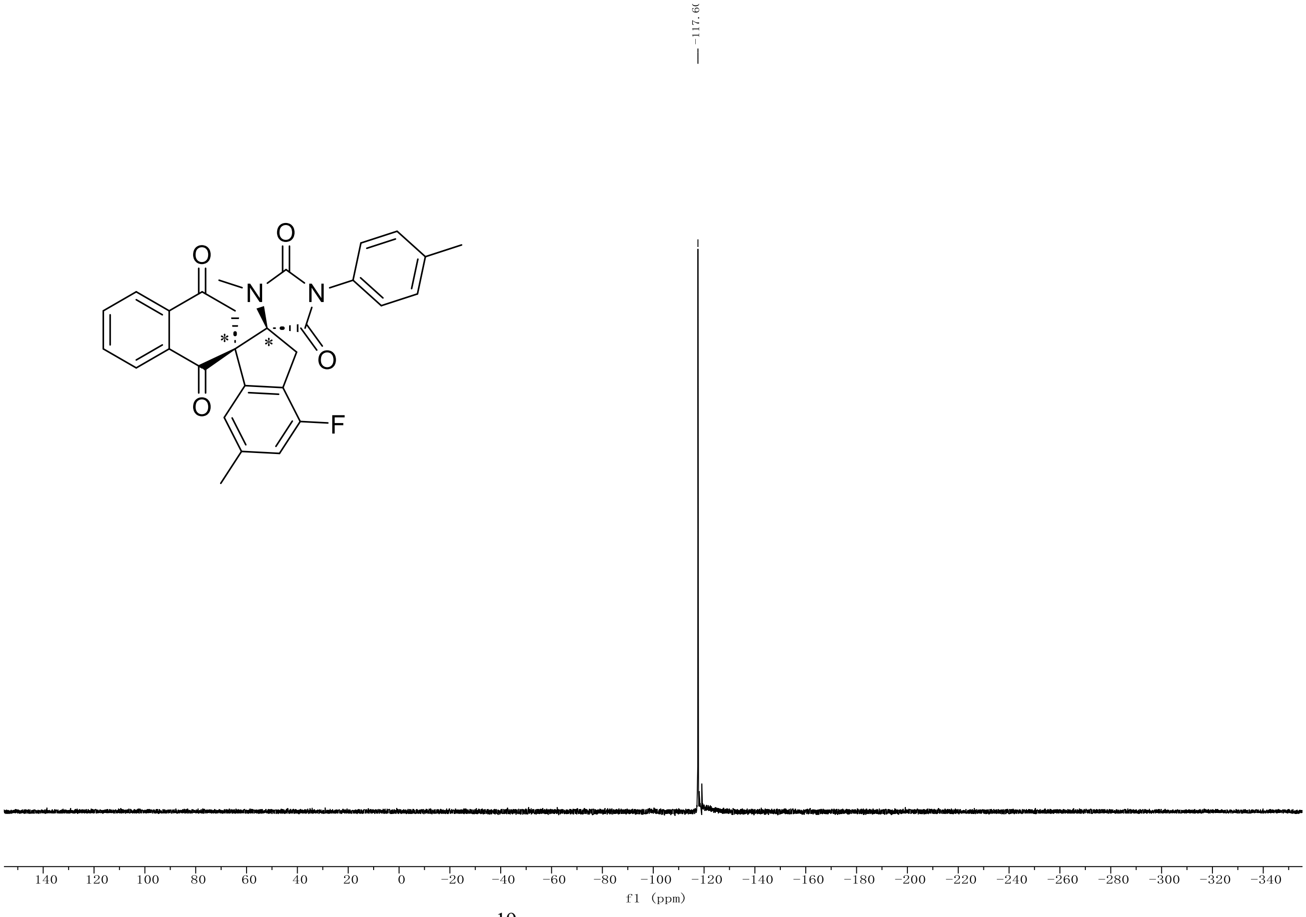

Figure S134: $^{19}F\{H\}$ NMR spectra of compound **2'r**

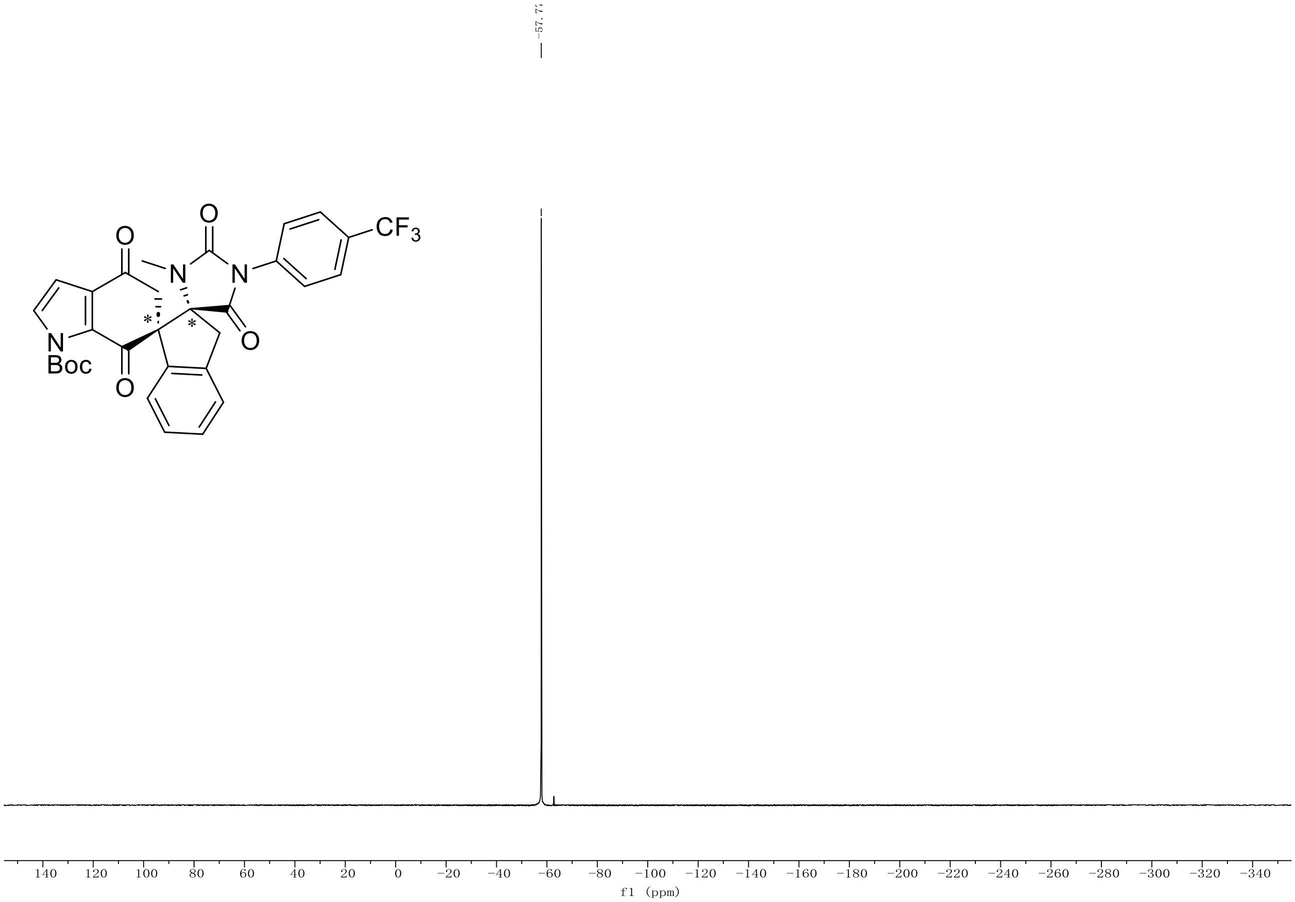

Figure S135: ^{19}F{H} NMR spectra of compound **5a**

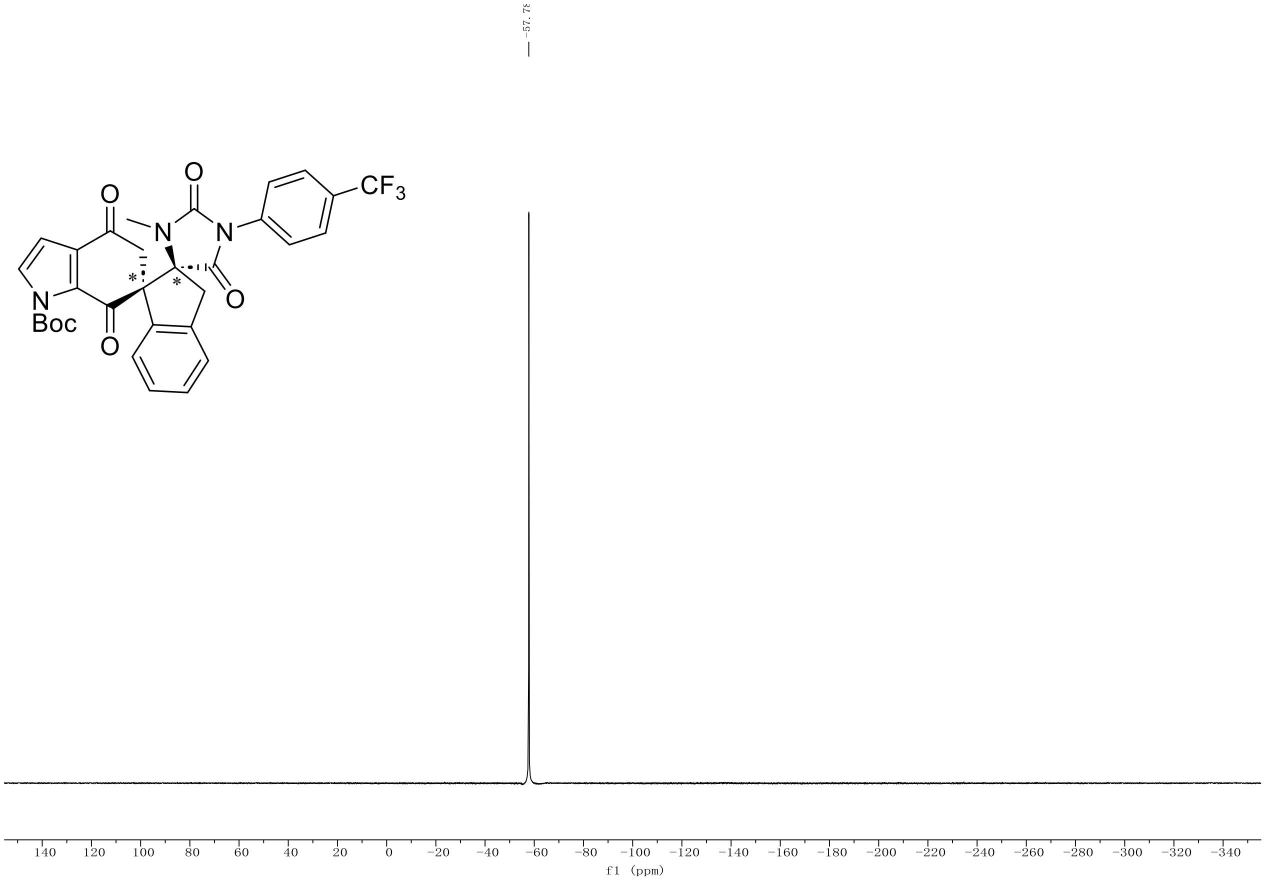

Figure S136: ^{19}F{H} NMR spectra of compound **5'a**

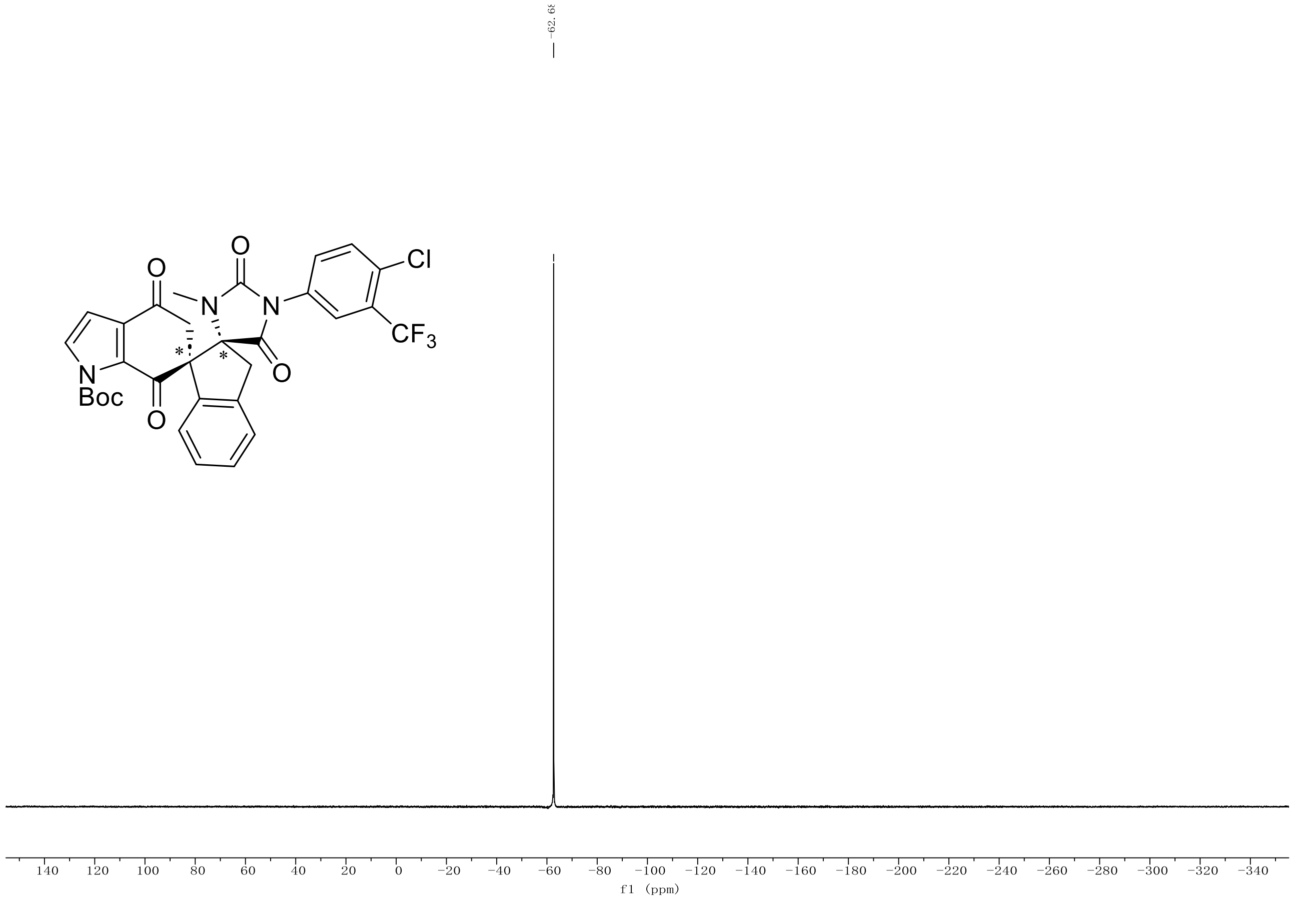

Figure S137: ^{19}F{H} NMR spectra of compound **5c**

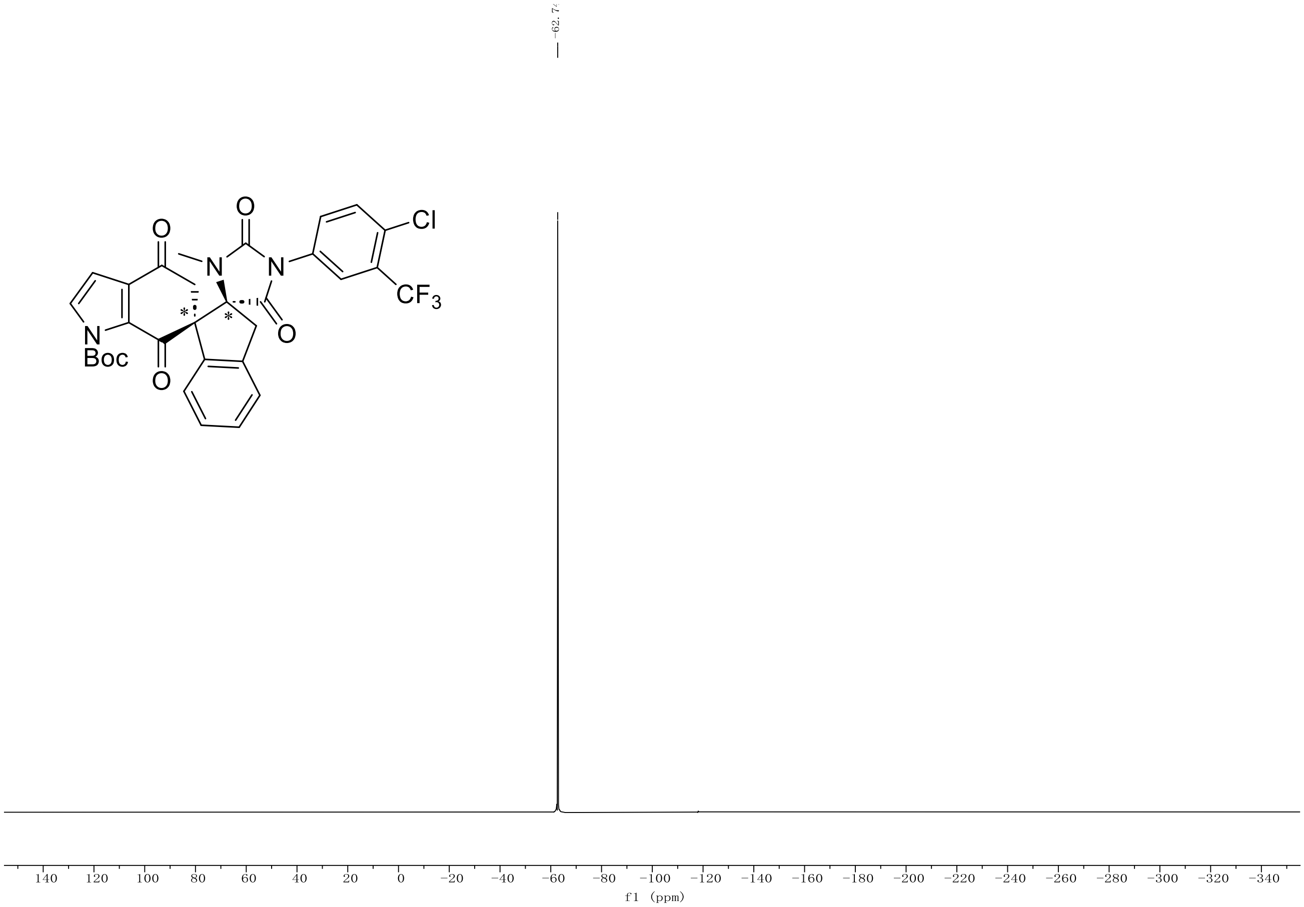

Figure S138: ^{19}F{H} NMR spectra of compound **5'c**

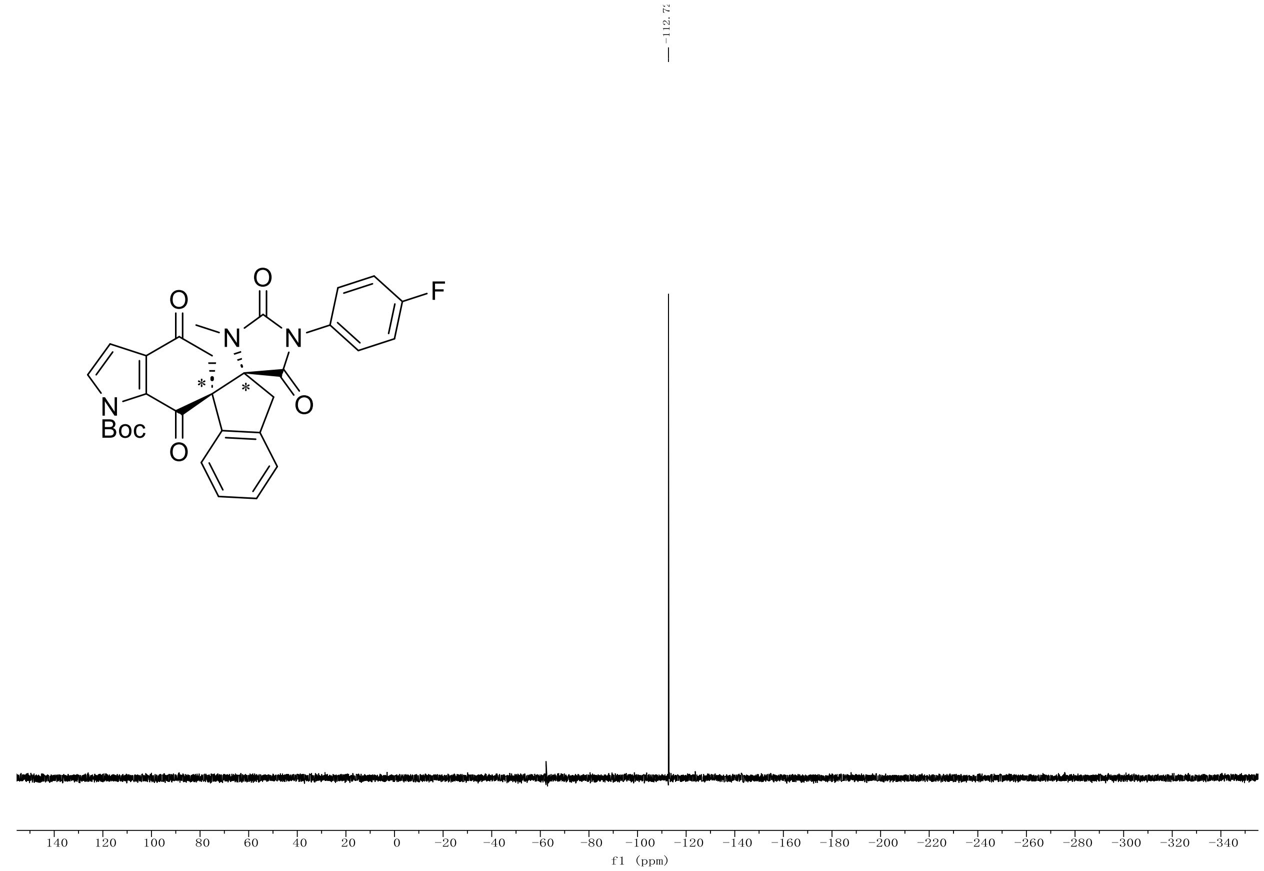

Figure S139: ^{19}F{H} NMR spectra of compound **5d**

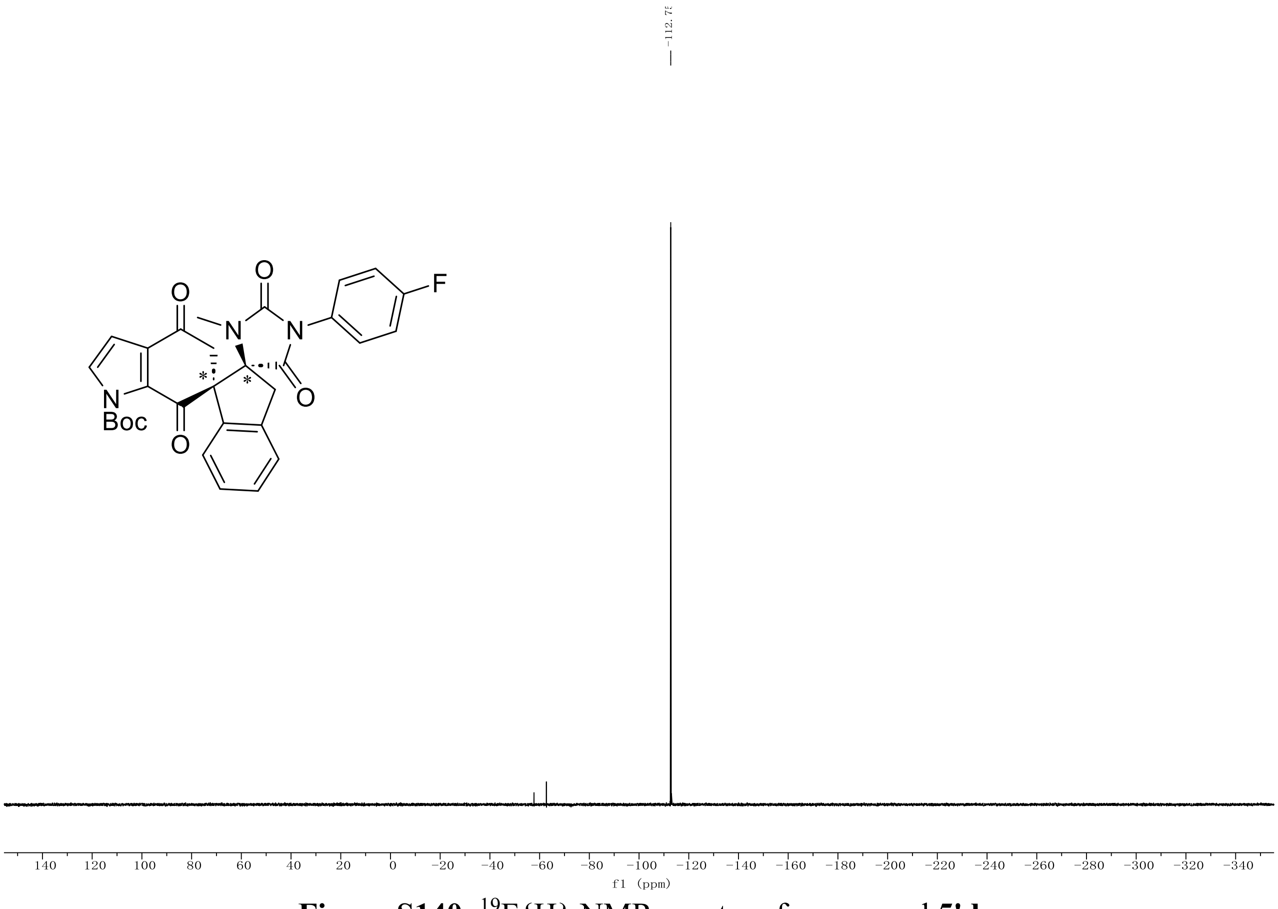

Figure S140: ^{19}F{H} NMR spectra of compound **5'd**

General procedure for the preparation of (±)-2'-methoxy-3-methyl-1-(*p*-tolyl)-5'*H*-spiro[imidazolidine-4,6'-tetraphene]-2,5,7',12'-tetraone **3m**

To a solution of 5-(2-(1,4-dioxo-1,4-dihydronaphthalen-2-yl)-4-methoxybenzyl)-1-methyl-3-(*p*-tolyl)imidazolidine-2,4-dione **1m** (480 mg, 1.0 mmol) in CH_2Cl_2 (5 mL) and CH_3OH (1 mL) was added NaOH (8 mg, 0.2 mmol) at 25 °C under air. Then the reaction was stirred at this temperature for 1h. The mixture was quenched with 1N HCl solution (10 mL), extracted with CH_2Cl_2 (20 mL×3), washed with brine (20 mL), dried over anhydrous Na_2SO_4, and concentrated under reduced pressure. The residue was purified by column chromatography (silica gel, PET/EtOAc=5:1) to give compound **3m** (yellow solid, 392 mg, 82%).

3m: ^{1}H NMR (400 MHz, $CDCl_3$) δ 8.24 – 8.18 (m, 1H), 8.15 – 8.08 (m, 1H), 7.85 (d, *J* = 2.4 Hz, 1H), 7.84 – 7.76 (m, 2H), 7.53 (d, *J* = 8.4 Hz, 2H), 7.34 (d, *J* = 8.4 Hz, 2H), 7.28 (s, 1H), 7.05 (dd, *J* = 8.4, 2.8 Hz, 1H), 3.90 (s, 3H), 3.72 (d, *J* = 16.4 Hz, 1H), 3.13 (d, *J* = 16.4 Hz, 1H), 2.48 (s, 3H), 2.42 (s, 3H); ^{13}C NMR (125MHz, $CDCl_3$) δ 184.1, 182.5, 174.8, 159.1, 154.7, 143.8, 138.4, 135.6, 134.5, 134.4, 132.7, 131.5, 129.9, 129.9 (2C), 129.0, 128.3, 127.2, 126.8, 126.6 (2C), 126.1, 118.1, 116.1, 62.1, 55.7, 38.1, 27.1, 21.4; HRMS (ESI) calcd for $C_{29}H_{23}N_2O_5$ $[M+H]^+$ 479.1601, found 479.1604.

(±)-3'-methoxy-3-methyl-1-(*p*-tolyl)-5'*H*-spiro[imidazolidine-4,6'-tetraphene]-2,5,7',12'-tetraone **3n**

Compound **3n** was prepared according to general procedure. Purification by column chromatography (silica gel, PET/EtOAc = 5:1, *v/v*) generated compound **3n** (yellow solid, 413 mg, 86%).

3n: ^{1}H NMR (500 MHz, $CDCl_3$) δ 8.25 (d, *J* = 9.0 Hz, 1H), 8.22 – 8.16 (m, 1H), 8.13 – 8.08 (m, 1H), 7.81 – 7.75 (m, 2H), 7.54 (d, *J* = 8.0 Hz, 2H), 7.34 (d, *J* = 8.0 Hz, 2H), 6.97 (dd, *J* = 9.0, 3.0 Hz, 1H), 6.88 (s, 1H), 3.91 (s, 3H), 3.78 (d, *J* = 16.5 Hz, 1H), 3.12 (d, *J* = 16.5 Hz, 1H), 2.52 (s, 3H), 2.42 (s, 3H); ^{13}C NMR (100 MHz, $CDCl_3$) δ 184.5, 182.3, 174.9, 162.7, 154.7, 143.9, 138.4, 136.9, 134.4, 134.2, 133.2, 132.8, 132.6, 131.7, 129.9 (3C), 127.1, 126.7, 126.6 (2C), 120.3, 114.1, 113.3, 62.2, 55.7, 39.3, 27.1, 21.4; HRMS (ESI) calcd for $C_{29}H_{23}N_2O_5$ $[M+H]^+$ 479.1601, found 479.1599.

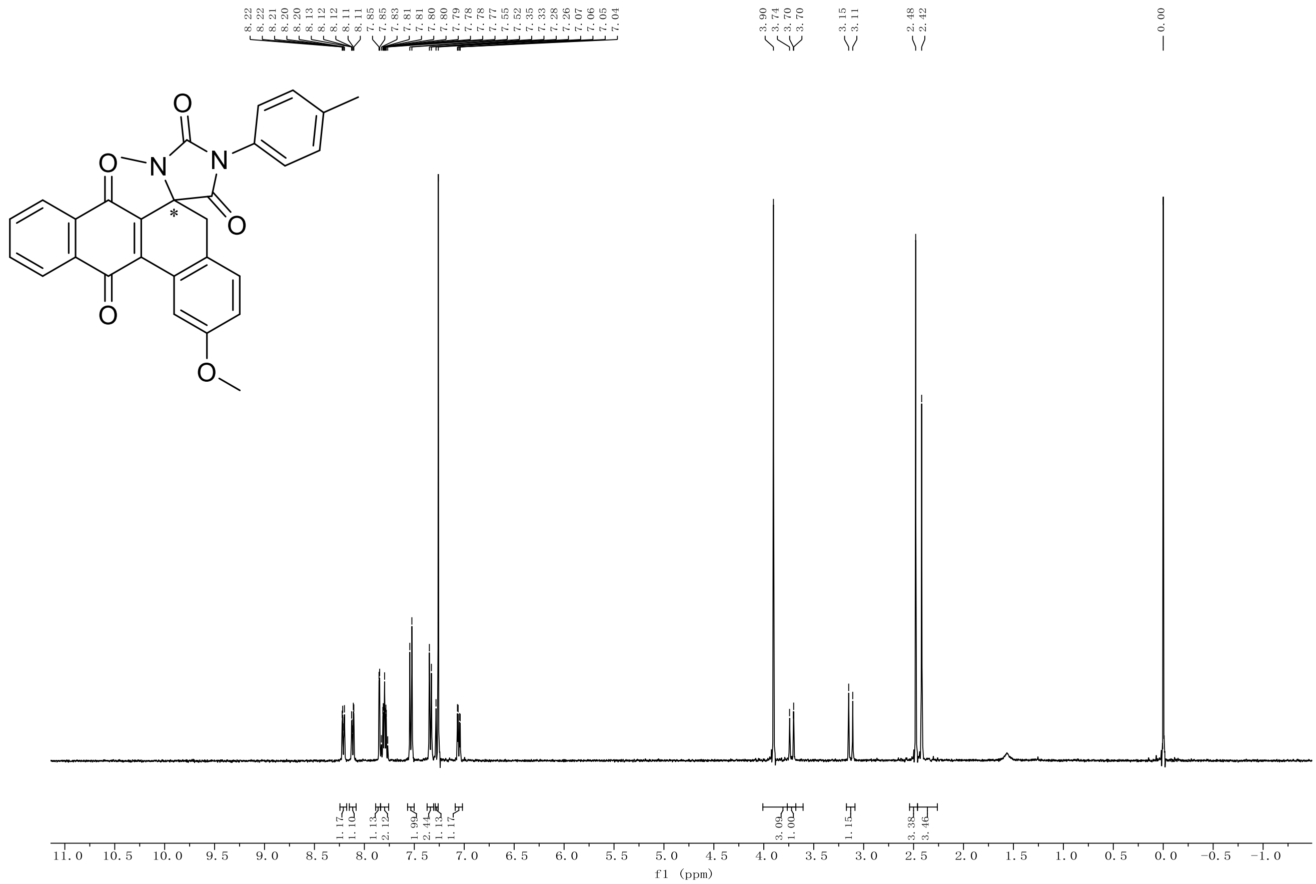

Figure S141: ^{1}H NMR spectra of compound **3m**

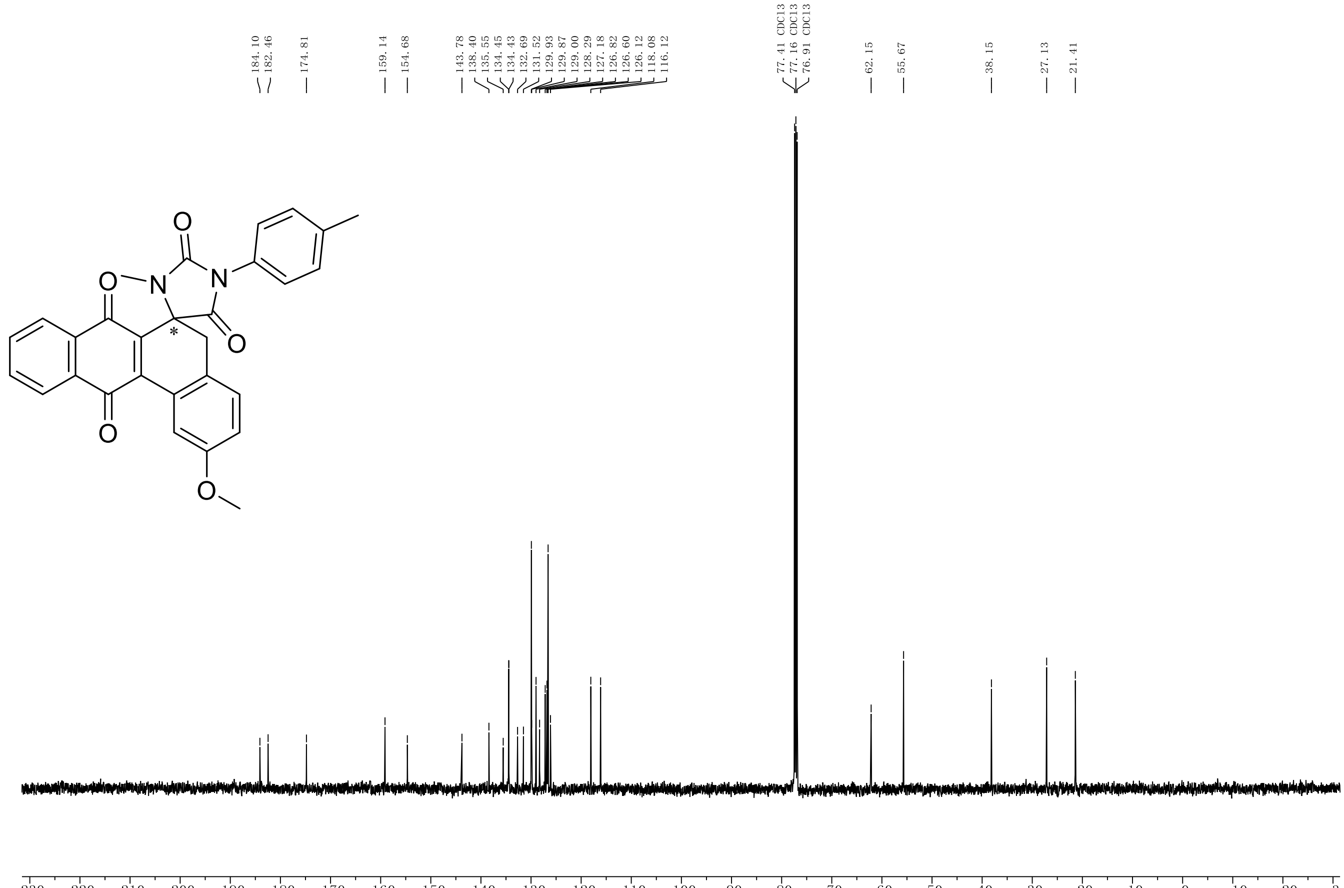

Figure S142: ^{13}C NMR spectra of compound **3m**

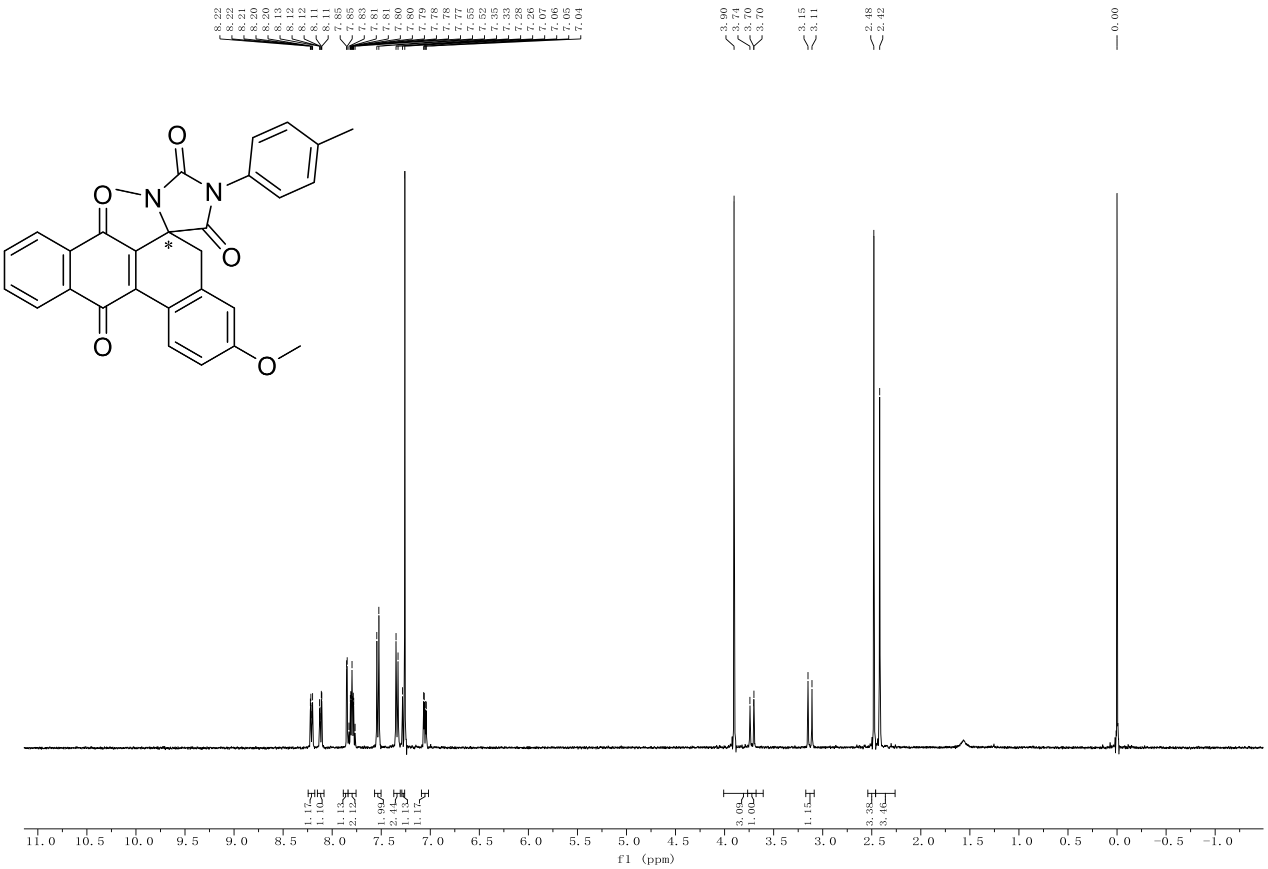

Figure S143: ^{1}H NMR spectra of compound **3n**

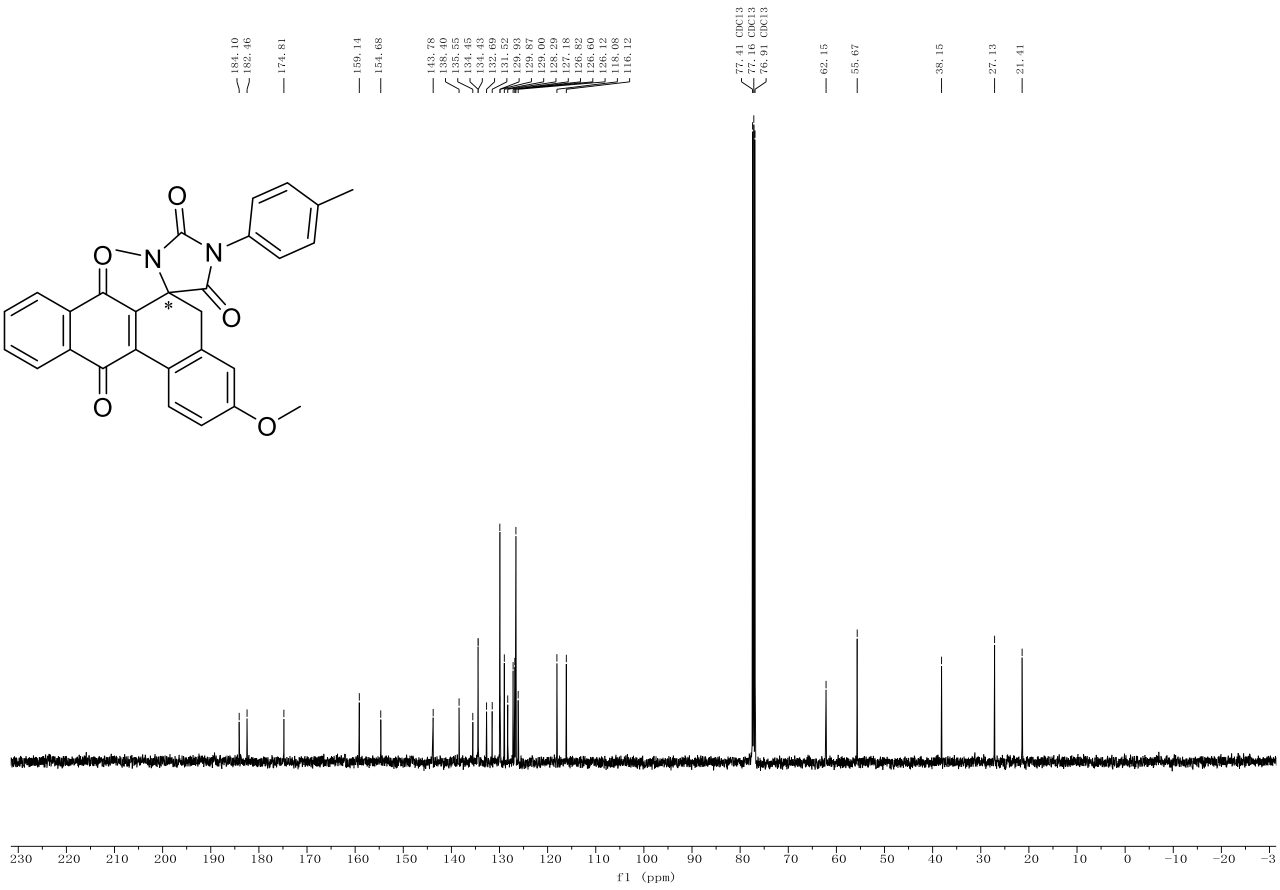

Figure S144: ^{13}C NMR spectra of compound **3n**

Figure S145: Single-crystal X-ray diffraction analysis of **2a**

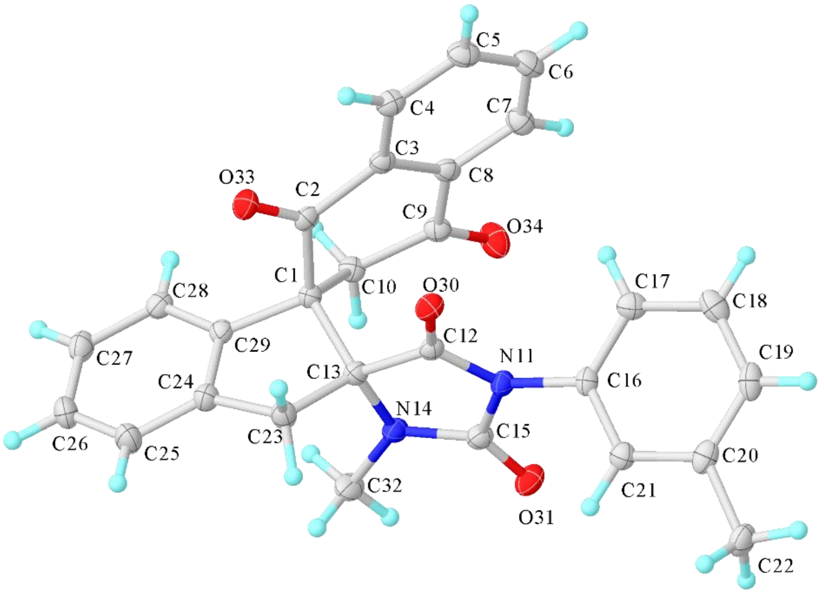

Figure S146: Single-crystal X-ray diffraction analysis of **2'b**

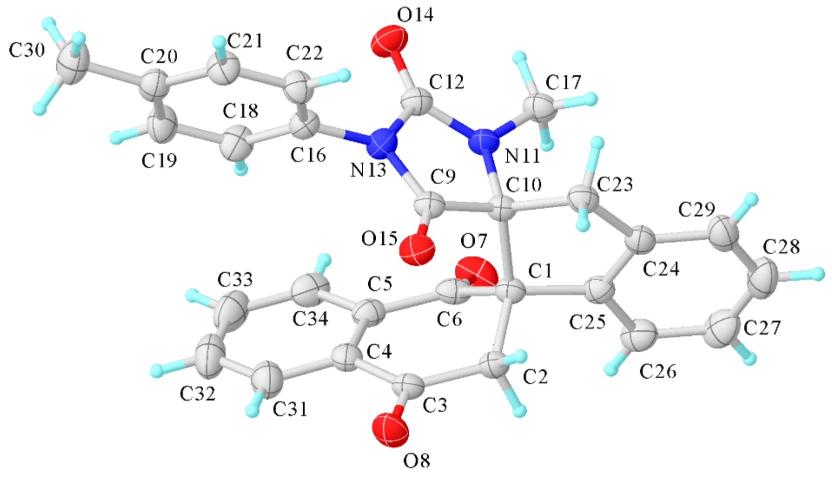

Supplement: Supplementary file 1 [file molecules-30-03164-s001.zip › molecules-3732244-supplementary.pdf]
